# Supplementary material for: Differentiation between enamines and tautomerizable imines in the oxidation reaction with TEMPO
Source: Nat Commun. 2018 Nov 27;9:5002. doi: 10.1038/s41467-018-07534-x (PMC6258700; doi:10.1038/s41467-018-07534-x)
Supplement: Supplementary file 1 — Supplementary Information [file 41467_2018_7534_MOESM1_ESM.pdf]

Supporting Information

Differentiation between enamines and tautomerizable imines in the oxidation reaction with TEMPO

Jie et al.

Supplementary Methods

General Considerations. All reactions were conducted under an atmosphere of nitrogen with dry solvents. Unless otherwise noted, chemical reagents were purchased from commercial supplies and used directly without further purification. Toluene, DME, dioxane, and THF were distilled from metal Na and stored under nitrogen atmosphere. DMF, DMSO, CH_3CN, DCE, *tert*-amyl alcohol and 1,2-dichlorobenzene were distilled over CaH_2 and stored under nitrogen atmosphere. 3Å molecular sieve was dried at 150°C overnight and stored in the nitrogen-filled glove-box. NMR spectra were recorded on a Bruker AVANCE 400 spectrometer using $CDCl_3$ as solutions (^{1}H NMR: 400 MHz, ^{13}C NMR: 100 MHz, ^{19}F NMR: 377 MHz). The chemical shift δ was calibrated using TMS (0 ppm for ^{1}H NMR) and residual undeuterated solvent $CDCl_3$ (77.0 ppm for ^{13}C NMR). HRMS (High resolution mass spectra) were performed by the Shanghai Institute of Organic Chemistry, Chinese Academic of Sciences (Instrument Themo Fisher Scientific LTQFT Ultra, Operated Mode: DART Positive)

Synthesis of ethyl 4-(3-oxocyclohexyl)benzoate

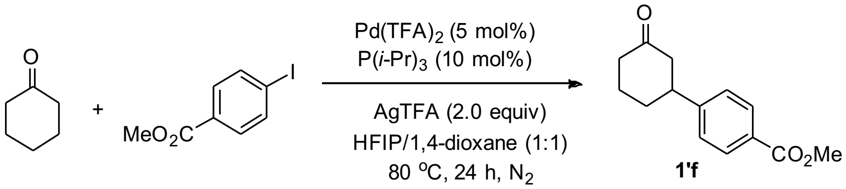

According to the literature[1], in a nitrogen-filled glovebox, the Schlenk tube was charged with methyl 4-iodobenzoate (1.57 g, 6.0 mmol), cyclohexanone (1.4723g, 15 mmol), $Pd(TFA)_2$ (0.1000 g, 0.3 mmol, 5 mol%), AgTFA (2.6500 g, 12 mmol), and P(*i*-Pr)$_3$ (114 μL, 0.60 mmol).The tube was fitted with a rubber septum and move out of the glove box. Then HFIP/1,4-dioxane (7.5 mL/7.5 mL) was added to the tube, and the septum was replaced with a Teflon screwcap under nitrogen flow. The reaction mixture was stirred at 80 °C for 24 h. After the reaction mixture was cooled to room temperature, the reaction mixture was filtered through a pad of silica gel and

washed with ethyl acetate. The filtrate was concentrated under reduced pressure and the residue was purified by flash chromatography on silica gel (eluent = petroleum ether/ Et_2O = 100:8) obtained the product **1’f** as white solid. **^{1}H NMR** (400 MHz, $CDCl_3$): δ 8.00 (d, J = 8.3 Hz, 2H), 7.29 (d, J = 8.3 Hz, 2H), 3.91 (s, 3H), 3.12-3.04 (m, 1H), 2.62-2.35 (m, 4H), 2.205-2.08 (m, 2H), 1.93-1.75 (m, 2H); **^{13}C NMR** (100 MHz, $CDCl_3$): δ 210.3, 166.8, 149.4, 130.0, 128.6, 126.6, 52.0, 48.4, 44.6, 41.1, 32.4, 25.4.

Synthesis of *N*-phenylcyclohexanimine

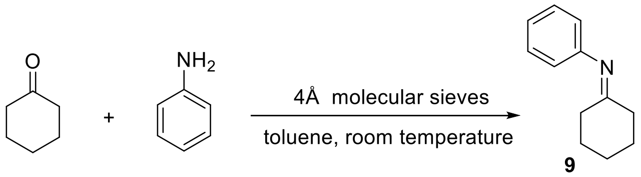

9

According to the literature[2], to a 150 mL round bottom flask was charged with aniline (5.0000 g, 53.69 mmol), cyclohexanone (6.3230 g, 64.42 mmol) 4Å molecular sieves (15 g) in toluene (20.0 mL). The round flask was fitted with a rubber septum, and the reaction mixture was stirred at room temperature for 2 days. After the reaction was finished, the solution was filtered through celite, and the filtrate was concentrated under reduced pressure. The crude residue was distilled to provide the product **9** as slightly yellow oil. **^{1}H NMR** (400 MHz, $CDCl_3$): δ 7.30-7.26 (m, 2H), 7.05-7.01 (m, 1H), 6.73-6.70 (m, 2H), 2.46 (t, J = 6.4 Hz, 2H), 2.19-2.16 (m, 2H), 1.89-1.83 (m, 2H), 1.70-1.60 (m, 4H); **^{13}C NMR** (100 MHz, $CDCl_3$): δ 175.0, 150.7, 128.7, 122.9, 119.8, 39.4, 31.2, 27.8, 27.6, 25.7.

Synthesis of 4-(cyclohex-1-en-1-yl)morpholine

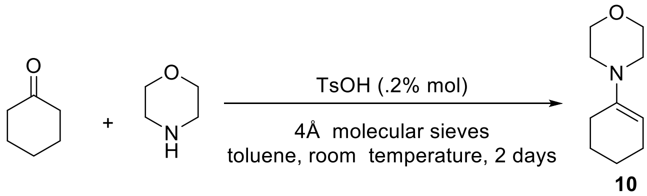

10

According to the literature[3], to a 150 mL round bottom flask was charged with cyclohexanone (4.9075 g, 60.0 mmol), morpholine (5.2272 g, 60.0 mmol),

p-toluenesulfonic acid (0.1000 g, 1.2 mol%) and 4Å molecular sieves (15 g) in toluene (20.0 mL). The round flask was fitted with a rubber septum, and the reaction mixture was stirred at room temperature for 2 days. After the reaction was finished, the solution was filtered through celite, and the filtrate was concentrated under reduced pressure. The crude residue was distilled to provide the product **10** as slightly yellow oil. ^{1}**H NMR** (400 MHz, $CDCl_3$): δ 4.68 (t, J = 3.7 Hz, 1H), 3.74 (t, J = 4.8 Hz, 4H), 2.78 (t, J = 4.8 Hz, 4H), 2.10-2.03 (m, 4H), 1.71-1.65 (m, 2H), 1.59-1.53 (m, 2H); ^{13}**C NMR** (100 MHz, $CDCl_3$): δ 145.4, 100.4, 67.0, 48.4, 26.8, 24.4, 23.2, 22.7.

Supplementary Table 1. The optimization of *α*-amino-enone formation from primary amine.[a]

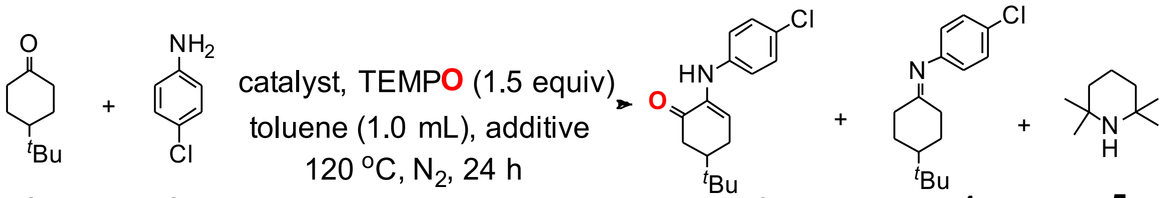

entry	catalyst (10 mol%)	additive	yield of **3a** (%)[b]	conv. of TEMPO (%)[c]	yield of **4** (%)[b]	yield of **5** (%)[c]
1	$Cu(OAc)_2$, bpy	-	63	96	trace	94
2	$Cu(OAc)_2$	-	69	97	trace	94
3	$Cu(OTf)_2$	-	36	72	16	46
4	$Sc(OTf)_3$	-	11	73	33	15
5	$Yb(OTf)_3$	-	9	71	38	15
6	$Zn(OTf)_2$	-	10	71	34	17
7	TsOH	-	19	70	32	23
8	$AlCl_3$	-	48	95	trace	73
9	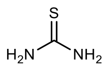	-	15	81	21	45
10	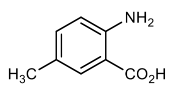	-	37	78	20	43

11	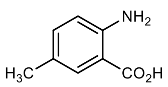	4Å MS (400 mg)	73	100	9	94
12	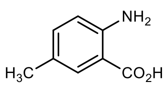	3Å MS (400 mg)	77	100	11	92
**13**[d]	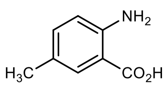	**3Å MS (400 mg)**	**86**	**100**	**trace**	**91**
14	-	3Å MS (400 mg)	n.d.	65	40	44

[a] Reaction conditions: **1a** (0.3 mmol), **2a** (0.2 mmol), catalyst (10 mol%), additive, solvent (1.0 mL), N_2, 120 °C for 24 h. [b] Yields were determined by GC analysis using dodecane as an internal standard. [c] Conversion of TEMPO and yield of **5** were based on the amount of TEMPO and determined by GC analysis. [d] 15 mol% catalyst was used.

Supplementary Table 2. The optimization of arylamine formation from secondary amine.[a]

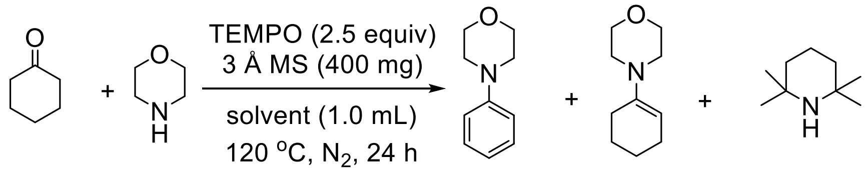

1a **6a** **7a** **8** **5**

entry	solvent	additive (mol%)	yield of **7a** (%)[b]	conv. of TEMPO (%)[c]	yield of **8** (%)[b]	yield of **5** (%)[c]
1	toluene	4-Methylanthranilic acid (15)	44	97	19	47
2	toluene	TsOH (15)	35	68	7	47
3	toluene	$PhCO_2H$ (15)	32	76	trace	trace
4	toluene	CCl_3CO_2H (15)	45	71	8	52
5	toluene	LiOAc (100)	57	67	13	43

6	toluene	NaOAc (100)	59	67	15	39
7	toluene	KOAc (100)	59	69	11	42
8	toluene	CsOAc (100)	56	71	21	trace
9	toluene	Et_3N (100)	53	67	18	37
**10**	**toluene**	**-**	**62**	**64**	**23**	**36**
11	dioxane	-	57	65	24	21
12	THF	-	53	61	40	17
13	DMF	-	41	59	37	trace
14	DMSO	-	28	52	43	trace
15	*o*-dichlorobenzene	-	50	67	11	44
16	CH_3CN	-	52	51	13	39
17	DCE	-	n.d.	85	0	22
18	tert-amyl alcohol	-	50	64	33	21

[a] Reaction conditions: **1a** (0.2 mmol), **6a** (0.3 mmol), additive (3Å MS 400 mg), solvent (1.0 mL), N_2, 120 °C for 24 h. [b] Yields were determined by GC analysis using dodecane as an internal standard. [c] Conversion of TEMPO and yield of **5** were based on the amount of TEMPO and determined by GC analysis.

Supplementary Table 3. The effect of TEMPO equivalents on the reaction outcome for the *α*-amino enone formation reaction.

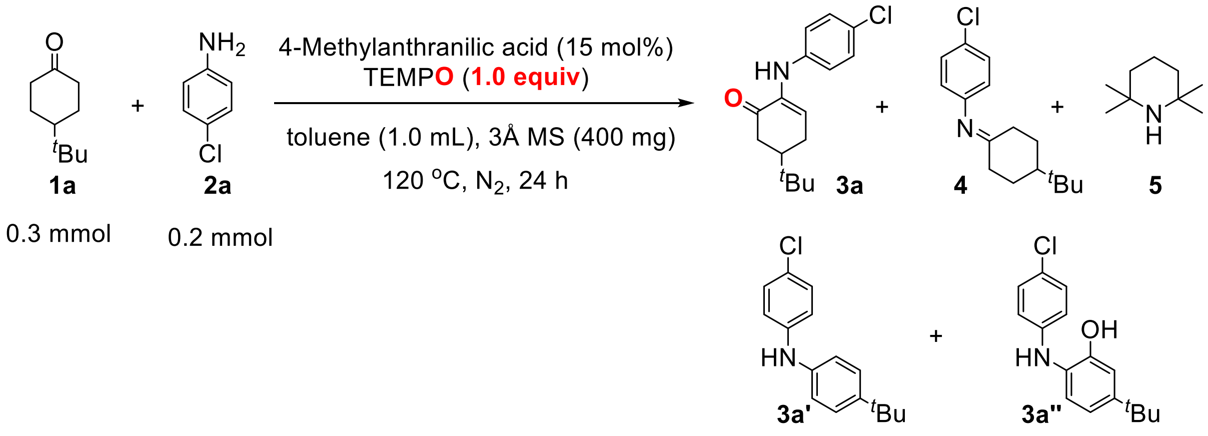

conv. of TEMPO[a,b]	yield of **3a** (%)[a]	yield of **5** (%)[a,b]	yield of **4** (%)[a]	yield of **3a’** (%)[a]	yield of **3a”** (%)[a]
100	58	86	34	6	trace

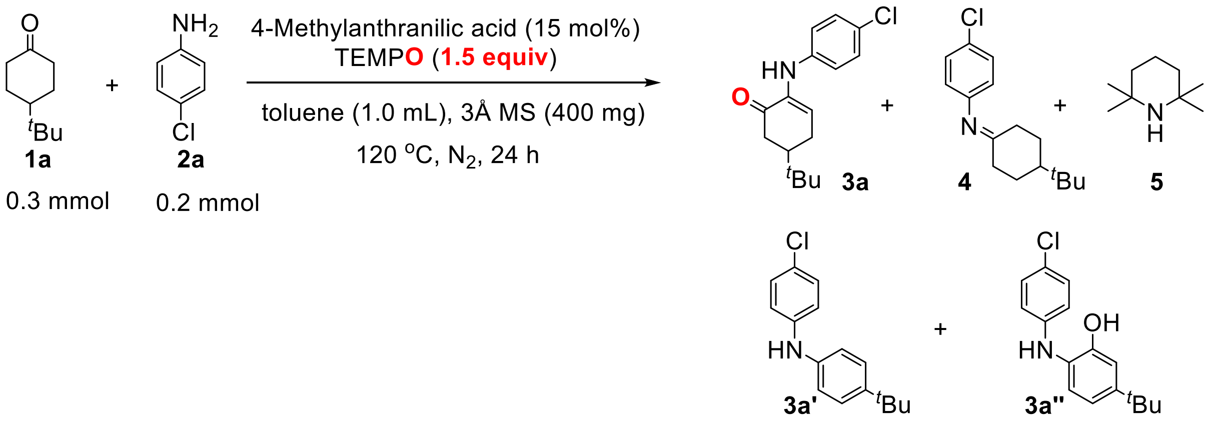

conv. of TEMPO[a,b]	yield of **3a** (%)[a]	yield of **5** (%)[a,b]	yield of **4** (%)[a]	yield of **3a’** (%)[a]	yield of **3a”** (%)[a]
100	86	91	trace	trace	trace

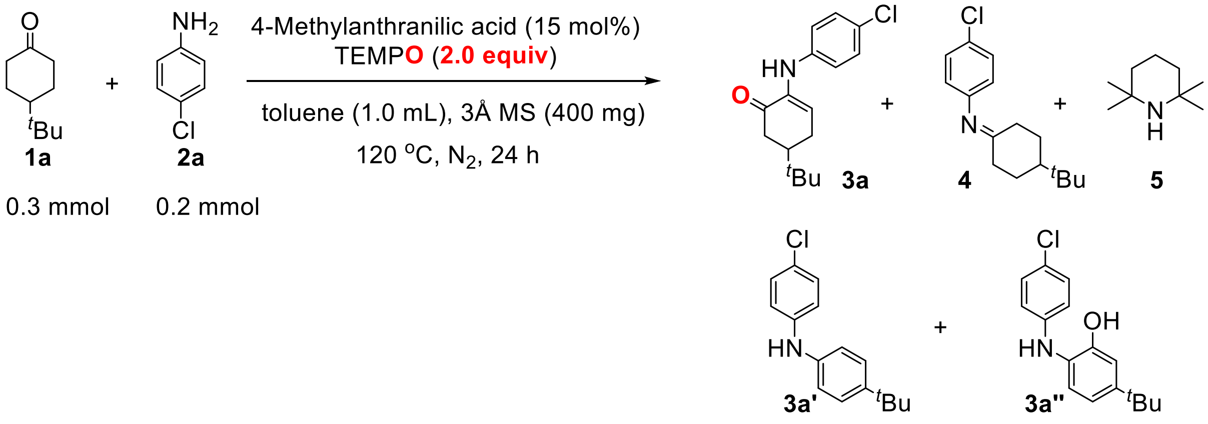

conv. of TEMPO[a,b]	yield of **3a** (%)[a]	yield of **5** (%)[a,b]	yield of **4** (%)[a]	yield of **3a’** (%)[a]	yield of **3a”** (%)[a]
97	81	89	trace	trace	trace

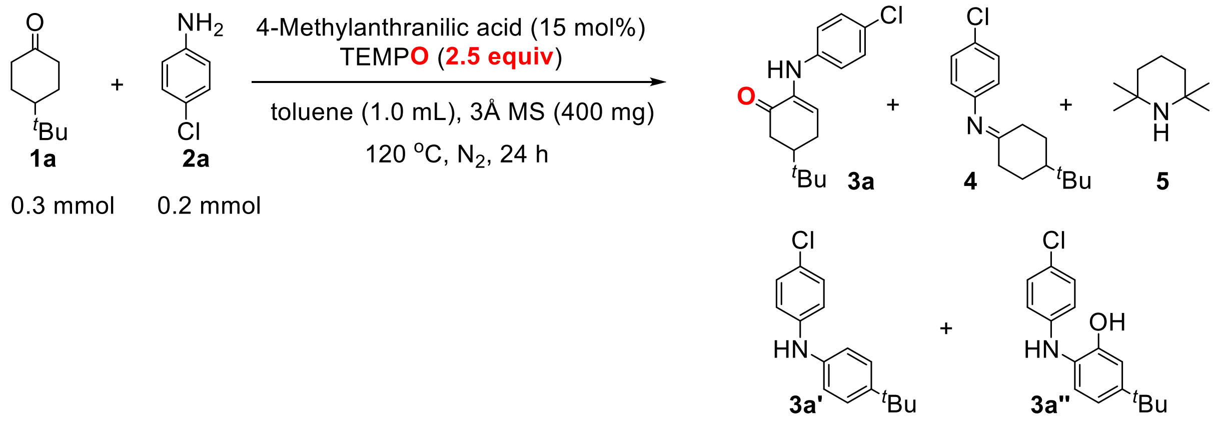

conv. of TEMPO[a,b]	yield of **3a** (%)[a]	yield of **5** (%)[a,b]	yield of **4** (%)[a]	yield of **3a'** (%)[a]	yield of **3a"** (%)[a]
97	18	89	trace	7	20

[a] Reaction conditions: **1a** (0.3 mmol), **2a** (0.2 mmol), 4-methylanthranilic acid (15 mol%), 3Å MS (400 mg), TEMPO (1.0 – 2.5 equiv.), toluene (1.0 mL), N_2, 120 °C for 24 h. Yields were determined by GC analysis using dodecane as an internal standard. [b] Conversion of TEMPO and yields of **5** were based on the scale of TEMPO.

Supplementary Table 4. The effect of TEMPO equivalents on the reaction outcome for the arylamine formation reaction.

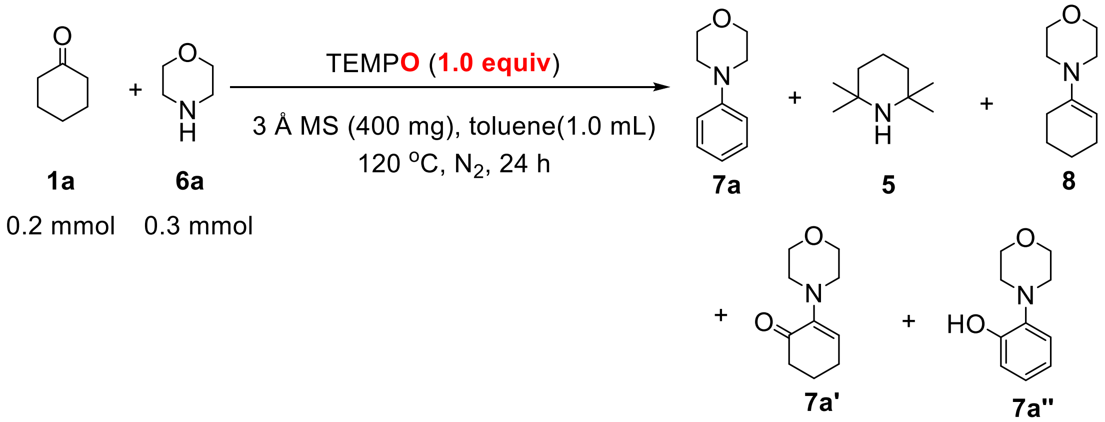

conv. of TEMPO[a,b]	yield of **7a**(%)[a]	yield of **5** (%)[a,b]	yield of **8** (%)[a]	yield of **7a'** (%)[a]	yield of **7a"** (%)[a]
80	19	53	22	trace	trace

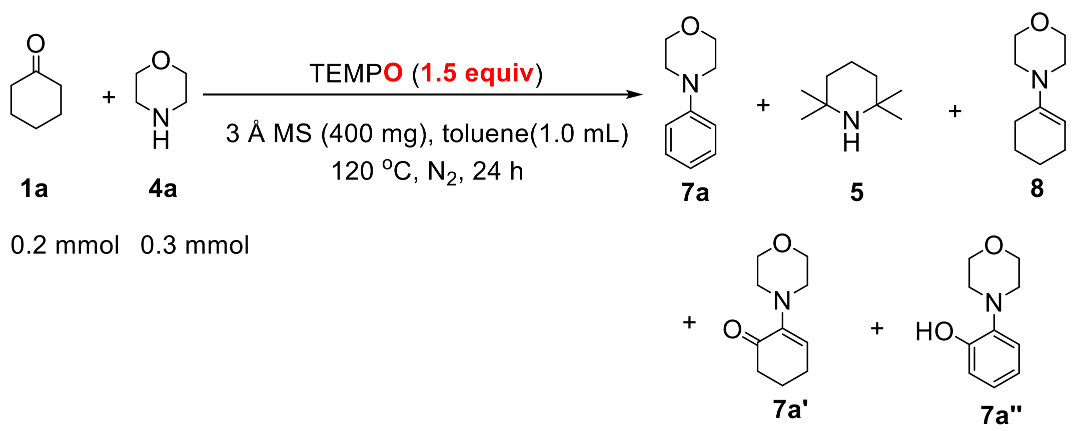

conv. of TEMPO[a,b]	yield of **7a**(%)[a]	yield of **5** (%)[a,b]	yield of **8** (%)[a]	yield of **7a’** (%)[a]	yield of **7a”** (%)[a]
75	32	46	26	trace	trace

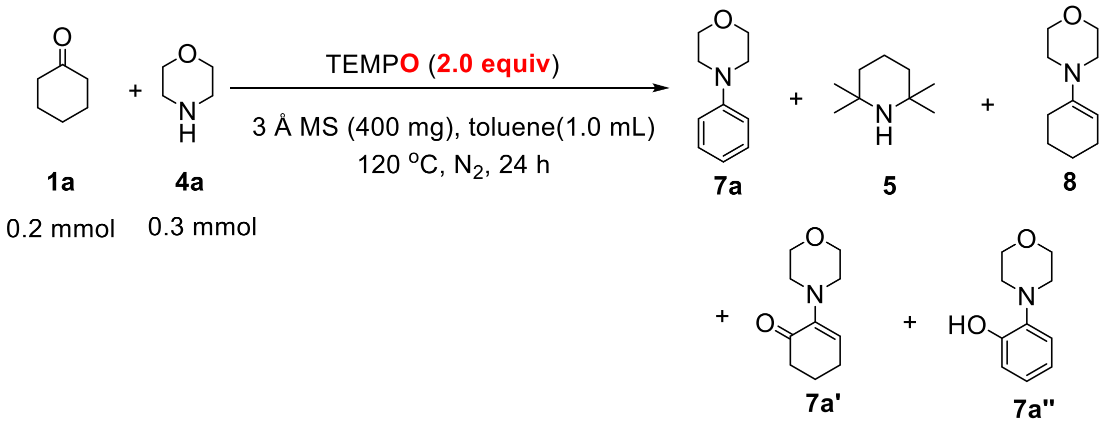

conv. of TEMPO[a,b]	yield of **7a**(%)[a]	yield of **5** (%)[a,b]	yield of **8** (%)[a]	yield of **7a’** (%)[a]	yield of **7a”** (%)[a]
66	45	35	28	trace	trace

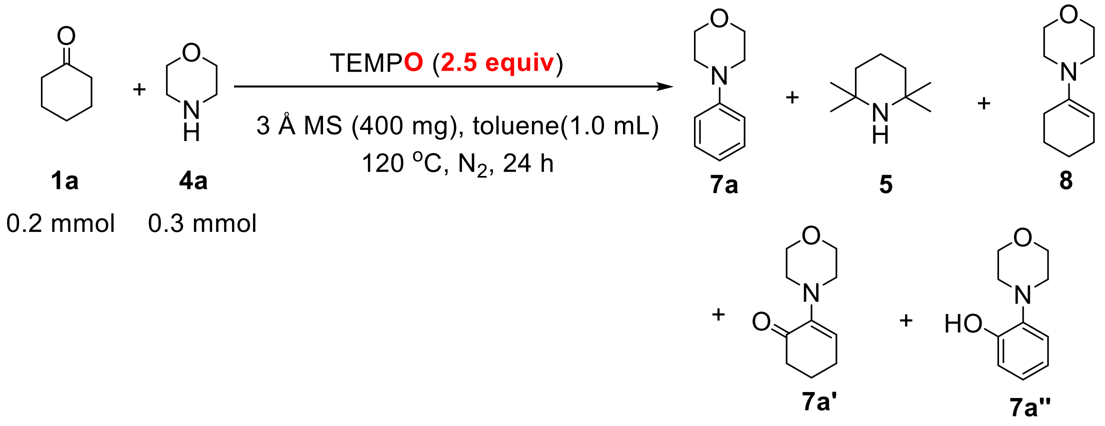

conv. of TEMPO[a,b]	yield of **7a**(%)[a]	yield of **5** (%)[a,b]	yield of **8** (%)[a]	yield of **7a’** (%)[a]	yield of **7a”** (%)[a]
64	62	36	23	trace	trace

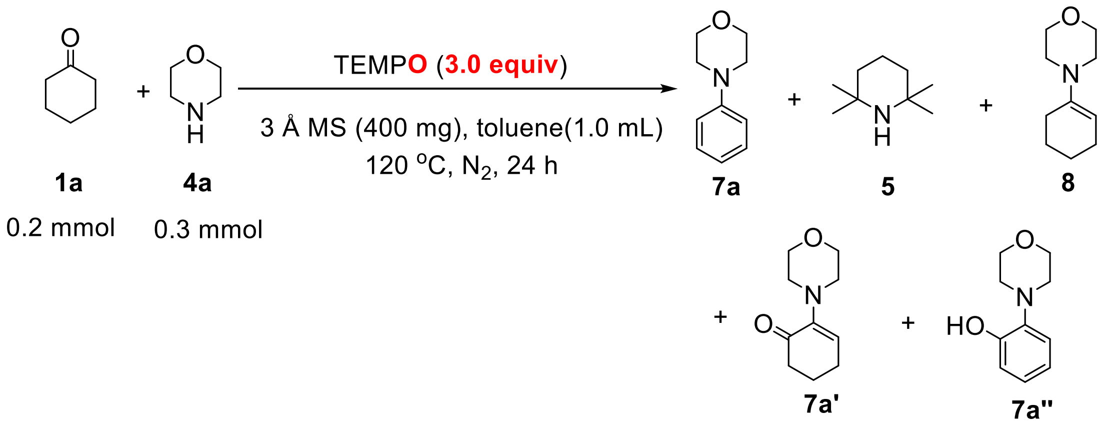

conv. of TEMPO[a,b]	yield of **7a**(%)[a]	yield of **5** (%)[a,b]	yield of **8** (%)[a]	yield of **7a’** (%)[a]	yield of **7a”** (%)[a]
63	65	35	20	trace	trace

[a] Reaction conditions: **1a** (0.2 mmol), **4a** (0.3 mmol), 3Å MS (400 mg), TEMPO (1.0 – 3.0 equiv.), toluene (1.0 mL), N_2, 120 °C for 24 h. Yields were determined by GC analysis using dodecane as an internal standard. [b] Conversion of TEMPO and yields of **5** were based on the scale of TEMPO.

Treatment of the pre-synthesized imine with TEMPO at different conditions.

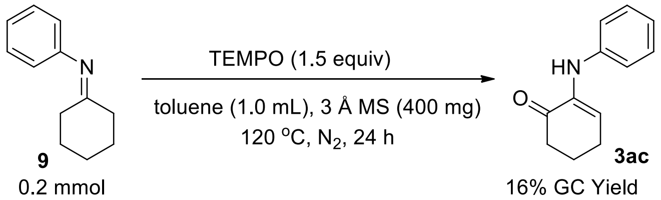

(eq. 1)

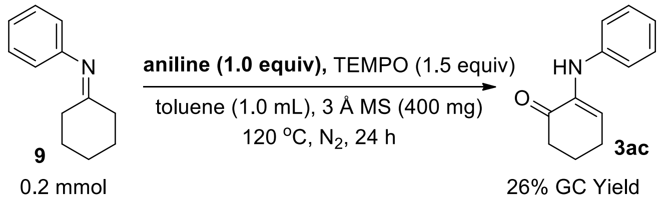

(eq. 2)

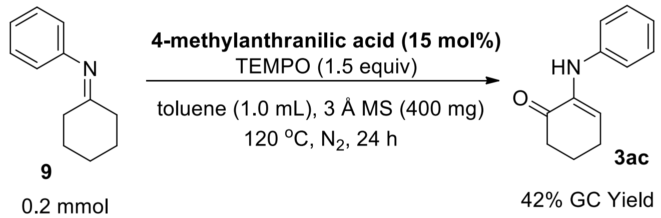

(eq. 3)

(1) In the Supplementary Equation 1, the reaction was conducted with **9** (0.0346g, 0.2 mmol), TEMPO (0.0468g, 0.3 mmol) and 3Å molecular sieve (400 mg) in toluene (1.0 mL) at 120 °C for 24 h. Then the reaction was analyzed by GC using dodecane as an internal standard. GC yields (**3ac**) were reported in the Supplementary Equation 1.

(2) In the Supplementary Equation 2, the reaction was conducted with **9** (0.0346g, 0.2 mmol), TEMPO (0.0468g, 0.3 mmol), aniline (0.0186g, 0.2 mmol), and 3Å molecular sieve (400 mg) in toluene (1.0 mL) at 120 °C for 24 h. Then the reaction was analyzed by GC using dodecane as an internal standard. GC yields (**3ac**) were reported in the Supplementary Equation 2.

(3) In the Supplementary Equation 3, the reaction was conducted with **9** (0.0346g, 0.2 mmol), TEMPO (0.0468g, 0.3 mmol), 4-methylanthranilic acid (0.0046g, 0.03 mmol) and 3Å molecular sieve (400 mg) in toluene (1.0 mL) at 120 °C for 24 h. Then the reaction was analyzed by GC using dodecane as an internal standard. GC yields (**3ac**) were reported in the Supplementary Equation 3.

Treatment of the pre-synthesized enamine with TEMPO at different conditions.

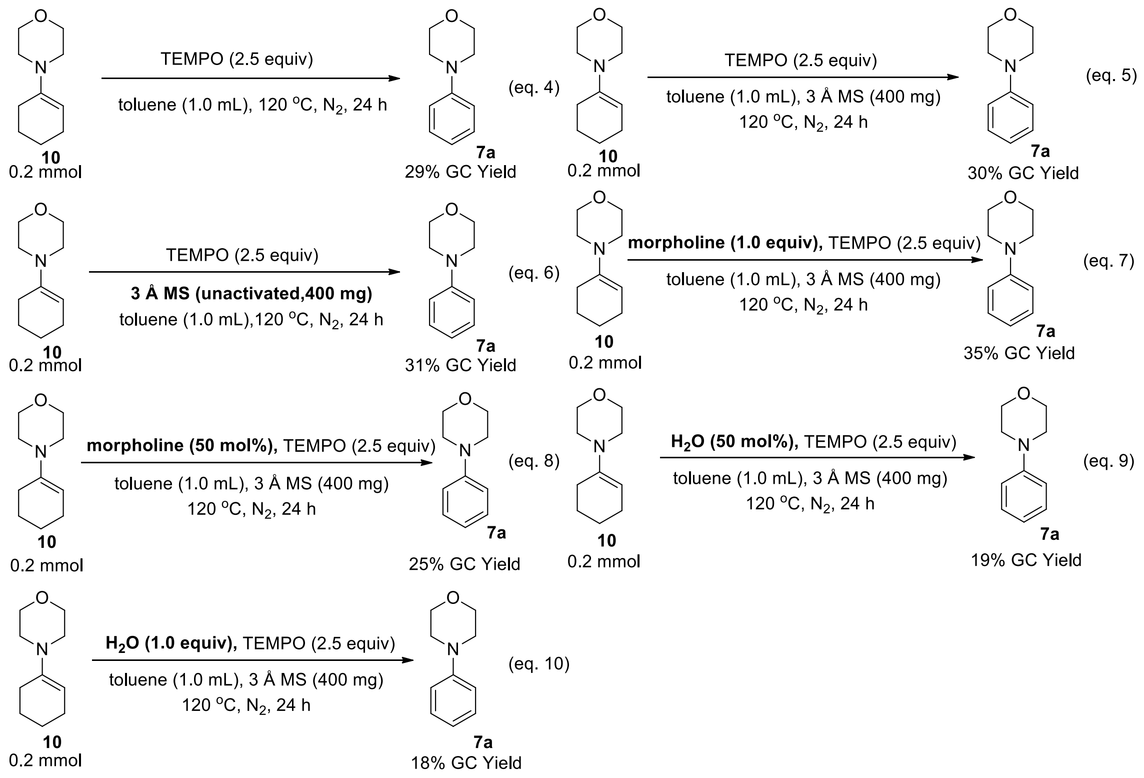

(1) In the Supplementary Equation 4, the reaction was conducted with **9** (0.0335g, 0.2 mmol) and TEMPO (0.0780g, 0.5 mmol) in toluene (1.0 mL) at 120 °C for 24 h. Then the reaction was analyzed by GC using dodecane as an internal standard. GC yields (**7a**) were reported in the Supplementary Equation 4.

(2) In the Supplementary Equation 5, the reaction was conducted with **9** (0.0335g, 0.2 mmol), TEMPO (0.0780g, 0.5 mmol) and 3Å molecular sieve (400 mg) in toluene (1.0 mL) at 120 °C for 24 h. Then the reaction was analyzed by GC using dodecane as an internal standard. GC yields (**7a**) were reported in the Supplementary Equation 5.

(3) In the Supplementary Equation 6, the reaction was conducted with **9** (0.0335g, 0.2 mmol), TEMPO (0.0780g, 0.5 mmol) and 3Å molecular sieve (unactivated, 400 mg) in toluene (1.0 mL) at 120 °C for 24 h. Then the reaction was analyzed by GC using dodecane as an internal standard. GC yields (**7a**) were reported in the Supplementary Equation 6.

(4) In the Supplementary Equation 7, the reaction was conducted with **9** (0.0335g, 0.2 mmol), morpholine (0.0174g, 0.2 mmol), TEMPO (0.0780g, 0.5 mmol) and 3Å molecular sieve (400 mg) in toluene (1.0 mL) at 120 °C for 24 h. Then the reaction was analyzed by GC using dodecane as an internal standard. GC yields (**7a**) were reported in the Supplementary Equation 7.

(5) In the Supplementary Equation 8, the reaction was conducted with **9** (0.0335g, 0.2 mmol), morpholine (0.00087g, 0.1 mmol), TEMPO (0.0780g, 0.5 mmol) and 3Å molecular sieve (400 mg) in toluene (1.0 mL) at 120 °C for 24 h. Then the reaction was analyzed by GC using dodecane as an internal standard. GC yields (**7a**) were reported in the Supplementary Equation 8.

(6) In the Supplementary Equation 9, the reaction was conducted with **9** (0.0335g, 0.2 mmol), H_2O (0.0018g, 0.1 mmol), TEMPO (0.0780g, 0.5 mmol) and 3Å molecular sieve (400 mg) in toluene (1.0 mL) at 120 °C for 24 h. Then the reaction was analyzed by GC using dodecane as an internal standard. GC yields (**7a**) were reported in the Supplementary Equation 9.

(7) In the Supplementary Equation 10, the reaction was conducted with **9** (0.0335g, 0.2 mmol), H_2O (0.0036g, 0.2 mmol), TEMPO (0.0780g, 0.5 mmol) and 3Å molecular sieve (400 mg) in toluene (1.0 mL) at 120 °C for 24 h. Then the reaction was analyzed by GC using dodecane as an internal standard. GC yields (**7a**) were reported in the Supplementary Equation 10.

Identification of *α*-TEMPO-Substituted Imine Intermediate by NMR

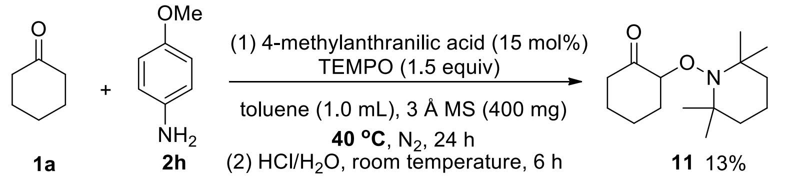

(eq. 11)

In order to directly observe the formation of *α*-TEMPO-substituted imine intermediate **11**, the reaction was conducted with **1a** (0.0294g, 0.3 mmol), *p*-anisidine (0.0246g, 0.2 mmol), 4-methylanthranilic acid (0.0046g, 0.03 mmol), 3Å molecular sieve (400 mg) and TEMPO (0.0468g, 0.3 mmol) in toluene (1.0 mL) at 40 °C for 24 h. After cooling to room temperature, 15 % aqueous HCl solution (2.0 mL) was added to the reaction mixture and stirred for 4 h at room temperature. When the reaction was finished, the reaction mixture was quenched with water (15 mL) and extracted with ether (15 mL) three times. The combined organic layer was dried with anhydrous $NaSO_4$, followed by evaporation under reduced pressure to remove the solvent. The residue was purified by flash chromatography on silica gel (eluent = petroleum ether/ Et_2O = 100:2) to obtain 6.5 mg of isolated product **11**[4] (13%). **^{1}H NMR** (400 MHz, $CDCl_3$): δ 4.17-4.15 (m, 1H), 2.80-2.73 (m, 1H), 2.29-2.23 (m, 1H), 2.15-2.08 (m, 1H), 2.05-1.97 (m, 1H), 1.95-1.86 (m, 2H), 1.80-1.73 (m, 1H), 1.65-1.57 (m, 2H), 1.45-1.44 (m, 4H), 1.32-1.25 (m, 1H), 1.17-1.14 (m, 9H), 0.99 (s, 3H); **^{13}C NMR** (100 MHz, $CDCl_3$): δ 211.7, 89.1, 59.8, 59.6, 40.8, 40.1, 34.7, 33.9, 33.4, 28.4, 22.0, 20.1, 17.0.

Synthesis of TEMP^{18}O

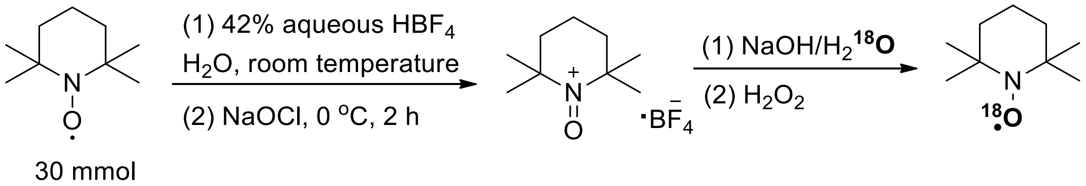

(1) To a solution of TEMPO (4.68g, 30 mmol) in H_2O (15 mL, 2M) was added dropwise 42% aqueous HBF_4 (14.9 mL, 30 mol) at room temperature. After the solution became to amber color, the aqueous NaOCl solution (16.0 mL, 30 mmol) was added dropwise at 0 °C. When it finished, the reaction mixture stirred for additional 1 h at 0 °C. Finally, the reaction mixture was filtered and the yellow crystalline precipitate was washed with ice-cold 5% aqueous $NaHCO_3$ (6.0 ml), water (6.0 mL), and ice-clod ether (60.0 mL). The bright yellow solid was dried at 50 °C in vacuo to gain the $TEMPO^+BF_4^-$ (5.1 g, 70 %).[5]

(2) To the solution of TEMPO$^+$BF$_4^-$ (0.9710g, 4 mmol) in H_2**^{18}O** (1.7 mL) was added concentrated NaOH (12N, 1.5 mL H_2**O^{18}**) at 0 °C for 2 h and the color of solution was changed from orange to slightly yellow. Then, 30% H_2O_2 (0.2 mL) was added to the reaction mixture. When the color of reaction mixture became slightly red, the reaction mixture was extracted with ether. The organic layer was dried over anhydrous Na_2SO_4, filtered and concentrated under reduced pressure to gain the red crystalline solid (TEMP**^{18}O**), which was dried at room temperature in vacuo. [6]

The ratio of TEMP^{18}O/TEMP^{16}O was 1:0.201 determined by the HRMS analysis.

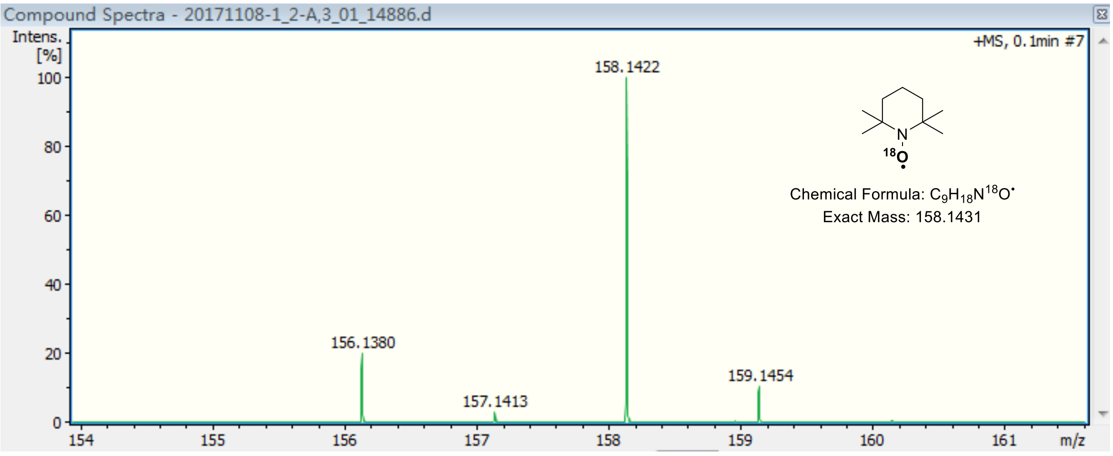

Supplementary Figure 1. The HRMS spectra of TEMP^{18}O.

The ^{18}O-Labeling Experiment

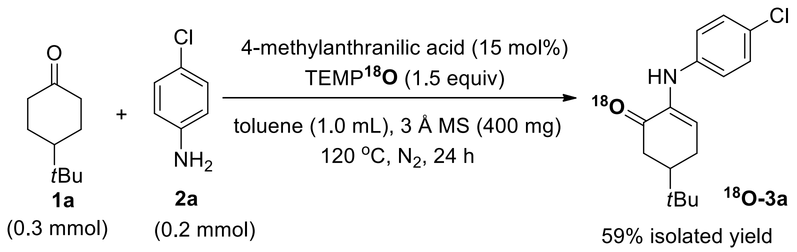

In order to confirm the oxygen source of the α-amino enone, the model reaction was choosen to conduct the ^{18}O-labeling experiment. The reaction was carried with 4-(tert-butyl)cyclohexanone (0.0463, 0.3 mmol), 4-chloroaniline (0.0260g, 0.2 mmol), 4-methylanthranilic acid (0.0046g, 0.03 mmol), 3Å molecular sieve (400 mg)

and TEMP^{18}O (0.0474g, 0.3 mmol) in toluene (1.0 mL) at 120 °C for 24 h. After cooling to room temperature, the reaction mixture was filtered through a pad of silica gel and washed with 10 mL of ethyl acetate. The filtrate was concentrated under reduced pressure and purified by flash chromatography on silica gel (eluent = petroleum ether/ Et_2O = 100:5), the desired ^{18}O-enriched product of **^{18}O-3a** was obtained in 59% (33.0 mg). Isotopic distribution of **^{18}O-3a** and **^{16}O-3a** was analyzed by HRMS, with the ratio 1: 0.256.

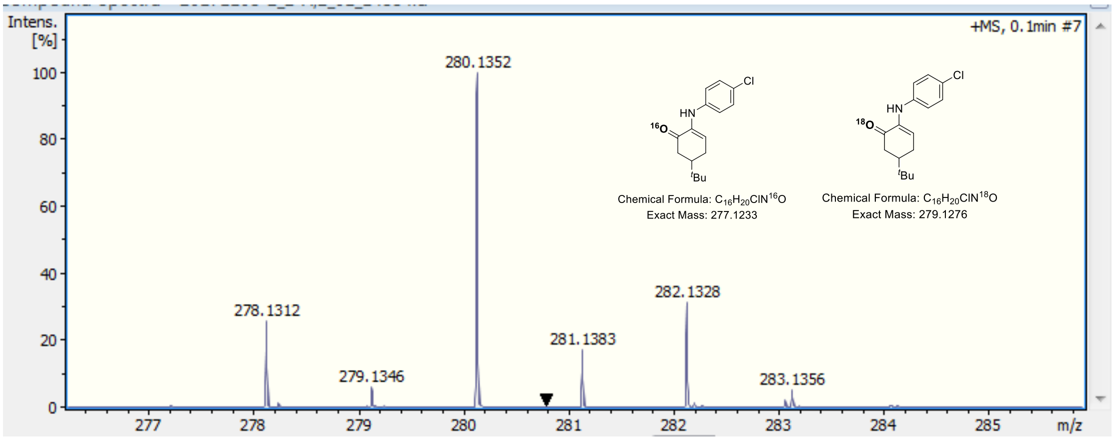

Supplementary Figure 2. The HRMS spectra of **^{18}O-3a** .

General Procedure A for *α*-Amino-Enone Formation Reaction.

In a nitrogen-filled glovebox, a 25 mL Schlenk tube equipped with a stir bar was charged with primary amine (0.2 mmol), 2-amino-5-methylbenzoic acid (0.0046g, 0.03 mmol, 15 mol%), cyclic ketone (0.3 mmol), TEMPO (0.0472g, 0.3 mmol), molecular sieve (3Å MS). The tube was covered with a rubber septum and moved out of the glove box. Then toluene (1.0 mL) was added to the Schlenk tube through the rubber septum using syringes, and then the rubber septum was replaced with a Teflon screwcap under nitrogen flow. The reaction mixture was stirred at 120 °C for 24 h. Upon cooling to room temperature, the mixture was filtered through a pad of silica gel and washed with 10 mL of ethyl acetate. The filtrate was concentrated under reduced pressure and purified by flash chromatography on silica gel to provide the corresponding product.

General Procedure B for Dehydrogenative Aromatization Reaction.

In a nitrogen-filled glovebox, a 25 mL Schlenk tube equipped with a stir bar was charged with cyclic ketone (0.2 mmol), secondary amines (0.3 mmol), TEMPO (0.0472g, 0.3 mmol), molecular sieve (3Å MS). The tube was covered with a rubber septum and moved out of the glove box. Then toluene (1.0 mL) was added to the Schlenk tube through the rubber septum using syringes, and then the rubber septum was replaced with a Teflon screwcap under nitrogen flow. The reaction mixture was stirred at 120 °C for 24 h. Upon cooling to room temperature, the mixture was filtered through a pad of silica gel and washed with 10 mL of ethyl acetate. The filtrate was concentrated under reduced pressure and purified by flash chromatography on silica gel to provide the corresponding product.

5-*tert*-Butyl-2-(4-chlorophenylamino)cyclohex-2-enone

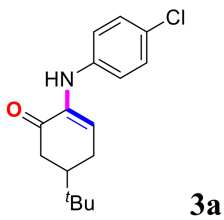

3a

3a (0.2 mmol scale) was synthesized following *the procedure A*. The reaction was carried out with 4-chloroaniline (0.0255 g, 0.2 mmol), 2-amino-5-methylbenzoic acid (0.0046 g, 0.03 mmol, 15 mol%), 4-*tert*-butylcyclohexanone (0.0463 g, 0.3 mmol), TEMPO (0.0472 g, 0.3 mmol), molecular sieve (400 mg, 3Å MS). The reaction mixture was stirred at 120 °C in toluene (1.0 mL) for 24 h. After concentration and purification by flash chromatography on silica gel (eluent = petroleum ether/ Et_2O = 100:5), the product **3a** was obtained in 74% yield (41.1 mg, white solid). **^{1}H NMR** (400 MHz, $CDCl_3$): δ 7.22-7.18 (m, 2H), 6.97-6.93 (m, 2H), 6.37 (dd, *J* = 6.9, 2.8 Hz, 1H), 6.31 (brs, 1H), 2.72-2.66 (m, 1H), 2.526-2.449 (m, 1H), 2.271-2.185 (m, 2H), 1.90-1.83 (m, 1H), 0.93 (s, 9H); **^{13}C NMR** (100 MHz, $CDCl_3$): δ 196.4, 140.6, 135.5, 129.1, 125.5, 119.5, 116.9, 45.3, 39.3, 32.2, 27.0, 26.1; **HRMS (ESI)** Calcd. for $C_{16}H_{21}ONCl$ ($[M+H]^+$): 278.1306, found: 278.1305.

5-*tert*-Butyl-2-(4-fluorophenylamino)cyclohex-2-enone

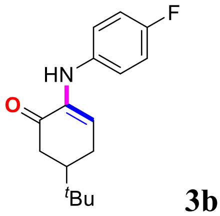

3b

3b (0.2 mmol scale) was synthesized following *the procedure A*. The reaction was carried out with 4-fluoroaniline (0.0222 g, 0.2 mmol), 2-amino-5-methylbenzoic acid (0.0046 g, 0.03 mmol, 15 mol%), 4-*tert*-butylcyclohexanone (0.0463 g, 0.3 mmol), TEMPO (0.0472 g, 0.3 mmol), molecular sieve (400 mg, 3Å MS). The reaction mixture was stirred at 120 °C in toluene (1.0 mL) for 24 h. After concentration and purification by flash chromatography on silica gel (eluent = petroleum ether/ Et_2O = 100:5), the product **3b** was obtained in 71% yield (37.2 mg, white solid). **^{1}H NMR** (400 MHz, $CDCl_3$): δ 6.98-6.94 (m, 4H), 6.25 (dd, *J* = 6.8, 2.6 Hz, 1H), 6.18 (brs, 1H), 2.71-2.67 (m, 1H), 2.48-2.41 (m, 1H), 2.27-2.16 (m, 2H), 1.89-1.81 (m, 1H), 0.92 (s, 9H); **^{13}C NMR** (100 MHz, $CDCl_3$): δ 196.4, 157.7 (d, $^1J_{C\text{-}F}$ = 240.3 Hz), 137.9 (d, $^4J_{C\text{-}F}$ = 2.4 Hz), 136.5, 120.9 (d, $^3J_{C\text{-}F}$ = 7.8 Hz), 115.8 (d, $^2J_{C\text{-}F}$ = 22.5 Hz), 115.3, 45.4, 39.4, 32.2, 27.0, 25.9; **^{19}F NMR** (377 MHz, $CDCl_3$): δ -121.94. **HRMS (ESI)** Calcd. for $C_{16}H_{21}ONF$ ($[M+H]^+$): 262.1602, found: 262.1601.

2-(4-Bromophenylamino)-5-*tert*-butylcyclohex-2-enone

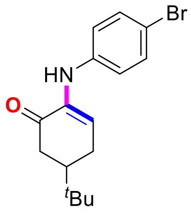

3c

3c (0.2 mmol scale) was synthesized following *the procedure A*. The reaction was carried out with 4-bromoaniline (0.0344 g, 0.2 mmol), 2-amino-5-methylbenzoic acid (0.0046 g, 0.03 mmol, 15 mol%), 4-*tert*-butylcyclohexanone (0.0463 g, 0.3 mmol), TEMPO (0.0472 g, 0.3 mmol), molecular sieve (400 mg, 3Å MS). The reaction mixture was stirred at 120 °C in toluene (1.0 mL) for 24 h. After concentration and purification by flash chromatography on silica gel (eluent = petroleum ether/ Et_2O =

100:5), the product **3c** was obtained in 65% yield (41.9 mg, white solid). **^{1}H NMR** (400 MHz, $CDCl_3$): δ 7.36-7.32 (m, 2H), 6.92-6.88 (m, 2H), 6.386 (dd, *J* = 6.9, 2.8 Hz, 1H), 6.32 (brs, 1H), 2.72-2.67 (m, 1H), 2.53-2.45 (m, 1H), 2.27-2.19 (m, 2H), 1.90-1.83 (m, 1H), 0.93 (s, 9H); **^{13}C NMR** (100 MHz, $CDCl_3$): δ 196.4, 141.1, 135.4, 132.1, 119.8, 117.2, 112.8, 45.3, 39.3, 32.2, 27.0, 26.1; **HRMS (ESI)** Calcd. for $C_{16}H_{21}ONBr$ ($[M+H]^+$): 322.0801, found: 322.0800.

5-*tert*-Butyl-2-(4-iodophenylamino)cyclohex-2-enone

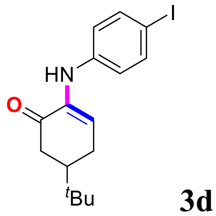

3d

3d (0.2 mmol scale) was synthesized following *the procedure A*. The reaction was carried out with 4-iodoaniline (0.0438 g, 0.2 mmol), 2-amino-5-methylbenzoic acid (0.0046 g, 0.03 mmol, 15 mol%), 4-*tert*-butylcyclohexanone (0.0463 g, 0.3 mmol), TEMPO (0.0472 g, 0.3 mmol), molecular sieve (400 mg, 3Å MS). The reaction mixture was stirred at 120 °C in toluene (1.0 mL) for 24 h. After concentration and purification by flash chromatography on silica gel (eluent = petroleum ether/ Et_2O = 100:5), the product **3d** was obtained in 58% yield (42.5 mg, white solid). **^{1}H NMR** (400 MHz, $CDCl_3$): δ 7.53-7.49 (m, 2H), 6.81-6.77 (m, 2H), 6.395 (dd, *J* = 6.9, 2.8 Hz, 1H), 6.33 (brs, 1H), 2.71-2.66 (m, 1H), 2.53-2.46 (m, 1H), 2.27-2.19 (m, 2H), 1.90-1.83 (m, 1H), 0.93 (s, 9H); **^{13}C NMR** (100 MHz, $CDCl_3$): δ 196.3, 141.8, 137.9, 135.2, 120.0, 117.5, 82.4, 45.2, 39.3, 32.2, 27.0, 26.1; **HRMS (ESI)** Calcd. for $C_{16}H_{21}ONI$ ($[M+H]^+$): 370.0662, found: 370.0661.

5-*tert*-Butyl-2-(4-(trifluoromethyl)phenylamino)cyclohex-2-enone

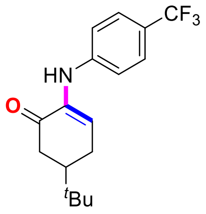

3e

3e (0.2 mmol scale) was synthesized following *the procedure A*. The reaction was carried out with 4-aminobenzotrifluoride (0.0322 g, 0.2 mmol), 2-amino-5-methylbenzoic acid (0.0046 g, 0.03 mmol, 15 mol%), 4-*tert*-butylcyclohexanone (0.0463 g, 0.3 mmol), TEMPO (0.0472 g, 0.3 mmol), molecular sieve (400 mg, 3Å MS). The reaction mixture was stirred at 120 °C in toluene (1.0 mL) for 24 h. After concentration and purification by flash chromatography on silica gel (eluent = petroleum ether/ Et_2O = 100:5), the product **3e** was obtained in 53% yield (33.0 mg, pale yellow solid). **^{1}H NMR** (400 MHz, $CDCl_3$): δ 7.47 (d, J = 8.6 Hz, 2H), 7.05 (d, J = 8.6 Hz, 2H), 6.58-6.55 (m, 2H), 2.74-2.69 (m, 1H), 2.59-2.51 (m, 1H), 2.31-2.21 (m, 2H), 1.93-1.85 (m, 1H), 0.94 (s, 9H); **^{13}C NMR** (100 MHz, $CDCl_3$): δ 196.3, 145.2, 134.7, 126.5 (q, $^3J_{C\text{-}F}$ = 3.8 Hz), 124.5 (q, $^1J_{C\text{-}F}$ = 270.8 Hz), 121.9 (q, $^2J_{C\text{-}F}$ = 32.8 Hz), 119.8, 116.5, 45.1, 39.2, 32.2, 27.0, 26.2; **^{19}F NMR** (377 MHz, $CDCl_3$): δ -61.55. **HRMS (ESI)** Calcd. for $C_{17}H_{21}ONF_3$ ([M+H]$^+$): 312.1570, found: 312.1569.

Methyl 4-(4-*tert*-butyl-6-oxocyclohex-1-enylamino)benzoate

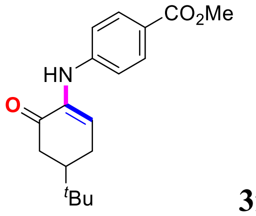

3f

3f (0.2 mmol scale) was synthesized following *the procedure A*. The reaction was carried out with methyl 4-aminobenzoate (0.0302 g, 0.2 mmol), 2-amino-5-methylbenzoic acid (0.0046 g, 0.03 mmol, 15 mol%), 4-*tert*-butylcyclohexanone (0.0463 g, 0.3 mmol), TEMPO (0.0472 g, 0.3 mmol), molecular sieve (400 mg, 3Å MS). The reaction mixture was stirred at 120 °C in toluene (1.0 mL) for 24 h. After concentration and purification by flash chromatography on silica gel (eluent = petroleum ether/ Et_2O = 100:10), the product **3f** was obtained in 61% yield (36.6 mg, white solid). **^{1}H NMR** (400 MHz, $CDCl_3$): δ 7.92 (d, J = 8.7 Hz, 2H), 7.00 (d, J = 8.7 Hz, 2H), 6.67 (brs, 1H), 6.61 (dd, J = 6.8, 2.6 Hz, 1H), 3.87 (s, 3H), 2.73-2.68 (m, 1H), 2.60-2.53 (m, 1H), 2.32-2.21 (m, 2H),

1.93-1.85 (m, 1H), 0.94 (s, 9H); **^{13}C NMR** (100 MHz, $CDCl_3$): δ 196.2, 166.8, 146.4, 134.5, 131.2, 121.4, 120.6, 115.7, 51.7, 45.0, 39.2, 32.2, 27.0, 26.2; **HRMS (ESI)** Calcd. for $C_{18}H_{24}O_3N$ ($[M+H]^+$): 302.1751, found: 302.1751.

5-*tert*-Butyl-2-(4-nitrophenylamino)cyclohex-2-enone

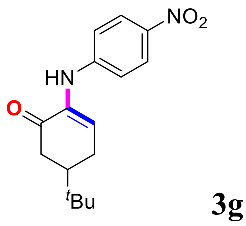

3g

3g (0.2 mmol scale) was synthesized following *the procedure A*. The reaction was carried out with 4-nitroaniline (0.0276 g, 0.2 mmol), 2-amino-5-methylbenzoic acid (0.0046 g, 0.03 mmol, 15 mol%), 4-*tert*-butylcyclohexanone (0.0463 g, 0.3 mmol), TEMPO (0.0472 g, 0.3 mmol), molecular sieve (400 mg, 3Å MS). The reaction mixture was stirred at 120 °C in toluene (1.0 mL) for 24 h. After concentration and purification by flash chromatography on silica gel (eluent = petroleum ether/ Et_2O = 100:10), the product **3g** was obtained in 42% yield (24.1 mg, yellow solid). **^{1}H NMR** (400 MHz, $CDCl_3$): δ 8.15-8.11 (m, 2H), 7.03-6.99 (m, 2H), 6.89 (brs, 1H), 6.72 (dd, J = 6.8, 2.8 Hz, 1H), 2.76-2.71 (m, 1H), 2.66-2.58 (m, 1H), 2.37-2.23 (m, 2H), 1.96-1.88 (m, 1H), 0.96 (s, 9H); **^{13}C NMR** (100 MHz, $CDCl_3$): δ 195.9, 148.2, 140.0, 134.0, 125.9, 123.6, 115.1, 44.9, 39.1, 32.2, 27.0, 26.4; **HRMS (ESI)** Calcd. for $C_{16}H_{21}O_3N_2$ ($[M+H]^+$): 289.1547, found: 289.1547.

5-*tert*-Butyl-2-(4-methoxyphenylamino)cyclohex-2-enone

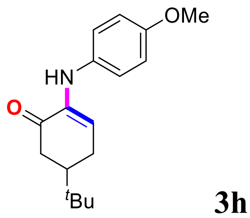

3h

3h (0.2 mmol scale) was synthesized following *the procedure A*. The reaction was carried out with *p*-anisidine (0.0246 g, 0.2 mmol), 2-amino-5-methylbenzoic acid (0.0046 g, 0.03 mmol, 15 mol%), 4-*tert*-butylcyclohexanone (0.0463 g, 0.3 mmol),

TEMPO (0.0472 g, 0.3 mmol), molecular sieve (400 mg, 3Å MS). The reaction mixture was stirred at 120 °C in toluene (1.0 mL) for 24 h. After concentration and purification by flash chromatography on silica gel (eluent = petroleum ether/ Et_2O = 100:10), the product **3h** was obtained in 82% yield (44.7 mg, pale yellow oil). **^{1}H NMR** (400 MHz, $CDCl_3$): δ 6.98 (d, J = 8.8 Hz, 2H), 6.83 (d, J = 8.8 Hz, 2H), 6.14 (dd, J = 6.8, 2.8 Hz, 1H), 6.06 (brs, 1H), 3.77 (s, 3H), 2.69-2.65 (m, 1H), 2.45-2.37 (m, 1H), 2.26-2.13 (m, 2H), 1.88-1.80 (m, 1H), 0.91 (s, 9H); **^{13}C NMR** (100 MHz, $CDCl_3$): δ 196.5, 154.8, 137.2, 135.0, 121.9, 114.4, 113.9, 55.4, 45.5, 39.4, 32.1, 27.0, 25.9; **HRMS (ESI)** Calcd. for $C_{17}H_{24}O_2N$ ($[M+H]^+$): 274.1802, found: 274.1801.

5-*tert*-Butyl-2-(4-hydroxyphenylamino)cyclohex-2-enone

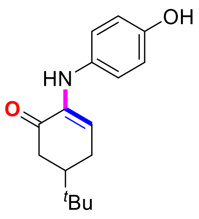

3i

3i (0.2 mmol scale) was synthesized following *the procedure A*. The reaction was carried out with 4-aminophenol (0.0218 g, 0.2 mmol), 2-amino-5-methylbenzoic acid (0.0046 g, 0.03 mmol, 15 mol%), 4-*tert*-butylcyclohexanone (0.0463 g, 0.3 mmol), TEMPO (0.0472 g, 0.3 mmol), molecular sieve (400 mg, 3Å MS). The reaction mixture was stirred at 120 °C in toluene (1.0 mL) for 24 h. After concentration and purification by flash chromatography on silica gel (eluent = petroleum ether/ Et_2O = 100:10), the product **3i** was obtained in 60% yield (31.1 mg, pale yellow solid). **^{1}H NMR** (400 MHz, $CDCl_3$): δ 6.94-6.90 (m, 2H), 6.81-6.77 (m, 2H); 6.14 (dd, J = 6.8, 2.9 Hz, 1H), 5.99 (brs, 1H), 5.77 (brs, 1H), 2.71-2.65 (m, 1H), 2.45-2.37 (m, 1H), 2.28-2.13 (m, 2H), 1.88-1.80 (m, 1H), 0.91 (s, 9H); **^{13}C NMR** (100 MHz, $CDCl_3$): δ 197.2, 151.0, 137.4, 134.8, 122.4, 116.0, 114.8, 45.6, 39.4, 32.2, 27.0, 25.9; **HRMS (ESI)** Calcd. for $C_{16}H_{22}O_2N$ ($[M+H]^+$): 260.1645, found: 260.1644.

5-(*tert*-Butyl)-2-(*m*-tolylamino)cyclohex-2-en-1-one

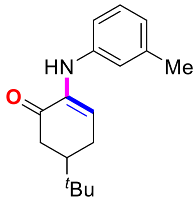

3j

3j (0.2 mmol scale) was synthesized following *the procedure A*. The reaction was carried out with 3-toluidine (0.0214 g, 0.2 mmol), 2-amino-5-methylbenzoic acid (0.0046 g, 0.03 mmol, 15 mol%), 4-*tert*-butylcyclohexanone (0.0463 g, 0.3 mmol), TEMPO (0.0472 g, 0.3 mmol), molecular sieve (400 mg, 3Å MS). The reaction mixture was stirred at 120 °C in toluene (1.0 mL) for 24 h. After concentration and purification by flash chromatography on silica gel (eluent = petroleum ether/ Et_2O = 100:5), the product **3j** was obtained in 80% yield (41.1 mg, pale yellow oil). **^{1}H NMR** (400 MHz, $CDCl_3$): δ 7.14 (t, J = 8.0 Hz, 1H), 6.85-6.83 (m, 2H), 6.73 (d, J = 7.4 Hz, 1H), 6.41 (dd, J = 6.6, 2.4 Hz, 1H), 6.28 (brs, 1H), 2.68 (d, J = 16.1 Hz, 1H), 2.50-2.45 (m, 1H), 2.30-2.19 (m, 5H), 1.89-1.83 (m, 1H), 0.93 (s, 9H); **^{13}C NMR** (100 MHz, $CDCl_3$): δ 196.5, 141.9, 139.0, 135.8, 129.0, 121.8, 119.2, 116.3, 115.5, 45.3, 39.4, 32.2, 27.0, 26.1, 21.5; **HRMS (ESI)** Calcd. for $C_{17}H_{24}ON$ ($[M+H]^+$): 258.1852, found: 258.1852.

5-(*tert*-Butyl)-2-(*o*-tolylamino)cyclohex-2-en-1-one

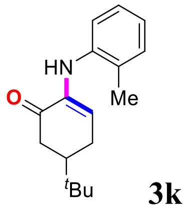

3k

3k (0.2 mmol scale) was synthesized following *the procedure A*. The reaction was carried out with 2-toluidine (0.0214 g, 0.2 mmol), 2-amino-5-methylbenzoic acid (0.0046 g, 0.03 mmol, 15 mol%), 4-*tert*-butylcyclohexanone (0.0463 g, 0.3 mmol), TEMPO (0.0472 g, 0.3 mmol), molecular sieve (400 mg, 3Å MS). The reaction mixture was stirred at 120 °C in toluene (1.0 mL) for 24 h. After concentration and purification by flash chromatography on silica gel (eluent = petroleum ether/ Et_2O =

100:5), the product **3k** was obtained in 77% yield (39.6 mg, 77% yield; pale yellow oil). **^{1}H NMR** (400 MHz, $CDCl_3$): δ 7.18-7.12 (m, 3H), 6.92-6.88 (m, 1H), 6.13 (dd, J = 6.8, 2.8 Hz, 1H), 6.06 (brs, 1H), 2.73-2.68 (m, 1H), 2.48-2.41 (m, 1H), 2.29-2.17 (m, 5H), 1.91-1.83 (m, 1H), 0.93 (s, 9H); **^{13}C NMR** (100 MHz, $CDCl_3$): δ 196.6, 140.1, 136.4, 130.9, 129.1, 126.5, 121.9, 119.3, 116.0, 45.5, 39.4, 32.2, 27.1, 26.0, 17.8; **HRMS (ESI)** Calcd. for $C_{17}H_{24}ON$ ($[M+H]^+$): 258.1852, found: 258.1852.

2-((1*H*-Indol-5-yl)amino)-5-(*tert*-butyl)cyclohex-2-en-1-one

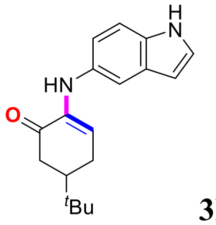

3l

3l (0.2 mmol scale) was synthesized following *the procedure A*. The reaction was carried out with 5-aminoindole (0.0264 g, 0.2 mmol), 2-amino-5-methylbenzoic acid (0.0046 g, 0.03 mmol, 15 mol%), 4-*tert*-butylcyclohexanone (0.0463 g, 0.3 mmol), TEMPO (0.0472 g, 0.3 mmol), molecular sieve (400 mg, 3Å MS). The reaction mixture was stirred at 120 °C in toluene (1.0 mL) for 24 h. After concentration and purification by flash chromatography on silica gel (eluent = petroleum ether/ ethyl acetate = 100:10), the product **3l** was obtained in 72% yield (40.9 mg, pale yellow oil). **^{1}H NMR** (400 MHz, $CDCl_3$): δ 8.18 (brs, 1H), 7.33 (s, 1H), 7.28 (d, J = 8.6 Hz, 1H), 7.15 (s, 1H), 6.91 (d, J = 7.4 Hz, 1H), 6.45 (s, 1H), 6.19 (dd, J = 6.6, 2.5 Hz, 1H), 6.13 (brs, 1H), 2.68 (d, J = 15.9 Hz, 1H), 2.41-2.37 (m, 1H), 2.28-2.12 (m, 2H), 1.88-1.82 (m, 1H), 0.91 (s, 9H); **^{13}C NMR** (100 MHz, $CDCl_3$): δ 196.9, 138.1, 134.4, 132.2, 128.3, 124.9, 118.0, 113.8, 112.5, 111.5, 102.2, 45.6, 39.5, 32.2, 27.0, 25.9; **HRMS (ESI)** Calcd. for $C_{18}H_{23}ON_2$ ($[M+H]^+$): 283.1805, found: 283.1804.

2-(Benzo[*d*]thiazol-2-ylamino)-5-(*tert*-butyl)cyclohex-2-en-1-one

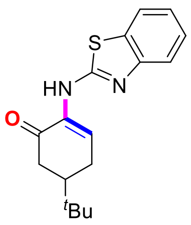

3m

3m (0.2 mmol scale) was synthesized following *the procedure A* with slight modifications. The reaction was carried out with 2-benzothiazolamine (0.0300 g, 0.2 mmol), $AlCl_3$ (0.0080 g, 0.06 mmol, 30 mol%), 4-*tert*-butylcyclohexanone (0.0617 g, 0.4 mmol), TEMPO (0.0472 g, 0.3 mmol). The reaction mixture was stirred at 120 °C in DCE (2.0 mL) for 24 h. After concentration and purification by flash chromatography on silica gel (eluent = petroleum ether/ ethyl acetate = 100:10), the product **3m** was obtained in 68% yield (41.0 mg, white solid). **^{1}H NMR** (400 MHz, $CDCl_3$): δ 7.93 (d, *J* = 4.7 Hz, 1H), 7.78 (brs, 1H), 7.65 (t, *J* = 8.4 Hz, 2H), 7.36-7.32 (m, 1H), 7.19-7.15 (m, 1H), 2.75-2.64 (m, 2H), 2.38-2.23 (m, 2H), 1.94-1.88 (m, 1H), 0.95 (s, 9H); **^{13}C NMR** (100 MHz, $CDCl_3$): δ 195.1, 161.0, 152.2, 133.2, 130.5, 126.4, 126.0, 122.8, 120.7, 120.1, 45.1, 38.8, 32.3, 27.0, 26.3; **HRMS (ESI)** Calcd. for $C_{17}H_{21}ON_2S$ ($[M+H]^+$): 301.1369, found: 301.1368.

5-*tert*-Butyl-2-(pyridin-3-ylamino)cyclohex-2-enone

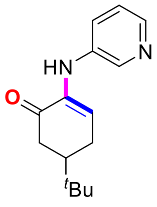

3n

3n (0.2 mmol scale) was synthesized following *the procedure A*. The reaction was carried out with 3-aminopyridine (0.0188 g, 0.2 mmol), 2-amino-5-methylbenzoic acid (0.0046 g, 0.03 mmol, 15 mol%), 4-*tert*-butylcyclohexanone (0.0463 g, 0.3 mmol), TEMPO (0.0472 g, 0.3 mmol), molecular sieve (400 mg, 3Å MS). The reaction mixture was stirred at 120 °C in toluene (1.0 mL) for 24 h. After concentration and purification by flash chromatography on silica gel (eluent = petroleum ether/ ethyl acetate = 100:10), the product **3n** was obtained in 71% yield

(34.5 mg, white solid). **^{1}H NMR** (400 MHz, $CDCl_3$): δ 8.38 (d, *J* = 2.7 Hz, 1H), 8.16-8.14 (m, 1H), 7.36-7.33 (m, 1H), 7.17 (dd, *J* = 8.3, 4.7 Hz, 1H), 6.43 (dd, *J* = 6.8, 2.8 Hz, 1H), 6.38 (brs, 1H), 2.74-2.69 (m, 1H), 2.55-2.47 (m, 1H), 2.29-2.22 (m, 2H), 1.92-1.84 (m, 1H), 0.94 (s, 9H); **^{13}C NMR** (100 MHz, $CDCl_3$): δ 196.2, 142.0, 140.8, 138.5, 135.2, 124.3, 123.5, 117.6, 45.2, 39.2, 32.2, 27.0, 26.0; **HRMS (ESI)** Calcd. for $C_{15}H_{21}ON_2$ ($[M+H]^+$): 245.1648, found: 245.1648.

2-(Benzylamino)-5-(*tert*-butyl)cyclohex-2-en-1-one

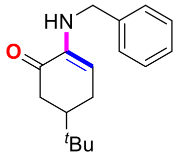

3o

3o (0.2 mmol scale) was synthesized following *the procedure A*. The reaction was carried out with benzylamine (0.0214 g, 0.2 mmol), 2-amino-5-methylbenzoic acid (0.0046 g, 0.03 mmol, 15 mol%), 4-*tert*-butylcyclohexanone (0.0463 g, 0.3 mmol), TEMPO (0.0472 g, 0.3 mmol), molecular sieve (400 mg, 3Å MS). The reaction mixture was stirred at 120 °C in toluene (1.0 mL) for 24 h. After concentration and purification by flash chromatography on silica gel (eluent = petroleum ether/ ethyl acetate = 100:5), the product **3o** was obtained in 68% yield (34.8 mg, pale yellow oil). **^{1}H NMR** (400 MHz, $CDCl_3$): δ 7.34-7.23 (m, 5H), 5.42 (dd, *J* = 6.8, 2.8 Hz, 1H), 4.56 (brs, 1H), 4.09 (d, *J* = 2.6 Hz, 2H), 2.64-2.59 (m, 1H), 2.39-2.32 (m, 1H), 2.22-2.08 (m, 2H), 1.83-1.74 (m, 1H), 0.89 (s, 9H); **^{13}C NMR** (100 MHz, $CDCl_3$): δ 196.8, 140.0, 139.1, 128.5, 127.3, 127.1, 111.6, 47.6, 45.9, 39.6, 32.2, 27.1, 25.9; **HRMS (ESI)** Calcd. for $C_{17}H_{24}ON$ ($[M+H]^+$): 258.1852, found: 258.1852.

5-(*tert*-Butyl)-2-((2-(thiophen-2-yl)ethyl)amino)cyclohex-2-en-1-one

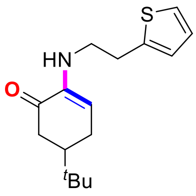

3p

3p (0.2 mmol scale) was synthesized following *the procedure A* with slight modifications. The reaction was carried out with thiophene-2-ethylamine (0.0254 g,

0.2 mmol), 2-amino-5-methylbenzoic acid (0.0046 g, 0.03 mmol, 15 mol%), 4-*tert*-butylcyclohexanone (0.0772 g, 0.5 mmol), TEMPO (0.0472 g, 0.3 mmol), molecular sieve (400 mg, 3Å MS). The reaction mixture was stirred at 120 ^{o}C in toluene (1.0 mL) for 24 h. After concentration and purification by neutral aluminium oxide column (eluent = petroleum ether/ ethyl acetate = 100:5), the product **3p** was obtained in 42% yield (23.5 mg, pale yellow oil). **^{1}H NMR** (400 MHz, $CDCl_3$): δ 7.14 (d, J = 4.4 Hz, 1H), 6.93 (dd, J = 5.0, 3.5 Hz, 1H), 6.83 (d, J = 3.0 Hz, 1H), 5.48 (dd, J = 6.8, 2.6 Hz, 1H), 4.25 (brs, 1H), 3.19-3.15 (m, 2H), 3.08-3.04 (m, 2H), 2.62-2.57 (m, 1H), 2.44-2.37 (m, 1H), 2.20-2.13 (m, 2H), 1.82-1.74 (m, 1H), 0.90 (s, 9H); **^{13}C NMR** (100 MHz, $CDCl_3$): δ 196.7, 141.8, 139.7, 126.9, 125.1, 123.7, 111.2, 45.9, 44.7, 39.6, 32.2, 29.2, 27.1, 26.0; **HRMS (ESI)** Calcd. for $C_{16}H_{24}ONS$ ($[M+H]^+$): 278.1573, found: 278.1573.

5-(*tert*-Butyl)-2-(cyclohexylamino)cyclohex-2-enone

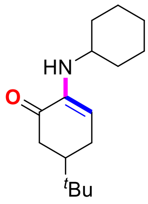

3q

3q (0.2 mmol scale) was synthesized following *the procedure A*. The reaction was carried out with cyclohexylamine (0.0198 g, 0.2 mmol), 2-amino-5-methylbenzoic acid (0.0046 g, 0.03 mmol, 15 mol%), 4-*tert*-butylcyclohexanone (0.0463 g, 0.3 mmol), TEMPO (0.0472 g, 0.3 mmol), molecular sieve (400 mg, 3Å MS). The reaction mixture was stirred at 120 ^{o}C in toluene (1.0 mL) for 24 h. After concentration and purification by flash chromatography on silica gel (eluent = petroleum ether/ ethyl acetate = 100:10), the product **3q** was obtained in 54% yield (26.9 mg, pale yellow oil). **^{1}H NMR** (400 MHz, $CDCl_3$): δ 5.44 (dd, J = 6.8, 2.8 Hz, 1H), 4.04 (brs, 1H), 2.94-2.88 (m, 1H), 2.62-2.57 (m, 1H), 2.43-2.35 (m, 1H), 2.20-2.11 (m, 2H), 1.95-1.92 (m, 2H), 1.76-1.71 (m, 2H), 1.62-1.58 (m, 1H), 1.35-1.05 (m, 6H), 0.90 (s, 9H); **^{13}C NMR** (100 MHz, $CDCl_3$): δ 197.2, 138.8, 110.7, 51.0, 45.9, 39.6, 32.9, 32.6, 32.2, 27.1, 26.00, 25.95, 24.9; **HRMS (ESI)** Calcd. for

$C_{16}H_{28}ON$ ([M+H]$^+$): 250.2165, found: 250.2165.

***tert*-Butyl (4-(*tert*-butyl)-6-oxocyclohex-1-en-1-yl)-*L*-tyrosinate**

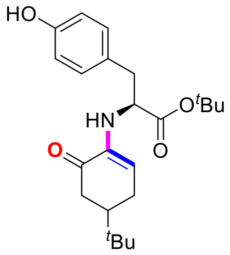

3r

3r (0.2 mmol scale) was synthesized following *the procedure A* with slight modifications. The reaction was carried out with D-tyrosinetert-butylester (0.0475 g, 0.2 mmol), 2-amino-5-methylbenzoic acid (0.0046 g, 0.03 mmol, 15 mol%), 4-*tert*-butylcyclohexanone (0.0772 g, 0.5 mmol), TEMPO (0.0472 g, 0.3 mmol), molecular sieve (400 mg, 3Å MS). The reaction mixture was stirred at 120 ^{o}C in toluene (1.0 mL) for 24 h. After concentration and purification by flash chromatography on silica gel (eluent = petroleum ether/ ethyl acetate = 100:15), the product **3r** was obtained in 57% yield (44.0 mg, pale yellow oil). **^{1}H NMR** (400 MHz, $CDCl_3$): δ 7.03 (d, J = 8.4 Hz, 2H), 6.73 (d, J = 7.5 Hz, 2H), 5.50-5.45 (m, 1H), 4.63-4.59 (m, 1H), 3.83-3.80 (m, 1H), 2.96-2.93 (m, 2H), 2.60-2.56 (m, 1H), 2.38-2.31 (m, 1H), 2.18-2.06 (m, 2H), 1.78-1.69 (m, 1H), 1.42 (s, 1H), 1.38 (s, 9H), 0.87 (s, 9H); **^{13}C NMR** (100 MHz, $CDCl_3$): δ 196.90, 196.87, 172.50, 172.33, 155.17, 138.88, 138.83, 130.31, 127.87, 127.80, 115.32, 113.53, 113.20, 81.62, 58.35, 58.07, 45.75, 45.52, 39.36, 39.29, 37.57, 37.48, 32.12, 32.08, 27.90, 26.96, 26.83, 25.94, 25.84; **HRMS (ESI)** Calcd. for $C_{23}H_{34}O_4N$ ([M+H]$^+$): 388.2482, found: 388.2482.

4-((4-Methoxyphenyl)amino)-1,6-dihydro-[1,1'-biphenyl]-3(2*H*)-one

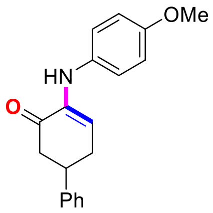

3s

3s (0.2 mmol scale) was synthesized following *the procedure A*. The reaction was

carried out with *p*-anisidine (0.0246 g, 0.2 mmol), 2-amino-5-methylbenzoic acid (0.0046 g, 0.03 mmol, 15 mol%), 4-phenylcyclohexanone (0.0523 g, 0.3 mmol), TEMPO (0.0472 g, 0.3 mmol), molecular sieve (400 mg, 3Å MS). The reaction mixture was stirred at 120 °C in toluene (1.0 mL) for 24 h. After concentration and purification by flash chromatography on silica gel (eluent = petroleum ether/ ethyl acetate = 100:5), the product **3s** was obtained in 63% yield (37.0 mg, pale yellow solid); **^{1}H NMR** (400 MHz, $CDCl_3$): δ 7.36-7.32 (m, 2H), 7.27-7.24 (m, 3H), 7.02 (d, *J* = 8.9 Hz, 2H), 6.86 (d, *J* = 8.9 Hz, 2H), 6.16-6.14 (m, 2H), 3.78(s, 3H), 3.39-3.31 (m, 1H), 2.88-2.72 (m, 2H), 2.68-2.54 (m, 2H); **^{13}C NMR** (100 MHz, $CDCl_3$): δ 194.8, 155.0, 143.3, 137.7, 134.6, 128.6, 126.8, 126.6, 122.3, 114.5, 112.4, 55.4, 44.1, 41.2, 32.5; **HRMS (ESI)** Calcd. for $C_{19}H_{20}O_2N$ ([M+H]$^+$): 294.1489, found: 294.1487.

Ethyl 4-((4-methoxyphenyl)amino)-5-oxocyclohex-3-ene-1-carboxylate

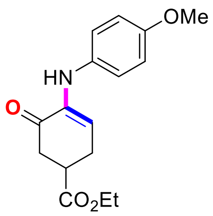

3t

3t (0.2 mmol scale) was synthesized following *the procedure A*. The reaction was carried out with *p*-anisidine (0.0246 g, 0.2 mmol), 2-amino-5-methylbenzoic acid (0.0046 g, 0.03 mmol, 15 mol%), ethyl 4-oxocyclohexanecarboxylate (0.0511 g, 0.3 mmol), TEMPO (0.0472 g, 0.3 mmol), molecular sieve (400 mg, 3Å MS). The reaction mixture was stirred at 120 °C in toluene (1.0 mL) for 24 h. After concentration and purification by flash chromatography on silica gel (eluent = petroleum ether/ ethyl acetate = 100:10), the product **3t** was obtained in 68% yield (37.2 mg, pale yellow oil). **^{1}H NMR** (400 MHz, $CDCl_3$): δ 6.99 (d, *J* = 8.9 Hz, 2H), 6.85 (d, *J* = 8.9 Hz, 2H), 6.09 (brs, 1H), 6.03-6.00 (m, 1H), 4.17 (qd, *J* = 7.2, 1.3 Hz, 2H), 3.78 (s, 3H), 3.09-3.01 (m, 1H), 2.86-2.62 (m, 4H), 1.27 (t, *J* = 7.2 Hz, 3H); **^{13}C NMR** (100 MHz, $CDCl_3$): δ 193.2, 173.1, 155.2, 137.7, 134.4, 122.5, 114.6, 110.3, 61.0, 55.5, 40.1, 39.3, 26.9, 14.1; **HRMS (ESI)** Calcd. for $C_{16}H_{20}O_4N$ ([M+H]$^+$): 290.1387, found: 290.1387.

Ethyl 2-(4-((4-methoxyphenyl)amino)-5-oxocyclohex-3-en-1-yl)acetate

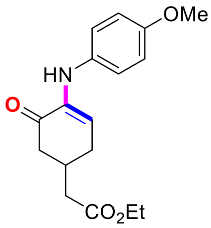

3u

3u (0.2 mmol scale) was synthesized following *the procedure A*. The reaction was carried out with *p*-anisidine (0.0246 g, 0.2 mmol), 2-amino-5-methylbenzoic acid (0.0046 g, 0.03 mmol, 15 mol%), ethyl 2-(4-oxocyclohexyl)acetate (0.0553 g, 0.3 mmol), TEMPO (0.0472 g, 0.3 mmol), molecular sieve (400 mg, 3Å MS). The reaction mixture was stirred at 120 °C in toluene (1.0 mL) for 24 h. After concentration and purification by flash chromatography on silica gel (eluent = petroleum ether/ ethyl acetate = 100:10), the product **3u** was obtained in 67% yield (38.6 mg, pale yellow oil). **^{1}H NMR** (400 MHz, CDCl3): δ 6.99 (d, *J* = 8.8 Hz, 2H), 6.84 (d, *J* = 8.8 Hz, 2H), 6.08-6.05 (m, 2H), 4.15 (q, *J* = 7.1 Hz, 2H), 3.78 (s, 3H), 2.71 (dd, *J* = 16.3, 2.6 Hz, 1H), 2.63-2.48 (m, 2H), 2.40-2.38 (m, 2H), 2.35-2.28 (m, 1H), 2.23-2.15 (m, 1H); 1.26 (t, *J* = 7.1 Hz, 3H); **^{13}C NMR** (100 MHz, CDCl3): δ 194.4, 171.6, 155.0, 137.6, 134.6, 122.3, 114.5, 111.9, 60.5, 55.4, 43.3, 40.0, 32.2, 30.3, 14.2; HRMS (ESI) Calcd. for C17H22O4N ([M+H]+): 304.1543, found: 304.1542.

tert-Butyl (4-((4-methoxyphenyl)amino)-5-oxocyclohex-3-en-1-yl)carbamate

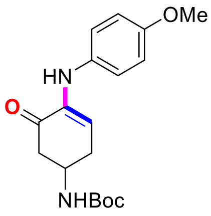

3v

3v (0.2 mmol scale) was synthesized following *the procedure A*. The reaction was carried out with *p*-anisidine (0.0246 g, 0.2 mmol), 2-amino-5-methylbenzoic acid (0.0046 g, 0.03 mmol, 15 mol%), 4-*N*-Boc-aminocyclohexanone (0.0640 g, 0.3 mmol), TEMPO (0.0472 g, 0.3 mmol), molecular sieve (400 mg, 3Å MS). The reaction mixture was stirred at 120 °C in toluene (1.0 mL) for 24 h. After

concentration and purification by flash chromatography on silica gel (eluent = petroleum ether/ ethyl acetate = 100:10), the product **3v** was obtained in 63% yield (42.1 mg, pale yellow oil). **^{1}H NMR** (400 MHz, $CDCl_3$): δ 7.01-6.97 (m, 2H), 6.87-6.83 (m, 2H), 6.10 (brs, 1H), 5.97 (dd, *J* = 5.5, 4.2 Hz, 1H), 4.83 (d, *J* = 6.6 Hz, 1H), 4.17 (brs, 1H), 3.78 (s, 3H), 2.86 (dd, *J* = 16.5, 3.6 Hz, 1H), 2.74 (d, *J* = 17.5 Hz, 1H), 2.56 (dd, *J* = 16.3, 9.7 Hz, 1H), 2.38-2.30 (m, 1H), 1.44 (s, 9H); **^{13}C NMR** (100 MHz, $CDCl_3$): δ 193.2, 155.2, 154.9, 138.0, 134.2, 122.6, 114.5, 109.3, 79.7, 55.5, 46.7, 44.0, 31.1, 28.3; **HRMS (ESI)** Calcd. for $C_{18}H_{25}O_4N_2$ ($[M+H]^+$): 333.1809, found: 333.1808.

2-(4-Methoxyphenylamino)-5,5-dimethylcyclohex-2-enone

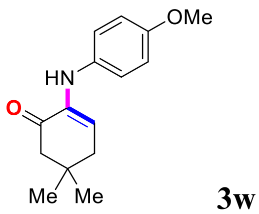

3w

3w (0.2 mmol scale) was synthesized following *the procedure A*. The reaction was carried out with *p*-anisidine (0.0246 g, 0.2 mmol), 2-amino-5-methylbenzoic acid (0.0046 g, 0.03 mmol, 15 mol%), 4,4-dimethylcyclohexanone (0.0379 g, 0.3 mmol), TEMPO (0.0472 g, 0.3 mmol), molecular sieve (400 mg, 3Å MS). The reaction mixture was stirred at 120 °C in toluene (1.0 mL) for 24 h. After concentration and purification by flash chromatography on silica gel (eluent = petroleum ether/ ethyl acetate = 100:5), the product **3w** was obtained in 71% yield (34.7 mg, pale yellow oil). **^{1}H NMR** (400 MHz, $CDCl_3$): δ 7.02-6.98 (m, 2H), 6.86-6.82 (m, 2H), 6.07 (brs, 1H), 5.99 (t, *J* = 4.9 Hz, 1H), 3.78 (s, 3H), 2.40 (s, 2H), 2.29 (d, *J* = 4.9 Hz, 2H), 1.08 (s, 6H); **^{13}C NMR** (100 MHz, $CDCl_3$): δ 195.6, 154.8, 136.8, 135.0, 121.9, 114.5, 111.3, 55.5, 51.1, 38.4, 34.1, 28.2; **HRMS (ESI)** Calcd. for $C_{15}H_{20}O_2N$ ($[M+H]^+$): 246.1489, found: 246.1488.

2-((4-Methoxyphenyl)amino)-4,4-dimethylcyclohex-2-en-1-one

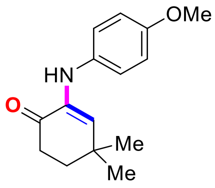

3x

3x (0.2 mmol scale) was synthesized following *the procedure A*. The reaction was carried out with *p*-anisidine (0.0246 g, 0.2 mmol), 2-amino-5-methylbenzoic acid (0.0046 g, 0.03 mmol, 15 mol%), 3,3-dimethylcyclohexanone (0.0379 g, 0.3 mmol), TEMPO (0.0472 g, 0.3 mmol), molecular sieve (400 mg, 3Å MS). The reaction mixture was stirred at 120 °C in toluene (1.0 mL) for 24 h. After concentration and purification by flash chromatography on silica gel (eluent = petroleum ether/ ethyl acetate = 100:5), the product **3x** was obtained in 74% yield (36.2 mg, pale yellow oil). **^{1}H NMR** (400 MHz, $CDCl_3$): δ 6.98 (d, *J* = 8.8 Hz, 2H), 6.85 (d, *J* = 8.8 Hz, 2H), 5.99 (brs, 1H), 5.87 (s, 1H), 3.78 (s, 3H), 2.60 (t, *J* = 6.7 Hz, 2H), 1.86 (t, *J* = 6.7 Hz, 2H), 1.16 (s, 6H); **^{13}C NMR** (100 MHz, $CDCl_3$): δ 195.3, 154.8, 135.2, 134.8, 124.3, 121.9, 114.5, 55.5, 36.0, 33.8, 32.2, 29.1; **HRMS (ESI)** Calcd. for $C_{15}H_{20}O_2N$ ($[M+H]^+$): 246.1489, found: 246.1488.

2-((4-Methoxyphenyl)amino)-5-pentylcyclohex-2-en-1-one

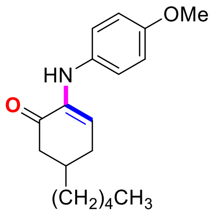

3y

3y (0.2 mmol scale) was synthesized following *the procedure A*. The reaction was carried out with *p*-anisidine (0.0246 g, 0.2 mmol), 2-amino-5-methylbenzoic acid (0.0046 g, 0.03 mmol, 15 mol%), 4-*n*-pentylcyclohexanone (0.0505 g, 0.3 mmol), TEMPO (0.0472 g, 0.3 mmol), molecular sieve (400 mg, 3Å MS). The reaction mixture was stirred at 120 °C in toluene (1.0 mL) for 24 h. After concentration and purification by flash chromatography on silica gel (eluent = petroleum ether/ ethyl acetate = 100:5), the product **3y** was obtained in 81% yield (46.6 mg, pale yellow oil).

^{1}H NMR (400 MHz, $CDCl_3$): δ 6.99 (d, J = 8.8 Hz, 2H), 6.84 (d, J = 8.8 Hz, 2H), 6.11-6.04 (m, 2H), 3.78 (s, 3H), 2.66 (d, J = 16.4 Hz, 1H), 2.48-2.40 (m, 1H), 2.24-2.07 (m, 3H), 1.36-1.29 (m, 8H), 0.90-0.87 (m, 3H); **^{13}C NMR** (100 MHz, $CDCl_3$): δ 195.9, 154.9, 137.6, 135.0, 122.1, 114.5, 113.3, 55.5, 44.0, 35.64, 35.56, 31.8, 31.0, 26.2, 22.6, 14.0; **HRMS (ESI)** Calcd. for $C_{18}H_{26}O_2N$ ($[M+H]^+$): 288.1958, found: 288.1957.

2-((4-Methoxyphenyl)amino)-4-methylcyclohex-2-en-1-one

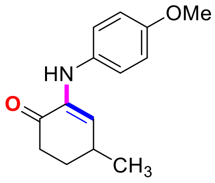

3z

3z (0.2 mmol scale) was synthesized following *the procedure A* with slight modifications. The reaction was carried out with *p*-anisidine (0.0246 g, 0.2 mmol), 2-amino-5-methylbenzoic acid (0.0046 g, 0.03 mmol, 15 mol%), 3-methylcyclohexanone (0.0337 g, 0.3 mmol), TEMPO (0.0472 g, 0.3 mmol), molecular sieve (400 mg, 3Å MS). The reaction mixture was stirred at 120 °C in toluene (1.0 mL) for 48 h. After concentration and purification by flash chromatography on silica gel (eluent = petroleum ether/ ethyl acetate = 100:5), the product **3z** was obtained in 43% yield (19.9 mg, Pale yellow oil). **^{1}H NMR** (400 MHz, $CDCl_3$): δ 7.02-6.98 (m, 2H), 6.87-6.83 (m, 2H), 6.06 (brs, 1H), 5.97 (dd, J = 3.2, 0.9 Hz, 1H), 3.78 (s, 3H), 2.67-2.60 (m, 2H), 2.50-2.42 (m, 1H), 2.10-2.03 (m, 1H), 1.68-1.58 (m, 1H), 1.12 (d, J = 7.0 Hz, 3H); **^{13}C NMR** (100 MHz, $CDCl_3$): δ 195.6, 154.9, 136.6, 134.8, 122.1, 120.1, 114.5, 55.5, 36.5, 31.1, 30.3, 21.9; **HRMS (ESI)** Calcd. for $C_{14}H_{18}O_2N$ ($[M+H]^+$): 232.1332, found: 232.1331.

2-(4-Methoxyphenylamino)cyclopent-2-enone

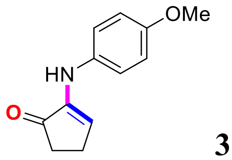

3aa

3aa (0.2 mmol scale) was synthesized following *the procedure A*. The reaction was

carried out with *p*-anisidine (0.0246 g, 0.2 mmol), 2-amino-5-methylbenzoic acid (0.0046 g, 0.03 mmol, 15 mol%), adipic ketone (0.0252 g, 0.3 mmol), TEMPO (0.0472 g, 0.3 mmol), molecular sieve (400 mg, 3Å MS). The reaction mixture was stirred at 120 °C in toluene (1.0 mL) for 24 h. After concentration and purification by flash chromatography on silica gel (eluent = petroleum ether/ ethyl acetate = 100:5), the product **3aa** was obtained in 66% yield (26.9 mg, white solid). **^{1}H NMR** (400 MHz, $CDCl_3$): δ 7.01-6.97 (m, 2H), 6.88-6.84 (m, 2H), 6.59 (t, *J* = 3.2 Hz, 1H), 6.02 (brs, 1H), 3.78 (s, 3H), 2.63-2.60 (m, 2H), 2.48-2.46 (m, 2H); **^{13}C NMR** (100 MHz, $CDCl_3$): δ 204.7, 154.2, 141.0, 135.2, 122.9, 118.6, 114.6, 55.6, 32.6, 23.7; **HRMS (ESI)** Calcd. for $C_{12}H_{14}O_2N$ ($[M+H]^+$): 204.1019, found: 204.1018.

2-((4-Methoxyphenyl)amino)cyclohex-2-en-1-one

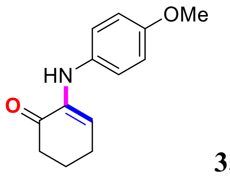

3ab

3ab (0.2 mmol scale) was synthesized following *the procedure A*. The reaction was carried out with *p*-anisidine (0.0246 g, 0.2 mmol), 2-amino-5-methylbenzoic acid (0.0046 g, 0.03 mmol, 15 mol%), cyclohexanone (0.0294 g, 0.3 mmol), TEMPO (0.0472 g, 0.3 mmol), molecular sieve (400 mg, 3Å MS). The reaction mixture was stirred at 120 °C in toluene (1.0 mL) for 24 h. After concentration and purification by flash chromatography on silica gel (eluent = petroleum ether/ ethyl acetate = 100:5), the product **3ab** was obtained in 70% yield (30.3 mg, pale yellow oil). **^{1}H NMR** (400 MHz, CDC13): δ 7.01-6.97 (m, 2H), 6.86-6.82 (m, 2H), 6.13 (t, *J* = 4.8 Hz, 1H), 6.09 (brs, 1H), 3.77 (s, 3H), 2.56-2.53 (m, 2H), 2.41-2.37 (m, 2H), 2.02-1.96 (m, 2H); **^{13}C NMR** (100 MHz, $CDCl_3$): δ 195.6, 154.8, 137.7, 134.8, 122.1, 114.5, 114.0, 55.5, 37.7, 24.4, 23.1; **HRMS (ESI)** Calcd. for $C_{13}H_{16}O_2N$ ($[M+H]^+$): 218.1176, found: 218.1175.

2-(phenylamino)cyclohex-2-en-1-one

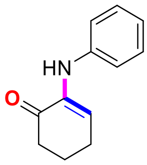

3ac

3ac (0.2 mmol scale) was synthesized following *the procedure A* with slight modifications. The reaction was carried out with aniline (0.0186 g, 0.2 mmol), 2-amino-5-methylbenzoic acid (0.0046 g, 0.03 mmol, 15 mol%), 4-*tert*-butylcyclohexanone (0.0463 g, 0.3 mmol), TEMPO (0.0472 g, 0.3 mmol), molecular sieve (400 mg, 3Å MS). The reaction mixture was stirred at 120 °C in toluene (1.0 mL) for 24 h. After concentration and purification by flash chromatography on silica gel (eluent = petroleum ether/ Et_2O = 100:5), the product **3ac** was obtained in 55% yield (20.6 mg, white solid) .[7] **^{1}H NMR** (400 MHz, $CDCl_3$): δ 7.28-7.24 (m, 2H), 7.04-7.02 (m, 2H), 6.92-6.88 (m, 1H), 6.41 (d, J = 4.8 Hz, 1H), 6.36 (bs, 1H), 2.55 (t, J = 6.7 Hz, 2H), 2.46-2.42 (m, 2H), 2.04-1.98 (m, 2H); **^{13}C NMR** (100 MHz, $CDCl_3$): δ 195.5, 141.9, 136.2, 129.2, 121.0, 118.6, 116.4, 37.7, 24.5, 22.9.

(5S,8S,9S,10R,13R,14S,17R)-3-((4-methoxyphenyl)amino)-10,13-dimethyl-17-((R)-6-methylheptan-2-yl)-1,5,6,7,8,9,10,11,12,13,14,15,16,17-tetradecahydro-2H-cyclopenta[a]phenanthren-2-one

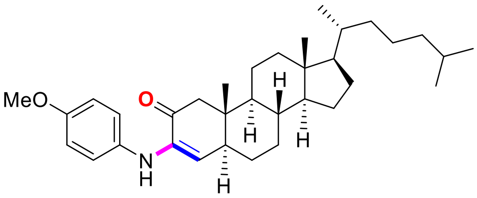

3ad

3ad (0.2 mmol scale) was synthesized following *the procedure A*. The reaction was carried out with *p*-anisidine (0.0246 g, 0.2 mmol), 2-amino-5-methylbenzoic acid (0.0046 g, 0.03 mmol, 15 mol%), 5α-cholestan-3-one (0.1160 g, 0.3 mmol), TEMPO (0.0472 g, 0.3 mmol), molecular sieve (400 mg, 3Å MS). The reaction mixture was stirred at 120 °C in toluene (1.0 mL) for 24 h. After concentration and purification by flash chromatography on silica gel (eluent = petroleum ether/ Et_2O = 100:10), the

product **3ad** was obtained in 72% yield (72.8 mg, pale yellow solid). **^{1}H NMR** (400 MHz, $CDCl_3$): δ 7.03-6.97 (m, 2H), 6.86-6.82 (m, 2H), 6.09 (s, 1H), 5.69 (d, *J* = 2.6 Hz, 1H), 3.78 (s, 3H), 2.69 (d, *J* = 16.2 Hz, 1H), 2.45-2.40 (m, 1H), 2.16 (d, *J* = 16.2 Hz, 1H), 2.02-1.99 (m, 1H), 1.86-1.73 (m, 2H), 1.62-0.85 (m, 34 H), 0.66 (s, 3H); **^{13}C NMR** (100 MHz, $CDCl_3$): δ 196.1, 154.7, 136.5, 135.0, 121.7, 116.9, 114.5, 56.2, 55.5, 53.0, 51.5, 45.8, 42.6, 41.2, 39.7, 39.5, 36.1, 35.7, 34.6, 31.8, 28.2, 28.0, 27.6, 24.1, 23.8, 22.8, 22.5, 21.0, 18.6, 12.8, 12.1; **HRMS (ESI)** Calcd. for $C_{34}H_{52}O_2N$ ($[M+H]^+$): 506.3993, found: 506.3992.

(5S,8R,9S,10R,13S,14S,17S)-17-acetyl-3-((4-methoxyphenyl)amino)-10,13-dimethyl-1,5,6,7,8,9,10,11,12,13,14,15,16,17-tetradecahydro-2H-cyclopenta[a]phenanthren-2-one

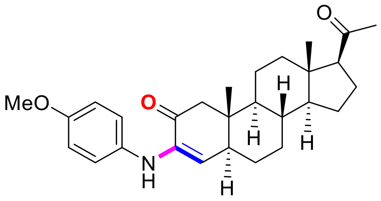

3ae

3ae (0.2 mmol scale) was synthesized following *the procedure A*. The reaction was carried out with aniline (0.0186 g, 0.2 mmol), 2-amino-5-methylbenzoic acid (0.0046 g, 0.03 mmol, 15 mol%), (5α)-pregnane-3,20-dione (0.0949 g, 0.3 mmol), TEMPO (0.0472 g, 0.3 mmol), molecular sieve (400 mg, 3Å MS). The reaction mixture was stirred at 120 °C in toluene (1.0 mL) for 24 h. After concentration and purification by flash chromatography on silica gel (eluent = petroleum ether/ Et_2O = 100:10), the product **3ae** was obtained in 65% yield (56.3 mg, pale yellow solid). **^{1}H NMR** (400 MHz, $CDCl_3$): δ 7.02-6.98 (m, 2H), 6.87-6.83 (m, 2H), 6.08 (brs, 1H), 5.68 (d, *J* = 2.7 Hz, 1H), 3.78 (s, 3H), 2.71 (d, *J* = 16.2 Hz, 1H), 2.56-2.52 (m, 1H), 2.45 (dt, *J* = 13.0, 3.0 Hz, 1H), 2.20-2.16 (m, 2H), 2.12 (s, 3H), 2.07-2.02 (m, 1H), 1.80-1.75 (m, 1H), 1.70-1.53 (m, 4H), 1.48-1.32 (m, 4H), 1.24-1.18 (m, 2H), 1.11-1.01 (m, 2H), 0.93 (s, 3H), 0.61 (s, 3H); **^{13}C NMR** (100 MHz, $CDCl_3$): δ 209.4, 195.8, 154.8, 136.6, 134.9, 121.8, 116.5, 114.5, 63.7, 56.4, 55.5, 52.9, 51.4, 45.8, 44.2, 41.1, 38.8, 34.6, 31.8, 31.5, 27.5, 24.3, 22.7, 21.1, 13.5, 12.8; **HRMS (ESI)** Calcd. for $C_{28}H_{38}O_3N$ ($[M+H]^+$):

436.2846, found: 436.2846.

4-Phenylmorpholine

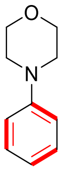

7a

7a (0.4 mmol scale) was synthesized following *the procedure B*. The reaction was carried out with cyclohexanone (0.0393 g, 0.4 mmol), morpholine (0.0523 g, 0.6 mmol), TEMPO (0.1572 g, 1.0 mmol), molecular sieve (800 mg, 3Å MS). The reaction mixture was stirred at 120 °C in toluene (1.0 mL) for 24 h. After concentration and purification by flash chromatography on silica gel (eluent = petroleum ether/ Et_2O = 100:5), the product **7a** was obtained in 62% yield (40.5 mg, pale white oil).This compound was known.[8]

4-Phenylthiomorpholine

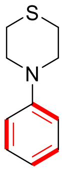

7b

7b (0.4 mmol scale) was synthesized following *the procedure B*. The reaction was carried out with cyclohexanone (0.0393 g, 0.4 mmol), thiomorpholine (0.0619 g, 0.6 mmol), TEMPO (0.1572 g, 1.0 mmol), molecular sieve (800 mg, 3Å MS). The reaction mixture was stirred at 120 °C in toluene (1.0 mL) for 24 h. After concentration and purification by flash chromatography on silica gel (eluent = petroleum ether/ Et_2O = 100:5), the product **7b** was obtained in 69% yield (49.5 mg, pale yellow oil). **^{1}H NMR** (400 MHz, $CDCl_3$): δ 7.28-7.23 (m, 2H), 6.90-6.84 (m, 3H), 3.54-3.52 (m, 4H), 2.75-2.73 (m, 4H); **^{13}C NMR** (100 MHz, $CDCl_3$): δ 151.3, 129.2, 119.8, 117.1, 52.1, 26.8;

4-(4-(*tert*-Butyl)phenyl)morpholine

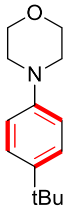

7c

7c (0.4 mmol scale) was synthesized following *the procedure B*. The reaction was carried out with 4-*tert*-butylcyclohexanone (0.0617 g, 0.4 mmol), morpholine (0.0523 g, 0.6 mmol), TEMPO (0.1572 g, 1.0 mmol), molecular sieve (800 mg, 3Å MS). The reaction mixture was stirred at 120 °C in toluene (1.0 mL) for 24 h. After concentration and purification by flash chromatography on silica gel (eluent = petroleum ether/ Et_2O = 100:5), the product **7c** was obtained in 60% yield (52.9 mg, white solid). **^{1}H NMR** (400 MHz, $CDCl_3$): δ 7.30 (d, *J* = 8.8 Hz, 2H), 6.87 (d, *J* = 8.8 Hz, 2H), 3.85 (t, *J* = 4.8 Hz, 4H), 3.13 (t, *J* = 4.8 Hz, 4H), 1.29 (s, 9H); **^{13}C NMR** (100 MHz, $CDCl_3$): δ 148.9, 142.7, 125.9, 115.4, 67.0, 49.5, 33.9, 31.4.

4-(4-(*tert*-Butyl)phenyl)thiomorpholine

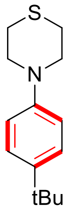

7d

7d (0.4 mmol scale) was synthesized following *the procedure B*. The reaction was carried out with 4-*tert*-butylcyclohexanone (0.0617 g, 0.4 mmol), thiomorpholine (0.0619 g, 0.6 mmol), TEMPO (0.1572 g, 1.0 mmol), molecular sieve (800 mg, 3Å MS). The reaction mixture was stirred at 120 °C in toluene (1.0 mL) for 24 h. After concentration and purification by flash chromatography on silica gel (eluent = petroleum ether/ Et_2O = 100:5), the product **7d** was obtained in 78% yield (73.2 mg, white solid). **^{1}H NMR** (400 MHz, $CDCl_3$): δ 7.28 (d, *J* = 8.8 Hz, 2H), 6.84 (d, *J* = 8.8 Hz, 2H), 3.49-3.47 (m, 4H), 2.75-2.73 (m, 4H), 1.29 (s, 9H); **^{13}C NMR** (100 MHz, $CDCl_3$): δ 149.2, 142.7, 125.96, 116.97, 52.4, 33.9, 31.4, 27.1.

4-([1,1'-Biphenyl]-4-yl)thiomorpholine

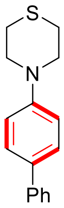

7e

7e (0.4 mmol scale) was synthesized following *the procedure B*. The reaction was carried out with 4-phenylcyclohexanone (0.0697 g, 0.4 mmol), thiomorpholine (0.0619 g, 0.6 mmol), TEMPO (0.1572 g, 1.0 mmol), molecular sieve (800 mg, 3Å MS). The reaction mixture was stirred at 120 °C in toluene (1.0 mL) for 24 h. After concentration and purification by flash chromatography on silica gel (eluent = petroleum ether/ Et_2O = 100:5), the product **7e** was obtained in 73% yield (74.7mg, pale yellow solid). **^{1}H NMR** (400 MHz, $CDCl_3$): δ 7.54 (d, J = 7.3 Hz, 2H), 7.50 (d, J = 8.7 Hz, 2H), 7.41-7.37 (m, 2H), 7.27 (t, J = 7.3 Hz, 1H), 6.93 (d, J = 8.6 Hz, 2H), 3.57 (t, J = 4.8 Hz, 4H), 2.73 (t, J = 4.8 Hz, 4H); **^{13}C NMR** (100 MHz, $CDCl_3$): δ 150.3, 140.7, 132.2, 128.6, 127.8, 126.4, 116.95, 51.8, 26.6.

Methyl 3'-thiomorpholino-[1,1'-biphenyl]-4-carboxylate

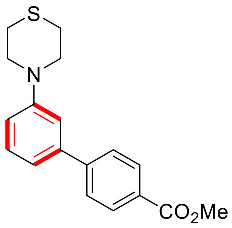

7f

7f (0.4 mmol scale) was synthesized following *the procedure B*. The reaction was carried out with ethyl 4-(3-oxocyclohexyl)benzoate (0.0928 g, 0.4 mmol), thiomorpholine (0.0619 g, 0.6 mmol), TEMPO (0.1572 g, 1.0 mmol), molecular sieve (800 mg, 3Å MS). The reaction mixture was stirred at 120 °C in toluene (1.0 mL) for 24 h. After concentration and purification by flash chromatography on silica gel (eluent = petroleum ether/ Et_2O = 100:15), the product **7f** was obtained in 75% yield (93.8 mg, white solid). **^{1}H NMR** (400 MHz, $CDCl_3$): δ 8.08 (d, J = 8.3 Hz, 2H), 7.62 (d, J = 8.3 Hz, 2H), 7.35-7.31 (m, 1H), 7.09-7.07 (m, 2H), 6.91 (d, J = 7.7 Hz, 1H), 3.92 (s, 3H), 3.60-3.58 (m, 4H), 2.77-2.74 (m, 4H); **^{13}C NMR** (100 MHz, $CDCl_3$): δ

166.8, 151.7, 145.9, 141.1, 129.9, 129.7, 128.7, 127.0, 118.8, 116.7, 115.9, 52.03, 51.99, 26.7.

4-(*m*-Tolyl)thiomorpholine

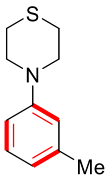

7g

7g (0.4 mmol scale) was synthesized following *the procedure B*. The reaction was carried out with ethyl 3-methylcyclohexanone (0.0449 g, 0.4 mmol), thiomorpholine (0.0619 g, 0.6 mmol), TEMPO (0.1572 g, 1.0 mmol), molecular sieve (800 mg, 3Å MS). The reaction mixture was stirred at 120 °C in toluene (1.0 mL) for 24 h. After concentration and purification by flash chromatography on silica gel (eluent = petroleum ether/ Et_2O = 100:5), the product **7g** was obtained in 70% yield (53.9 mg, pale yellow oil). **^{1}H NMR** (400 MHz, $CDCl_3$): δ 7.16-7.12 (m, 1H), 6.71-6.67 (m, 3H), 3.52-3.49 (m, 4H), 2.74-2.72 (m, 4H), 2.31 (s, 3H); **^{13}C NMR** (100 MHz, $CDCl_3$): δ 151.4, 138.9, 129.0, 120.7, 118.0, 114.2, 52.2, 26.9, 21.7.

Ethyl 4-thiomorpholinobenzoate

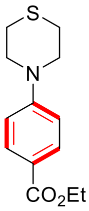

7h

7h (0.4 mmol scale) was synthesized following *the procedure B*. The reaction was carried out with ethyl 4-oxocyclohexanecarboxylate (0.0681 g, 0.4 mmol), thiomorpholine (0.0619 g, 0.6 mmol), TEMPO (0.1572 g, 1.0 mmol), molecular sieve (800 mg, 3Å MS). The reaction mixture was stirred at 120 °C in toluene (1.0 mL) for 24 h. After concentration and purification by flash chromatography on silica gel (eluent = petroleum ether/ Et_2O = 100:15), the product **7h** was obtained in 61% yield (61.8 mg, colorless oil). **^{1}H NMR** (400 MHz, $CDCl_3$): δ 7.92 (d, *J* = 8.8 Hz, 2H), 6.80

(d, *J* = 8.8 Hz, 2H), 4.325 (q, *J* = 7.1 Hz, 2H), 3.75-3.73 (m, 4H), 2.70-2.67 (m, 4H), 1.36 (t, *J* = 7.1 Hz, 3H); **^{13}C NMR** (100 MHz, $CDCl_3$): δ 166.4, 152.9, 131.3, 119.6, 113.8, 60.2, 50.4, 25.7, 14.3.

Ethyl 2-(4-thiomorpholinophenyl)acetate

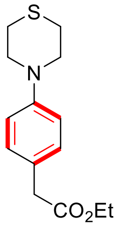

7i

7i (0.4 mmol scale) was synthesized following *the procedure B*. The reaction was carried out with ethyl 2-(4-oxocyclohexyl)acetate (0.0737 g, 0.4 mmol), thiomorpholine (0.0619 g, 0.6 mmol), TEMPO (0.1572 g, 1.0 mmol), molecular sieve (800 mg, 3Å MS). The reaction mixture was stirred at 120 °C in toluene (1.0 mL) for 24 h. After concentration and purification by flash chromatography on silica gel (eluent = petroleum ether/ Et_2O = 100:15), the product **7i** was obtained in 42% yield (45.0 mg, pale yellow oil). **^{1}H NMR** (400 MHz, $CDCl_3$): δ 7.17 (d, *J* = 8.7 Hz, 2H), 6.85 (d, *J* = 8.7 Hz, 2H), 4.14 (q, *J* = 7.1 Hz, 2H), 3.52-3.50 (m, 6H), 2.74-2.72 (m, 4H), 1.25 (t, *J* = 7.1 Hz, 3H); **^{13}C NMR** (100 MHz, $CDCl_3$): δ 171.9, 150.3, 130.0, 125.3, 117.1, 60.7, 52.1, 40.4, 26.7, 14.1.

***N*-(4-Thiomorpholinophenyl)acetamide**

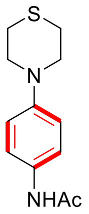

7j

7j (0.4 mmol scale) was synthesized following *the procedure B*. The reaction was carried out with 4-acetamidocyclohexanone (0.0621 g, 0.4 mmol), thiomorpholine (0.0619 g, 0.6 mmol), TEMPO (0.1572 g, 1.0 mmol), molecular sieve (800 mg, 3Å MS). The reaction mixture was stirred at 120 °C in toluene (1.0 mL) for 24 h. After concentration and purification by flash chromatography on silica gel (eluent =

petroleum ether/ Et_2O = 100:15), the product **7j** was obtained in 67% yield (62.9 mg, white solid). **^{1}H NMR** (400 MHz, $CDCl_3$): δ 7.69 (s, 1H), 7.37 (d, *J* = 8.6 Hz, 2H), 6.83 (d, *J* = 8.6 Hz, 2H), 3.46-3.44 (m, 4H), 2.74-2.72 (m, 4H), 2.12 (s, 3H); **^{13}C NMR** (100 MHz, $CDCl_3$): δ 168.5, 148.4, 130.5, 121.6, 117.7, 52.4, 26.9, 24.2.

tert-Butyl (4-thiomorpholinophenyl)carbamate

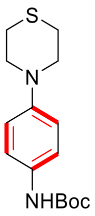

7k

7k (0.4 mmol scale) was synthesized following *the procedure B*. The reaction was carried out with 4-*N*-Boc-aminocyclohexanone (0.0853 g, 0.4 mmol), thiomorpholine (0.0619 g, 0.6 mmol), TEMPO (0.1572 g, 1.0 mmol), molecular sieve (800 mg, 3Å MS). The reaction mixture was stirred at 120 °C in toluene (1.0 mL) for 24 h. After concentration and purification by flash chromatography on silica gel (eluent = petroleum ether/ Et_2O = 100:20), the product **7k** was obtained in 55% yield (64.2 mg, white solid). **^{1}H NMR** (400 MHz, $CDCl_3$): δ 7.24 (d, *J* = 7.9 Hz, 2H), 6.84 (d, *J* = 8.8 Hz, 2H), 6.48 (brs, 1H), 3.41 (t, *J* = 4.8 Hz, 4H), 2.74 (t, *J* = 4.8 Hz, 4H), 1.50 (s, 9H); **^{13}C NMR** (100 MHz, $CDCl_3$): δ 153.1, 147.8, 131.2, 120.2, 118.3, 80.1, 52.8, 28.3, 27.0.

1-(4-(_tert_-Butyl)phenyl)piperidine

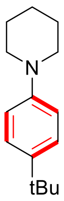

7l

7l (0.4 mmol scale) was synthesized following *the procedure B*. The reaction was carried out with 4-*tert*-butylcyclohexanone (0.0617 g, 0.4 mmol), cyclopentimine (0.0511 g, 0.6 mmol), TEMPO (0.1572 g, 1.0 mmol), molecular sieve (800 mg, 3Å MS). The reaction mixture was stirred at 120 °C in toluene (1.0 mL) for 24 h. After

concentration and purification by flash chromatography on silica gel (eluent = petroleum ether/ Et_2O = 100:5), the product **7l** was obtained in 62% yield (54.1 mg, pale yellow oil). ^{1}**H NMR** (400 MHz, $CDCl_3$): δ 7.26 (d, *J* = 8.8 Hz, 2H), 6.88 (d, *J* = 8.8 Hz, 2H), 3.12-3.09 (m, 4H), 1.72-1.67 (m, 4H), 1.57-1.53 (m, 2H), 1.28 (s, 9H); ^{13}**C NMR** (100 MHz, $CDCl_3$): δ 150.0, 141.8, 125.7, 116.2, 50.9, 33.9, 31.4, 26.0, 24.3.

1-(4-(*tert*-Butyl)phenyl)azepane

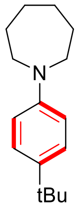

7m

7m (0.4 mmol scale) was synthesized following *the procedure B*. The reaction was carried out with 4-*tert*-butylcyclohexanone (0.0617 g, 0.4 mmol), hexamethyleneimine (0.0595 g, 0.6 mmol), TEMPO (0.1572 g, 1.0 mmol), molecular sieve (800 mg, 3Å MS). The reaction mixture was stirred at 120 °C in toluene (1.0 mL) for 24 h. After concentration and purification by flash chromatography on silica gel (eluent = petroleum ether/ Et_2O = 100:5), the product **7m** was obtained in 65% yield (59.8 mg, pale yellow oil). ^{1}**H NMR** (400 MHz, $CDCl_3$): δ 7.22 (d, *J* = 8.9 Hz, 2H), 6.63 (d, *J* = 8.9 Hz, 2H), 3.40 (t, *J* = 5.9 Hz, 4H), 1.78-1.75 (m, 4H), 1.55-1.52 (m, 4H); 1.28 (s, 9H); ^{13}**C NMR** (100 MHz, $CDCl_3$): δ 146.8, 137.6, 125.9, 110.7, 48.9, 33.6, 31.5, 28.1, 27.3.

2-(4-(*tert*-Butyl)phenyl)octahydro-1*H*-isoindole

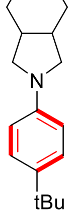

7n

7n (0.4 mmol scale) was synthesized following *the procedure B*. The reaction was carried out with 4-*tert*-butylcyclohexanone (0.0617 g, 0.4 mmol),

octahydro-1H-isoindole (0.0751 g, 0.6 mmol), TEMPO (0.1572 g, 1.0 mmol), molecular sieve (800 mg, 3Å MS). The reaction mixture was stirred at 120 °C in toluene (1.0 mL) for 24 h. After concentration and purification by flash chromatography on silica gel (eluent = petroleum ether/ Et_2O = 100:5), the product **7n** was obtained in 45% yield (45.9 mg, pale yellow solid). **^{1}H NMR** (400 MHz, $CDCl_3$): δ 7.25 (d, *J* = 8.8 Hz, 2H), 6.47 (d, *J* = 8.8 Hz, 2H), 3.30-3.14 (m, 4H), 2.32-2.26 (m, 2H), 1.64-1.35 (m, 8H), 1.29 (s, 9H); **^{13}C NMR** (100 MHz, $CDCl_3$): δ 146.0, 137.3, 125.9, 110.6, 51.7, 37.4, 33.7, 31.6, 26.5, 23.1.

8-(4-(*tert*-Butyl)phenyl)-1,4-dioxa-8-azaspiro[4.5]decane

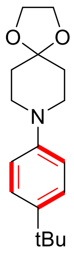

7o

7o (0.4 mmol scale) was synthesized following *the procedure B*. The reaction was carried out with 4-*tert*-butylcyclohexanone (0.0617 g, 0.4 mmol), 1,4-dioxa-8-azaspiro[4.5]decane (0.0859 g, 0.6 mmol), TEMPO (0.1572 g, 1.0 mmol), molecular sieve (800 mg, 3Å MS). The reaction mixture was stirred at 120 °C in toluene (1.0 mL) for 24 h. After concentration and purification by flash chromatography on silica gel (eluent = petroleum ether/ Et_2O = 100:10), the product **7o** was obtained in 55% yield (60.5 mg, colorless oil). **^{1}H NMR** (400 MHz, $CDCl_3$): δ 7.265 (d, *J* = 8.8 Hz, 2H), 6.89 (d, *J* = 8.8 Hz, 2H), 3.98 (s, 4H), 3.28 (t, *J* = 5.8 Hz, 4H), 1.84 (t, *J* = 5.8 Hz, 4H), 1.29 (s, 9H); **^{13}C NMR** (100 MHz, $CDCl_3$): δ 148.6, 142.1, 125.8, 116.4, 107.2, 64.25, 47.9, 34.6, 33.9, 31.4.

1-(4-(*tert*-Butyl)phenyl)-4-phenylpiperazine

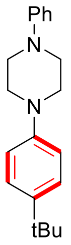

7p

7p (0.4 mmol scale) was synthesized following *the procedure B*. The reaction was carried out with 4-*tert*-butylcyclohexanone (0.0617 g, 0.4 mmol), 1-phenylpiperazine (0.0973 g, 0.6 mmol), TEMPO (0.1572 g, 1.0 mmol), molecular sieve (800 mg, 3Å MS). The reaction mixture was stirred at 120 °C in toluene (1.0 mL) for 24 h. After concentration and purification by flash chromatography on silica gel (eluent = petroleum ether/ Et_2O = 100:10), the product **7p** was obtained in 70% yield (82.4 mg, white solid). **^{1}H NMR** (400 MHz, $CDCl_3$): δ 7.33-7.25 (m, 4H), 6.98-6.86 (m, 5H), 3.31 (brs, 8H), 1.30 (s, 9H); **^{13}C NMR** (100 MHz, $CDCl_3$): δ 151.2, 148.8, 142.7, 129.1, 125.9, 119.9, 116.3, 116.0, 49.6, 49.4, 33.9, 31.4.

1-(4-(*tert*-Butyl)phenyl)-4-methylpiperazine

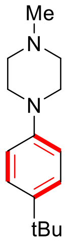

7q

7q (0.4 mmol scale) was synthesized following *the procedure B*. The reaction was carried out with 4-*tert*-butylcyclohexanone (0.0617 g, 0.4 mmol), 1-methylpiperazine (0.0601 g, 0.6 mmol), TEMPO (0.1572 g, 1.0 mmol), molecular sieve (800 mg, 3Å MS). The reaction mixture was stirred at 120 °C in toluene (1.0 mL) for 24 h. After concentration and purification by flash chromatography on silica gel (eluent = etroleum ether/EtOAc/Et_3N = 80:15:5), the product **7q** was obtained in 54% yield (50.1 mg, pale yellow solid). **^{1}H NMR** (400 MHz, $CDCl_3$): δ 7.28 (d, *J* = 8.8 Hz, 2H), 6.88 (d, *J* = 8.8 Hz, 2H), 3.20-3.17 (m, 4H), 2.58-2.56 (m, 4H), 2.34 (s, 3H), 1.29 (s, 9H); **^{13}C NMR** (100 MHz, $CDCl_3$): δ 148.9, 142.3, 125.8, 115.7, 55.2, 49.2, 46.1, 33.9, 31.4.

4-(*tert*-Butyl)-*N*,*N*-dipropylaniline

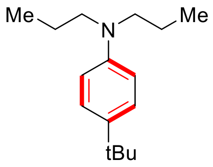

7r

7r (0.4 mmol scale) was synthesized following *the procedure B*. The reaction was carried out with 4-*tert*-butylcyclohexanone (0.0617 g, 0.4 mmol), dipropylamine (0.0607 g, 0.6 mmol), TEMPO (0.1572 g, 1.0 mmol), molecular sieve (800 mg, 3Å MS). The reaction mixture was stirred at 120 °C in toluene (1.0 mL) for 24 h. After concentration and purification by flash chromatography on silica gel (eluent = petroleum ether/Et_2O = 100:5), the product **7r** was obtained in 69% yield (64.5 mg, pale yellow oil). **^{1}H NMR** (400 MHz, $CDCl_3$): δ 7.22 (d, *J* = 8.8 Hz, 2H), 6.59 (d, *J* = 8.8 Hz, 2H), 3.20 (t, *J* = 7.6 Hz, 4H), 1.64-1.55 (m, 4H), 1.28 (s, 9H), 0.91 (t, *J* = 7.4 Hz, 6H); **^{13}C NMR** (100 MHz, $CDCl_3$): δ 146.0, 137.6, 125.9, 111.3, 53.0, 33.6, 31.5, 20.5, 11.5.

4-(*tert*-Butyl)-*N*,*N*-bis(2-methoxyethyl)aniline

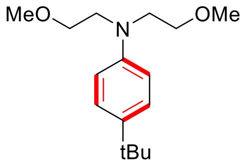

7s

7s (0.4 mmol scale) was synthesized following *the procedure B*. The reaction was carried out with 4-*tert*-butylcyclohexanone (0.0617 g, 0.4 mmol), bis(2-methoxyethyl)amine (0.0799 g, 0.6 mmol), TEMPO (0.1572 g, 1.0 mmol), molecular sieve (800 mg, 3Å MS). The reaction mixture was stirred at 120 °C in toluene (1.0 mL) for 24 h. After concentration and purification by flash chromatography on silica gel (eluent = petroleum ether/Et_2O = 100:10), the product **7s** was obtained in 70% yield (73.9 mg, pale yellow oil). **^{1}H NMR** (400 MHz, $CDCl_3$): δ 7.23 (d, *J* = 8.9 Hz, 2H), 6.66 (d, *J* = 8.9 Hz, 2H), 3.53 (s, 8H), 3.35 (s, 6H), 1.28 (s, 9H); **^{13}C NMR** (100 MHz, $CDCl_3$): δ 145.5, 138.6, 126.0, 111.3, 70.1, 58.9, 50.9, 33.6, 31.5.

***N*-Benzyl-4-(tert-butyl)-*N*-butylaniline**

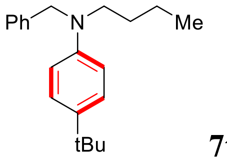

7t

7t (0.4 mmol scale) was synthesized following *the procedure B*. The reaction was carried out with 4-*tert*-butylcyclohexanone (0.0617 g, 0.4 mmol), *N*-benzyl-*N*-butylamine (0.980 g, 0.6 mmol), TEMPO (0.1572 g, 1.0 mmol), molecular sieve (800 mg, 3Å MS). The reaction mixture was stirred at 120 °C in toluene (1.0 mL) for 24 h. After concentration and purification by flash chromatography on silica gel (eluent = petroleum ether/Et_2O = 100:5), the product **7t** was obtained in 37% yield (43.9 mg, pale yellow oil). **^{1}H NMR** (400 MHz, $CDCl_3$): δ 7.32-7.19 (m, 7H), 6.62 (d, *J* = 8.8 Hz, 2H), 4.50 (s, 2H), 3.38-3.34 (m, 2H), 1.67-1.60 (m, 2H), 1.35 (q, *J* = 7.5 Hz, 2H), 1.27 (s, 9H), 0.93 (t, *J* = 7.4 Hz, 3H); **^{13}C NMR** (100 MHz, $CDCl_3$): δ 146.5, 139.6, 138.5, 128.5, 126.62, 126.59, 125.9, 111.7, 54.7, 51.0, 33.7, 31.5, 29.3, 20.4, 14.0;

7-*tert*-Butylphenoxazin-3-one

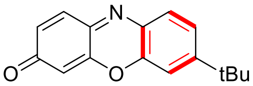

7u

7u (0.4 mmol scale) was synthesized following *the procedure B*. The reaction was carried out with 4-*tert*-butylcyclohexanone (0.0617 g, 0.4 mmol), 4-cloroaniline (0.765 0.6 mmol), TEMPO (0.3773 g, 2.4 mnol), molecular sieve (1000 mg, 3Å MS). The reaction mixture was stirred at 120 °C in toluene (1.0 mL) for 24 h. After concentration and purification by flash chromatography on silica gel (eluent = petroleum ether/ ethyl acetate = 100:5), the product **7u** was obtained in 54% yield (55.8 mg, orange crystal). **^{1}H NMR** (400 MHz, $CDCl_3$): δ 7.73 (d, *J* = 8.4 Hz, 1H), 7.45-7.41 (m, 2H), 7.35 (d, *J* = 1.8 Hz, 1H), 6.86 (dd, *J* = 9.8 Hz, 1.8 Hz, 1H), 6.33 (d, *J* = 1.8 Hz, 1H), 1.38 (s, 9H); **^{13}C NMR** (100 MHz, $CDCl_3$): δ 186.4, 158.0, 150.0, 147.8, 143.7, 134.8, 134.7, 131.4, 129.8, 123.1, 113.0, 106.7, 35.6, 31.0.

X-ray Data

Crystal data of product 3a.

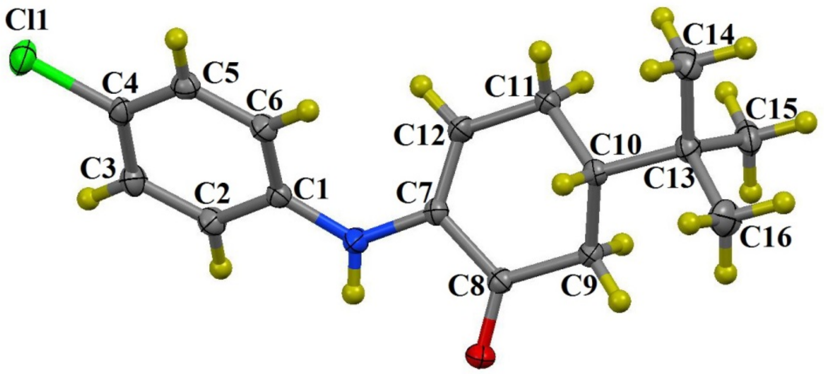

Supplementary Figure 3. X-ray structure of **3a**. Hydrogen atoms have been omitted for clarity. The thermal ellipsoid was drawn at the 50% probability level. (**CCDC 1816522**)

Identification code	full	
Empirical formula	C16 H20 Cl N O	
Formula weight	277.78	
Temperature	150(2) K	
Wavelength	0.71073 Å	
Crystal system	Orthorhombic	
Space group	Pbcn	
Unit cell dimensions	a = 27.949(2) Å	a= 90°.
	b = 9.3887(8) Å	b= 90°.
	c = 11.0135(8) Å	g = 90°.
Volume	2890.0(4) Å^3	
Z	8	
Density (calculated)	1.277 Mg/m^3	
Absorption coefficient	0.256 mm^{-1}	
F(000)	1184	
Crystal size	0.24 x 0.18 x 0.16 mm^3	
Theta range for data collection	2.29 to 27.50°.	
Index ranges	-35<=h<=36, -12<=k<=9, -14<=l<=13	

Reflections collected	22836
Independent reflections	3312 [R(int) = 0.0266]
Completeness to theta = 27.50°	99.9 %
Absorption correction	Semi-empirical from equivalents
Max. and min. transmission	0.9601 and 0.9410
Refinement method	Full-matrix least-squares on F^2
Data / restraints / parameters	3312 / 0 / 172
Goodness-of-fit on F^2	1.263
Final R indices [I>2sigma(I)]	R1 = 0.0453, wR2 = 0.1375
R indices (all data)	R1 = 0.0507, wR2 = 0.1454
Largest diff. peak and hole	0.582 and -0.758 e.$Å^{-3}$

Crystal data of product 3ad.

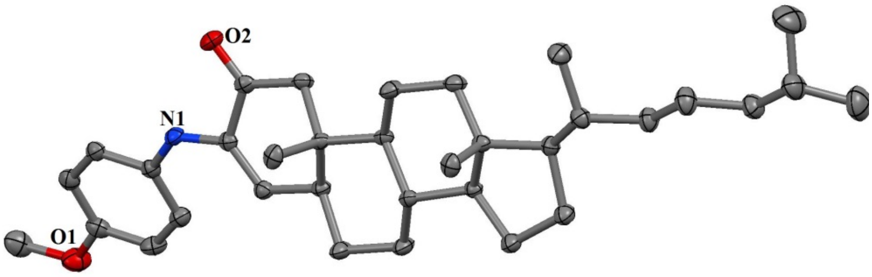

Supplementary Figure 4. X-ray structure of **3ad**. The thermal ellipsoid was drawn at the 50% probability level. (**CCDC 1816524**)

Identification code	full	
Empirical formula	C34 H51 N O2	
Formula weight	505.76	
Temperature	100(2) K	
Wavelength	0.71073 Å	
Crystal system	Triclinic	
Space group	P1	
Unit cell dimensions	a = 9.5297(14) Å	a= 81.641(6)°.
	b = 12.383(2) Å	b= 89.200(6)°.
	c = 25.595(4) Å	g = 88.955(6)°.
Volume	2987.5(8) $Å^3$	

Z	4
Density (calculated)	1.124 Mg/m^3
Absorption coefficient	0.068 mm^{-1}
F(000)	1112
Crystal size	0.22 x 0.22 x 0.10 mm^3
Theta range for data collection	2.14 to 28.75°.
Index ranges	-12<=h<=12, -16<=k<=16, -34<=l<=34
Reflections collected	95104
Independent reflections	30719 [R(int) = 0.0381]
Completeness to theta = 28.75°	99.7 %
Absorption correction	Semi-empirical from equivalents
Max. and min. transmission	0.9932 and 0.9852
Refinement method	Full-matrix least-squares on F^2
Data / restraints / parameters	30719 / 3273 / 1333
Goodness-of-fit on F^2	1.054
Final R indices [I>2sigma(I)]	R1 = 0.0464, wR2 = 0.1210
R indices (all data)	R1 = 0.0534, wR2 = 0.1260
Absolute structure parameter	-0.1(5)
Largest diff. peak and hole	0.422 and -0.440 e.Å^{-3}

Crystal data of product 3ae.

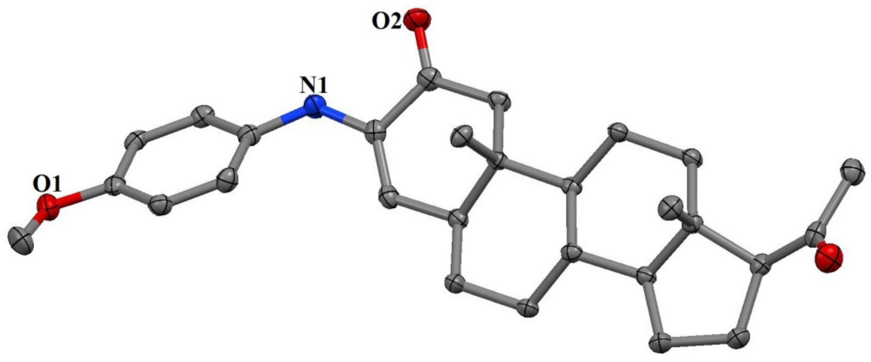

Supplementary Figure 5. X-ray structure of **3ae**. The thermal ellipsoid was drawn at the 50% probability level. (**CCDC 1816525**)

Identification code	full
Empirical formula	C28 H37 N O3
Formula weight	435.59
Temperature	446(2) K
Wavelength	1.54184 Å
Crystal system	Orthorhombic
Space group	$P2_12_12_1$
Unit cell dimensions	a = 7.44008(9) Å a= 90°.
	b = 14.57773(17) Å b= 90°.
	c = 21.4248(2) Å g = 90°.
Volume	2323.72(5) Å^3
Z	4
Density (calculated)	1.245 Mg/m^3
Absorption coefficient	0.624 mm^{-1}
F(000)	944
Crystal size	0.48 x 0.28 x 0.18 mm^3
Theta range for data collection	3.67 to 70.00°.
Index ranges	-8<=h<=6, -17<=k<=17, -26<=l<=25
Reflections collected	12297
Independent reflections	4221 [R(int) = 0.0172]
Completeness to theta = 70.00°	99.4 %
Absorption correction	Semi-empirical from equivalents
Max. and min. transmission	0.8959 and 0.6417
Refinement method	Full-matrix least-squares on F^2
Data / restraints / parameters	4221 / 0 / 289
Goodness-of-fit on F^2	1.052
Final R indices [I>2sigma(I)]	R1 = 0.0377, wR2 = 0.0991
R indices (all data)	R1 = 0.0392, wR2 = 0.1002
Absolute structure parameter	0.1(2)
Largest diff. peak and hole	0.342 and -0.324 e.Å^{-3}

Crystal data of product 7u.

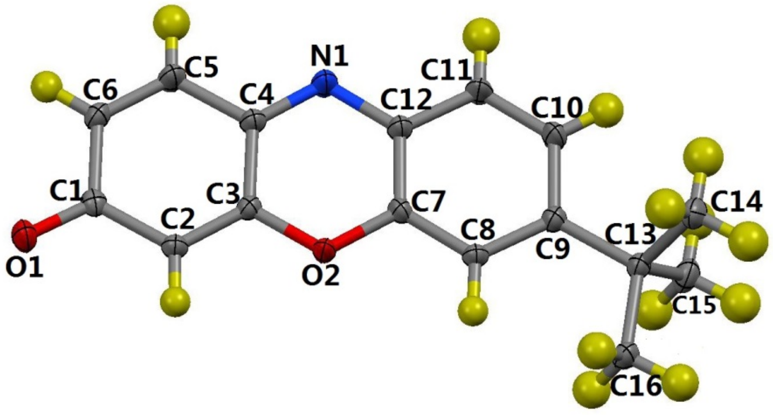

Supplementary Figure 6. X-ray structure of **7u**. Hydrogen atoms have been omitted for clarity. The thermal ellipsoid was drawn at the 50% probability level. (**CCDC 1821540**)

Identification code	full	
Empirical formula	C16 H15 N O2	
Formula weight	253.29	
Temperature	293(2) K	
Wavelength	0.71073 Å	
Crystal system	Monoclinic	
Space group	$P2_1/m$	
Unit cell dimensions	a = 6.6609(4) Å	a= 90°.
	b = 6.8533(3) Å	b= 97.561(3)°.
	c = 13.9387(7) Å	g = 90°.
Volume	630.76(6) $Å^3$	
Z	2	
Density (calculated)	1.334 Mg/m^3	
Absorption coefficient	0.088 mm^{-1}	
F(000)	268	
Crystal size	0.25 x 0.20 x 0.20 mm^3	
Theta range for data collection	2.95 to 27.56°.	
Index ranges	-8<=h<=8, -8<=k<=8, -18<=l<=18	
Reflections collected	14785	

Independent reflections	1565 [R(int) = 0.0291]
Completeness to theta = 27.56°	99.4 %
Absorption correction	Semi-empirical from equivalents
Max. and min. transmission	0.9826 and 0.9783
Refinement method	Full-matrix least-squares on F^2
Data / restraints / parameters	1565 / 0 / 112
Goodness-of-fit on F^2	1.082
Final R indices [I>2sigma(I)]	R1 = 0.0430, wR2 = 0.1310
R indices (all data)	R1 = 0.0468, wR2 = 0.1361
Largest diff. peak and hole	0.344 and -0.207 e.Å^{-3}

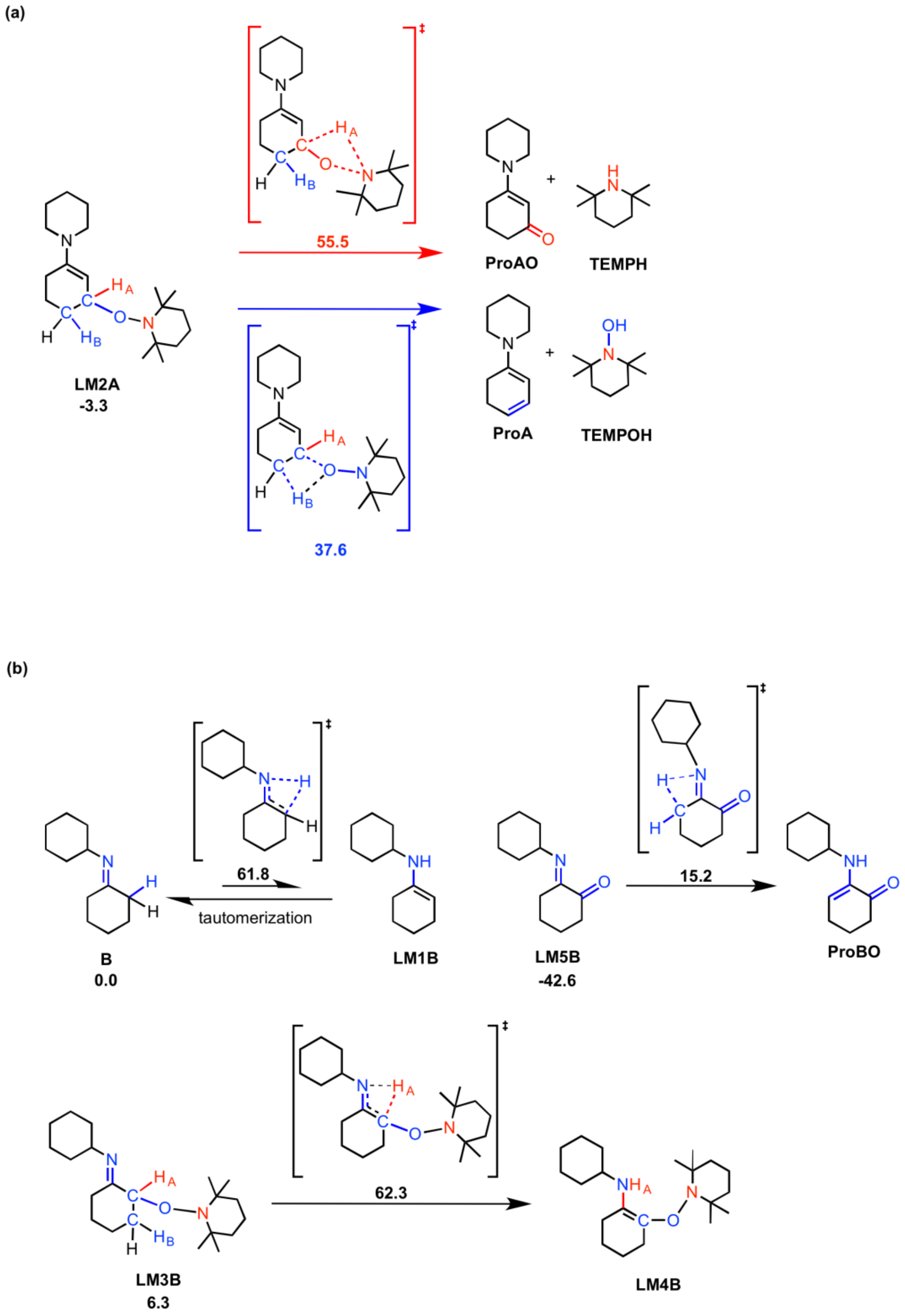

Supplementary Figure 7. Selected proton transfer processes in the absence of proton-relay chain with (a) enamine molecule **A** and (b) imine molecule **B** (Numbers represent the free energies with respect to molecule **A** and **B** respectively in kcal mol^{-1}). All transitions states feature relatively high barriers, suggesting the necessity of introducing the proper proton-relay chain to reduce the intrinsic reorganization energy of these transition states.

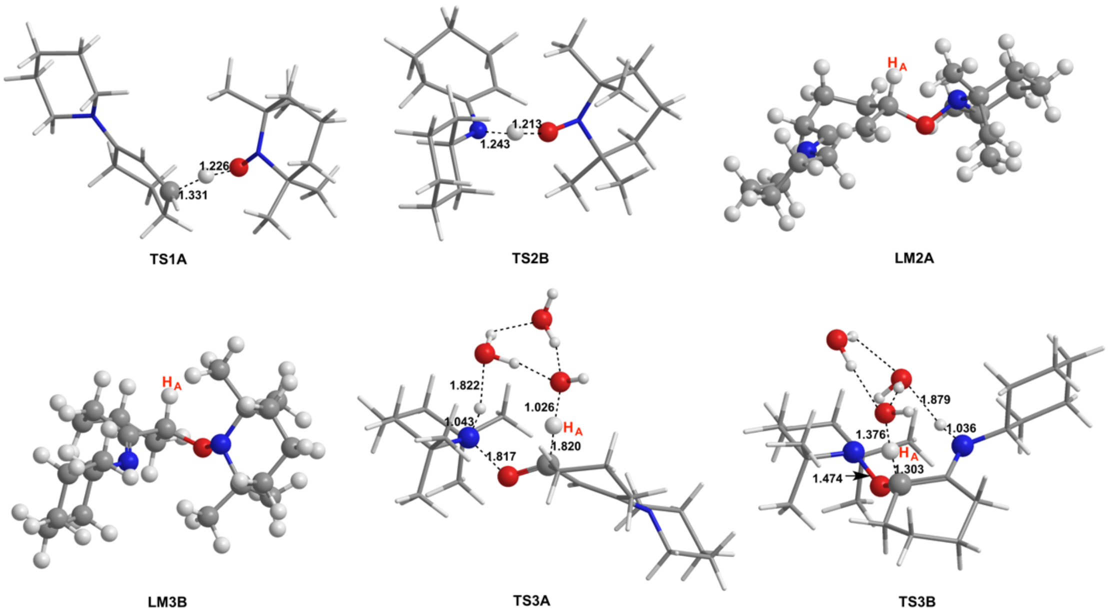

Supplementary Figure 8. Optimized structures of key stationary states (bond length in Å). Atoms of N, O, C, and H are labeled in blue, red, gray, and white, respectively. The "inert" H_A has been marked. The first hydrogen abstraction step starts from C-H of enamine A (**TS1A**), and N-H of imine B (**TS2B**), respectively, resulting in formation of aminoxylated intermediate **LM2A** and **LM3B**. Note that **LM3B** is the α-aminoxylated species detected in experiment. **TS3A** and **TS3B** are transition states for activation of "inert" C-H_A. **TS3A** corresponds to formation of amino enone, where the O-N bond (1.817 Å) is dissociated concertedly with C-H_A activation. **TS3B** corresponds to the C-H_A activation via an imine-enamine tautomerization process, where O-N bond (1.474 Å) status remains unchanged.

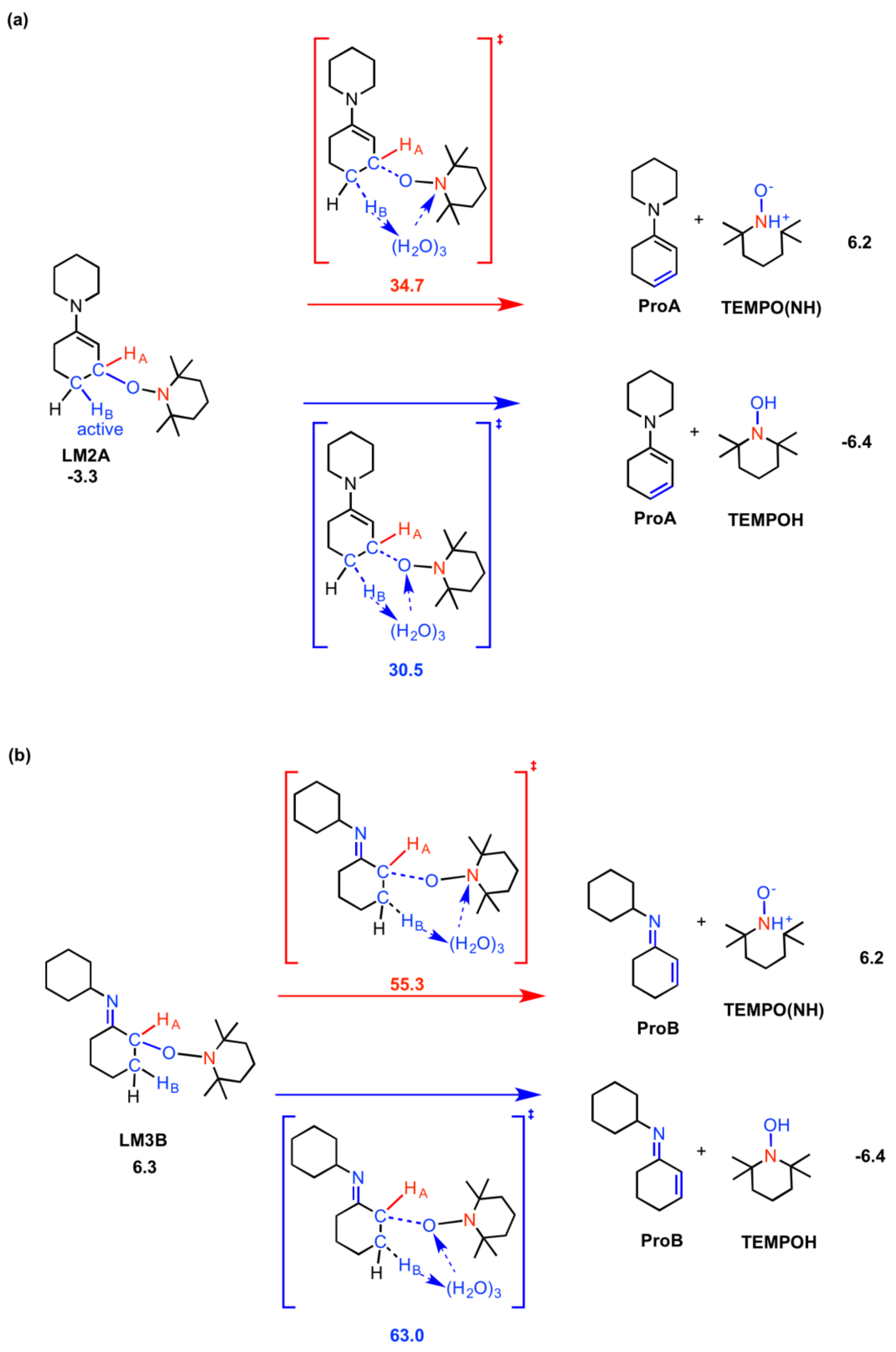

Supplementary Figure 9. Pathways for elimination of H_B/TEMPO from intermediate (a) **LM2A** and (b) **LM3B** for generation of arylamine (Numbers represent the free energies with respect to molecule **A** and **B** respectively in kcal mol^{-1}). The roles of oxygen atom and nitrogen atom of TEMPO as the reactive sites have both been considered.

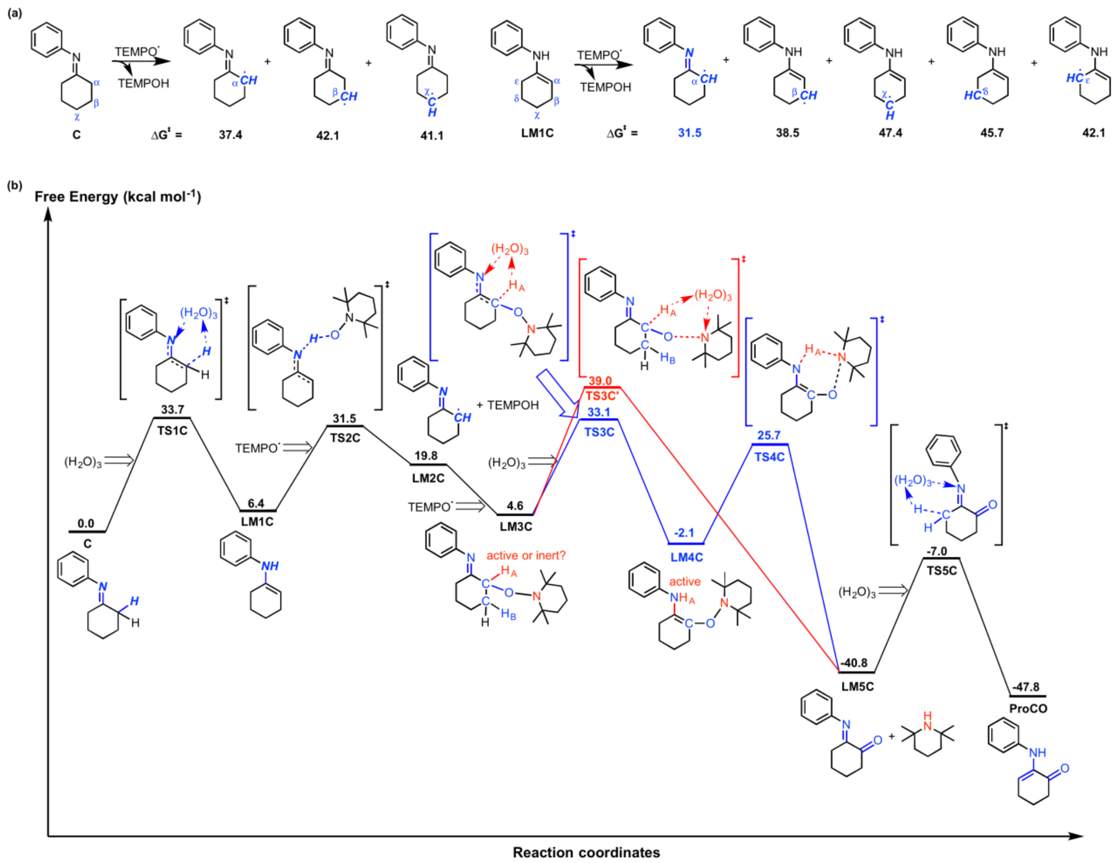

Supplementary Figure 10. Computational investigation on the mechanism for oxidation of phenyl-substituted imine by TEMPO. Numbers are Gibbs free energies (kcal mol^{-1}) with respect to the imine reagent **C** and TEMPO. (a) Activation free energies of the hydrogen abstraction of imine molecule **C** and its tautomer LM1C by TEMPO in different reactive sites. (b) Free energy profiles for oxidation of imine molecule C by TEMPO. The reactions of phenyl-substituted imine **C** and alkyl-substituted imine **B** follow the similar reaction pathway. The pathway for the elimination of H_A/piperdine via imine-enamine tautomerization is shown in blue line and the pathway for the direct elimination of H_A/piperdine is shown in red line.

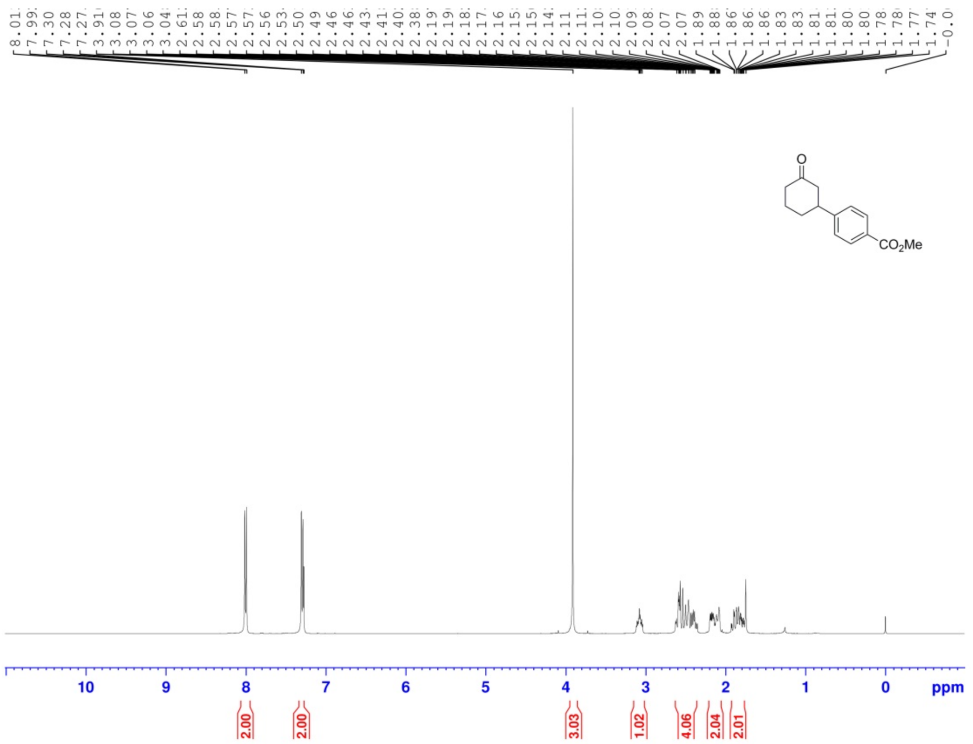

Supplementary Figure 11. ^{1}H NMR spectrum for ethyl 4-(3-oxocyclohexyl)benzoate (**1f'**).

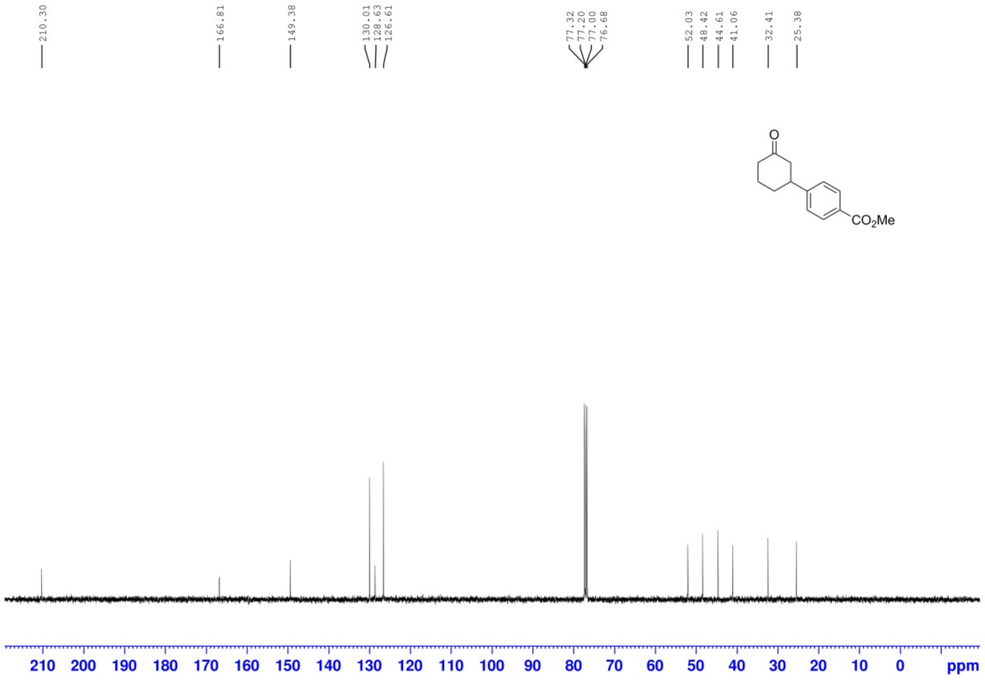

Supplementary Figure 12. ^{13}C NMR spectrum for ethyl 4-(3-oxocyclohexyl)benzoate (**1f'**).

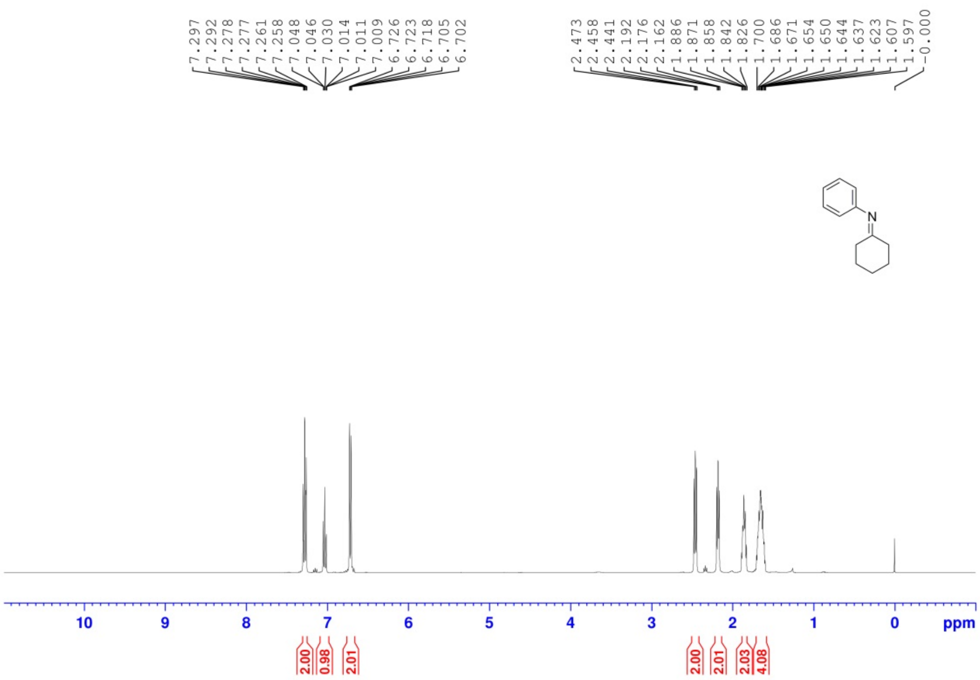

Supplementary Figure 13. ^{1}H NMR spectrum for *N*-phenylcyclohexanimine (**9**).

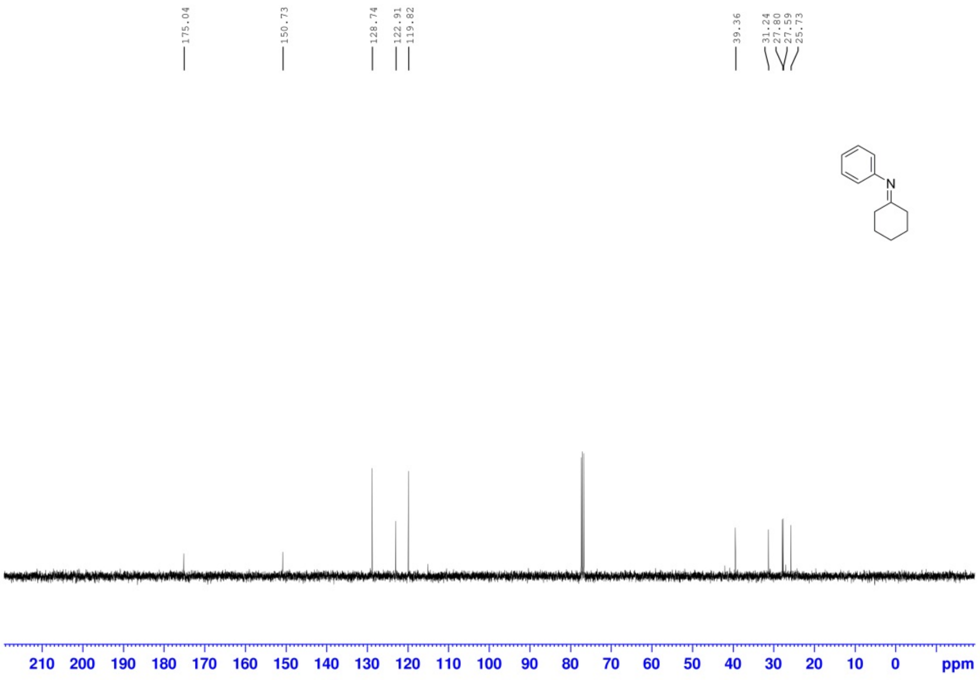

Supplementary Figure 14. ^{13}C NMR spectrum for *N*-phenylcyclohexanimine (**9**).

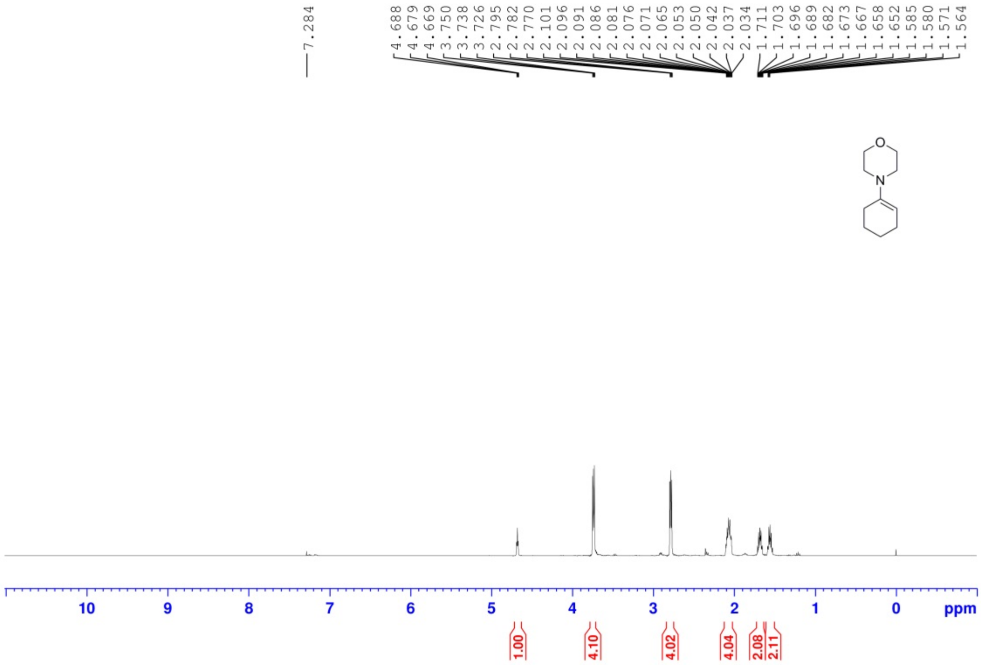

Supplementary Figure 15. ^{1}H NMR spectrum for 4-(cyclohex-1-en-1-yl)morpholine (**10**)

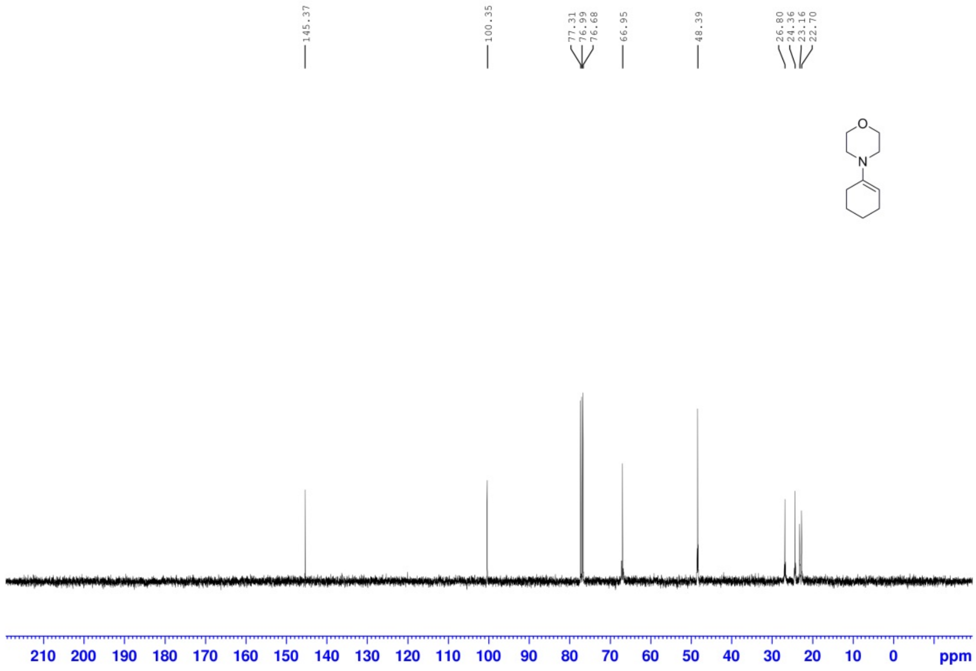

Supplementary Figure 16. ^{13}C NMR spectrum for 4-(cyclohex-1-en-1-yl)morpholine (**10**)

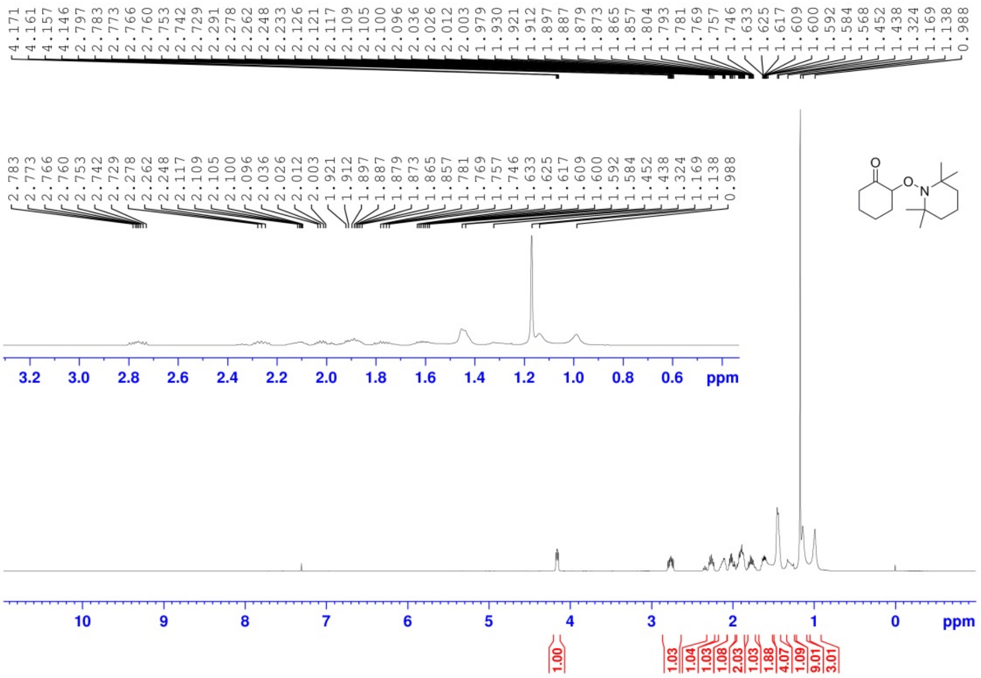

Supplementary Figure 17. ^{1}H NMR spectrum for 2-((2,2,6,6-tetramethylpiperidin-1-yl)oxy)cyclohexanone (**11**).

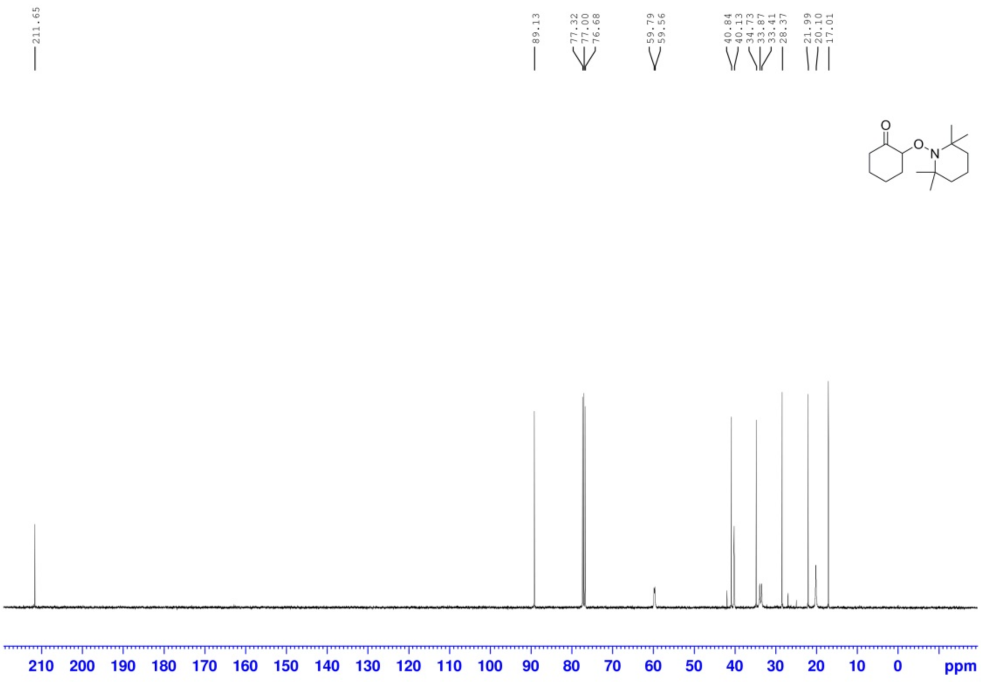

Supplementary Figure 18. ^{13}C NMR spectrum for 2-((2,2,6,6-tetramethylpiperidin-1-yl)oxy)cyclohexanone (**11**).

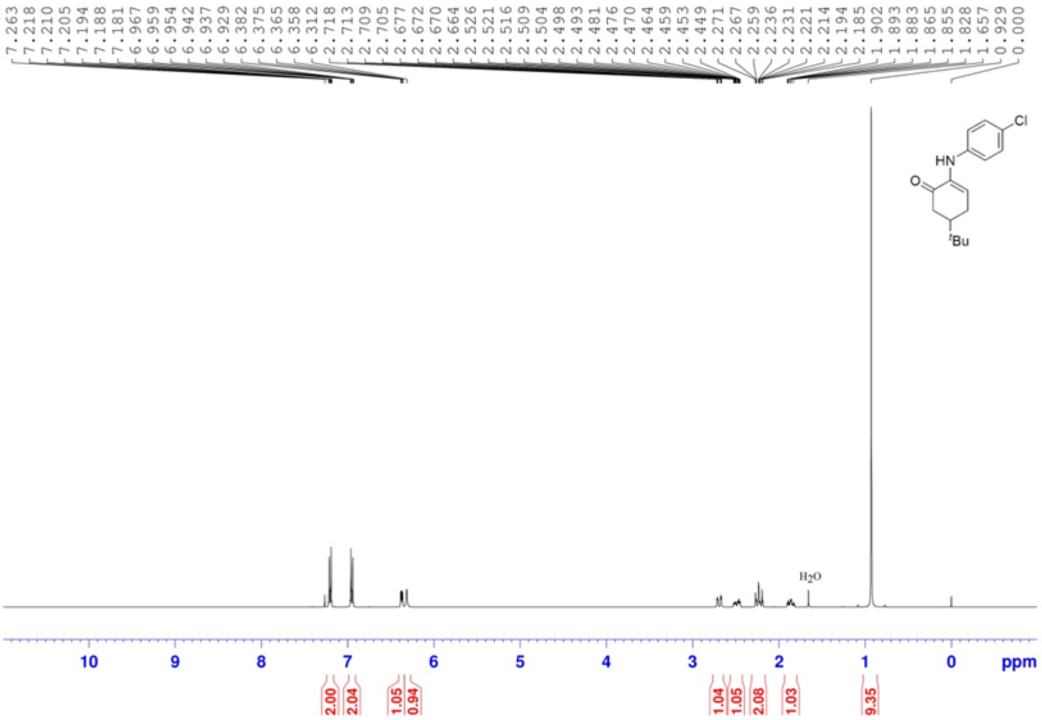

Supplementary Figure 19. ^{1}H NMR spectrum for

5-*tert*-Butyl-2-(4-chlorophenylamino)cyclohex-2-enone (**3a**).

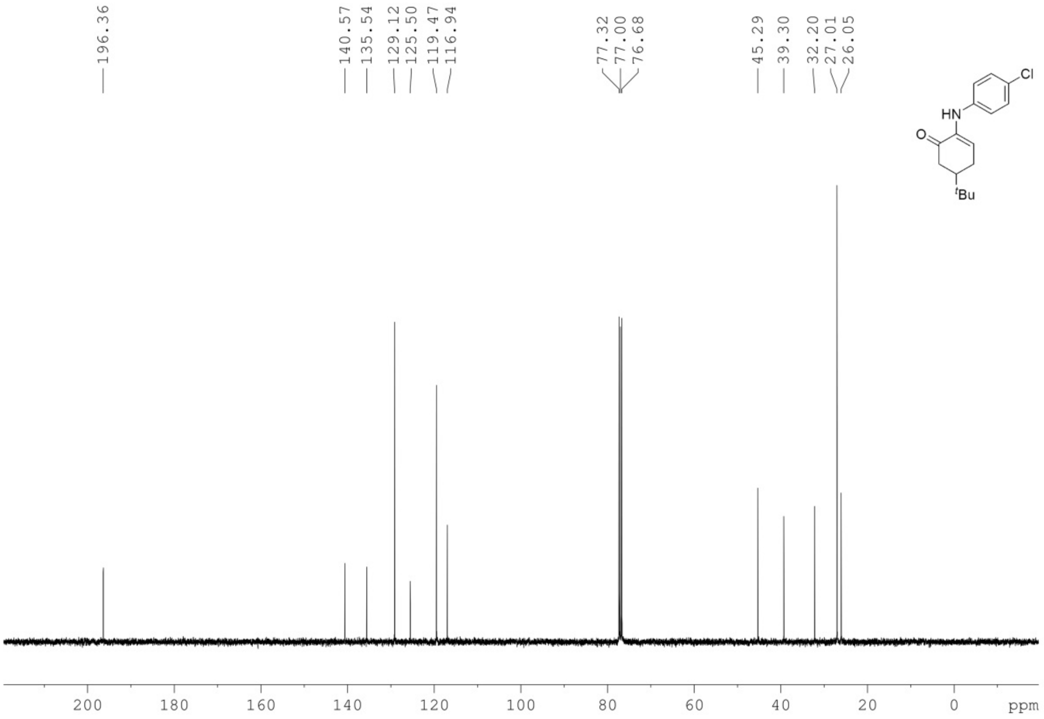

Supplementary Figure 20. ^{13}C NMR spectrum for

5-*tert*-Butyl-2-(4-chlorophenylamino)cyclohex-2-enone (**3a**).

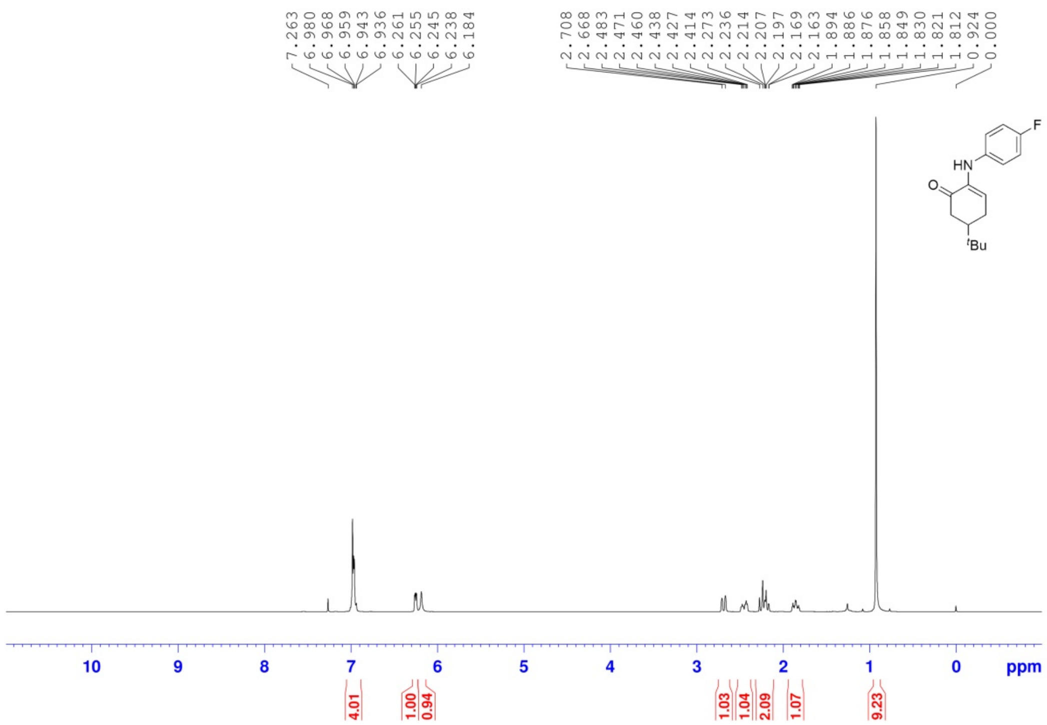

Supplementary Figure 21. ^{1}H NMR spectrum for 5-*tert*-Butyl-2-(4-fluorophenylamino)cyclohex-2-enone (**3b**).

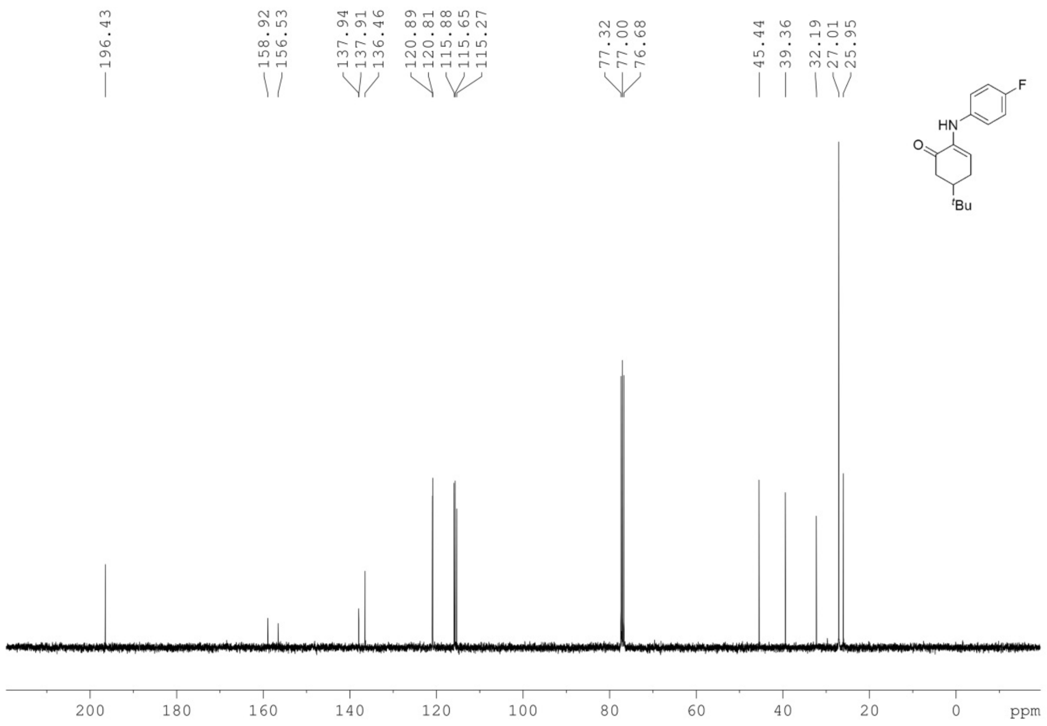

Supplementary Figure 22. ^{13}C NMR spectrum for 5-*tert*-Butyl-2-(4-fluorophenylamino)cyclohex-2-enone (**3b**).

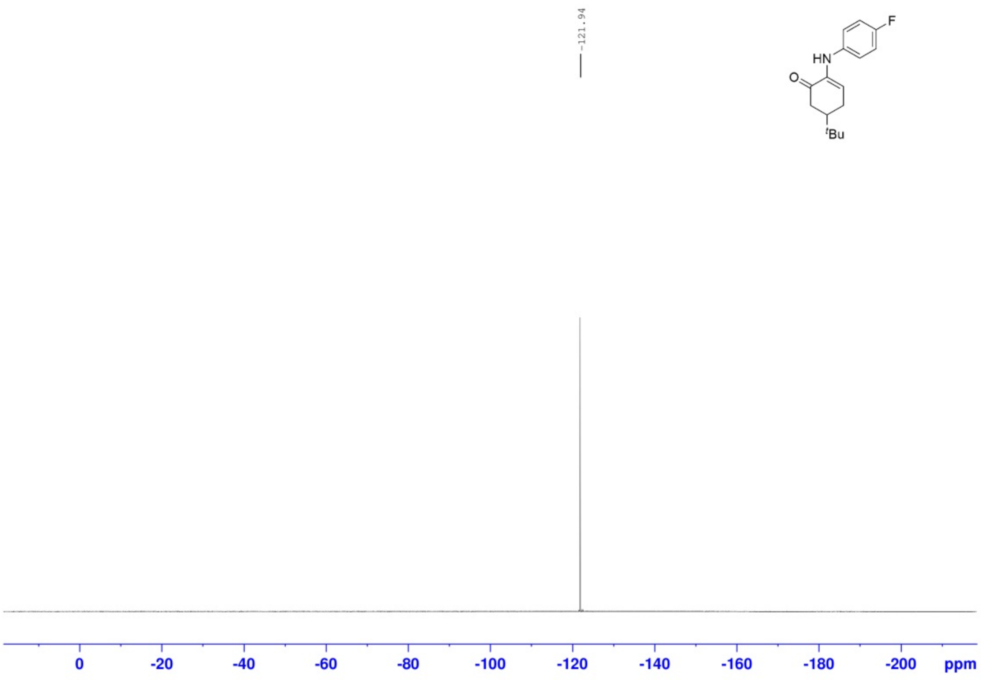

Supplementary Figure 23. ^{19}F NMR spectrum for 5-*tert*-Butyl-2-(4-fluorophenylamino)cyclohex-2-enone (**3b**).

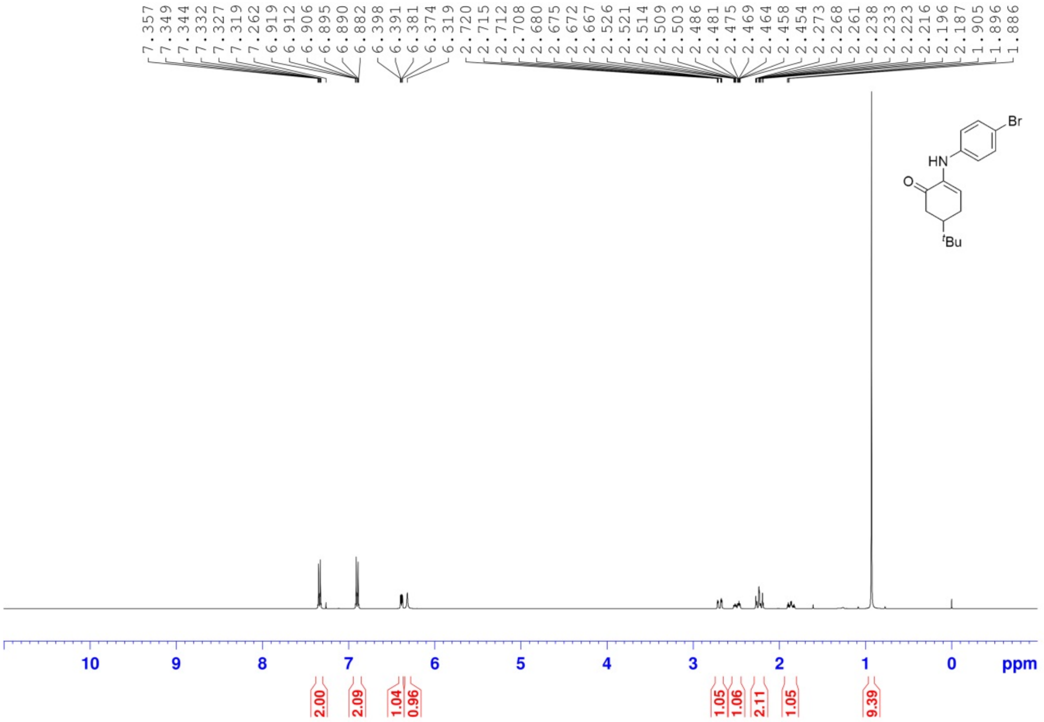

Supplementary Figure 24. ^{1}H NMR spectrum for 2-(4-bromophenylamino)-5-*tert*-butylcyclohex-2-enone (**3c**).

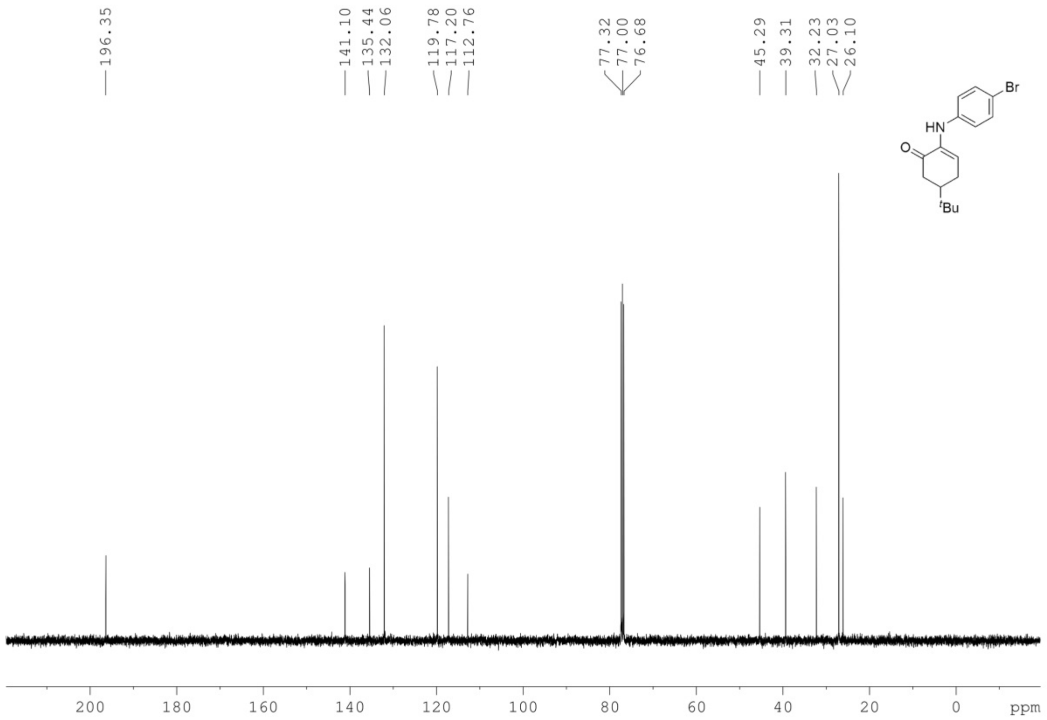

Supplementary Figure 25. ^{13}C NMR spectrum for 2-(4-bromophenylamino)-5-*tert*-butylcyclohex-2-enone (**3c**).

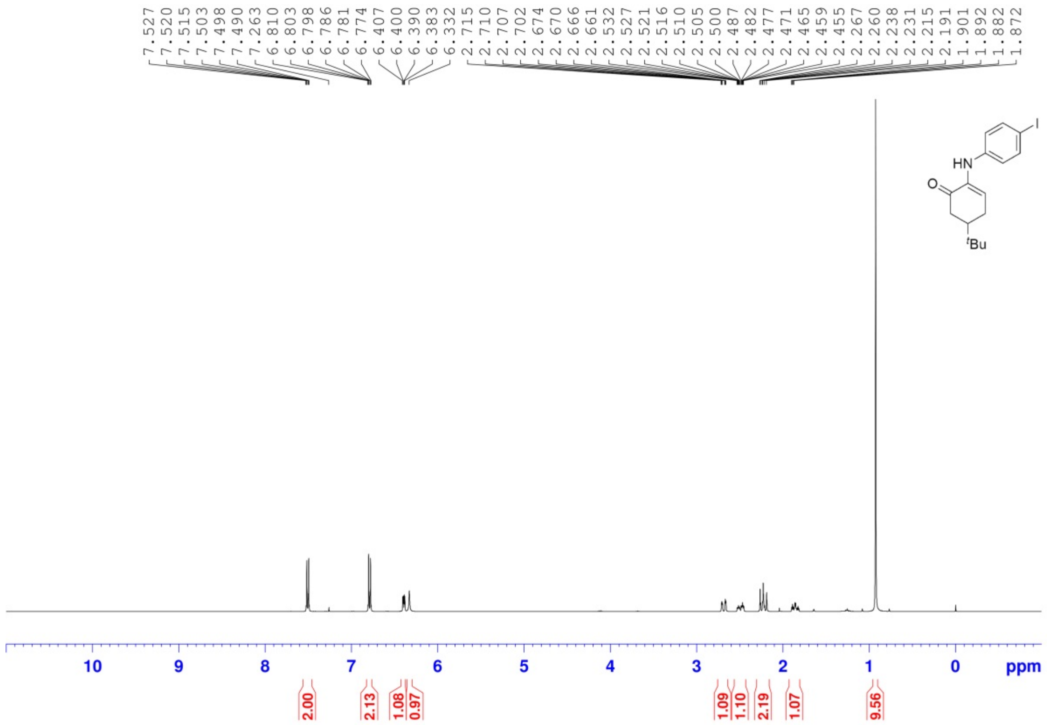

Supplementary Figure 26. ^{1}H NMR spectrum for 5-*tert*-butyl-2-(4-iodophenylamino)cyclohex-2-enone (**3d**).

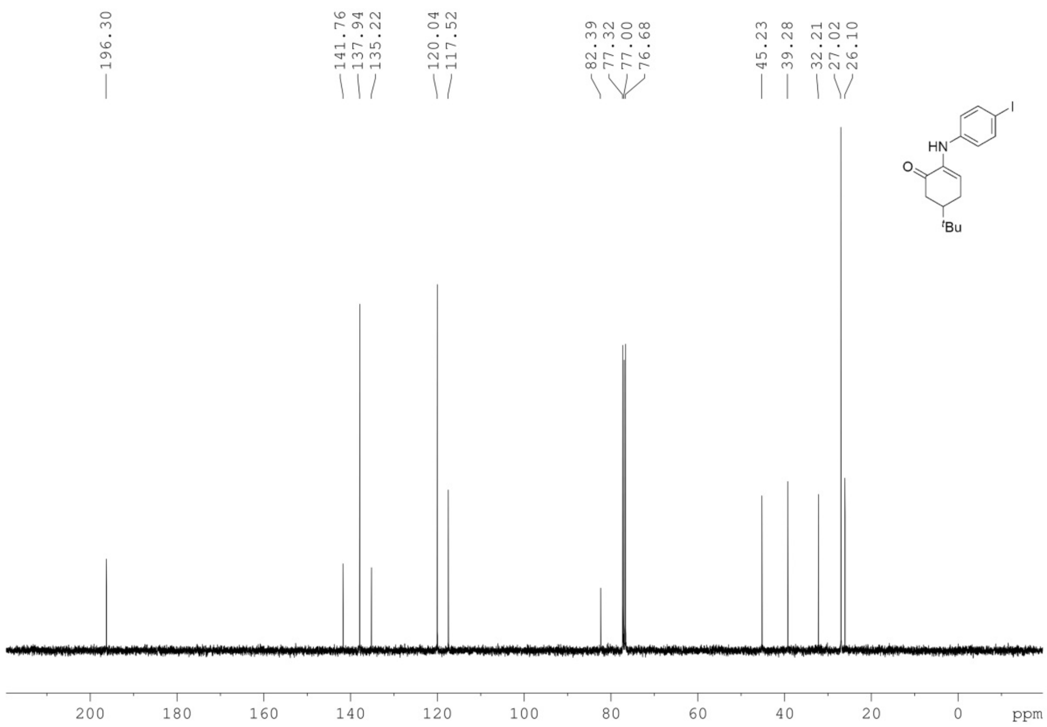

Supplementary Figure 27. ^{13}C NMR spectrum for 5-*tert*-butyl-2-(4-iodophenylamino)cyclohex-2-enone (3d).

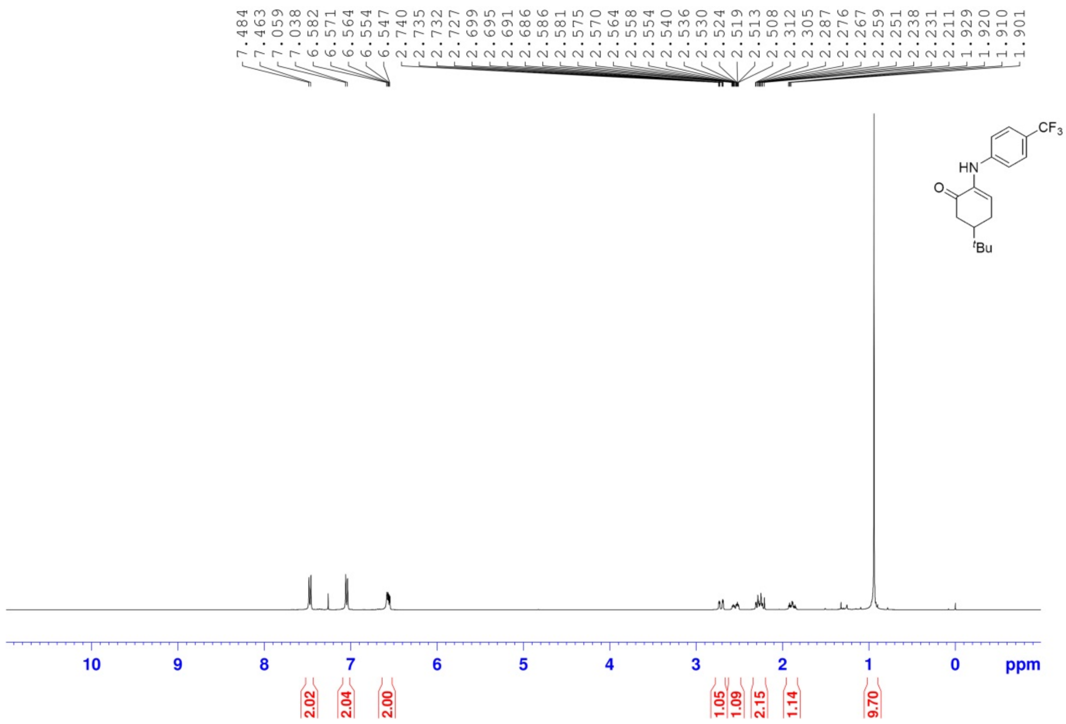

Supplementary Figure 28. ^{1}H NMR spectrum for 5-*tert*-butyl-2-(4-(trifluoromethyl)phenylamino)cyclohex-2-enone (**3e**).

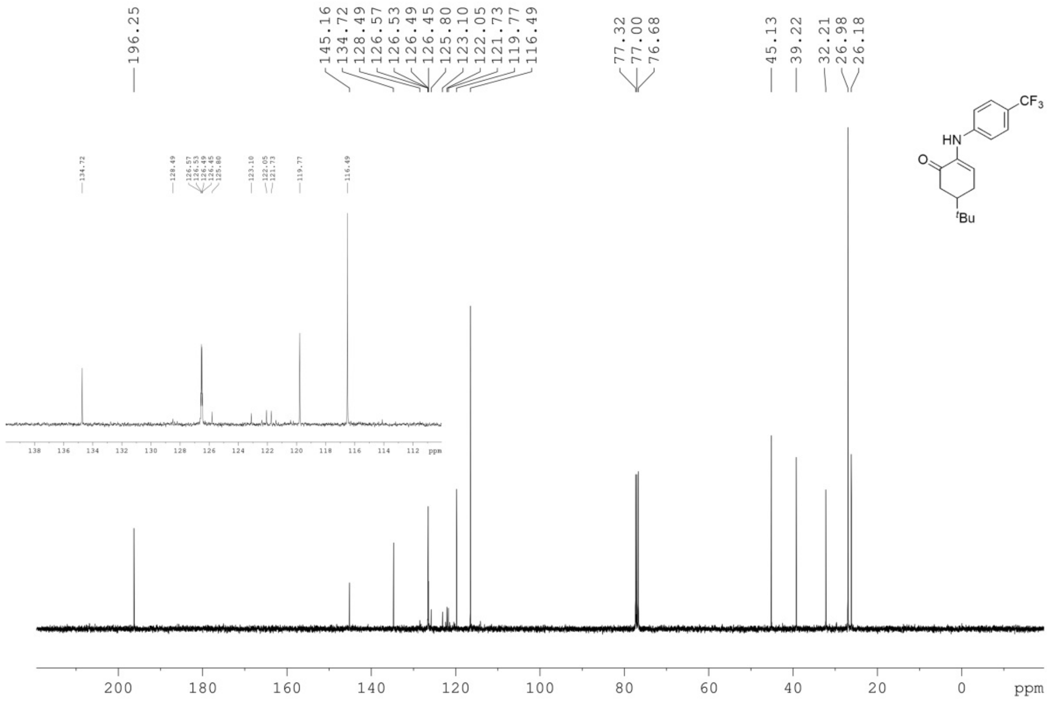

Supplementary Figure 29. ^{13}C NMR spectrum for

5-*tert*-butyl-2-(4-(trifluoromethyl)phenylamino)cyclohex-2-enone (**3e**).

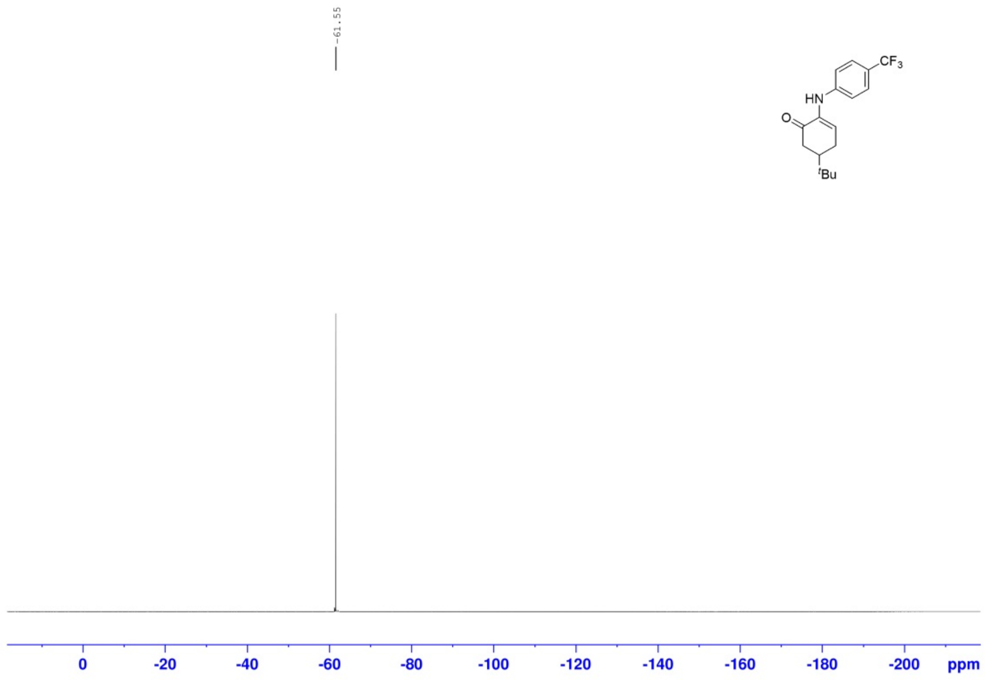

Supplementary Figure 30. ^{19}F NMR spectrum for

5-*tert*-butyl-2-(4-(trifluoromethyl)phenylamino)cyclohex-2-enone (**3e**).

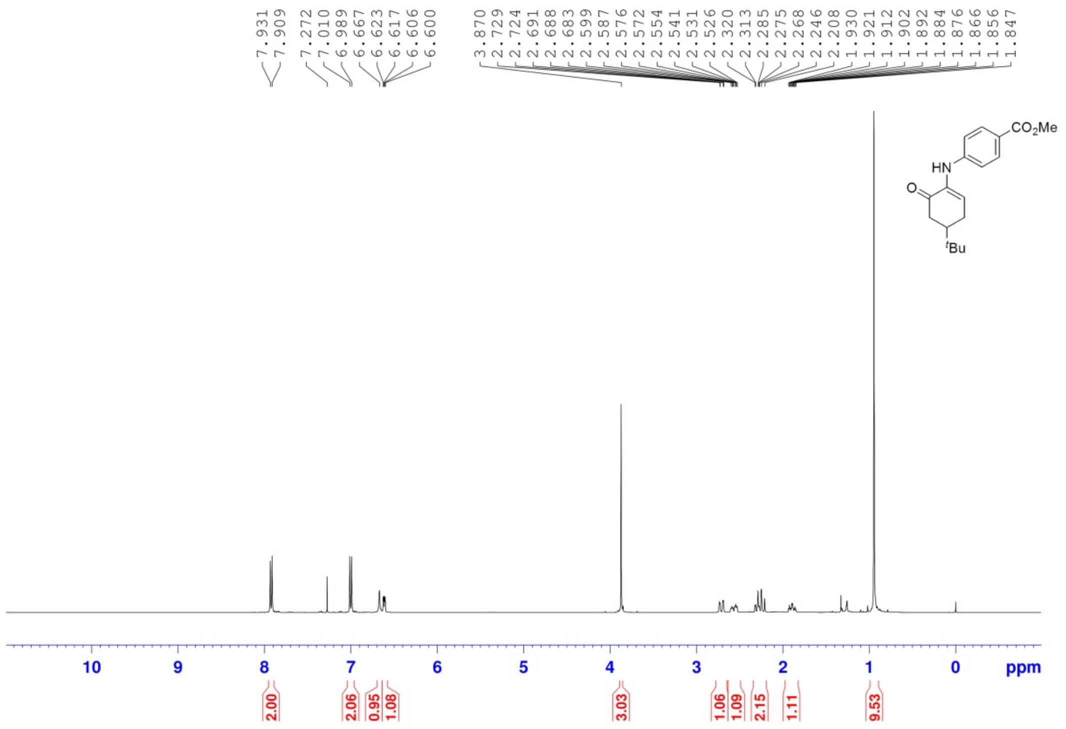

Supplementary Figure 31. ^{1}H NMR spectrum for methyl 4-(4-*tert*-butyl-6-oxocyclohex-1-enylamino)benzoate (**3f**).

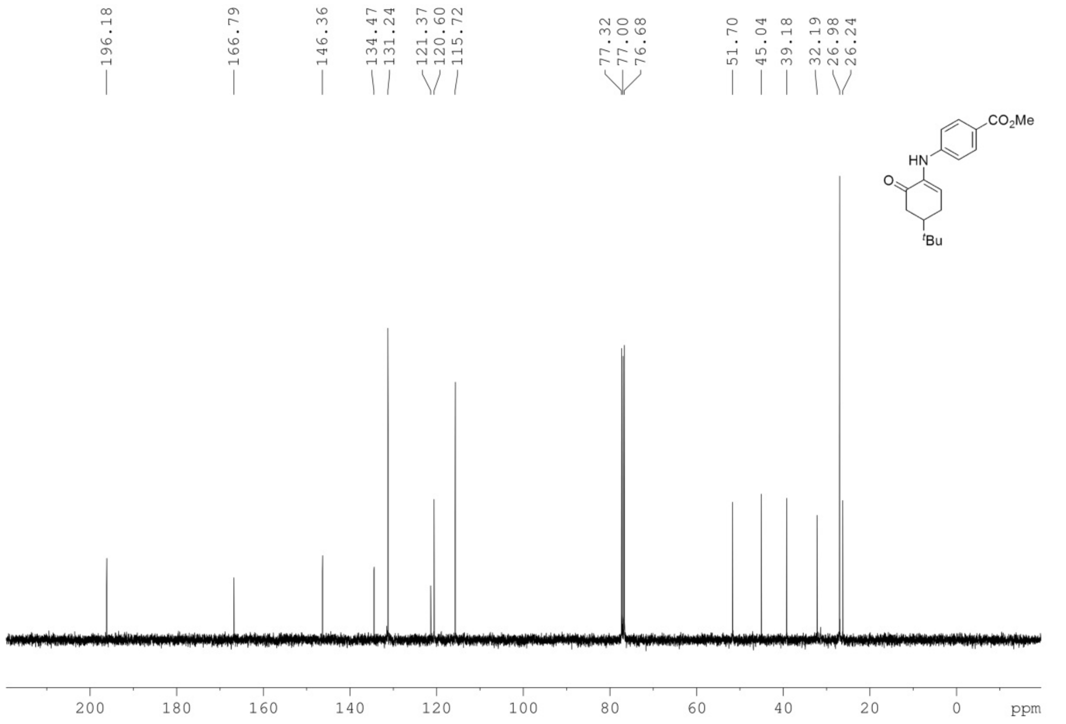

Supplementary Figure 32. ^{13}C NMR spectrum for methyl 4-(4-*tert*-butyl-6-oxocyclohex-1-enylamino)benzoate (**3f**).

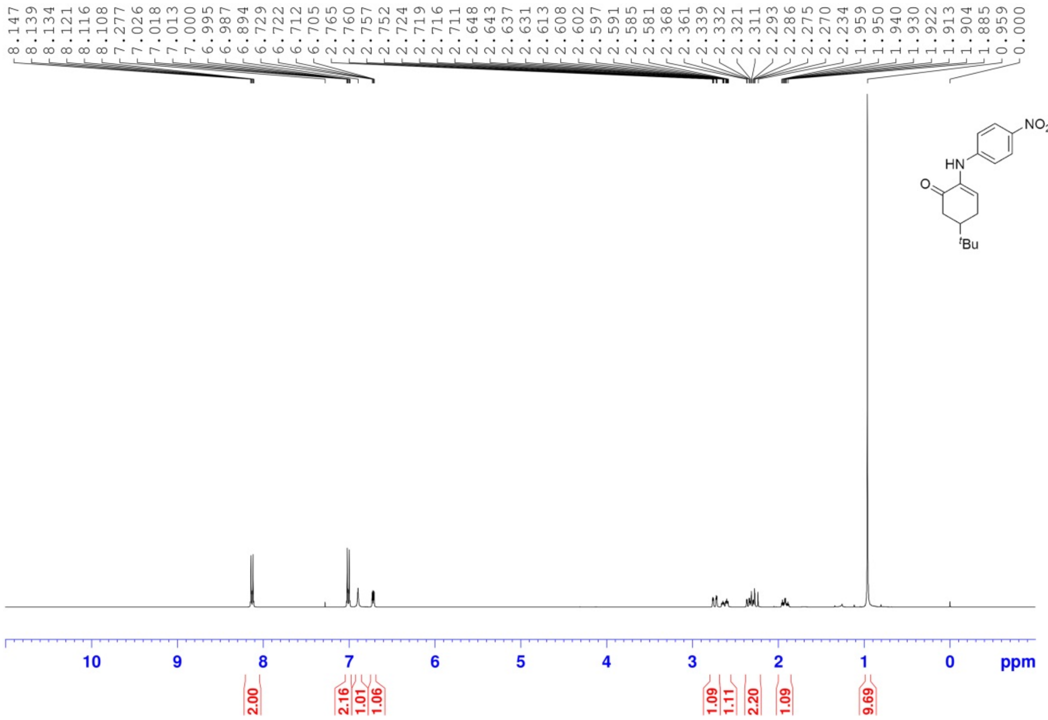

Supplementary Figure 33. ^{1}H NMR spectrum for

5-*tert*-butyl-2-(4-nitrophenylamino)cyclohex-2-enone (**3g).**

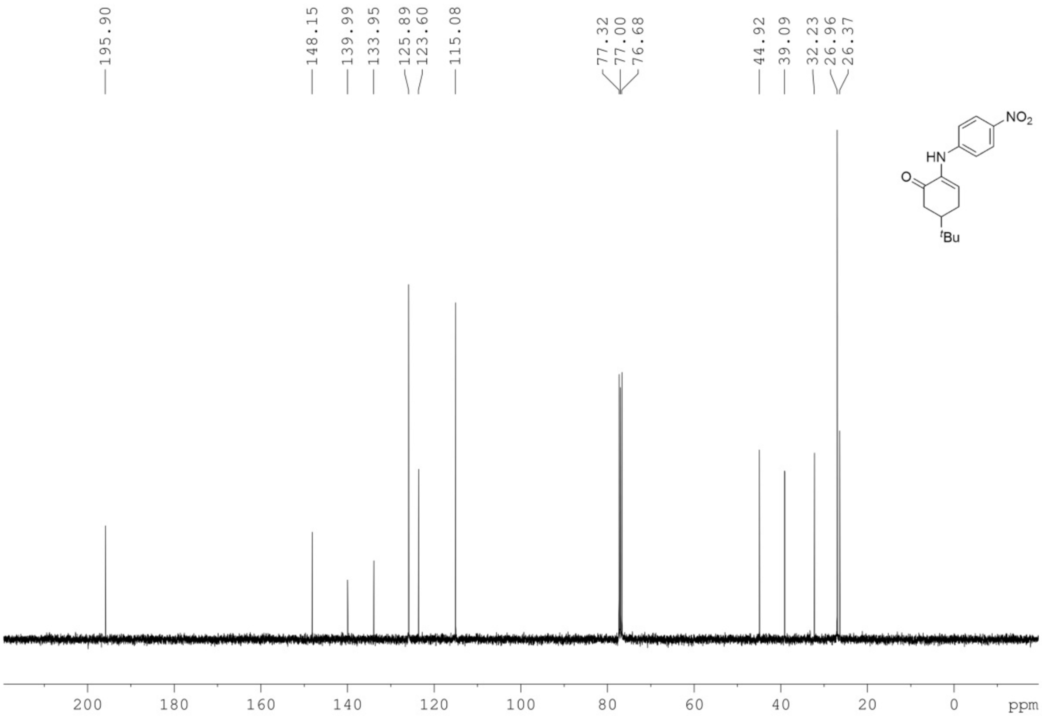

Supplementary Figure 34. ^{13}C NMR spectrum for

5-*tert*-butyl-2-(4-nitrophenylamino)cyclohex-2-enone (**3g).**

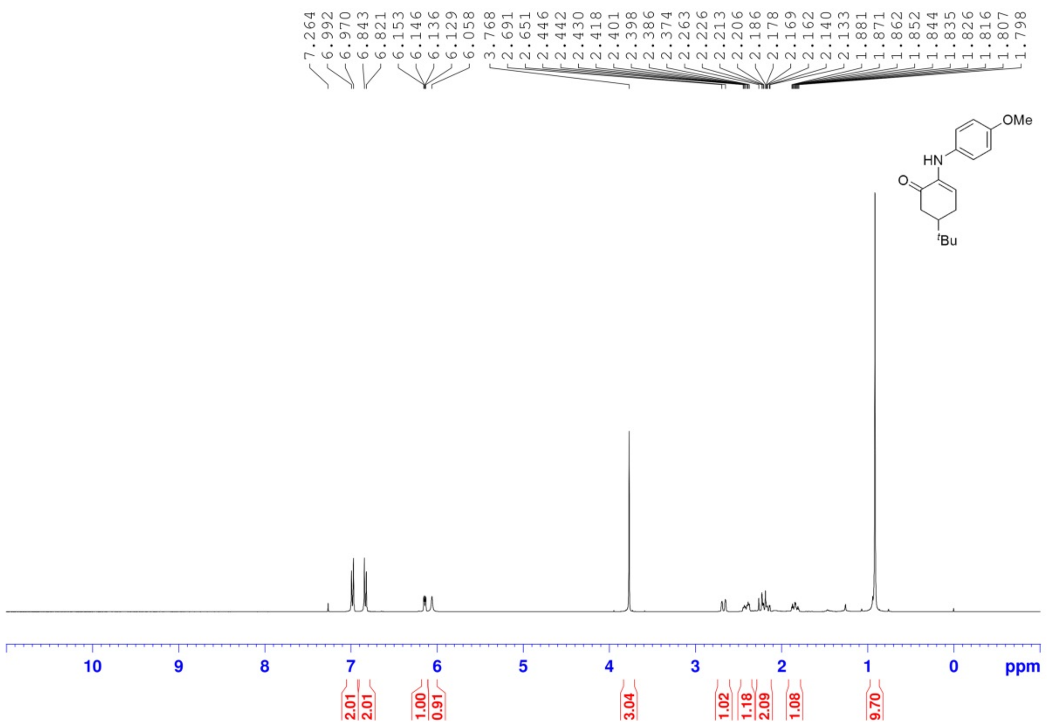

Supplementary Figure 35. ^{1}H NMR spectrum for 5-*tert*-butyl-2-(4-methoxyphenylamino)cyclohex-2-enone (**3h**).

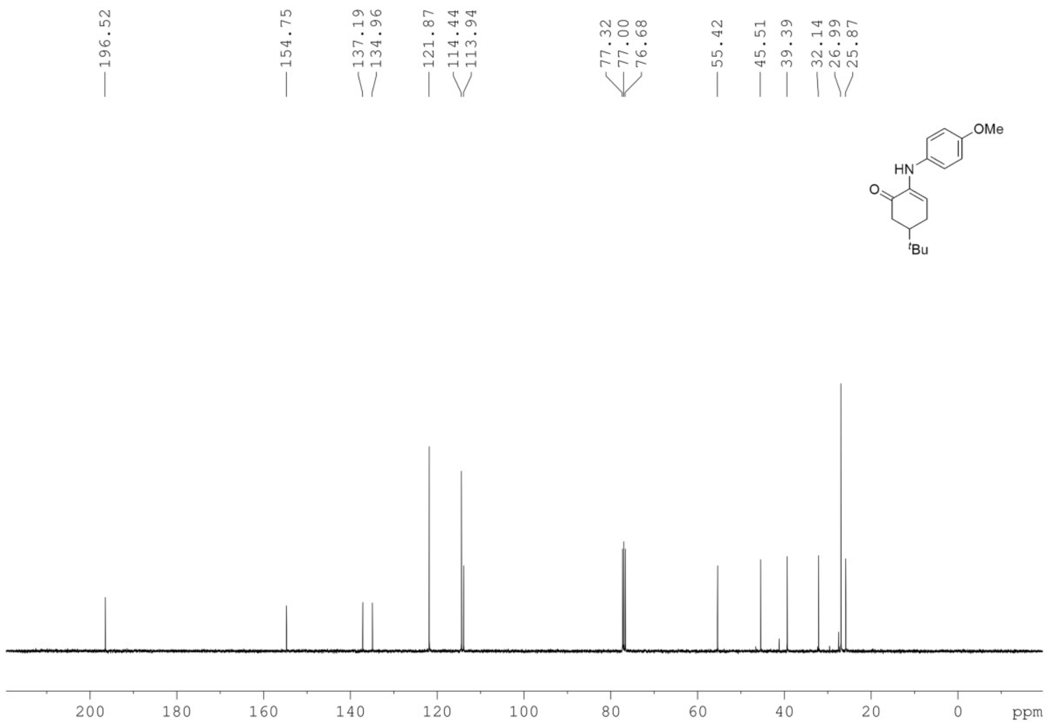

Supplementary Figure 36. ^{13}C NMR spectrum for 5-*tert*-butyl-2-(4-methoxyphenylamino)cyclohex-2-enone (**3h**).

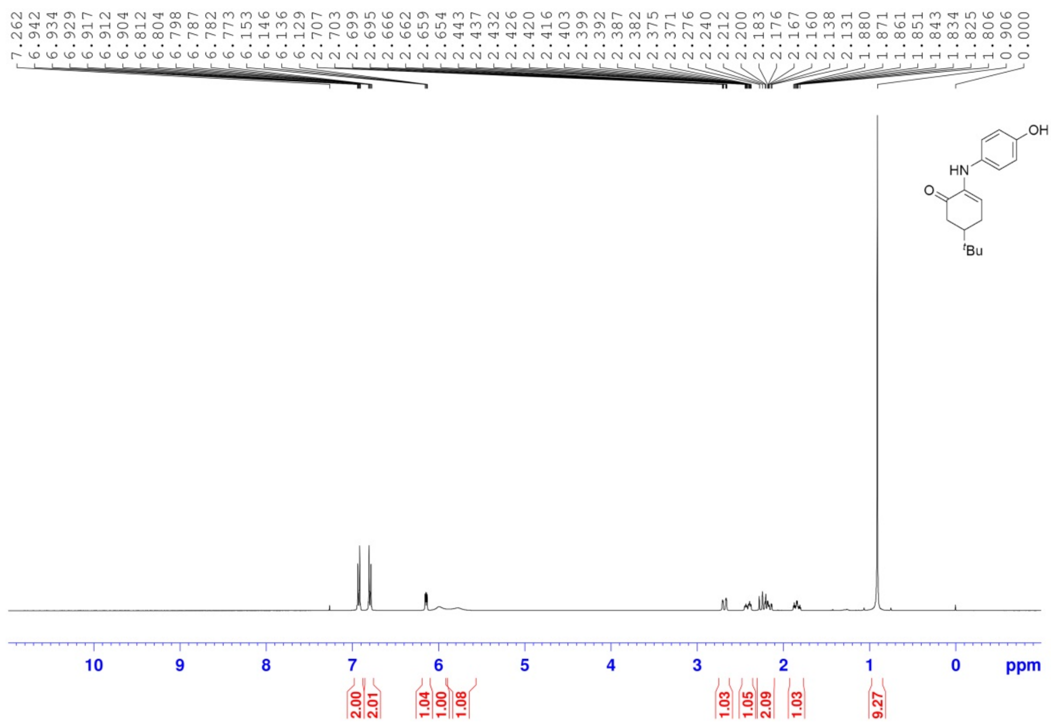

Supplementary Figure 37. ^{1}H NMR spectrum for

5-*tert*-butyl-2-(4-hydroxyphenylamino)cyclohex-2-enone (**3i**).

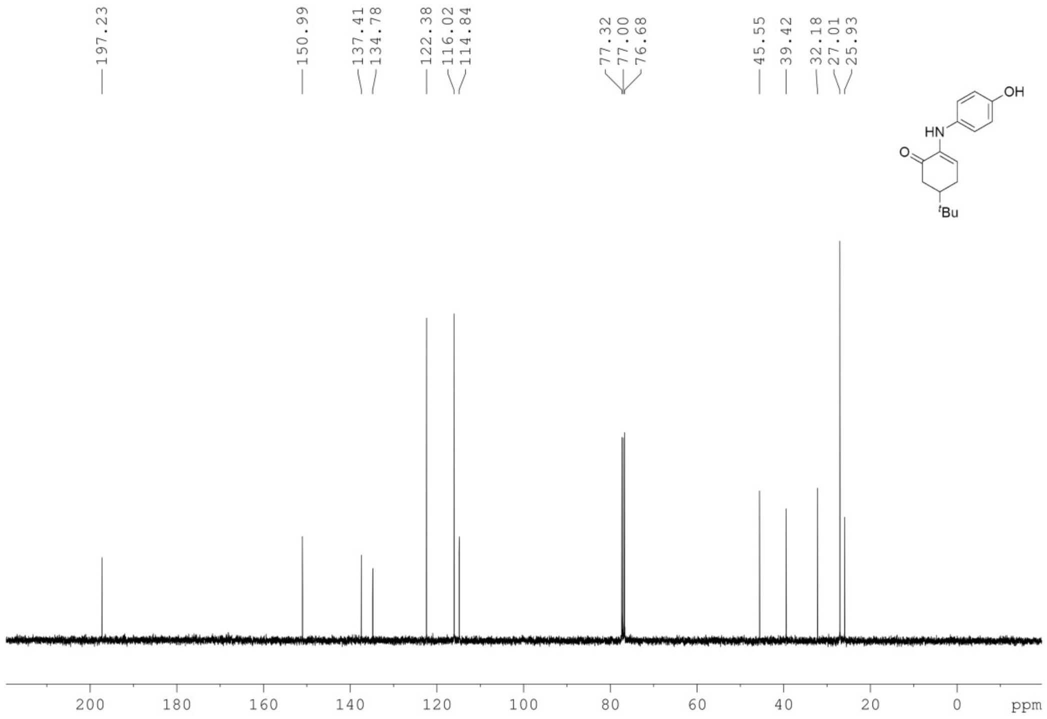

Supplementary Figure 38. ^{13}C NMR spectrum for

5-*tert*-butyl-2-(4-hydroxyphenylamino)cyclohex-2-enone (**3i**).

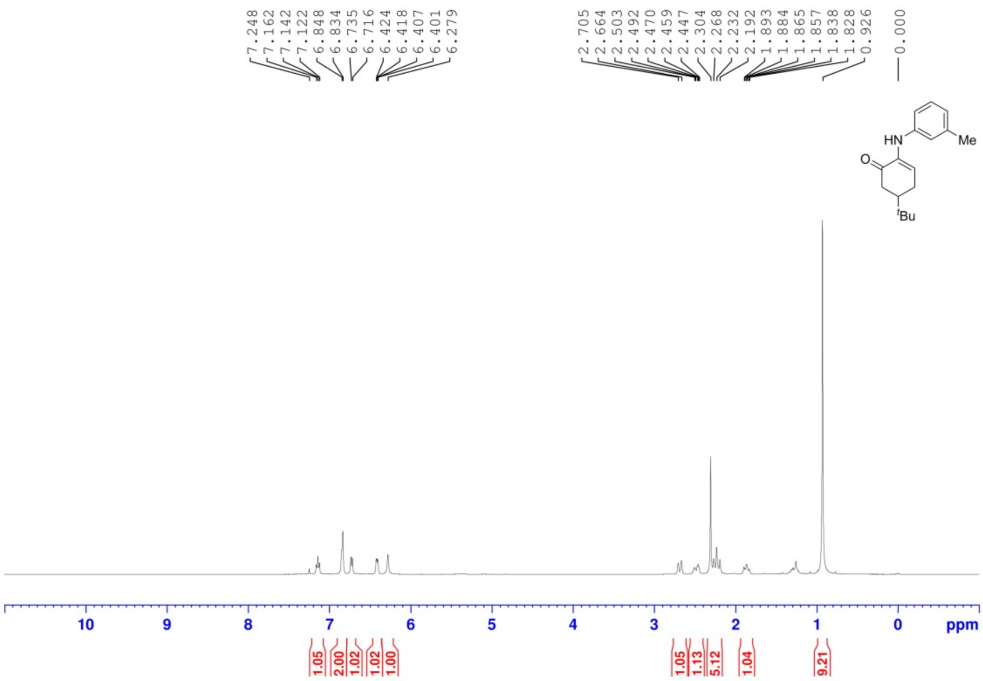

Supplementary Figure 39. ^{1}H NMR spectrum for

5-(*tert*-butyl)-2-(*m*-tolylamino)cyclohex-2-en-1-one (**3j**).

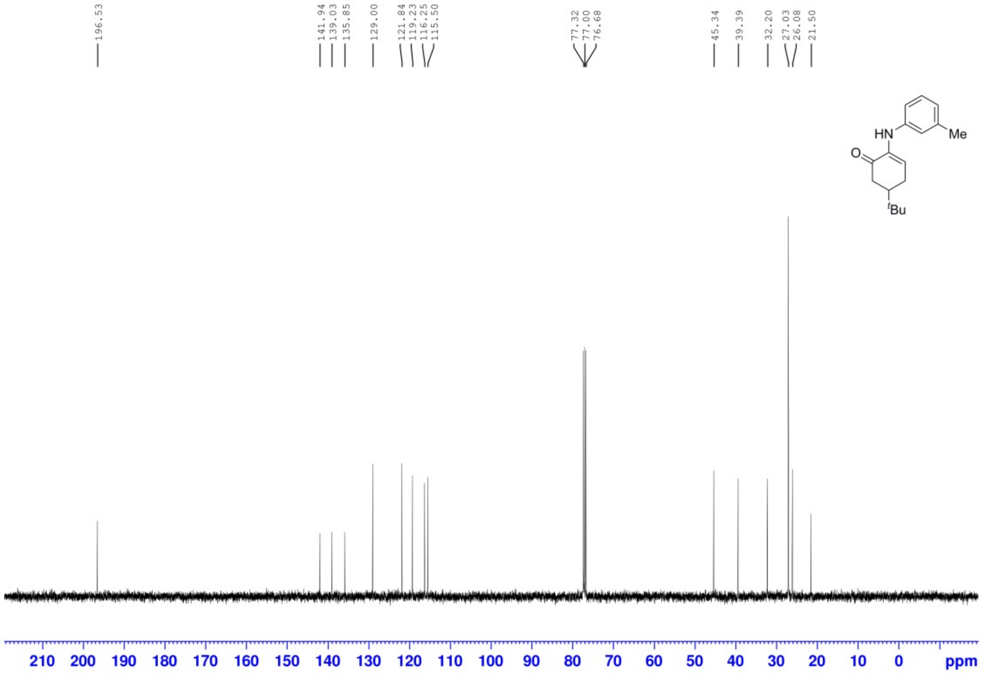

Supplementary Figure 40. ^{13}C NMR spectrum for

5-(*tert*-butyl)-2-(*m*-tolylamino)cyclohex-2-en-1-one (**3j**).

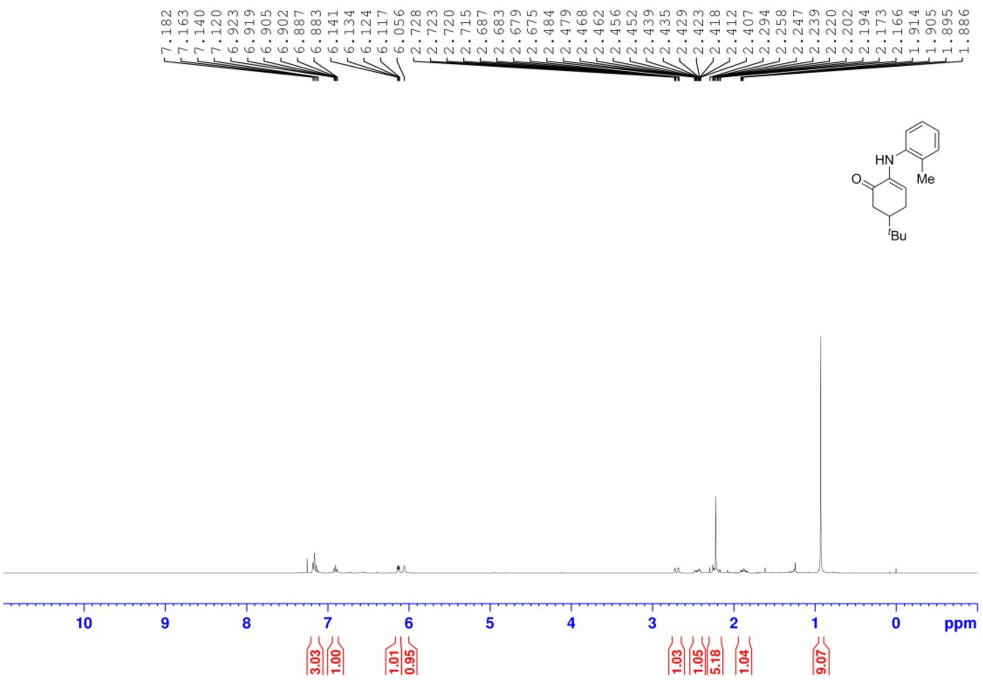

Supplementary Figure 41. ^{1}H NMR spectrum for

5-(*tert*-butyl)-2-(*o*-tolylamino)cyclohex-2-en-1-one (**3k**).

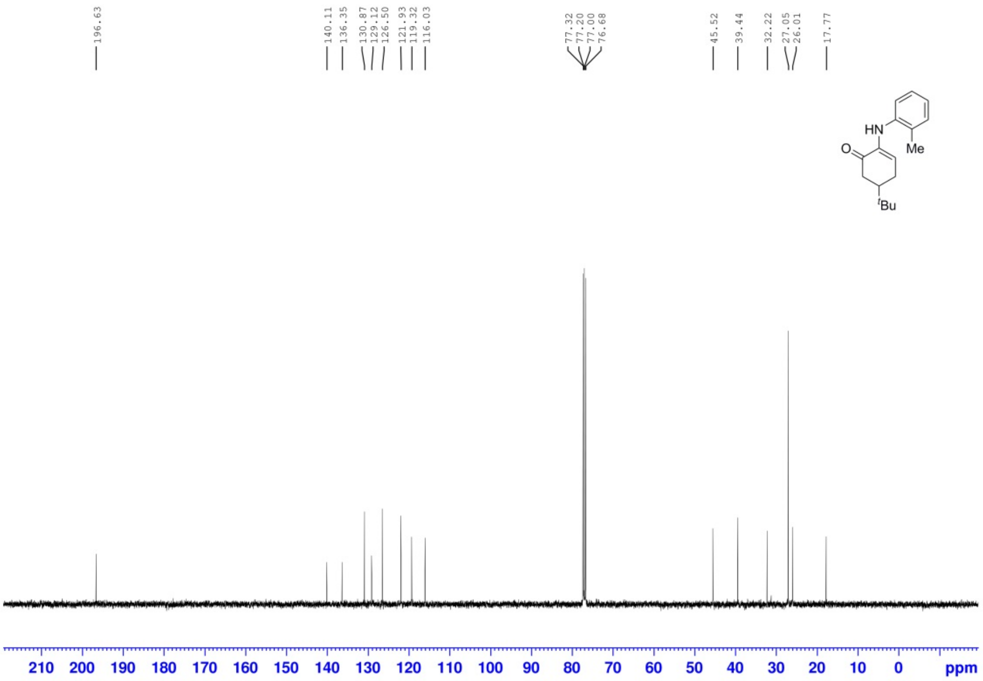

Supplementary Figure 42. ^{13}C NMR spectrum for

5-(*tert*-butyl)-2-(*o*-tolylamino)cyclohex-2-en-1-one (**3k**).

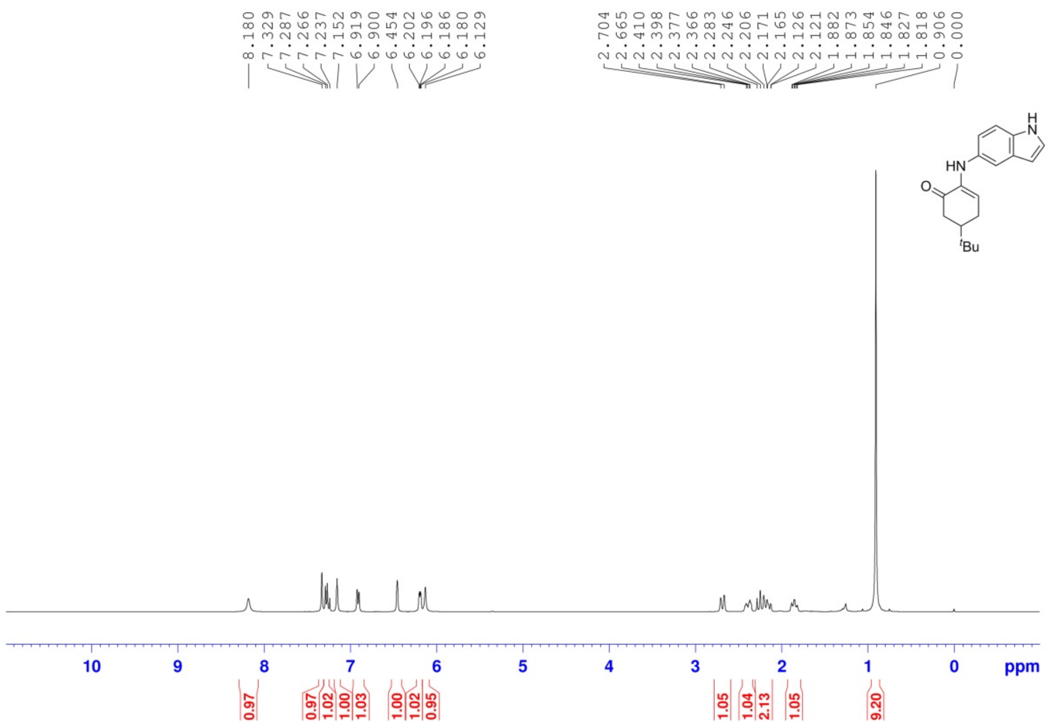

Supplementary Figure 43. ^{1}H NMR spectrum for 2-((1*H*-indol-5-yl)amino)-5-(*tert*-butyl)cyclohex-2-en-1-one (**3l**).

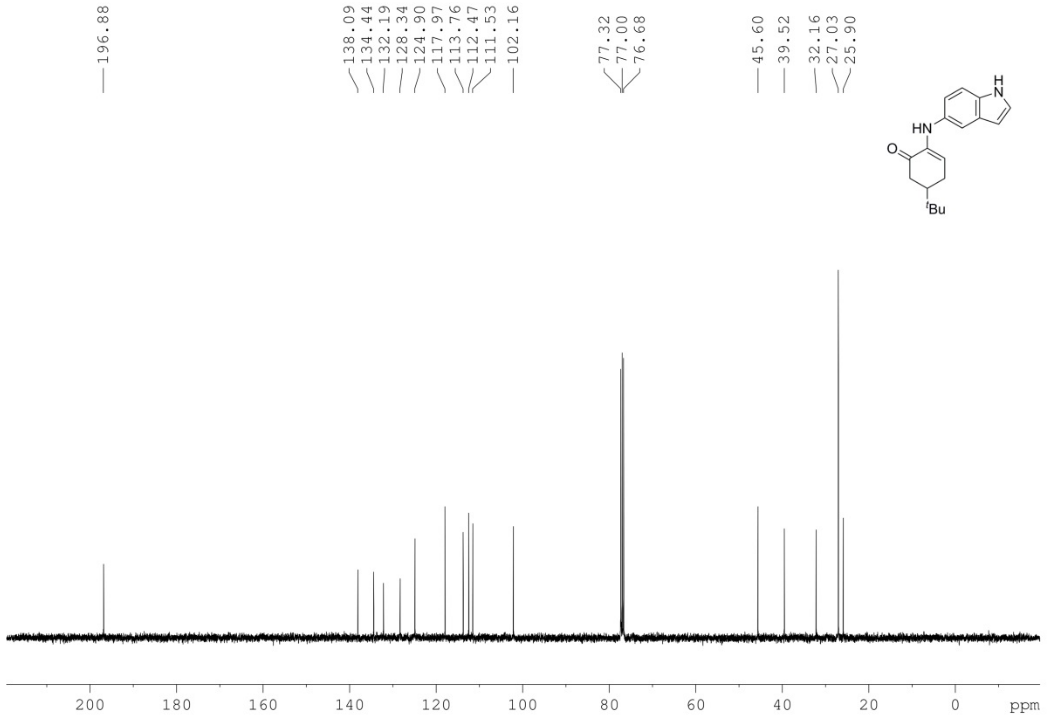

Supplementary Figure 44. ^{13}C NMR spectrum for 2-((1*H*-indol-5-yl)amino)-5-(*tert*-butyl)cyclohex-2-en-1-one (**3l**).

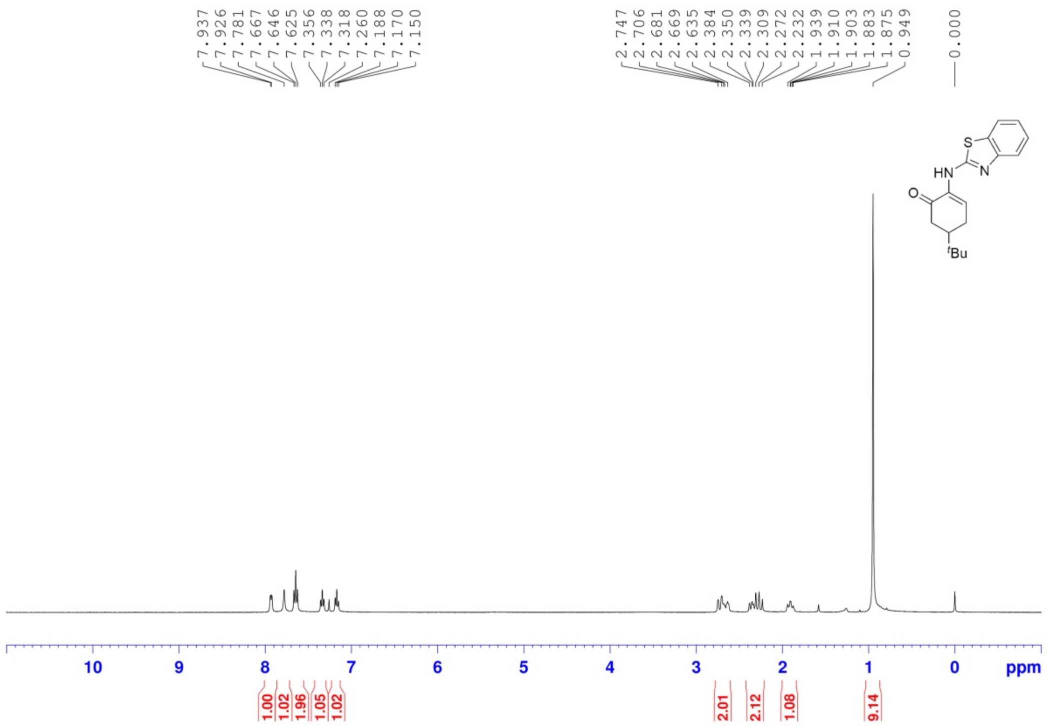

Supplementary Figure 45. ^{1}H NMR spectrum for 2-(benzo[*d*]thiazol-2-ylamino)-5-(*tert*-butyl)cyclohex-2-en-1-one (**3m**).

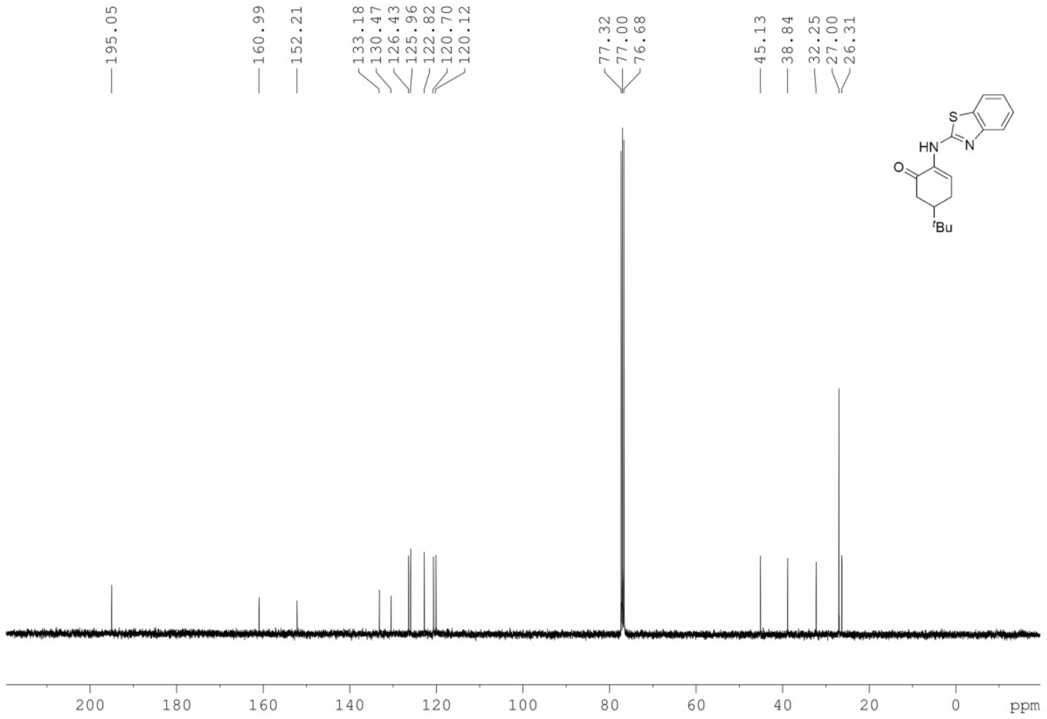

Supplementary Figure 46. ^{13}C NMR spectrum for 2-(benzo[*d*]thiazol-2-ylamino)-5-(*tert*-butyl)cyclohex-2-en-1-one (**3m**).

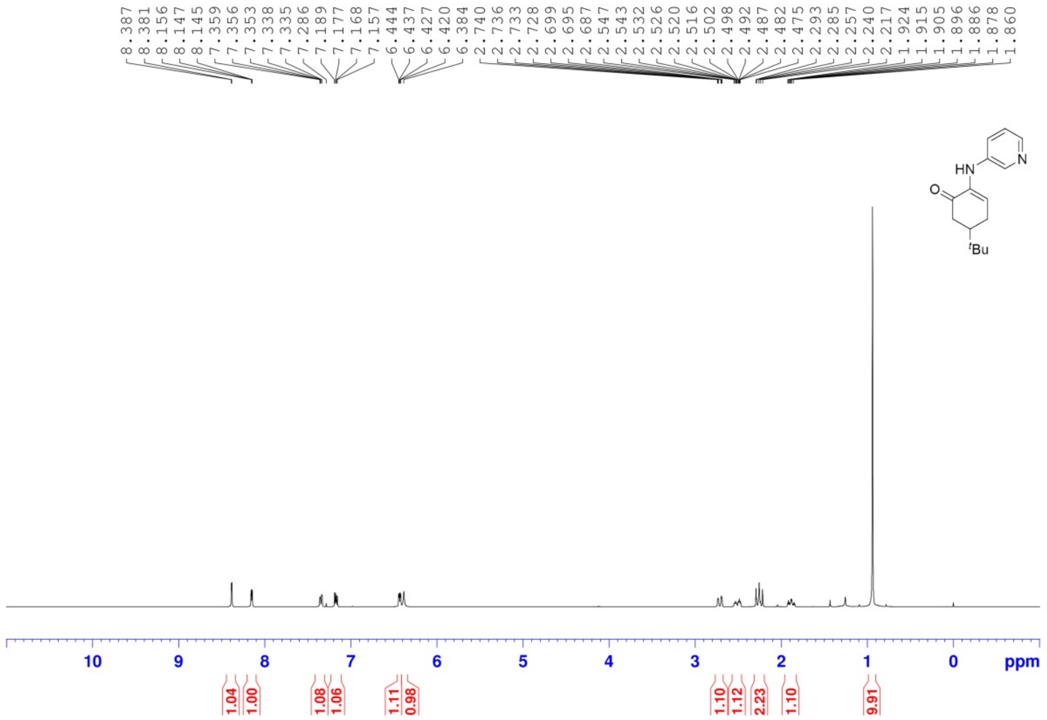

Supplementary Figure 47. ^{1}H NMR spectrum for 5-*tert*-butyl-2-(pyridin-3-ylamino)cyclohex-2-enone (**3n**).

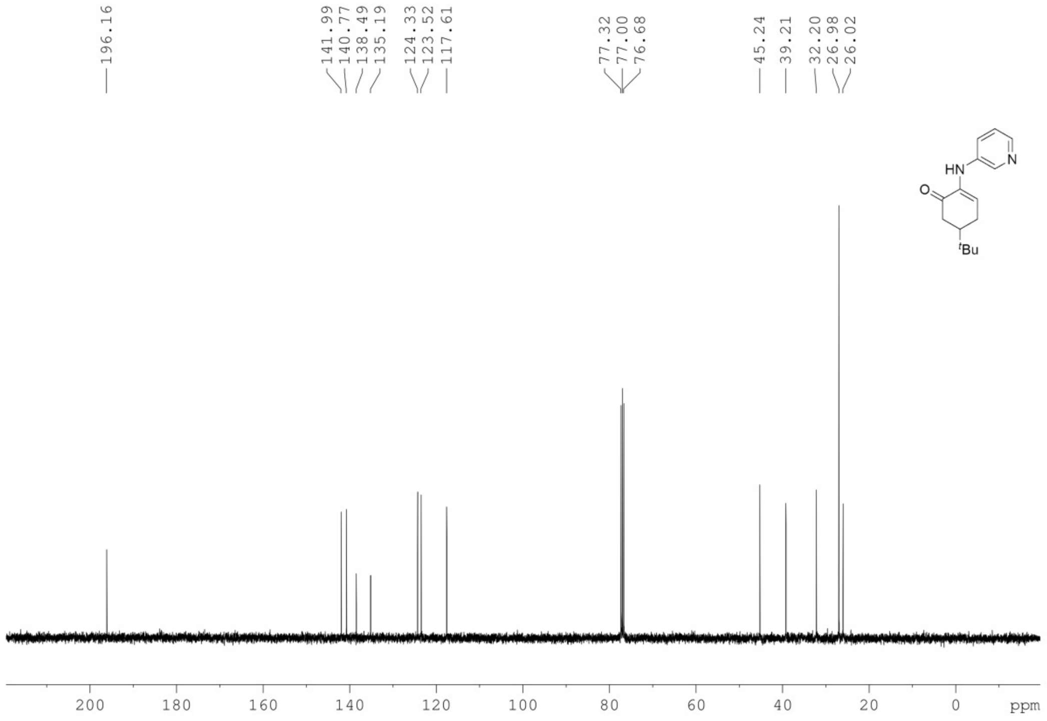

Supplementary Figure 48. ^{13}C NMR spectrum for 5-*tert*-butyl-2-(pyridin-3-ylamino)cyclohex-2-enone (**3n**).

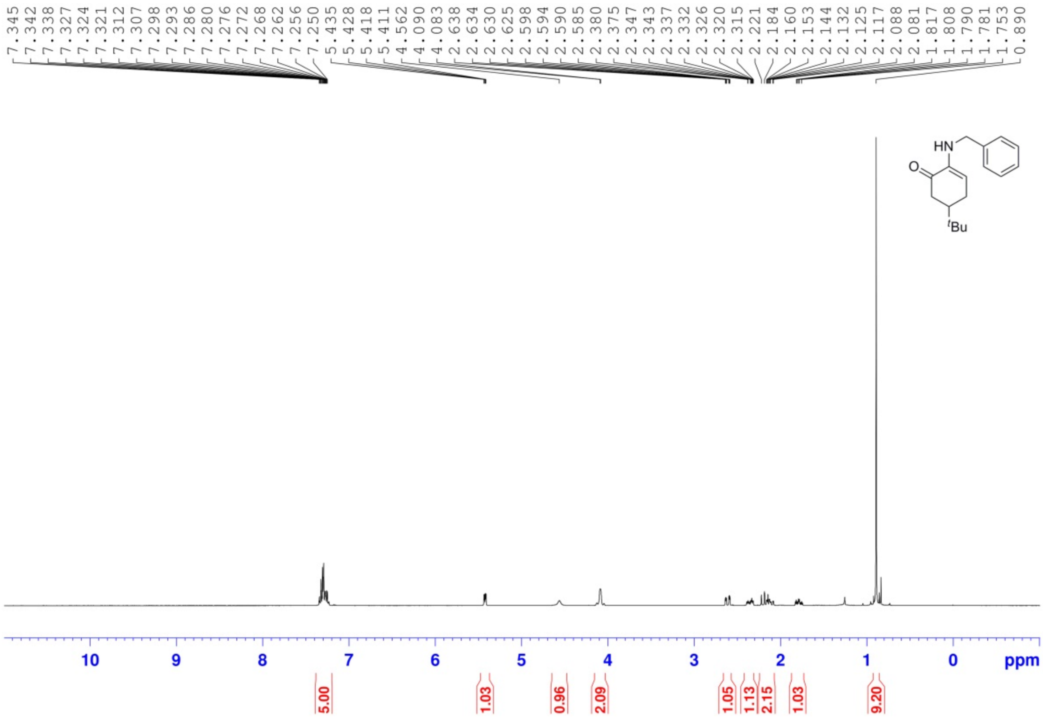

Supplementary Figure 49. ^{1}H NMR spectrum for 2-(benzylamino)-5-(*tert*-butyl)cyclohex-2-en-1-one (**3o**).

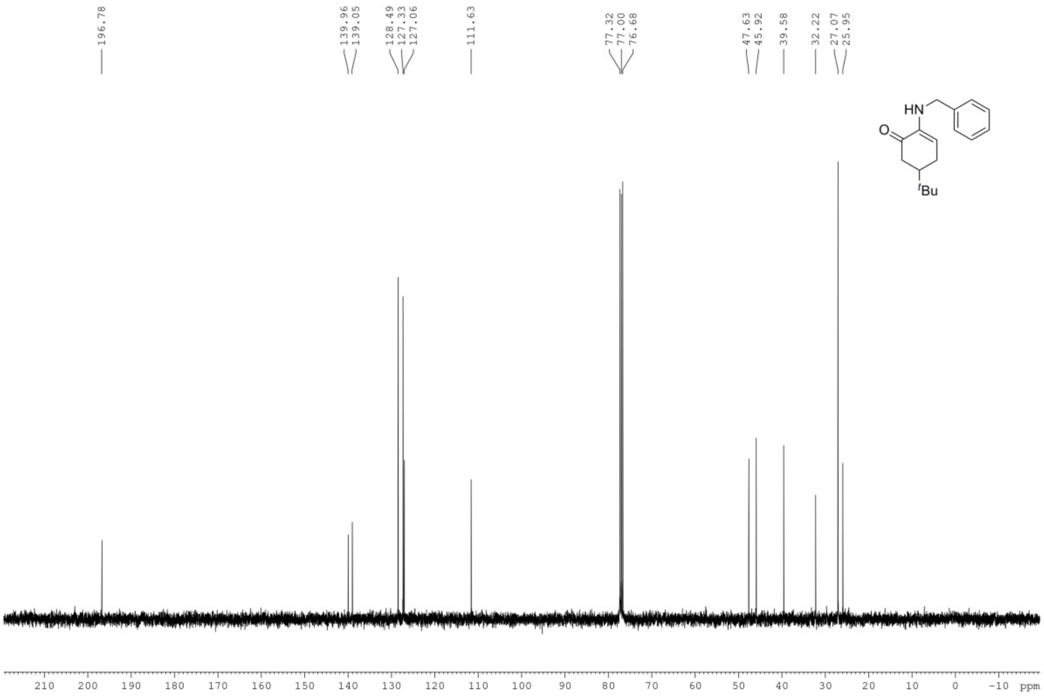

Supplementary Figure 50. ^{13}C NMR spectrum for 2-(benzylamino)-5-(*tert*-butyl)cyclohex-2-en-1-one (**3o**).

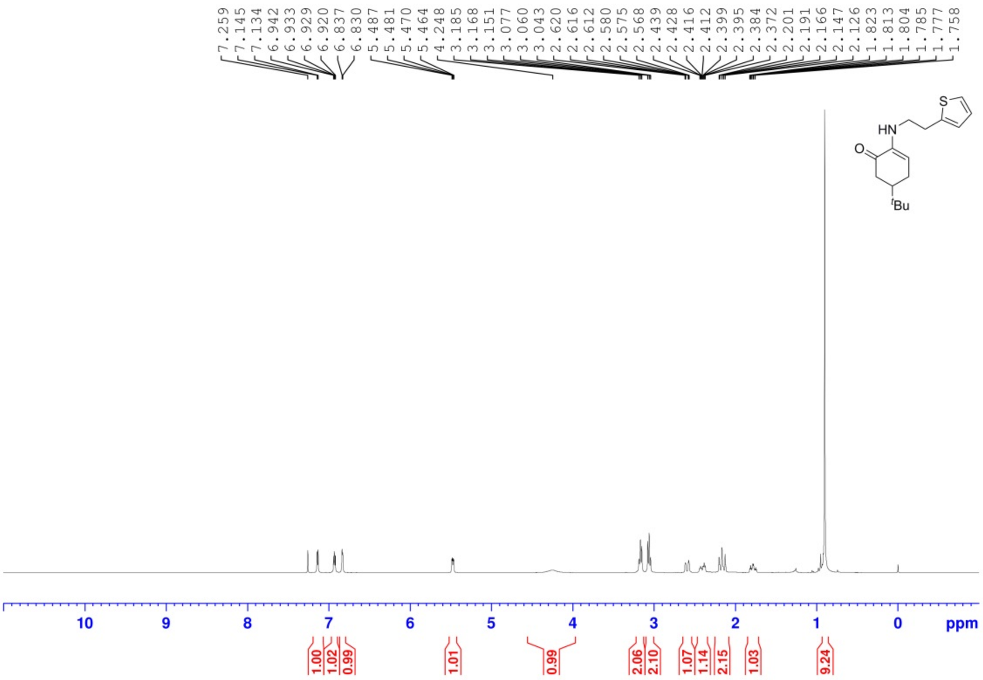

Supplementary Figure 51. ^{1}H NMR spectrum for 5-(*tert*-butyl)-2-((2-(thiophen-2-yl)ethyl)amino)cyclohex-2-en-1-one (**3p**).

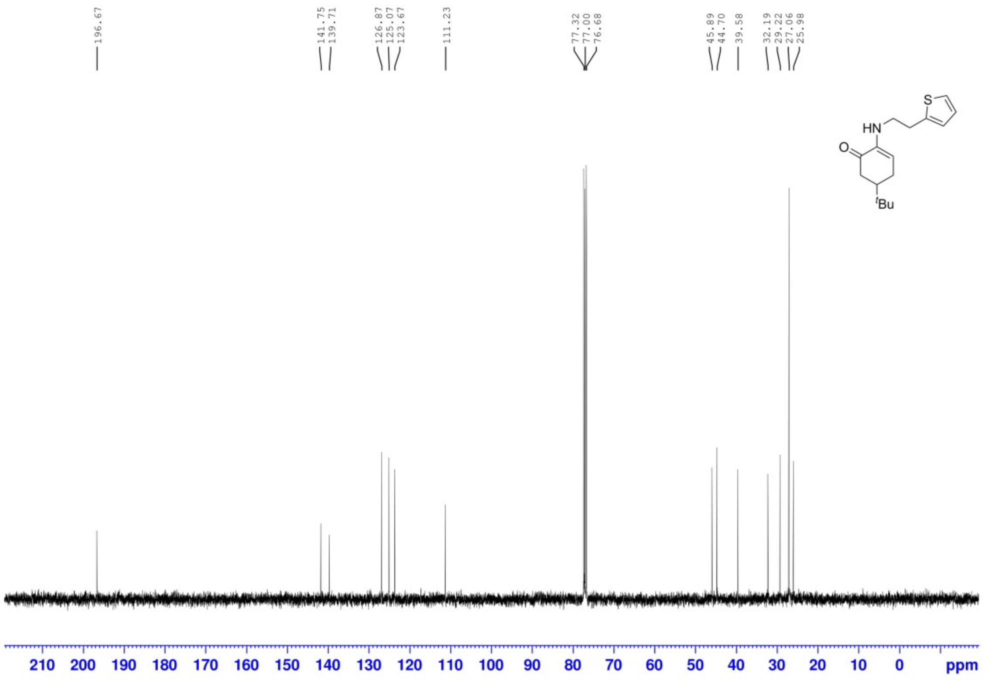

Supplementary Figure 52. ^{13}C NMR spectrum for 5-(*tert*-butyl)-2-((2-(thiophen-2-yl)ethyl)amino)cyclohex-2-en-1-one (**3p**).

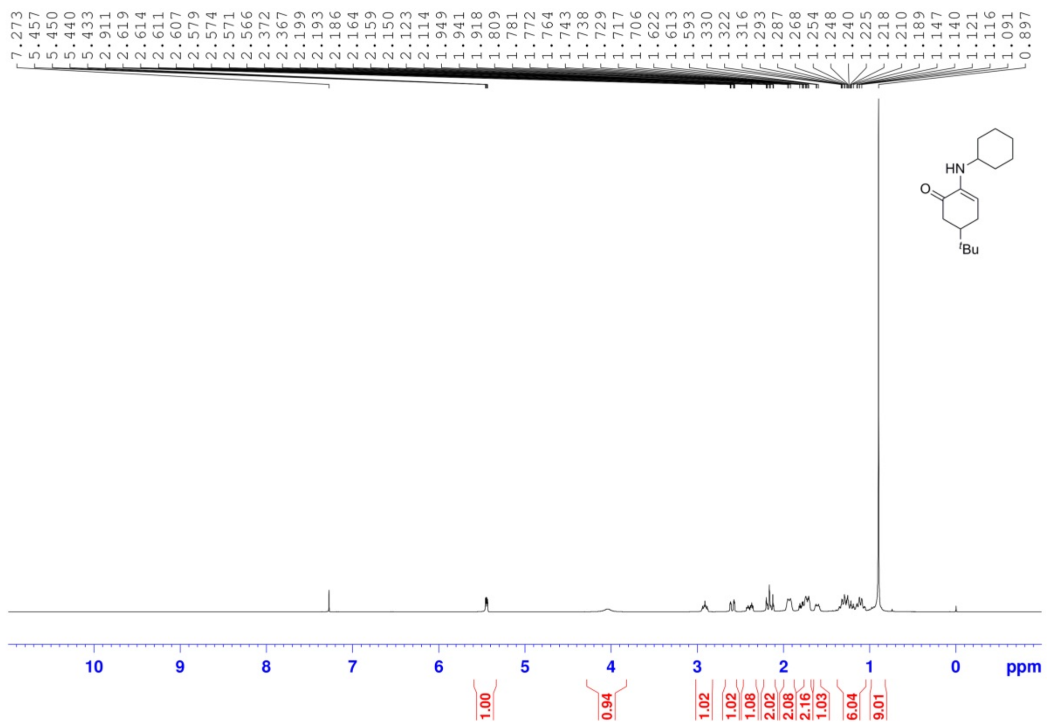

Supplementary Figure 53. ^{1}H NMR spectrum for 5-(*tert*-butyl)-2-(cyclohexylamino)cyclohex-2-enone (**3q**).

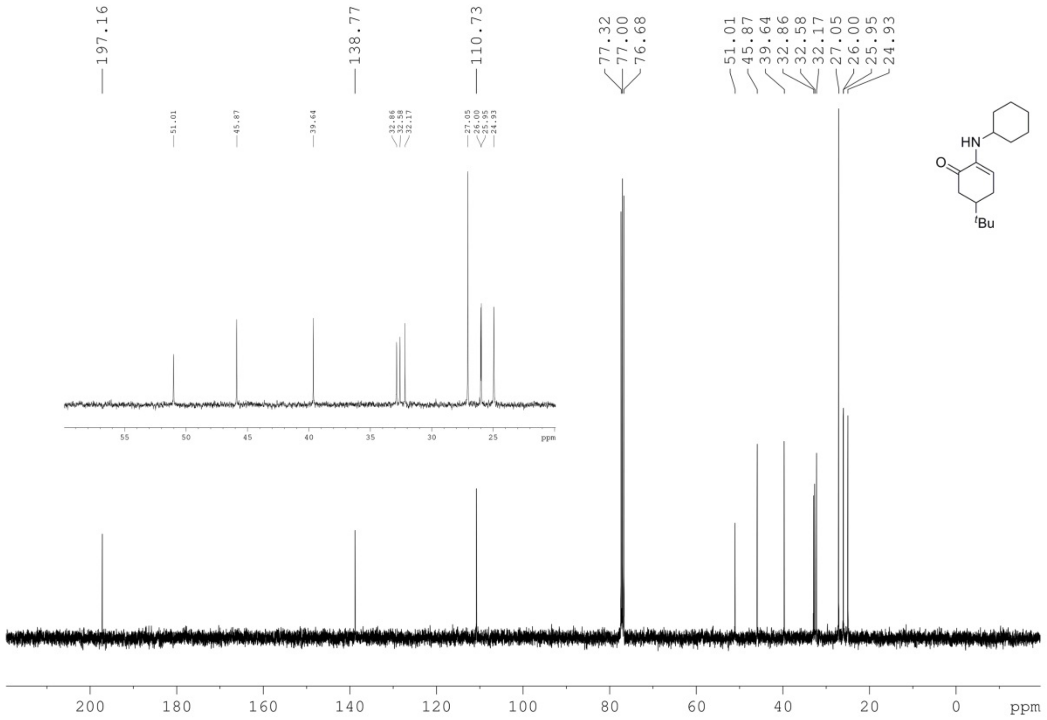

Supplementary Figure 54. ^{13}C NMR spectrum for 5-(*tert*-butyl)-2-(cyclohexylamino)cyclohex-2-enone (**3q**).

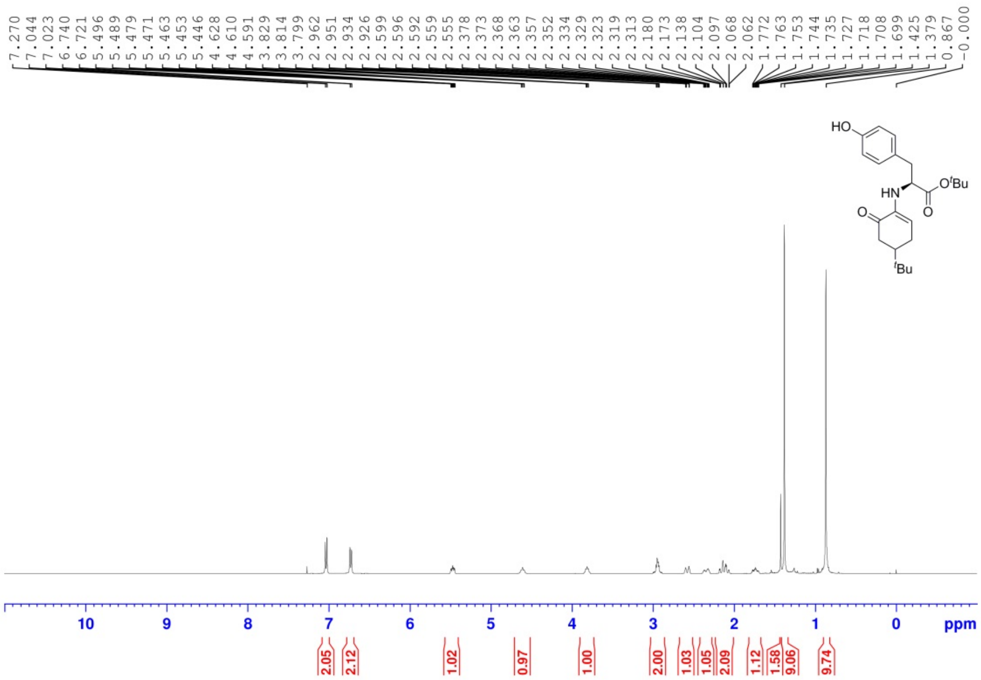

Supplementary Figure 55. ^{1}H NMR spectrum for *tert*-butyl (4-(*tert*-butyl)-6-oxocyclohex-1-en-1-yl)-*L*-tyrosinate (**3r**).

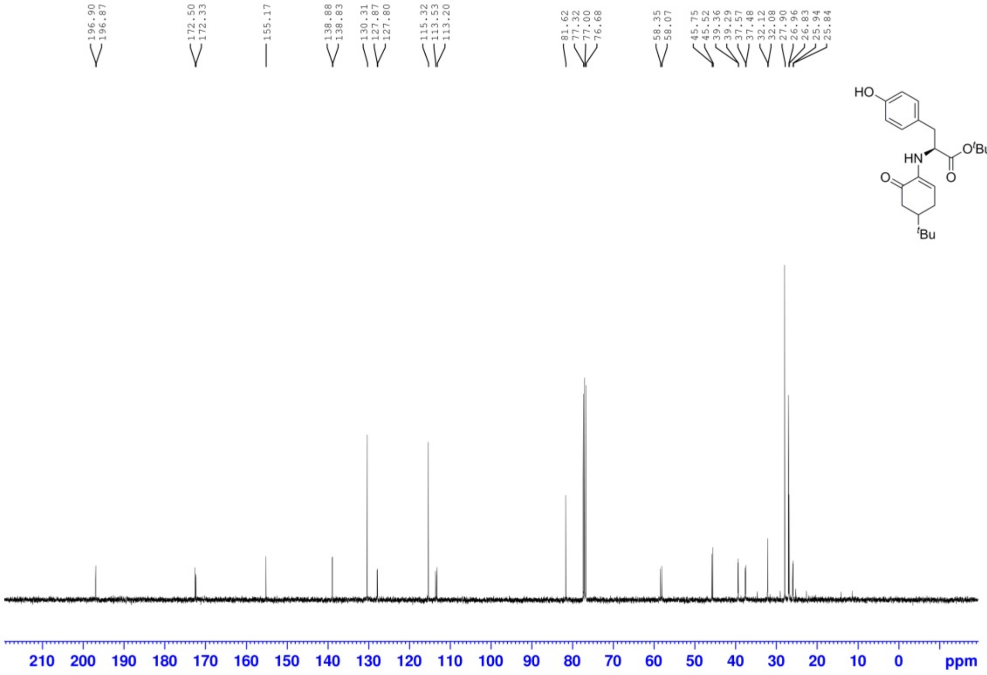

Supplementary Figure 56. ^{1}H NMR spectrum for *tert*-butyl (4-(*tert*-butyl)-6-oxocyclohex-1-en-1-yl)-*L*-tyrosinate (**3r**).

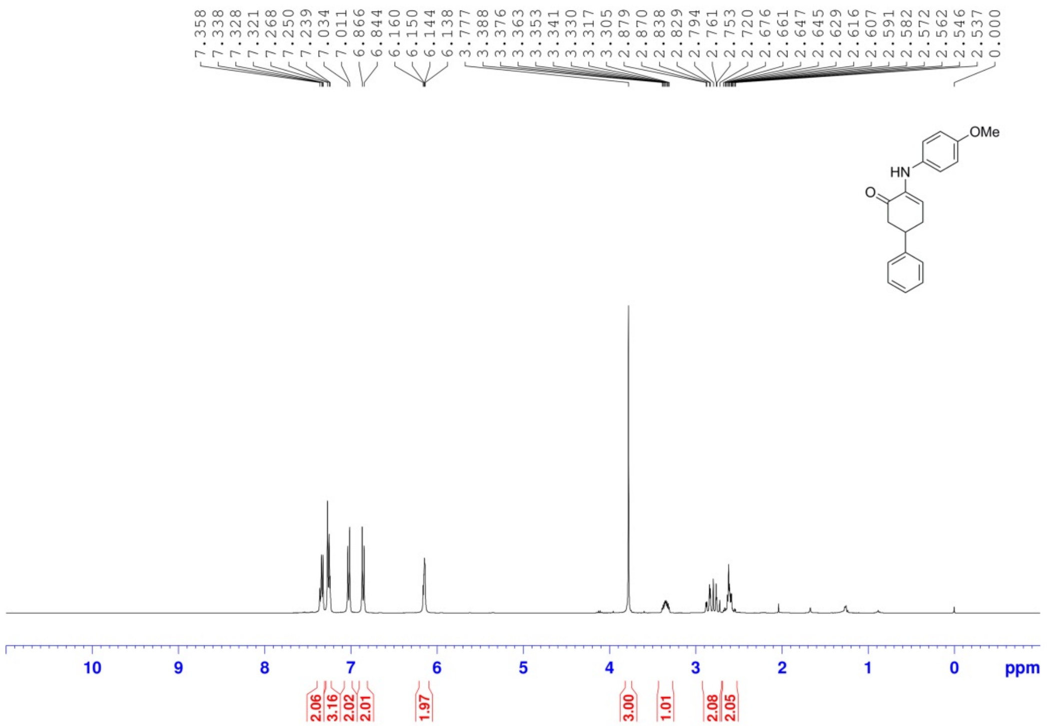

Supplementary Figure 57. ^{1}H NMR spectrum for 4-((4-methoxyphenyl)amino)-1,6-dihydro-[1,1'-biphenyl]-3(2*H*)-one (**3s**).

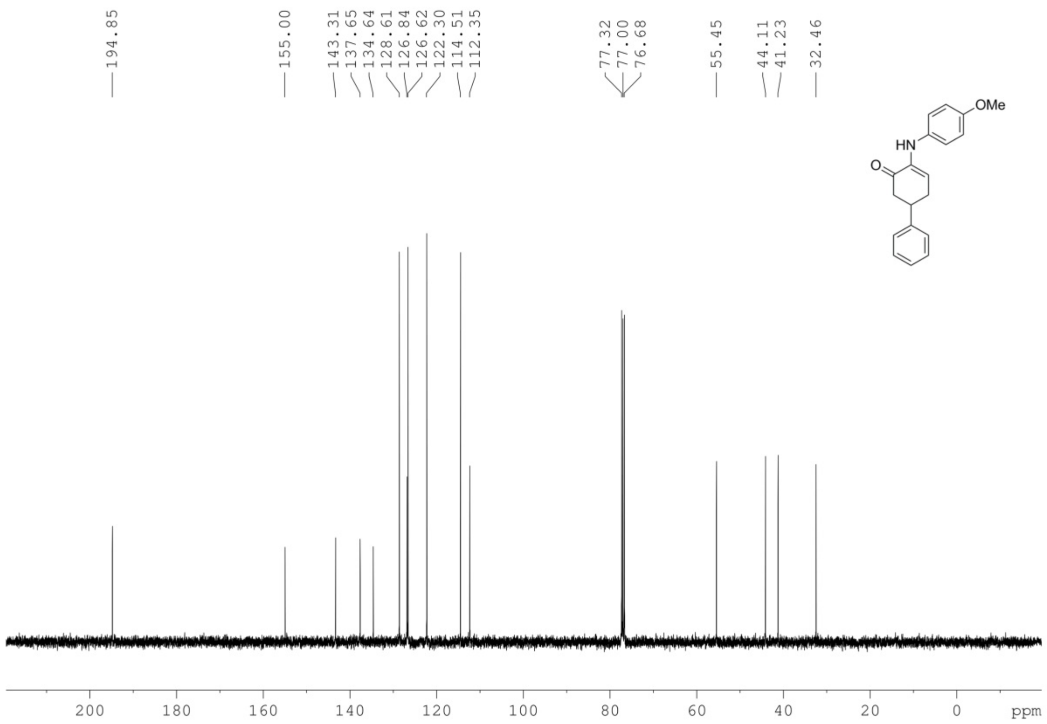

Supplementary Figure 58. ^{13}C NMR spectrum for 4-((4-methoxyphenyl)amino)-1,6-dihydro-[1,1'-biphenyl]-3(2*H*)-one (**3s**).

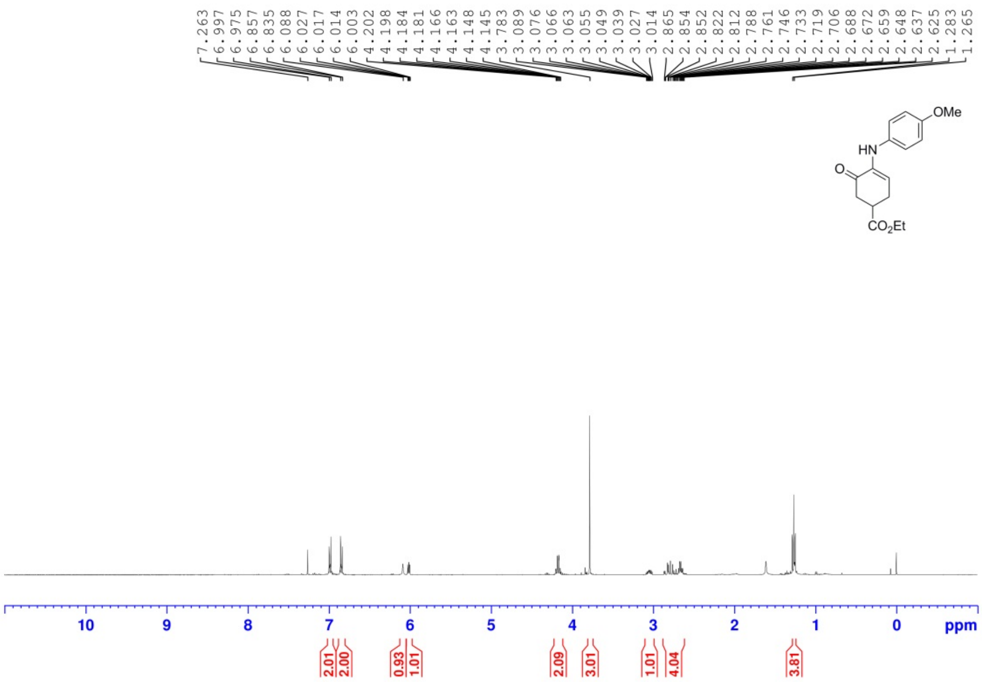

Supplementary Figure 59. ^{1}H NMR spectrum for ethyl 4-((4-methoxyphenyl)amino)-5-oxocyclohex-3-ene-1-carboxylate (**3t**).

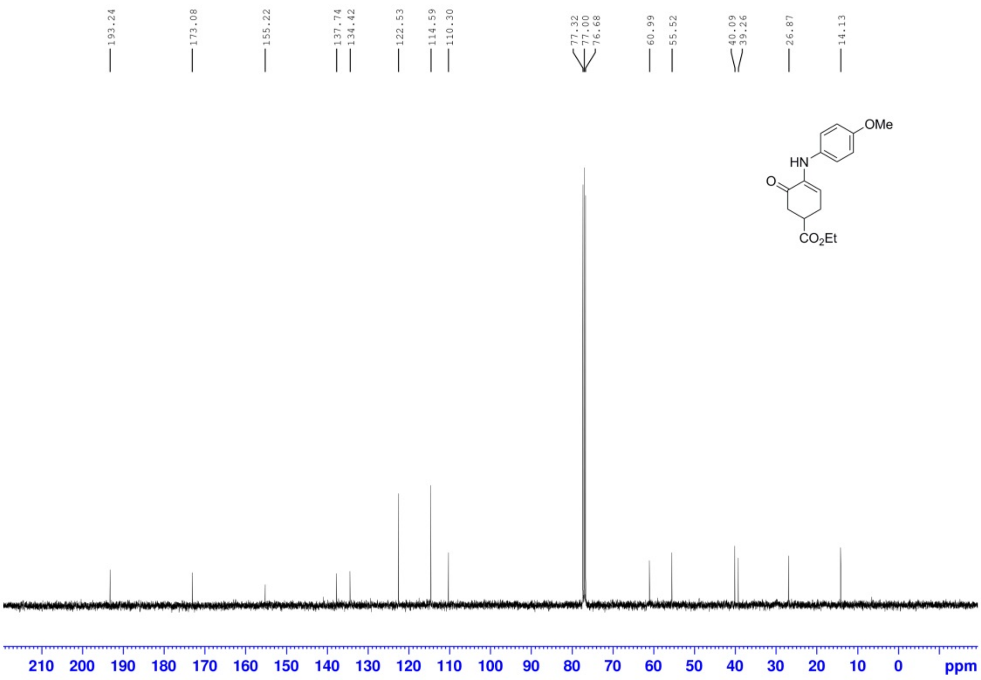

Supplementary Figure 60. ^{13}C NMR spectrum for ethyl 4-((4-methoxyphenyl)amino)-5-oxocyclohex-3-ene-1-carboxylate (**3t**).

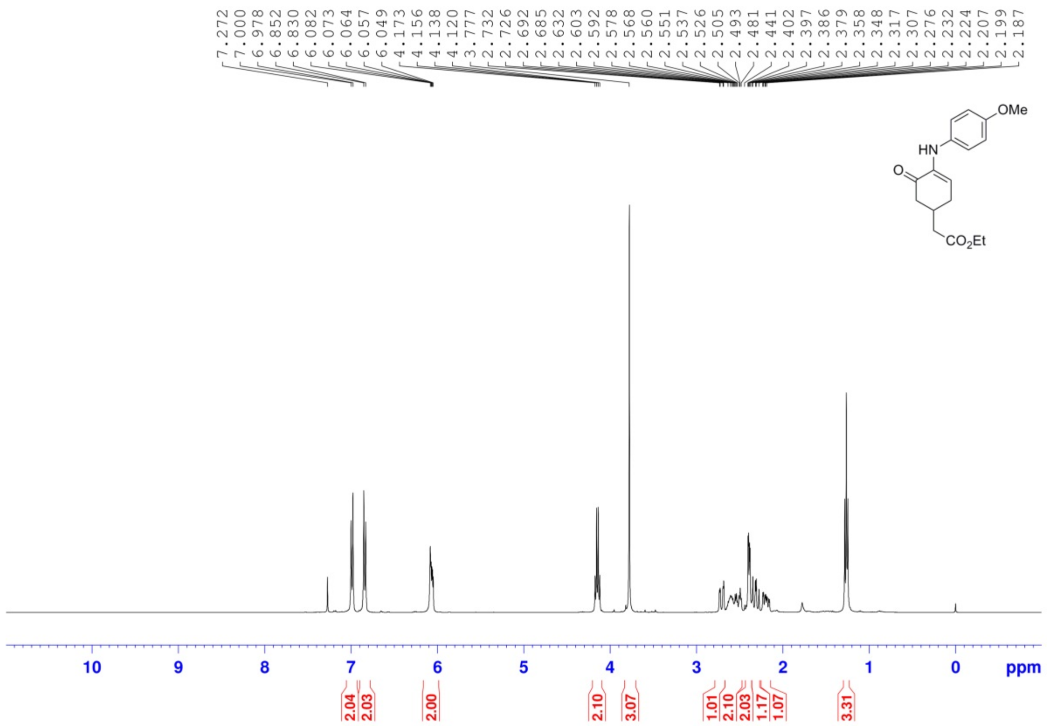

Supplementary Figure 61. ^{1}H NMR spectrum for ethyl 2-(4-((4-methoxyphenyl)amino)-5-oxocyclohex-3-en-1-yl)acetate (**3u**).

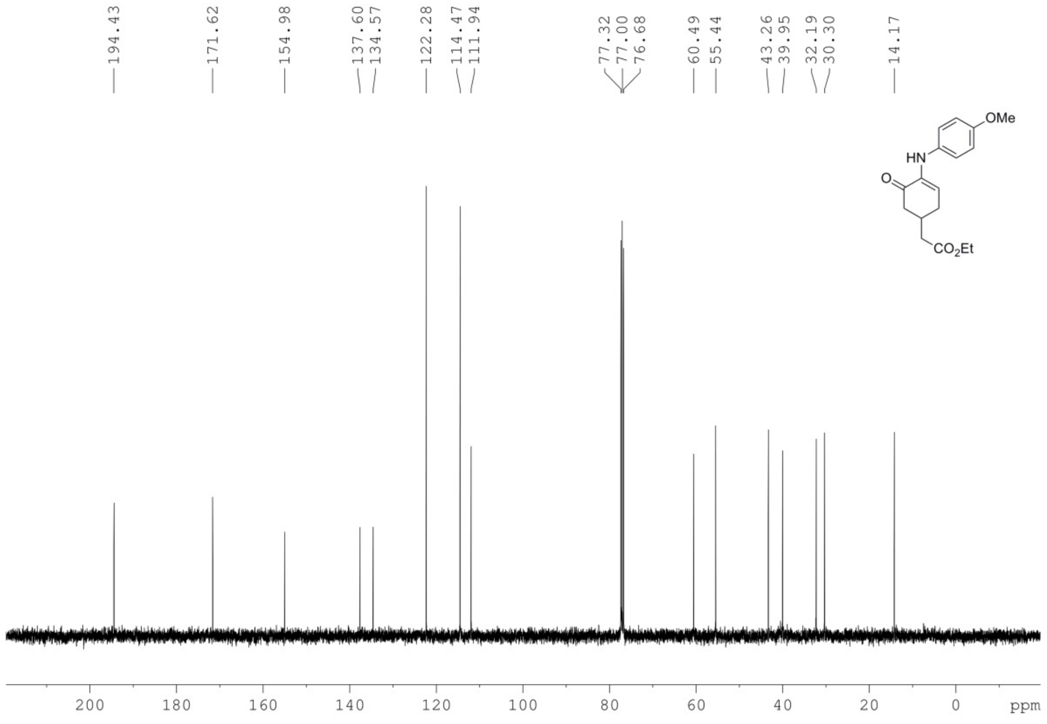

Supplementary Figure 62. ^{13}C NMR spectrum for ethyl 2-(4-((4-methoxyphenyl)amino)-5-oxocyclohex-3-en-1-yl)acetate (**3u**).

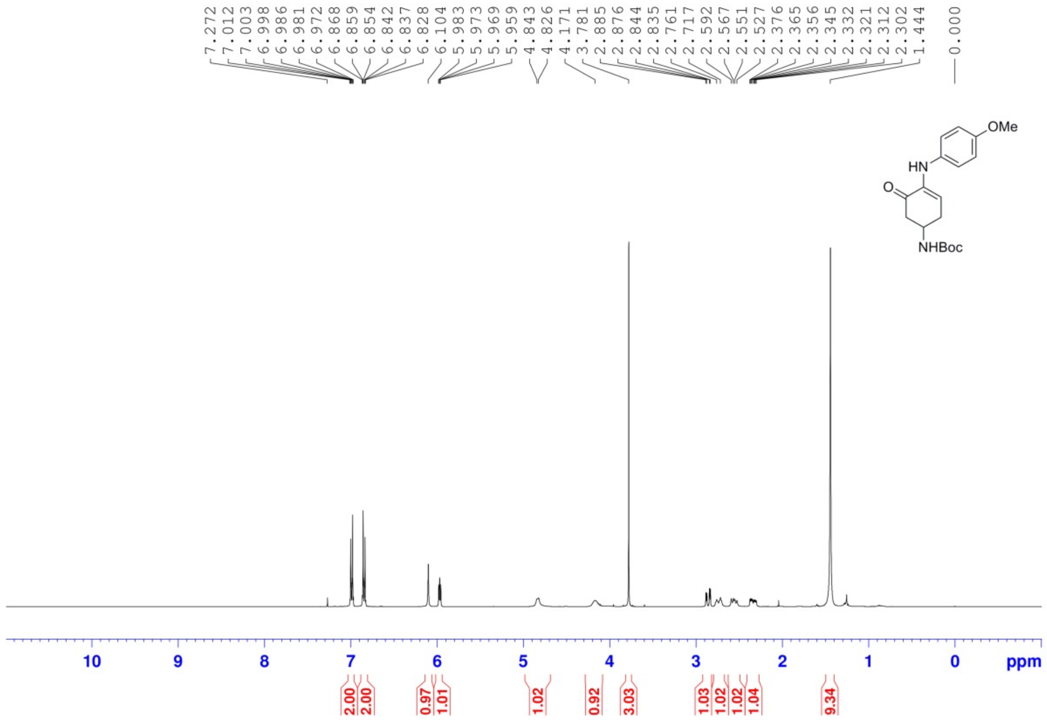

Supplementary Figure 63. ^{1}H NMR spectrum for tert-butyl (4-((4-methoxyphenyl)amino)-5-oxocyclohex-3-en-1-yl)carbamate (**3v**).

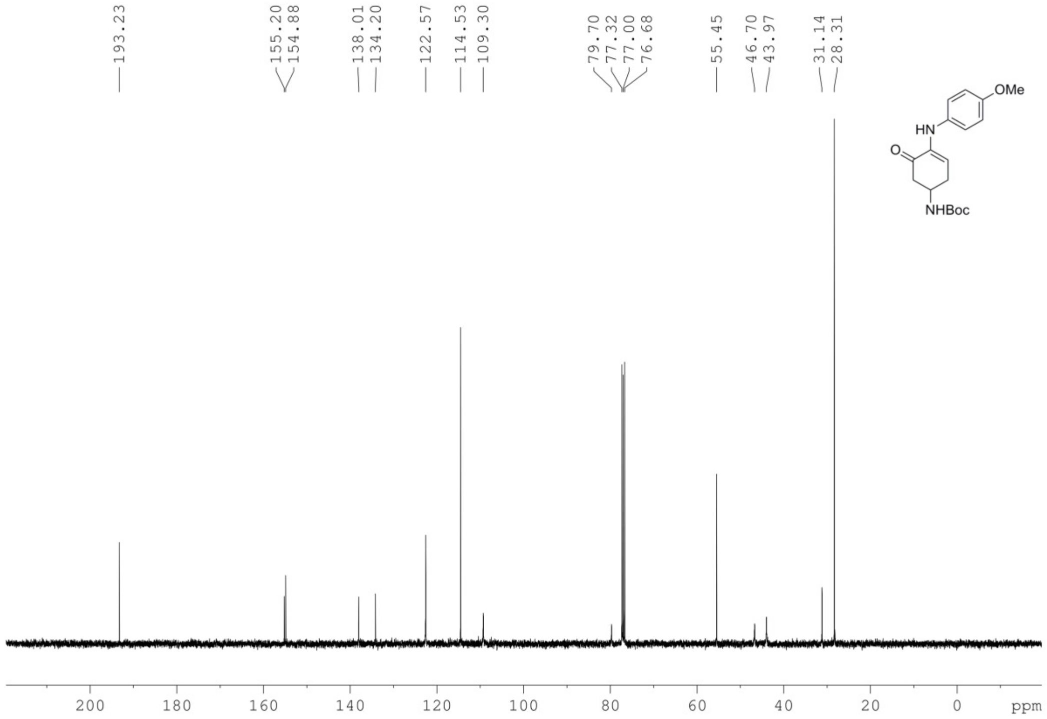

Supplementary Figure 64. ^{13}C NMR spectrum for tert-butyl (4-((4-methoxyphenyl)amino)-5-oxocyclohex-3-en-1-yl)carbamate (**3v**).

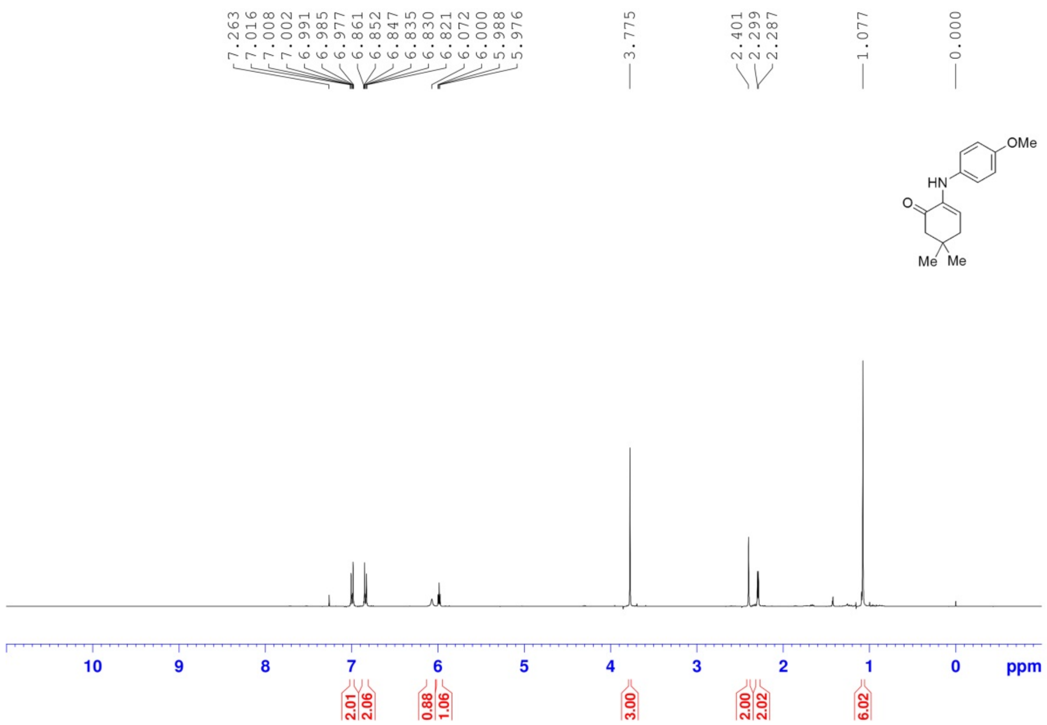

Supplementary Figure 65. ^{1}H NMR spectrum for 2-(4-methoxyphenylamino)-5,5-dimethylcyclohex-2-enone (**3w**).

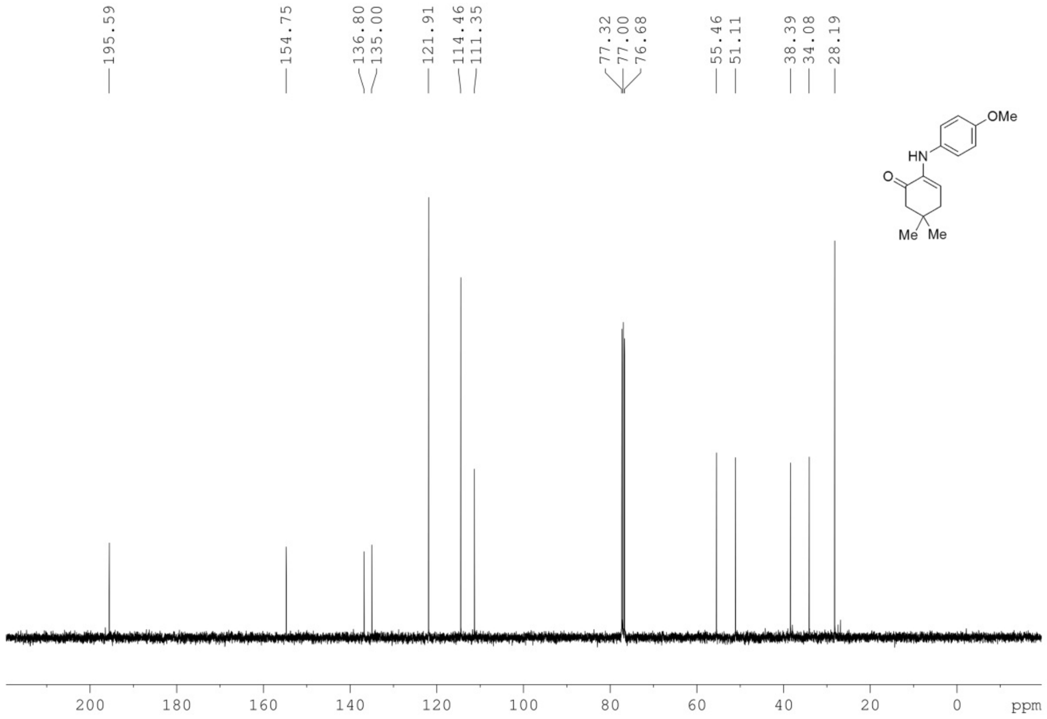

Supplementary Figure 66. ^{13}C NMR spectrum for 2-(4-methoxyphenylamino)-5,5-dimethylcyclohex-2-enone (**3w**).

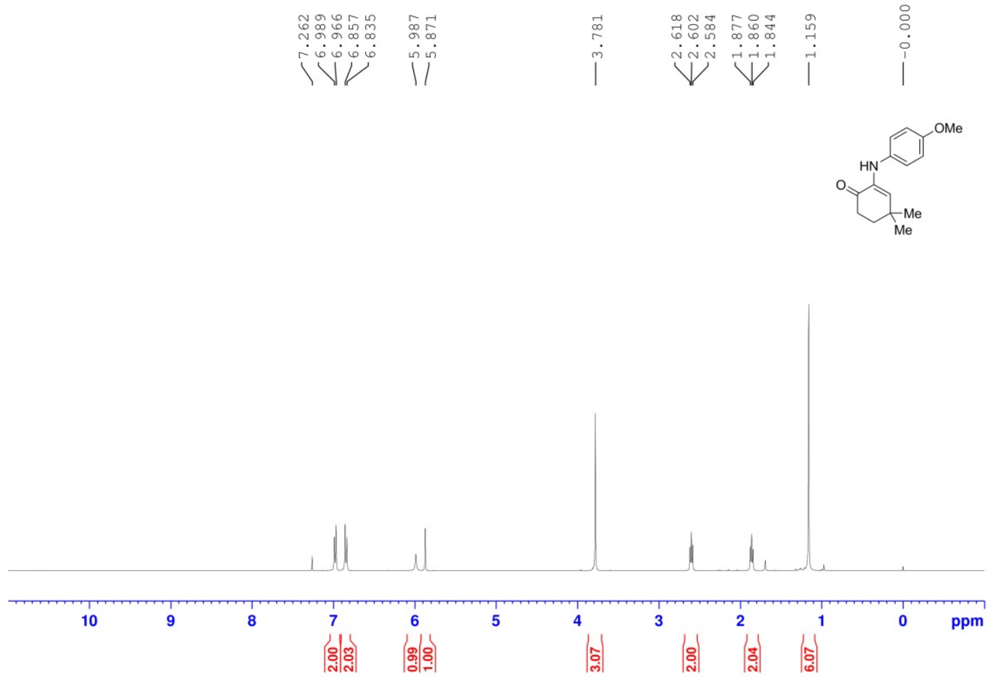

Supplementary Figure 67. ^{1}H NMR spectrum for 2-((4-methoxyphenyl)amino)-4,4-dimethylcyclohex-2-en-1-one (**3x**).

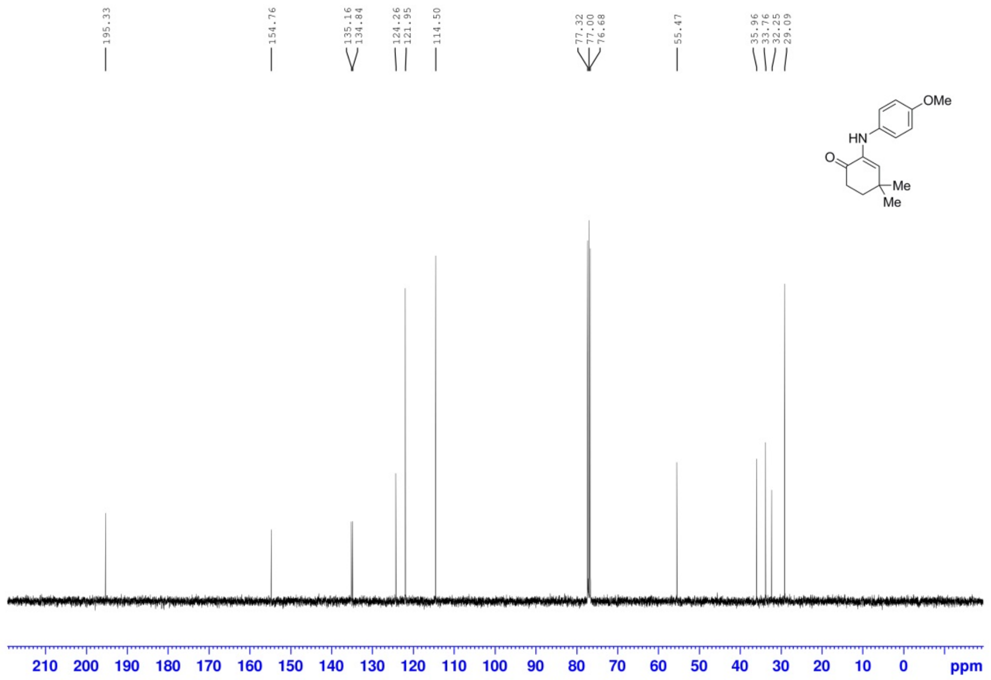

Supplementary Figure 68. ^{13}C NMR spectrum for 2-((4-methoxyphenyl)amino)-4,4-dimethylcyclohex-2-en-1-one (**3x**).

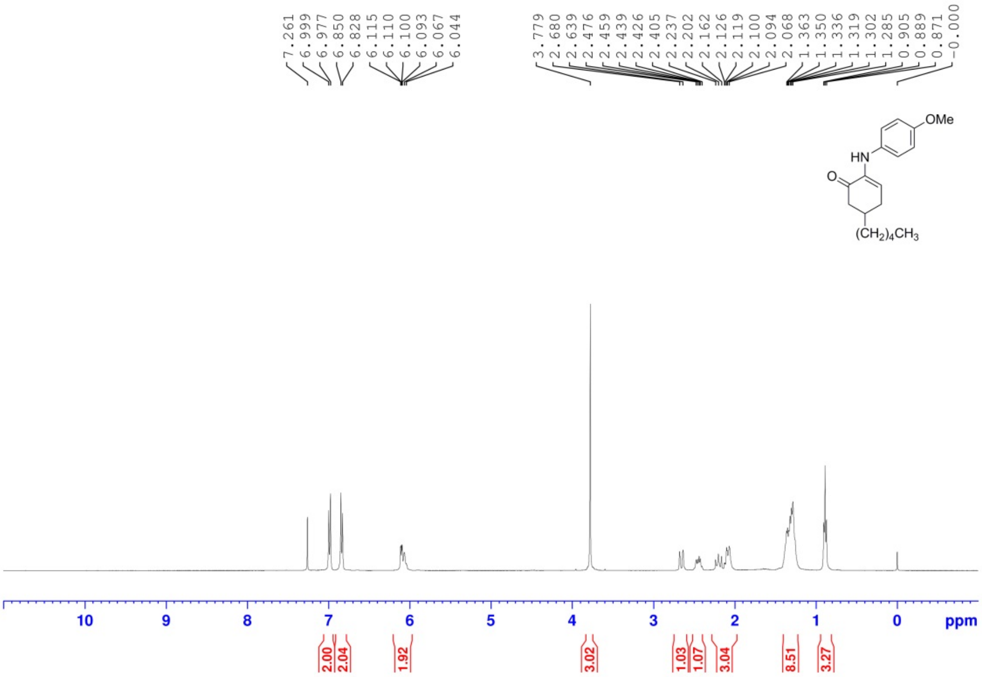

Supplementary Figure 69. ^{1}H NMR spectrum for 2-((4-methoxyphenyl)amino)-5-pentylcyclohex-2-en-1-one (**3y**).

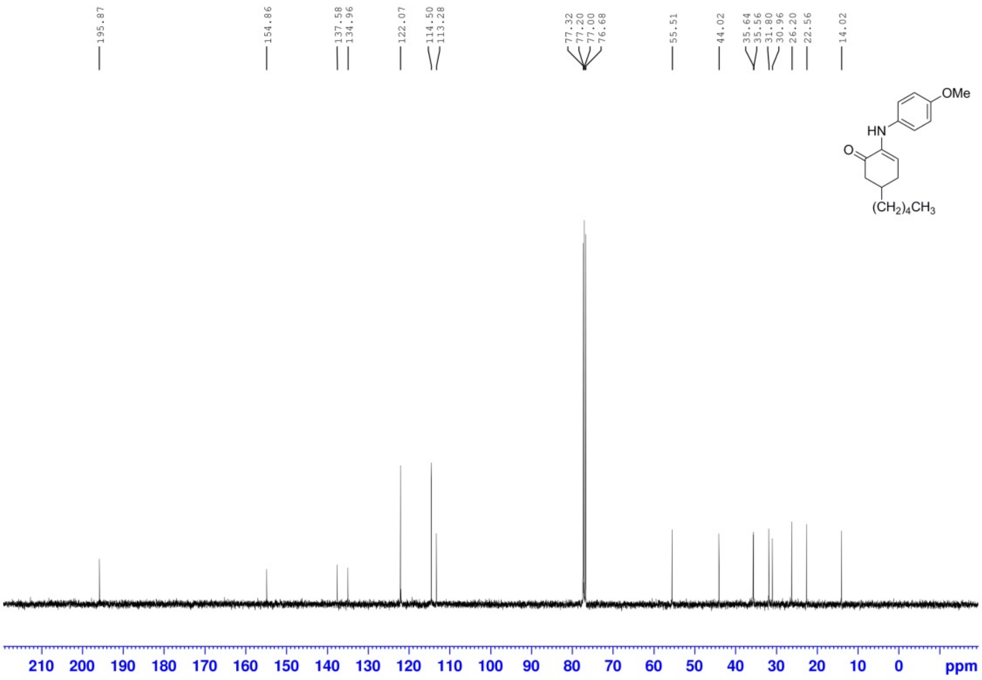

Supplementary Figure 70. ^{13}C NMR spectrum for 2-((4-methoxyphenyl)amino)-5-pentylcyclohex-2-en-1-one (**3y**).

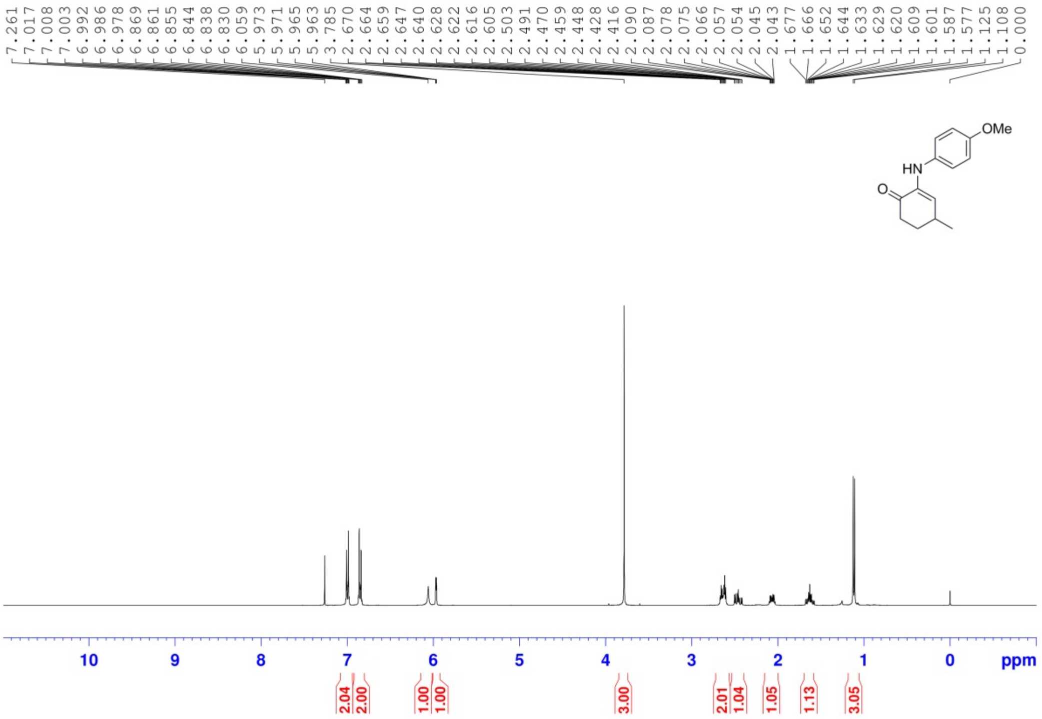

Supplementary Figure 71. ^{1}H NMR spectrum for 2-((4-methoxyphenyl)amino)-4-methylcyclohex-2-en-1-one (**3z**).

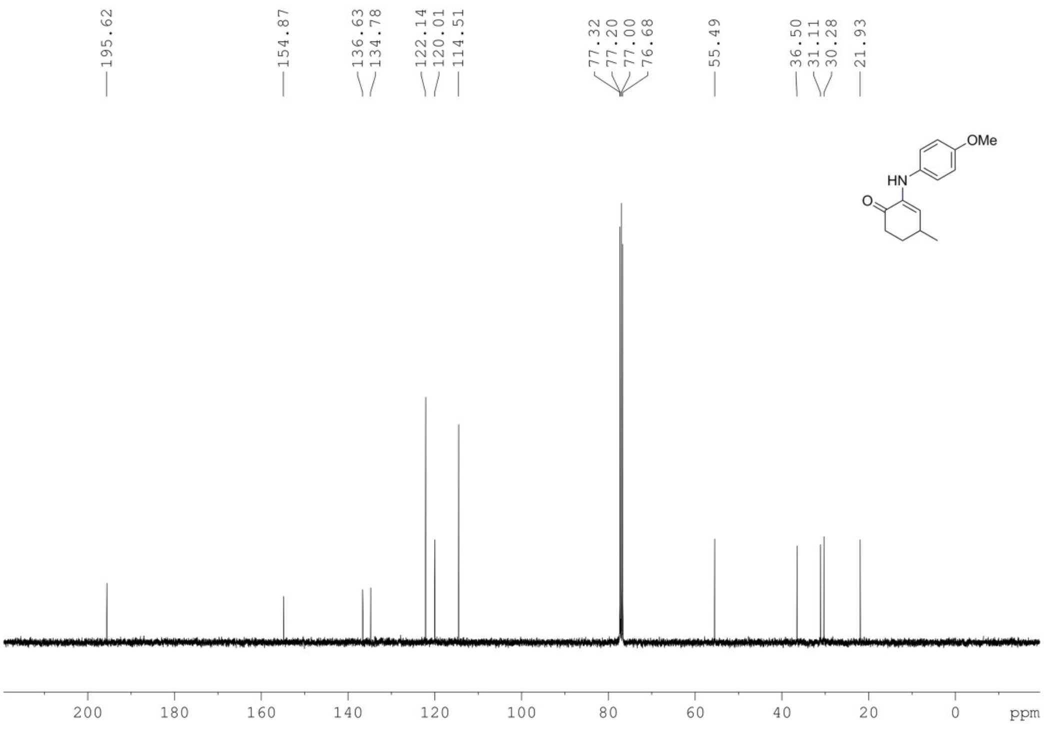

Supplementary Figure 72. ^{13}C NMR spectrum for 2-((4-methoxyphenyl)amino)-4-methylcyclohex-2-en-1-one (**3z**).

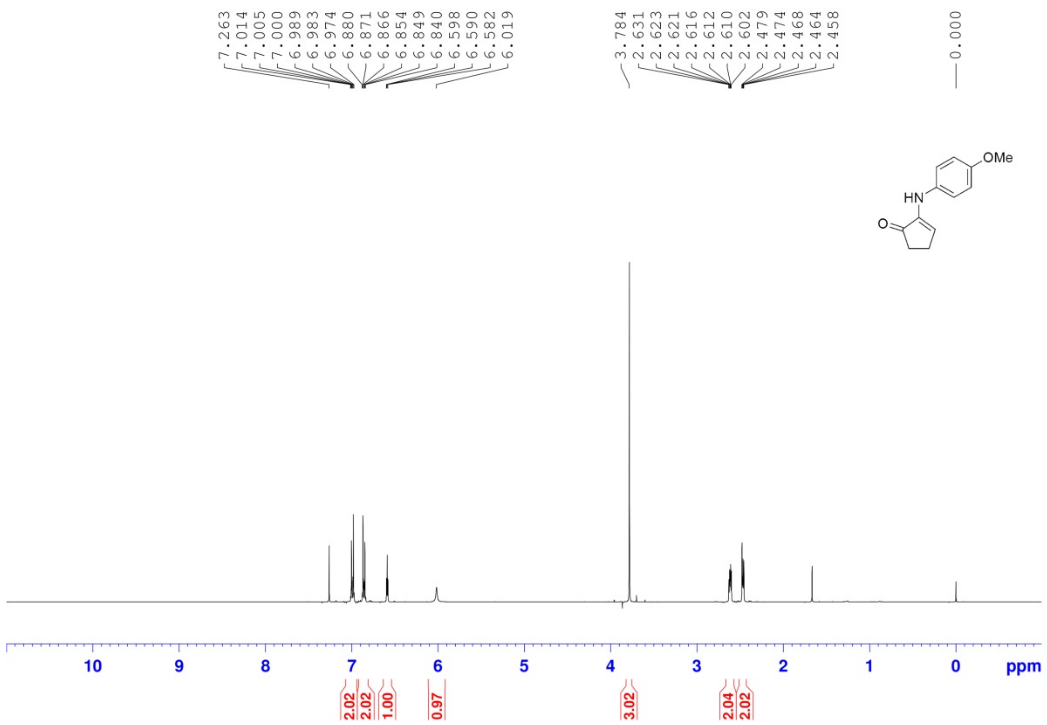

Supplementary Figure 73. ^{1}H NMR spectrum for 2-(4-methoxyphenylamino)cyclopent-2-enone (**3aa**).

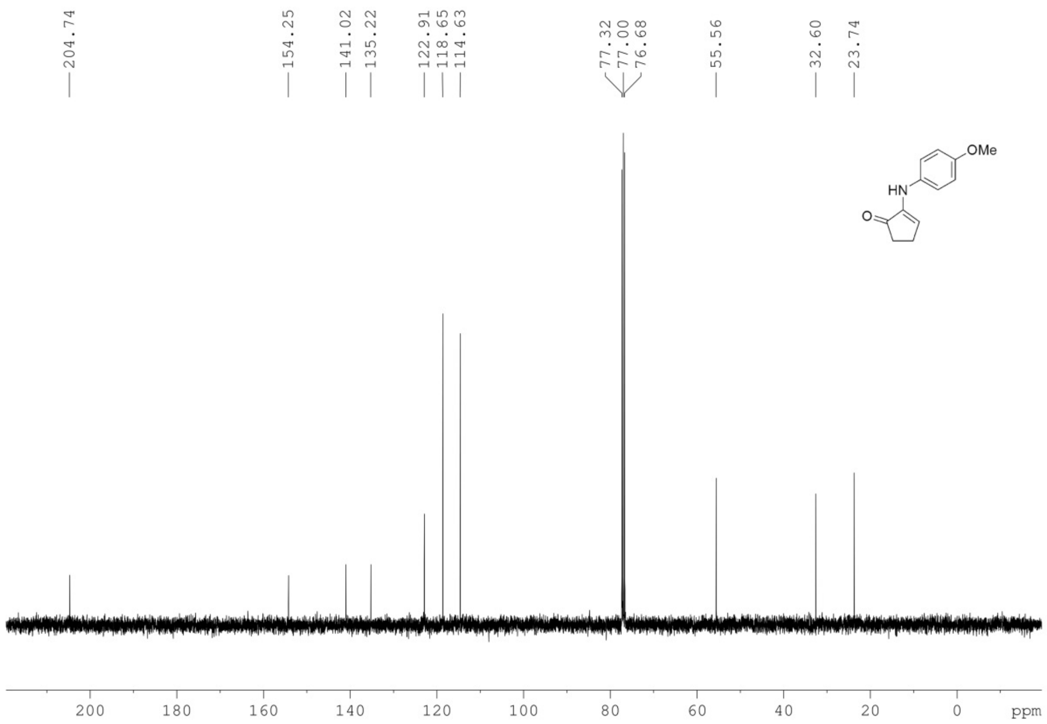

Supplementary Figure 74. ^{13}C NMR spectrum for 2-(4-methoxyphenylamino)cyclopent-2-enone (**3aa**).

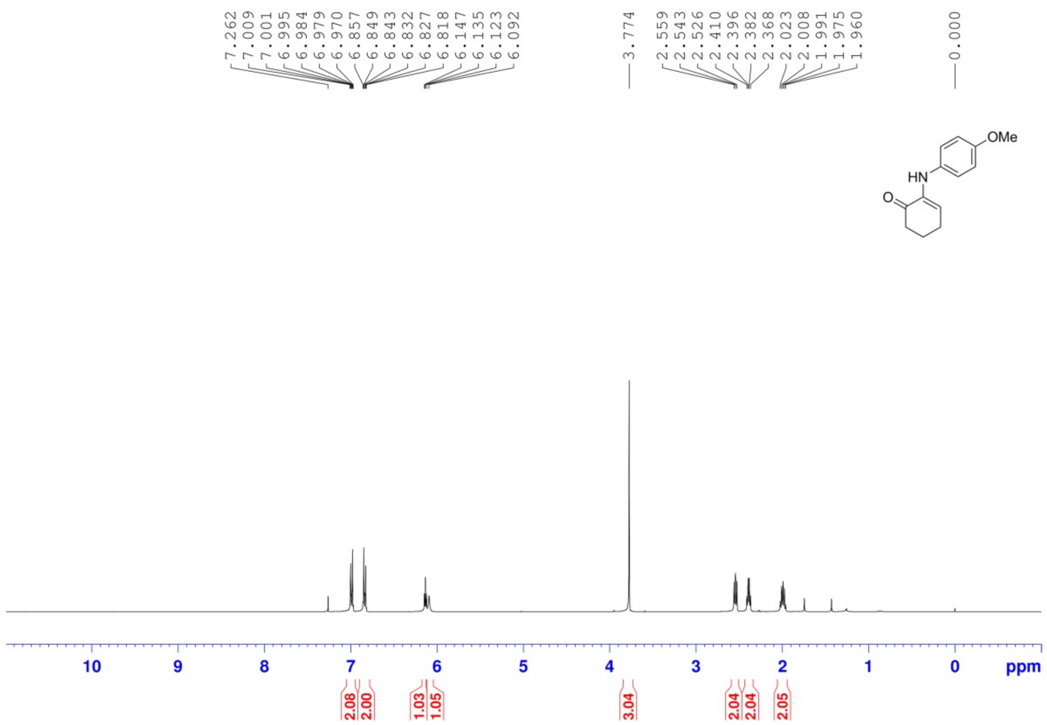

Supplementary Figure 75. ^{1}H NMR spectrum for 2-((4-methoxyphenyl)amino)cyclohex-2-en-1-one (**3ab**).

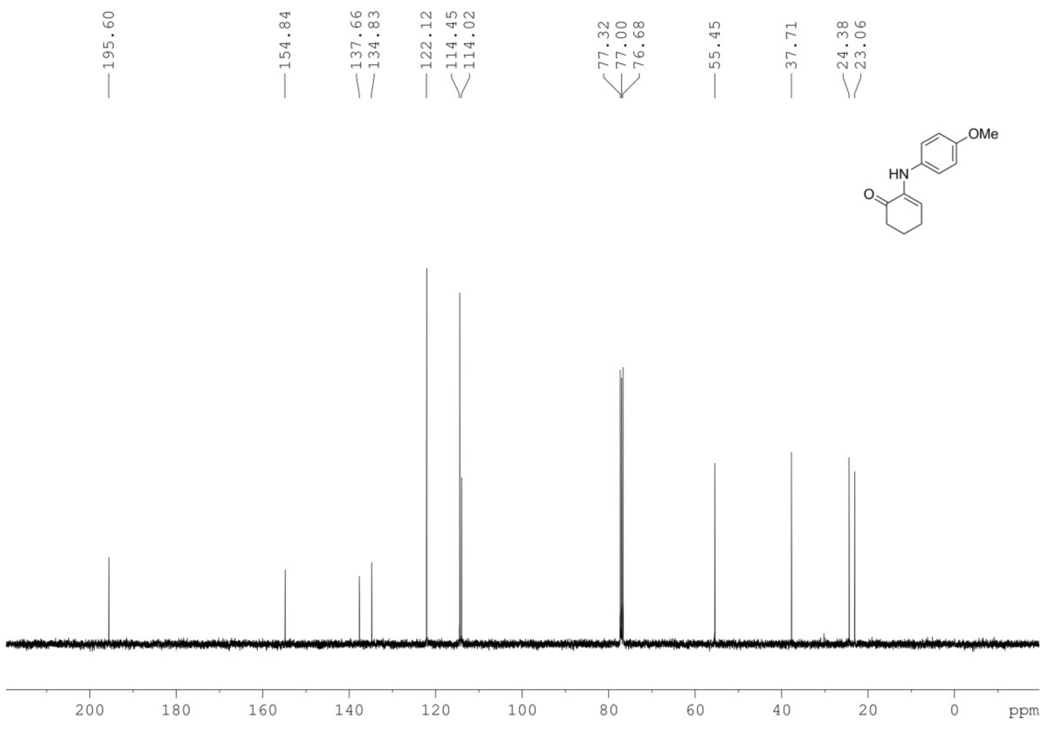

Supplementary Figure 76. ^{13}C NMR spectrum for 2-((4-methoxyphenyl)amino)cyclohex-2-en-1-one (**3ab**).

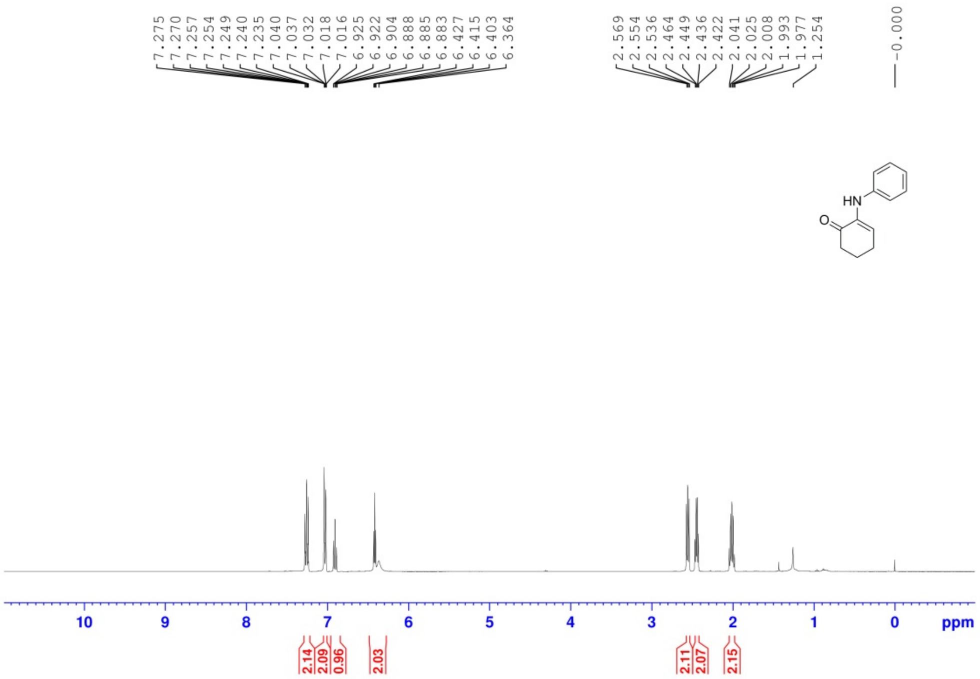

Supplementary Figure 77. ^{1}H NMR spectrum for 2-(phenylamino)cyclohex-2-en-1-one (**3ac**).

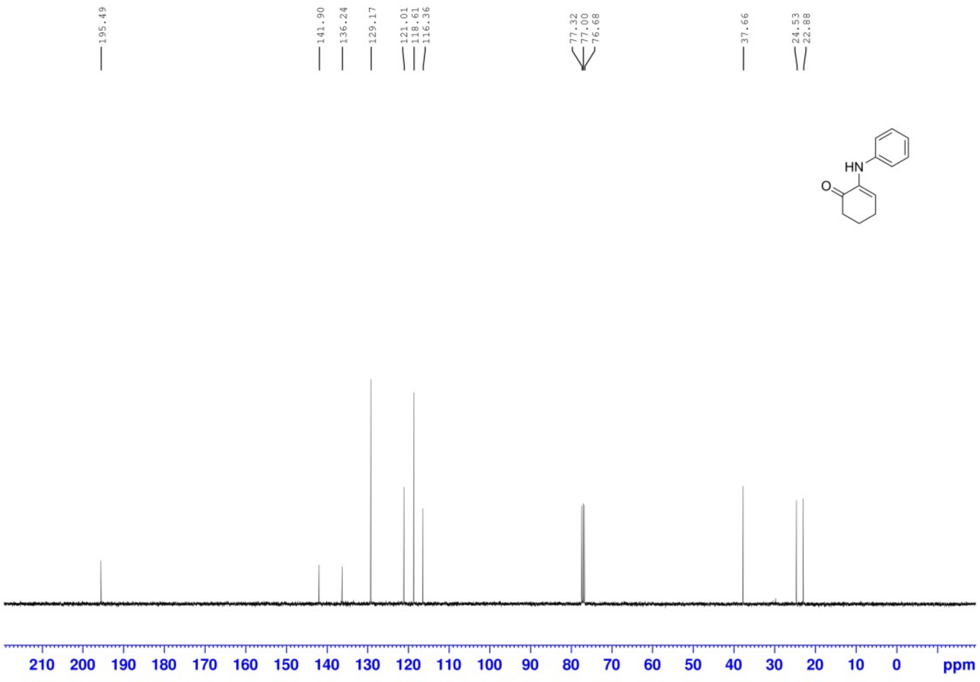

Supplementary Figure 78. ^{13}C NMR spectrum for 2-(phenylamino)cyclohex-2-en-1-one (**3ac**).

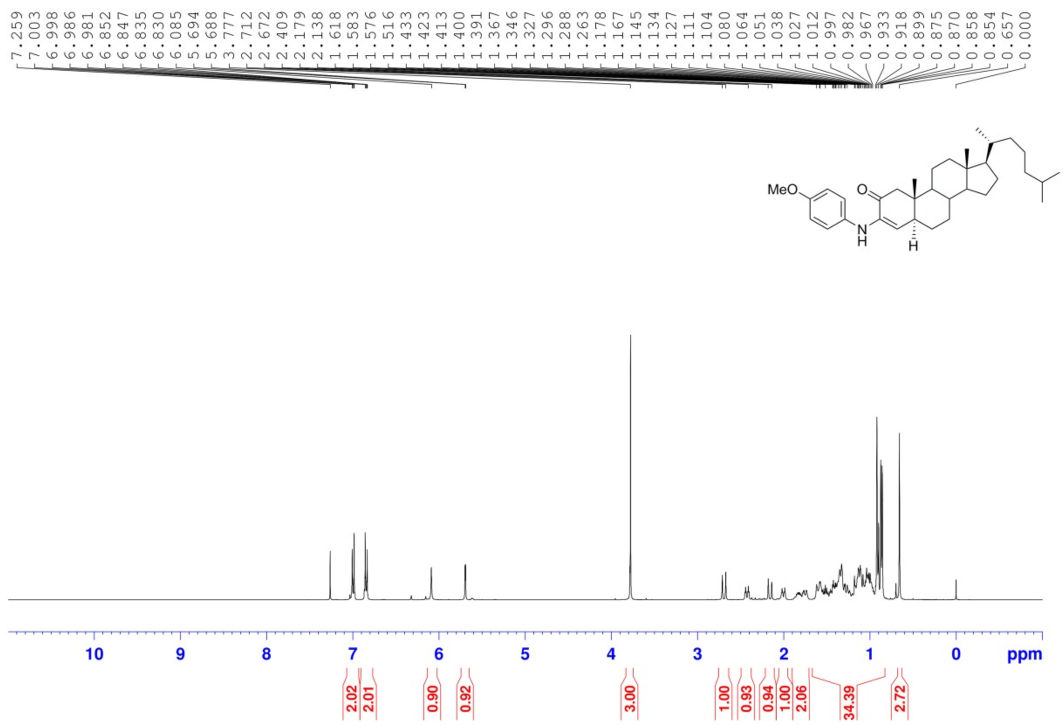

Supplementary Figure 79. ^{1}H NMR spectrum for (5S,8S,9S,10R,13R,14S,17R)-3-((4-methoxyphenyl)amino)-10,13-dimethyl-17-((R)-6-methylheptan-2-yl)-1,5,6,7,8,9,10,11,12,13,14,15,16,17-tetradecahydro-2H-cyclopenta[a]phenanthren-2-one (**3ad**).

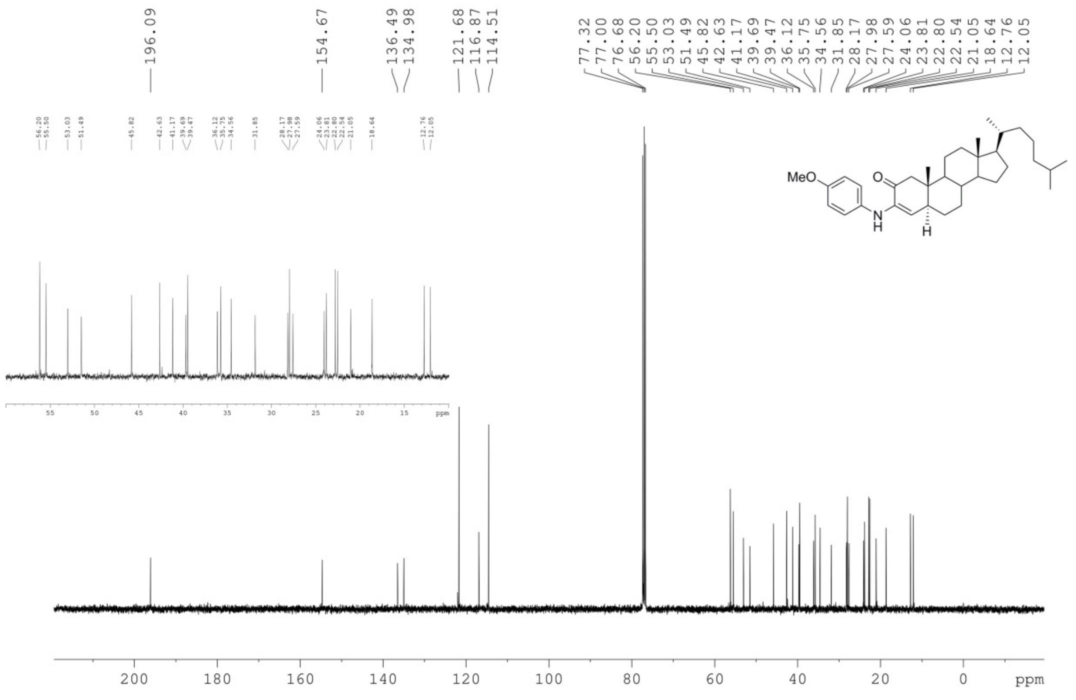

Supplementary Figure 80. ^{13}C NMR spectrum for

(5S,8S,9S,10R,13R,14S,17R)-3-((4-methoxyphenyl)amino)-10,13-dimethyl-17-((R)-6-methylheptan-2-yl)-1,5,6,7,8,9,10,11,12,13,14,15,16,17-tetradecahydro-2H-cyclopenta[a]phenanthren-2-one (**3ad**).

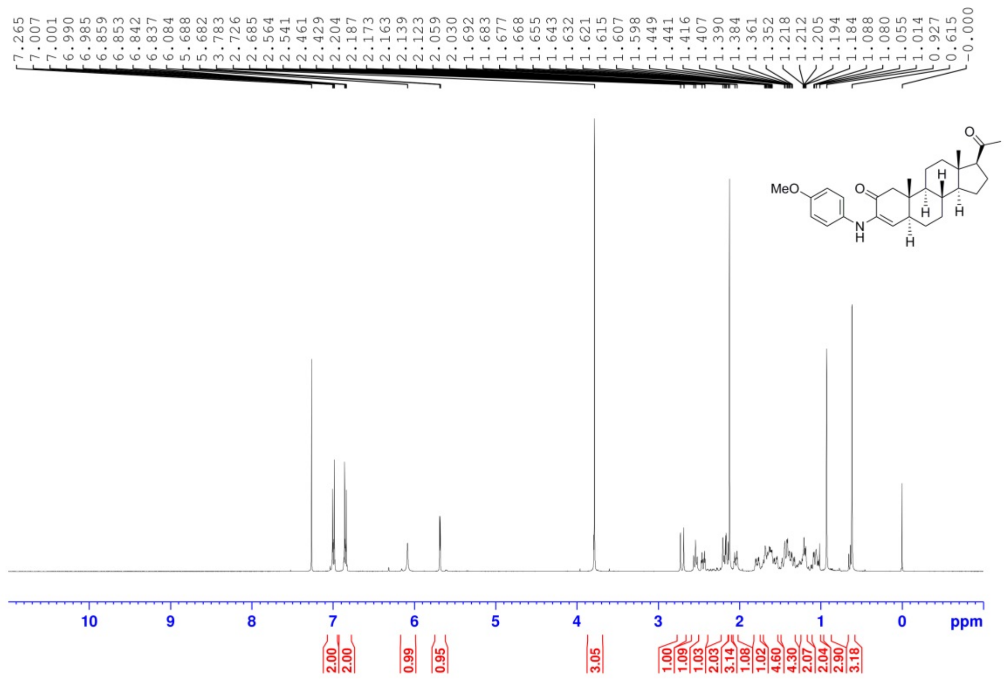

Supplementary Figure 81. ^{1}H NMR spectrum for (5S,8R,9S,10R,13S,14S,17S)-17-acetyl-3-((4-methoxyphenyl)amino)-10,13-dimethyl-1,5,6,7,8,9,10,11,12,13,14,15,16,17-tetradecahydro-2H-cyclopenta[a]phenanthren-2-one (**3ae**).

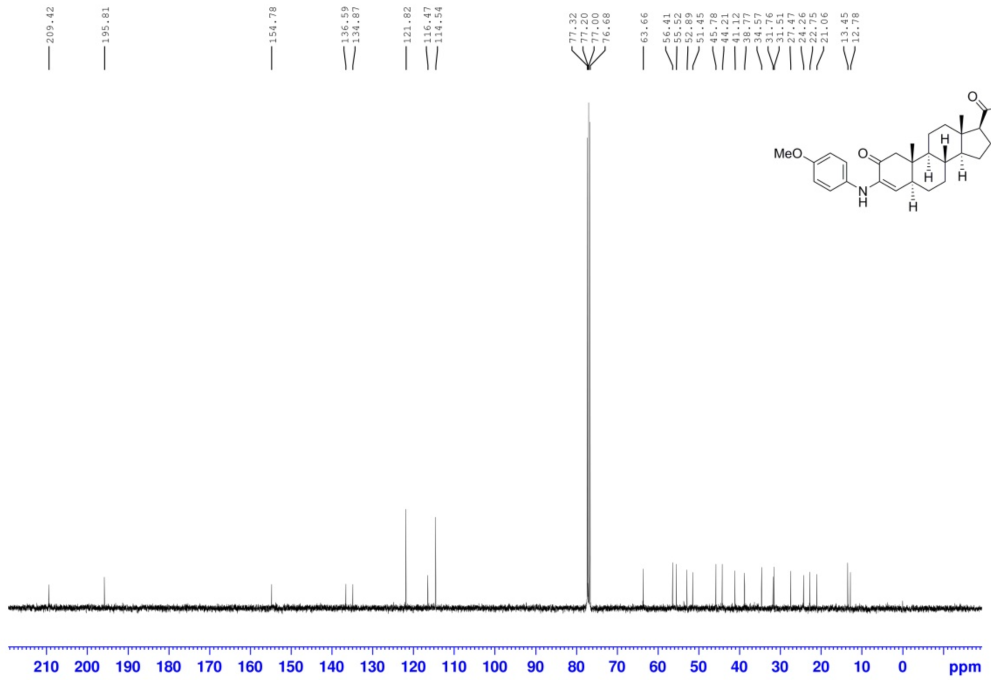

Supplementary Figure 82. ^{13}C NMR spectrum for (5S,8R,9S,10R,13S,14S,17S)-17-acetyl-3-((4-methoxyphenyl)amino)-10,13-dimethyl-1,5,6,7,8,9,10,11,12,13,14,15,16,17-tetradecahydro-2H-cyclopenta[a]phenanthren-2-one (**3ae**).

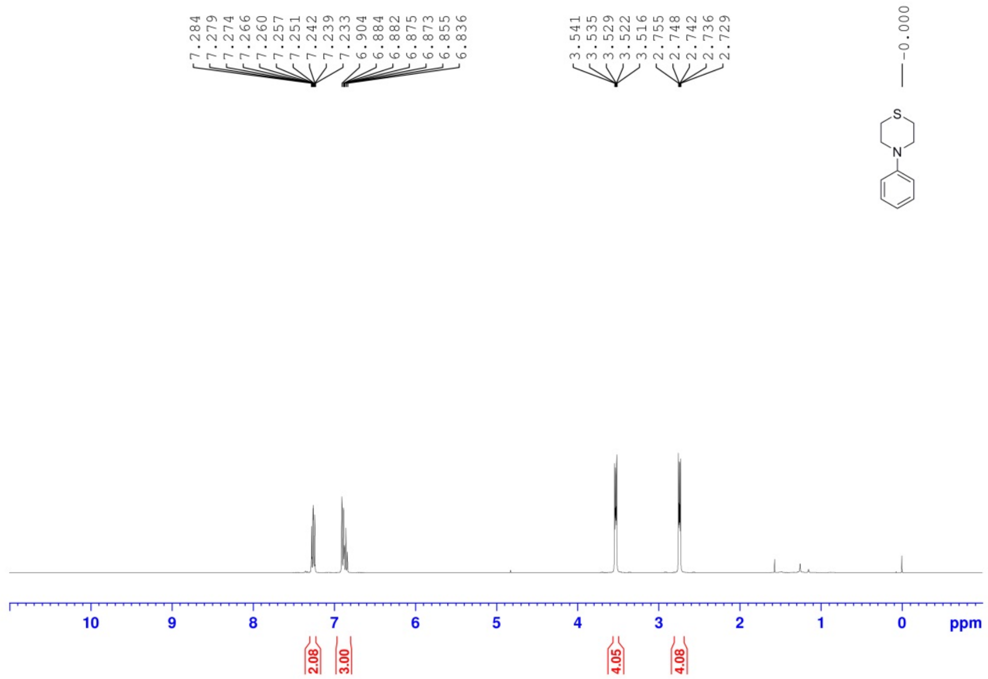

Supplementary Figure 83. ^{1}H NMR spectrum for 4-phenylthiomorpholine (**7b**).

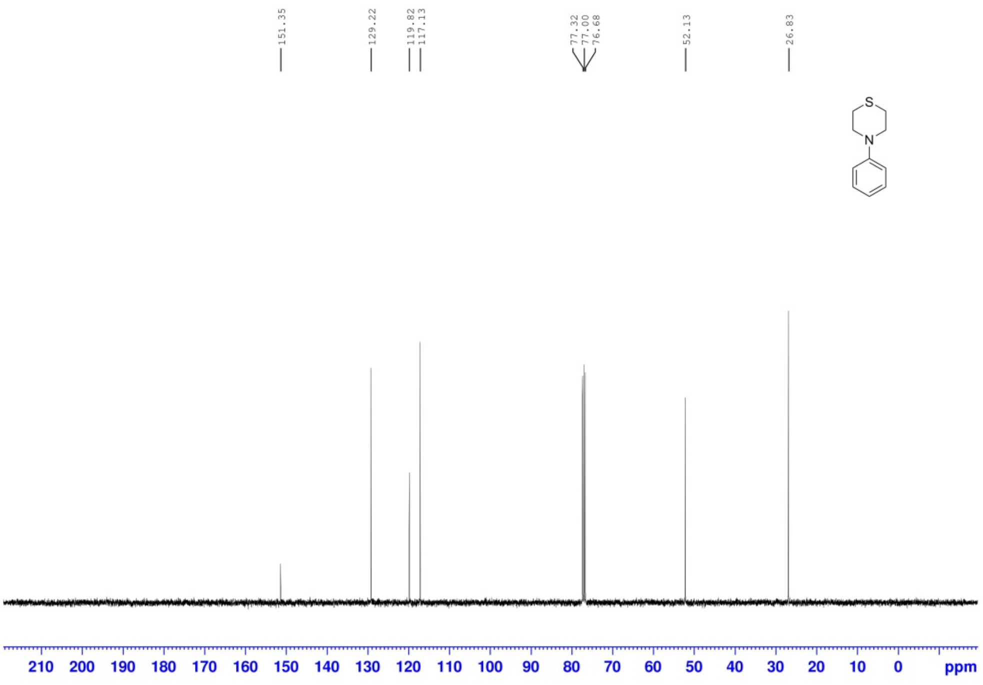

Supplementary Figure 84. ^{13}C NMR spectrum for 4-phenylthiomorpholine (**7b**).

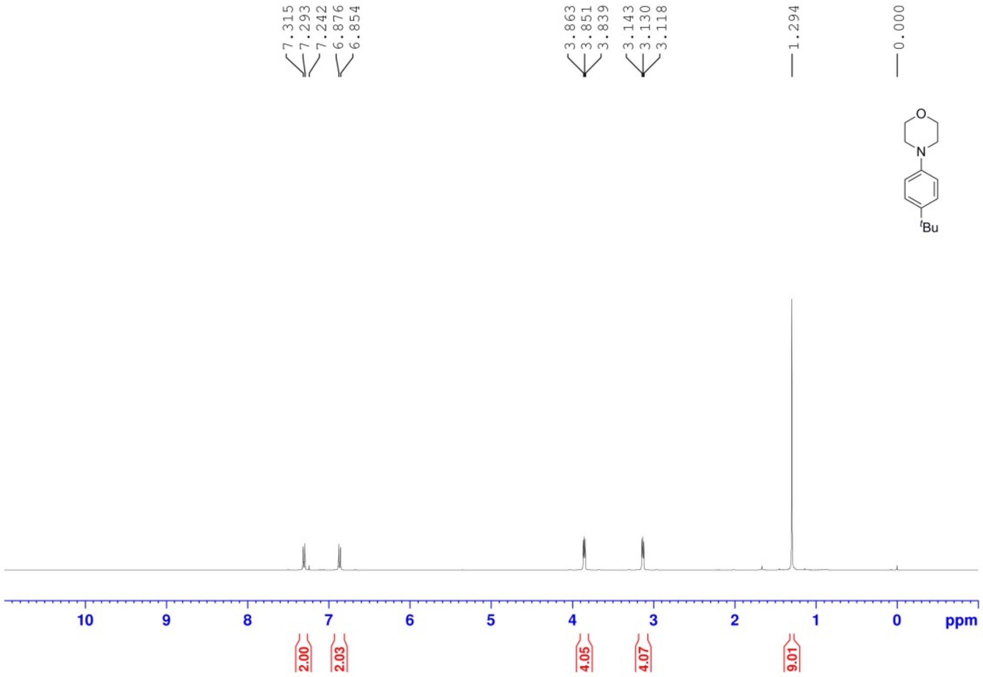

Supplementary Figure 85. ^{1}H NMR spectrum for 4-(4-(tert-butyl)phenyl)morpholine (**7c**).

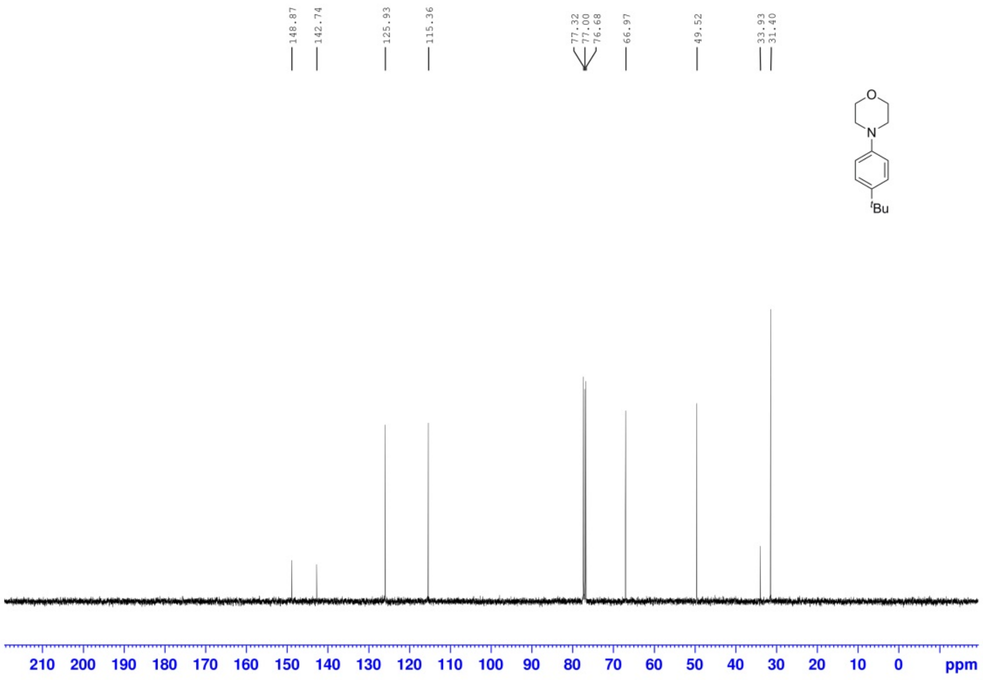

Supplementary Figure 86. ^{13}C NMR spectrum for 4-(4-(tert-butyl)phenyl)morpholine (**7c**).

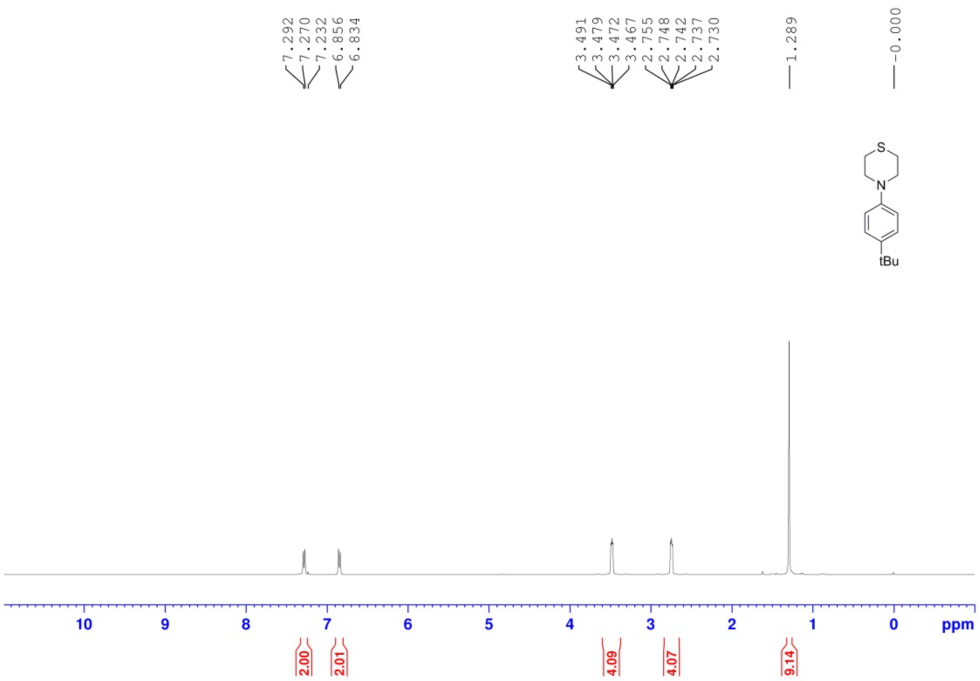

Supplementary Figure 87. ^{1}H NMR spectrum for 4-(4-(tert-butyl)phenyl)thiomorpholine (**7d**).

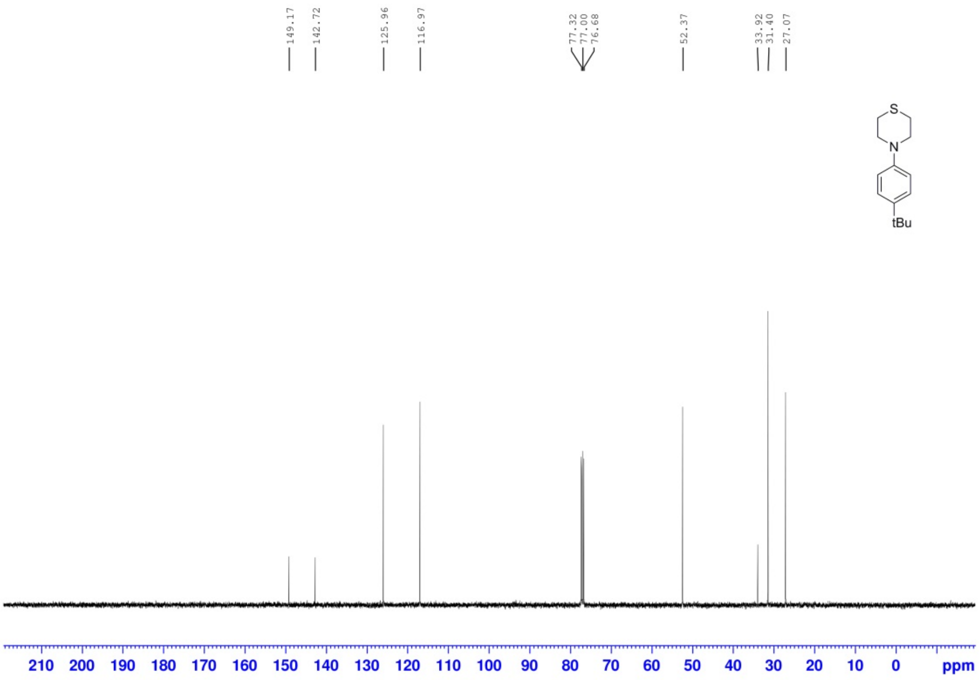

Supplementary Figure 88. ^{13}C NMR spectrum for 4-(4-(tert-butyl)phenyl)thiomorpholine (**7d**).

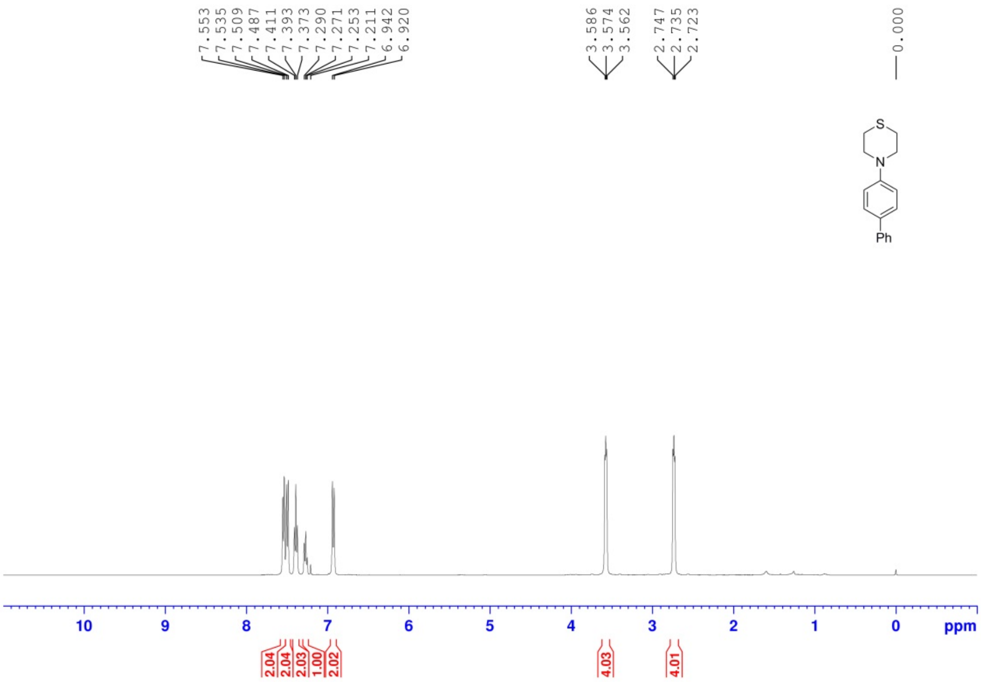

Supplementary Figure 89. ^{1}H NMR spectrum for 4-([1,1'-biphenyl]-4-yl)thiomorpholine (**7e**).

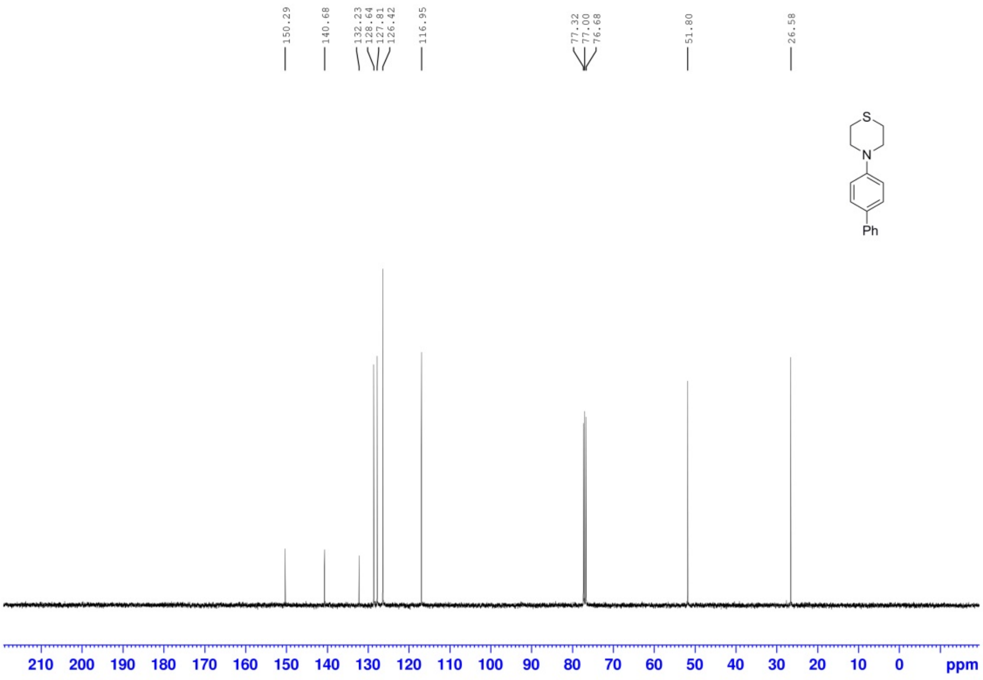

Supplementary Figure 90. ^{13}C NMR spectrum for 4-([1,1'-biphenyl]-4-yl)thiomorpholine (**7e**).

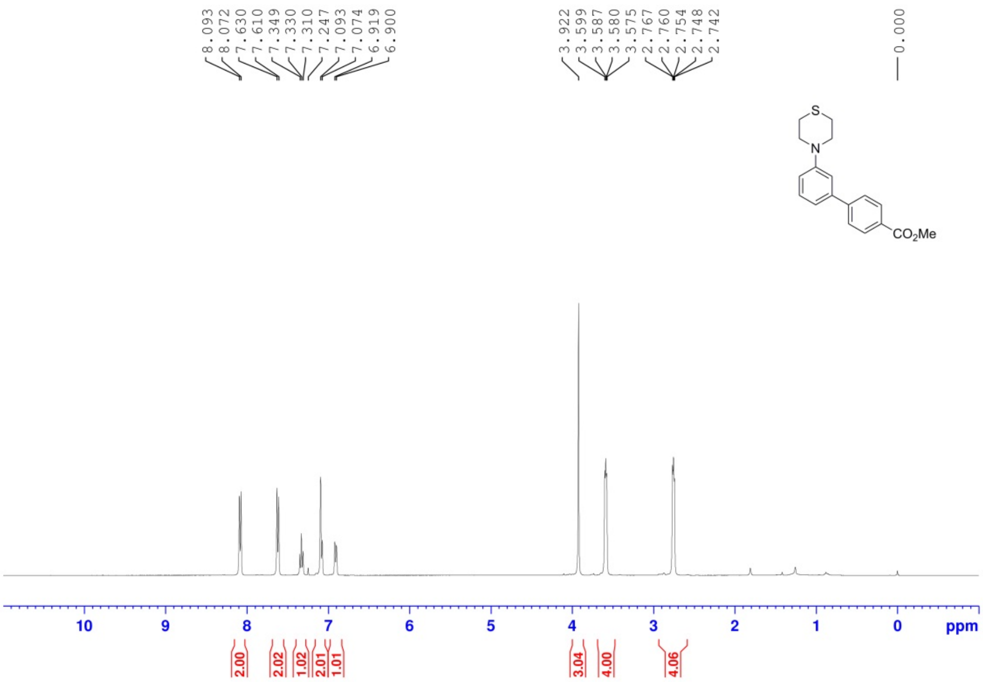

Supplementary Figure 91. ^{1}H NMR spectrum for methyl 3'-thiomorpholino-[1,1'-biphenyl]-4-carboxylate (**7f**).

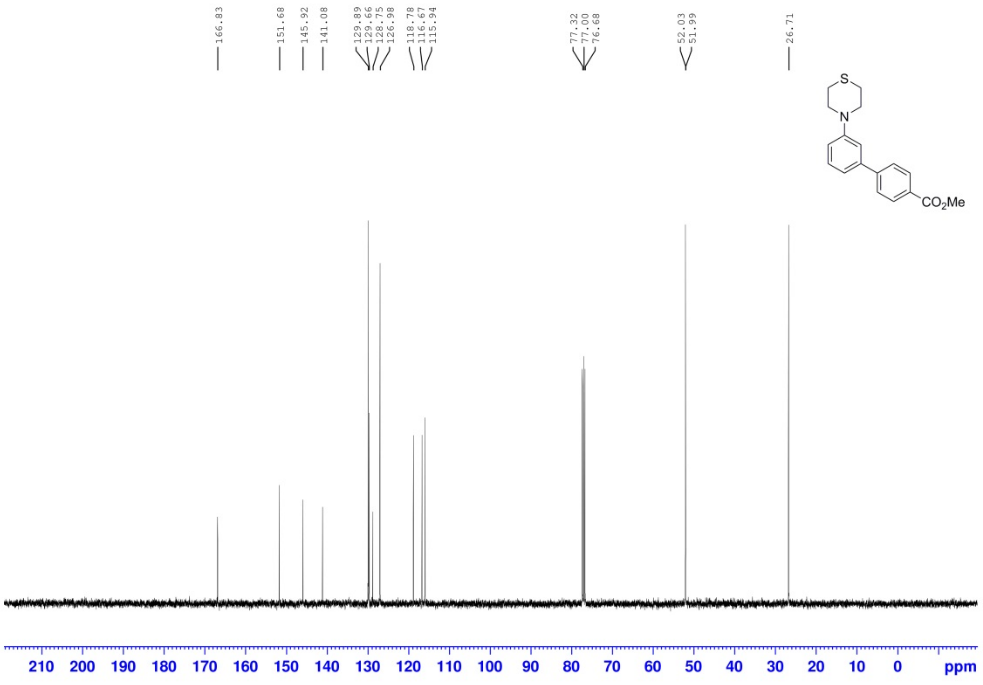

Supplementary Figure 92. ^{13}C NMR spectrum for methyl 3'-thiomorpholino-[1,1'-biphenyl]-4-carboxylate (**7f**).

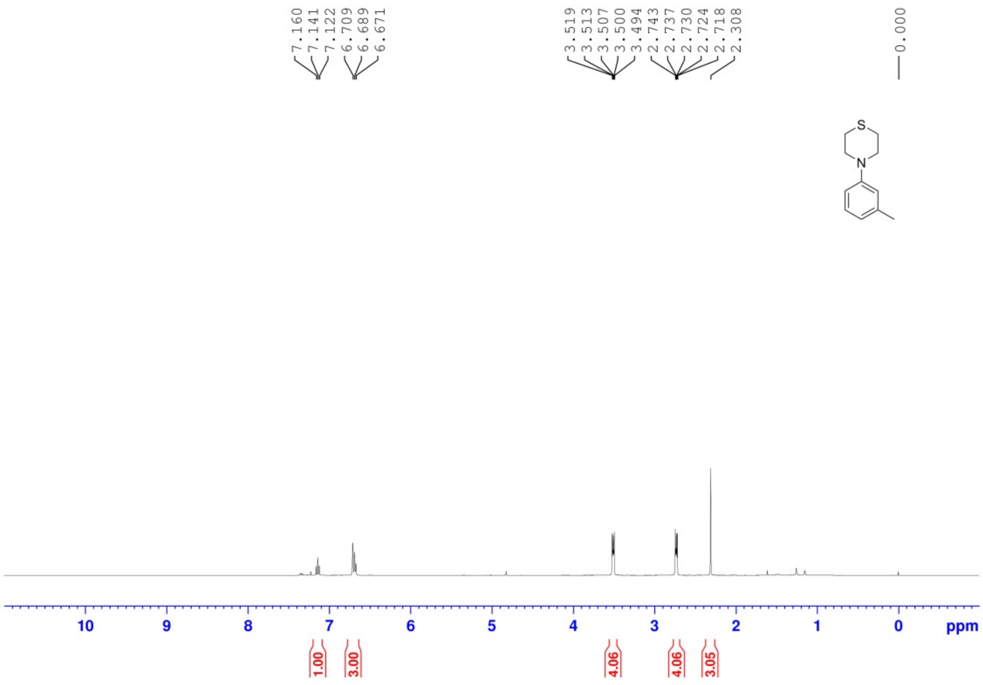

Supplementary Figure 93. ^{1}H NMR spectrum for 4-(*m*-tolyl)thiomorpholine (**7g**).

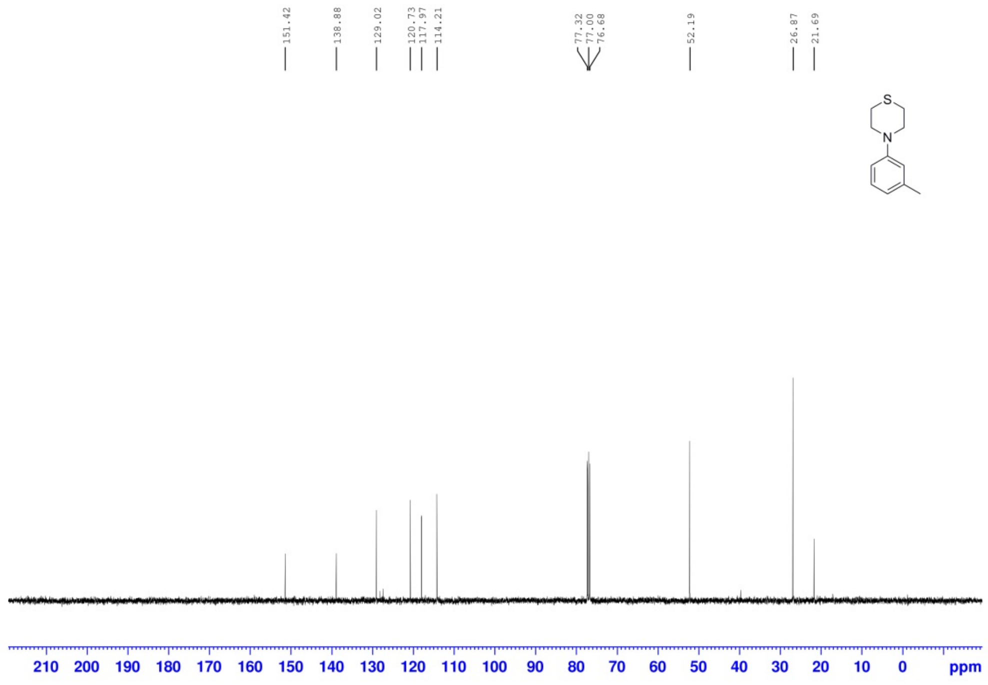

Supplementary Figure 94. ^{13}C NMR spectrum for 4-(*m*-tolyl)thiomorpholine (**7g**).

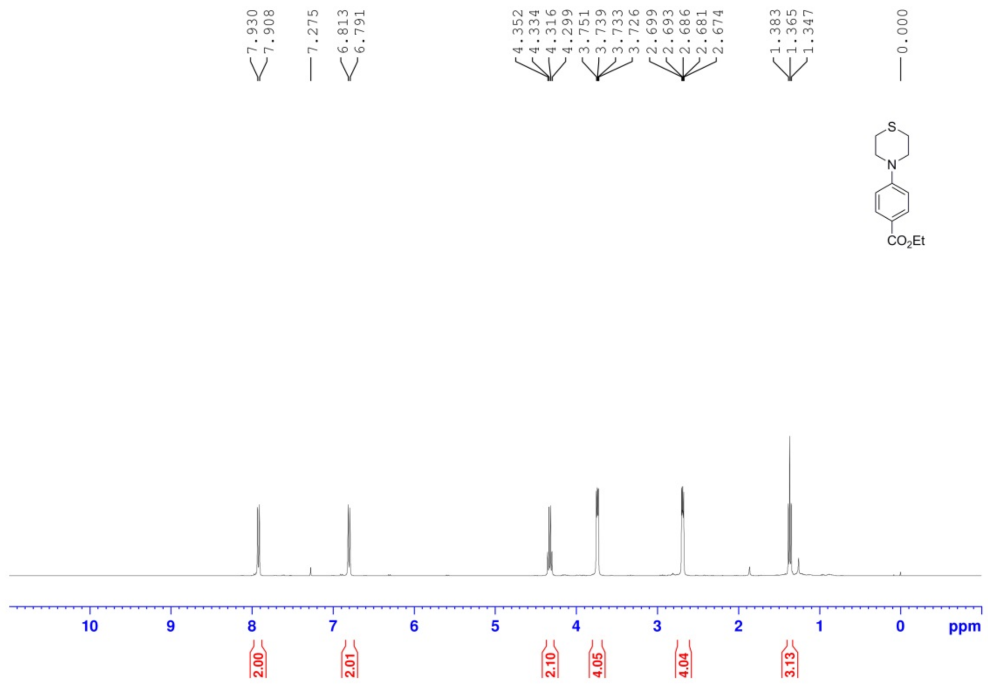

Supplementary Figure 95. ^{1}H NMR spectrum for ethyl 4-thiomorpholinobenzoate (**7h**).

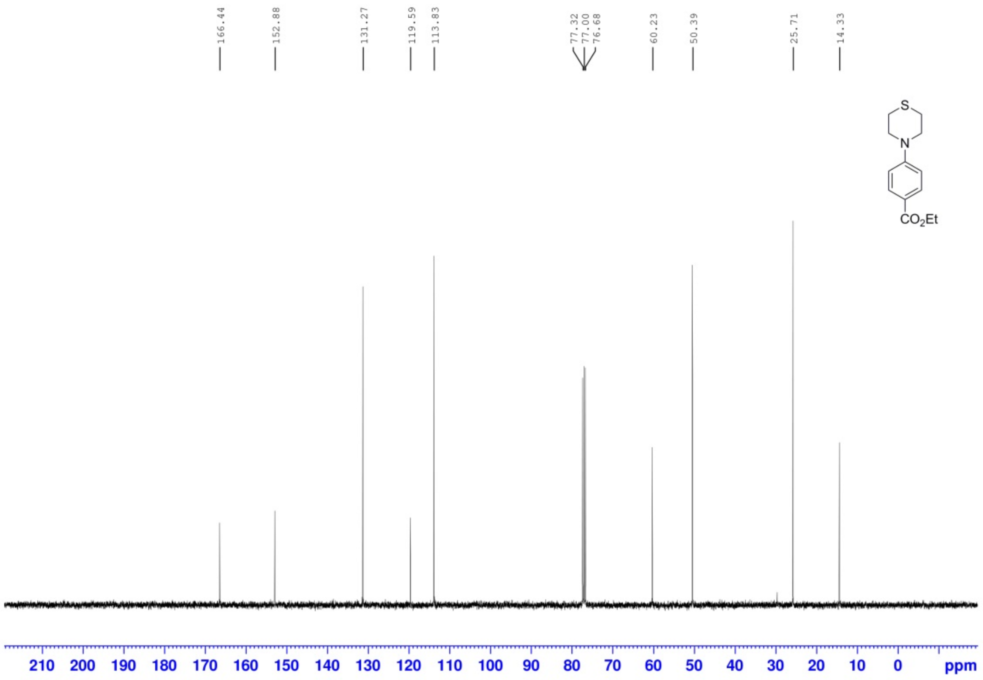

Supplementary Figure 96. ^{13}C NMR spectrum for ethyl 4-thiomorpholinobenzoate (**7h**).

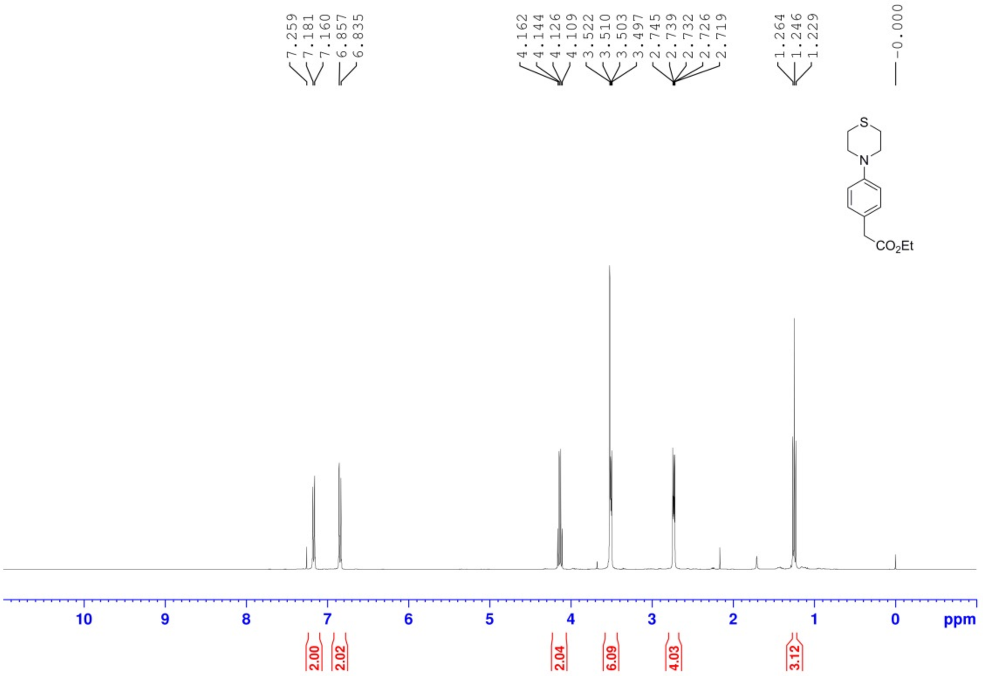

Supplementary Figure 97. ^{1}H NMR spectrum for ethyl 2-(4-thiomorpholinophenyl)acetate (**7i**).

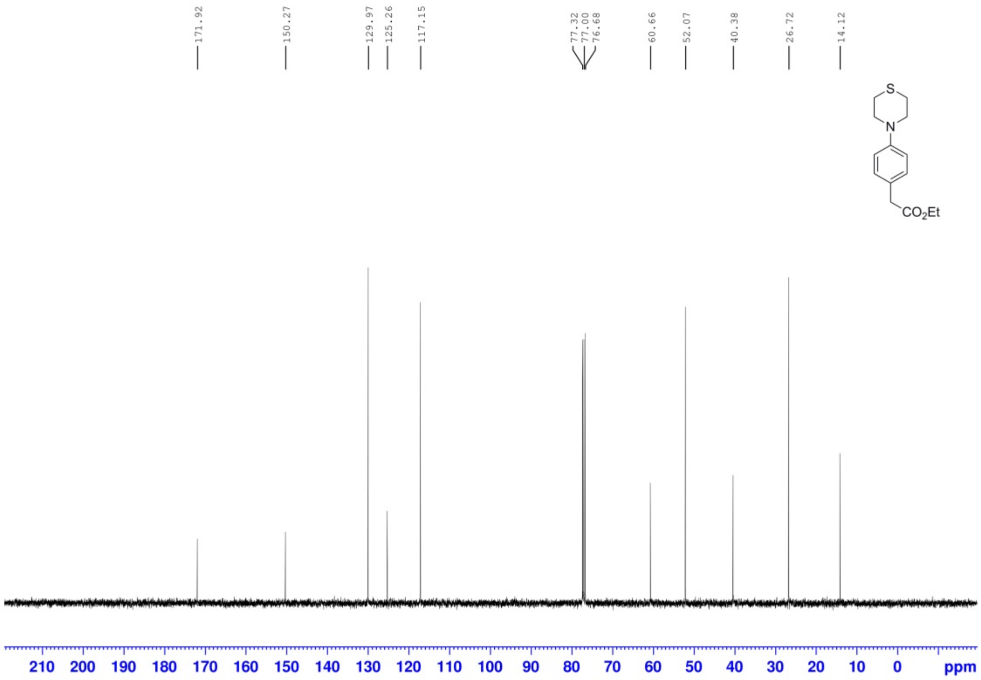

Supplementary Figure 98. ^{13}C NMR spectrum for ethyl 2-(4-thiomorpholinophenyl)acetate (**7i**).

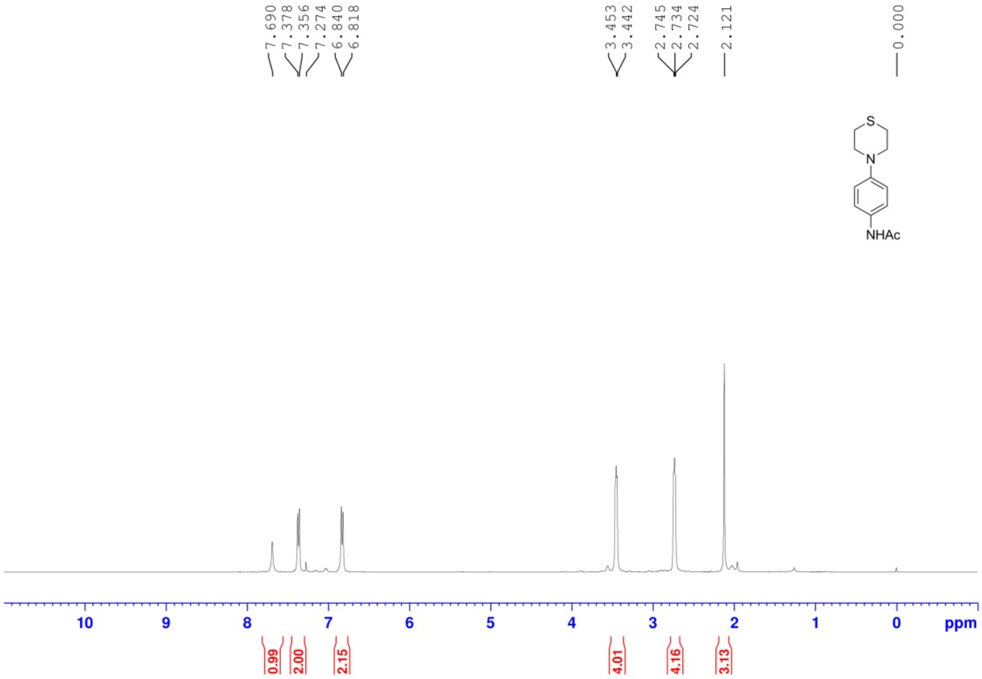

Supplementary Figure 99. ^{1}H NMR spectrum for *N*-(4-thiomorpholinophenyl)acetamide (**7j**).

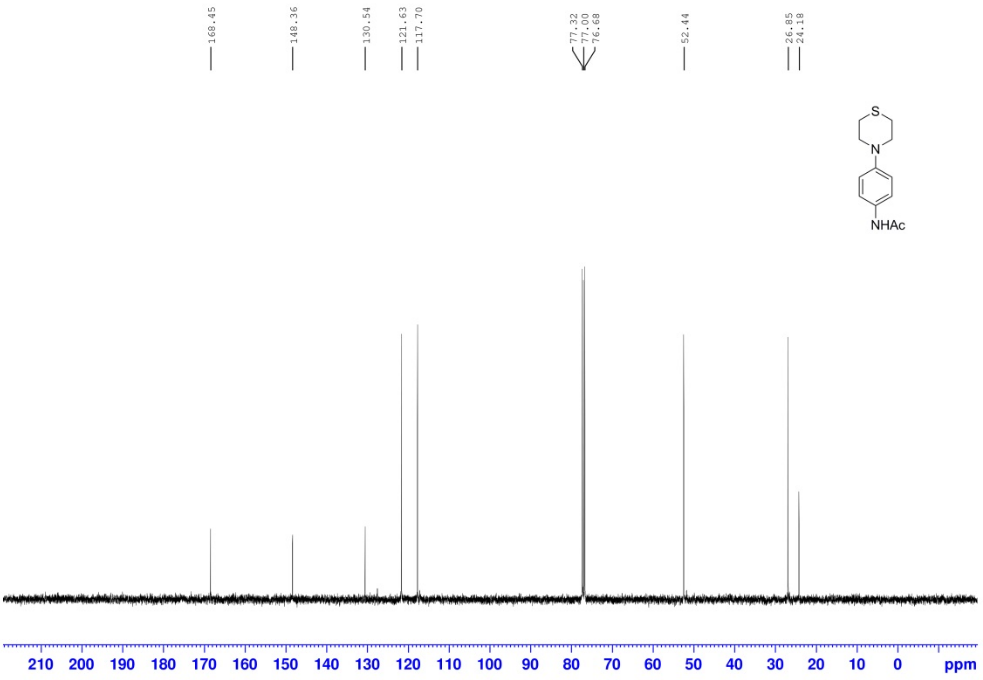

Supplementary Figure 100. ^{13}C NMR spectrum for *N*-(4-thiomorpholinophenyl)acetamide (**7j**).

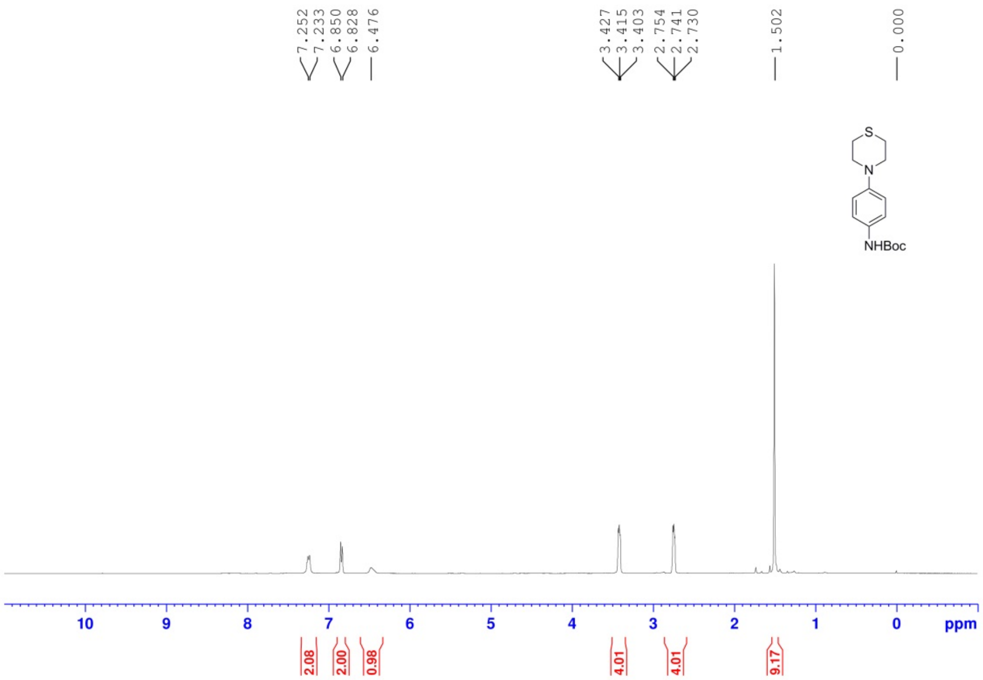

Supplementary Figure 101. ^{1}H NMR spectrum for *tert*-butyl (4-thiomorpholinophenyl)carbamate (**7k**).

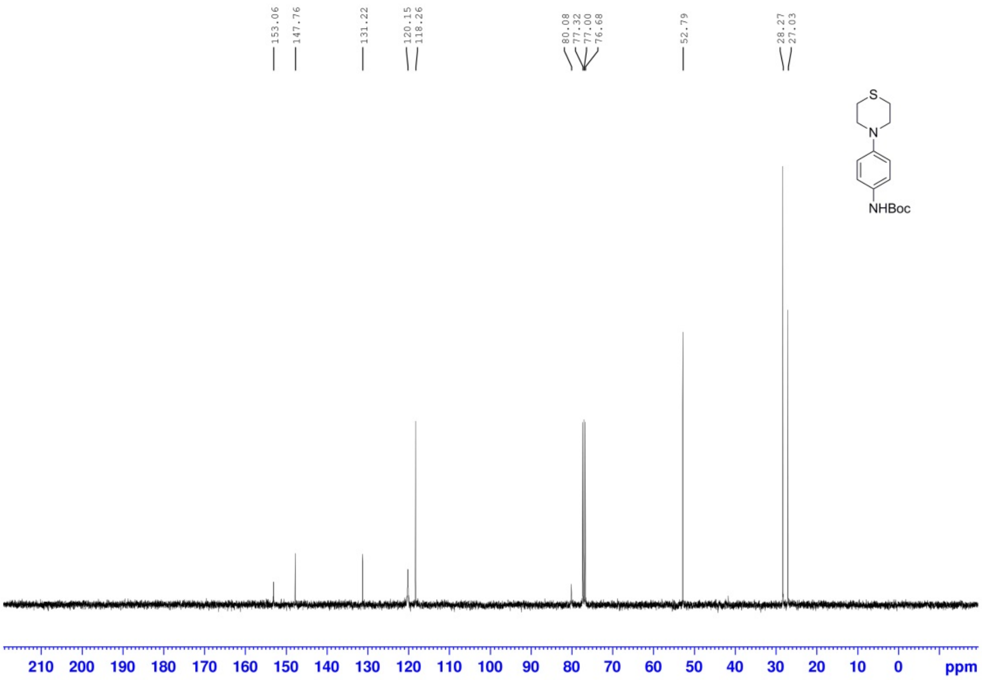

Supplementary Figure 102. ^{13}C NMR spectrum for *tert*-butyl (4-thiomorpholinophenyl)carbamate (**7k**).

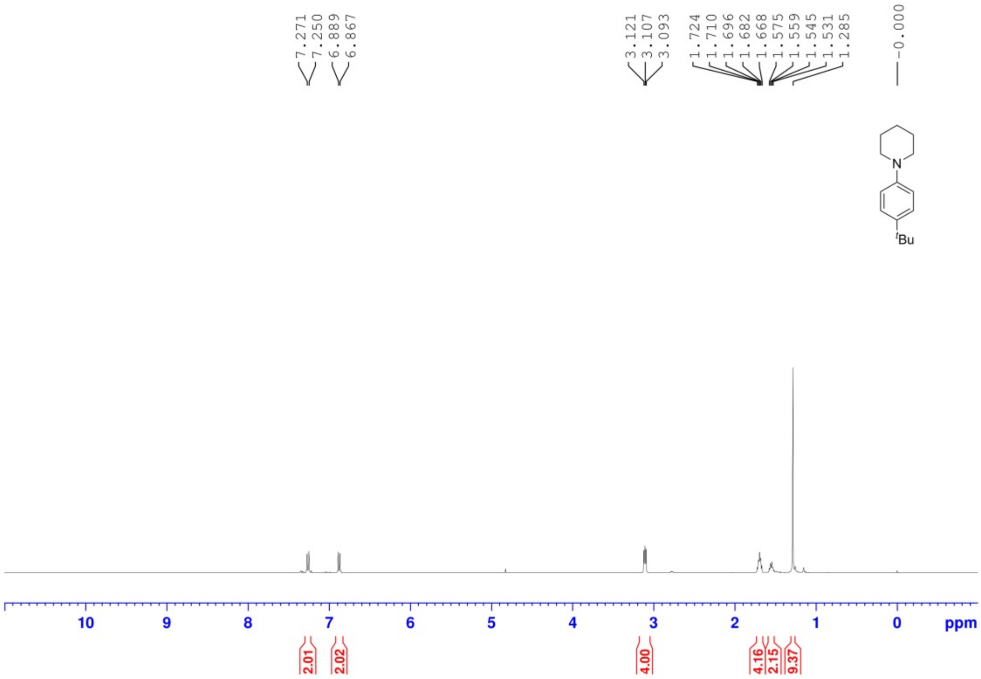

Supplementary Figure 103. ^{1}H NMR spectrum for 1-(4-(*tert*-butyl)phenyl)piperidine (**7l**).

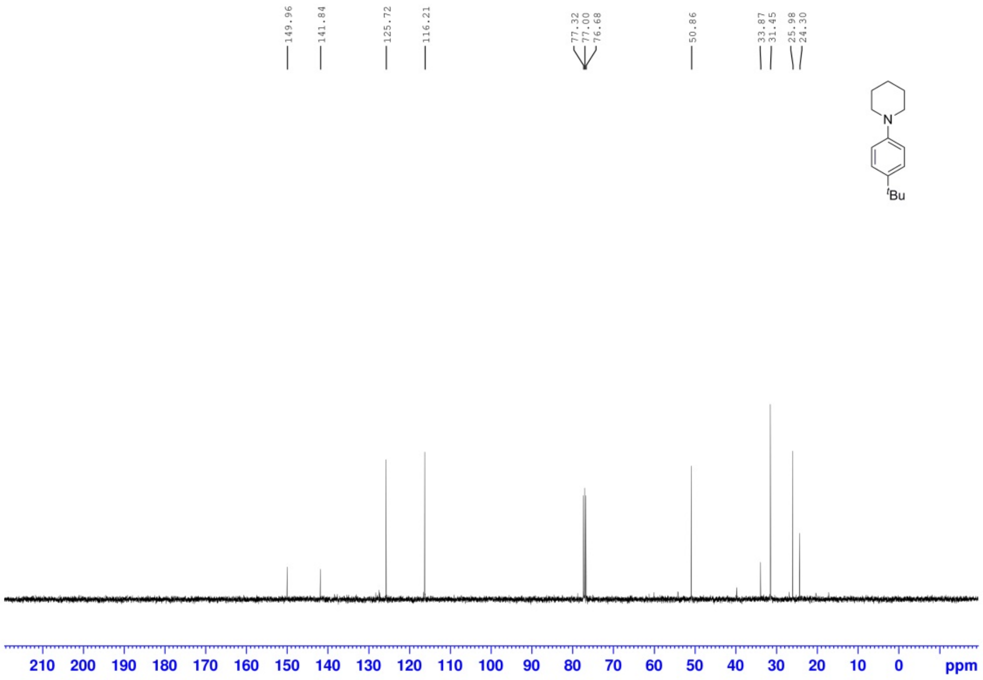

Supplementary Figure 104. ^{13}C NMR spectrum for 1-(4-(*tert*-butyl)phenyl)piperidine (**7l**).

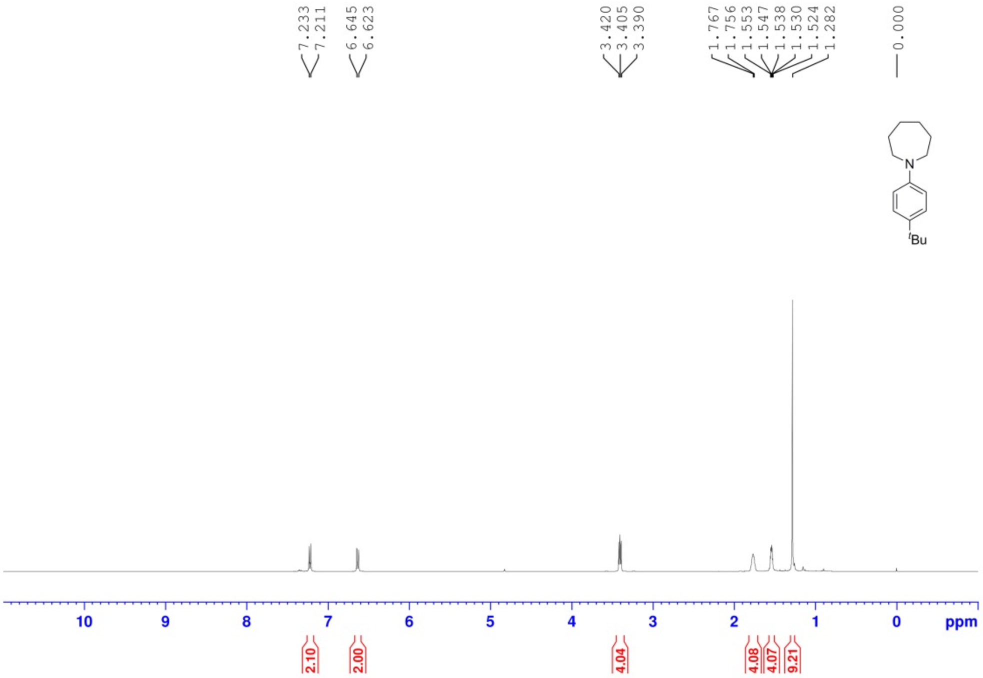

Supplementary Figure 105. ^{1}H NMR spectrum for 1-(4-(*tert*-butyl)phenyl)azepane (**7m**).

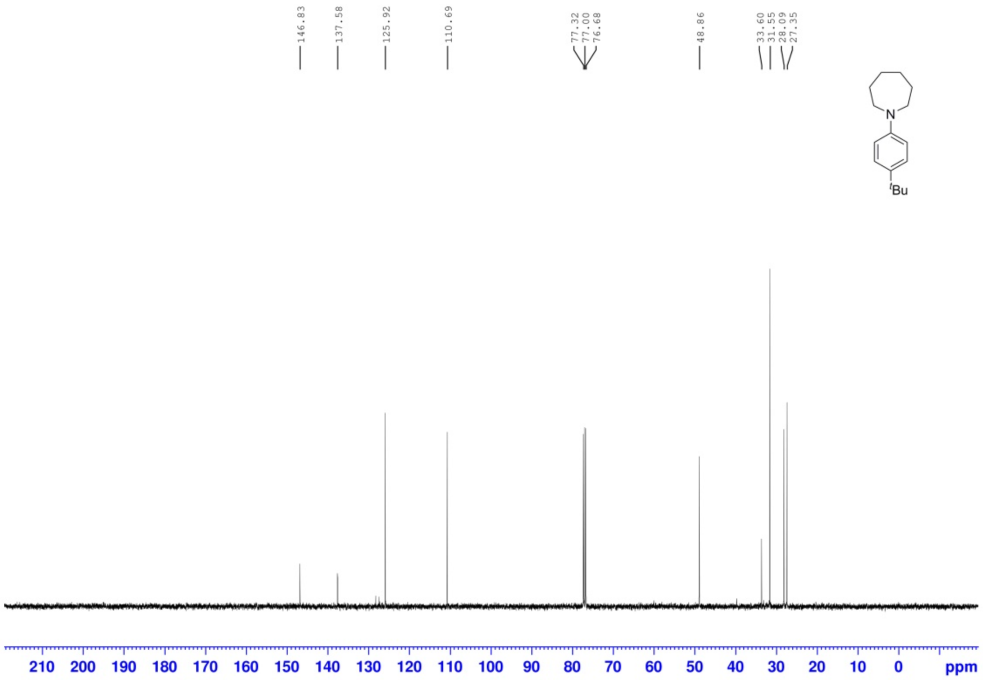

Supplementary Figure 106. ^{13}C NMR spectrum for 1-(4-(*tert*-butyl)phenyl)azepane (**7m**).

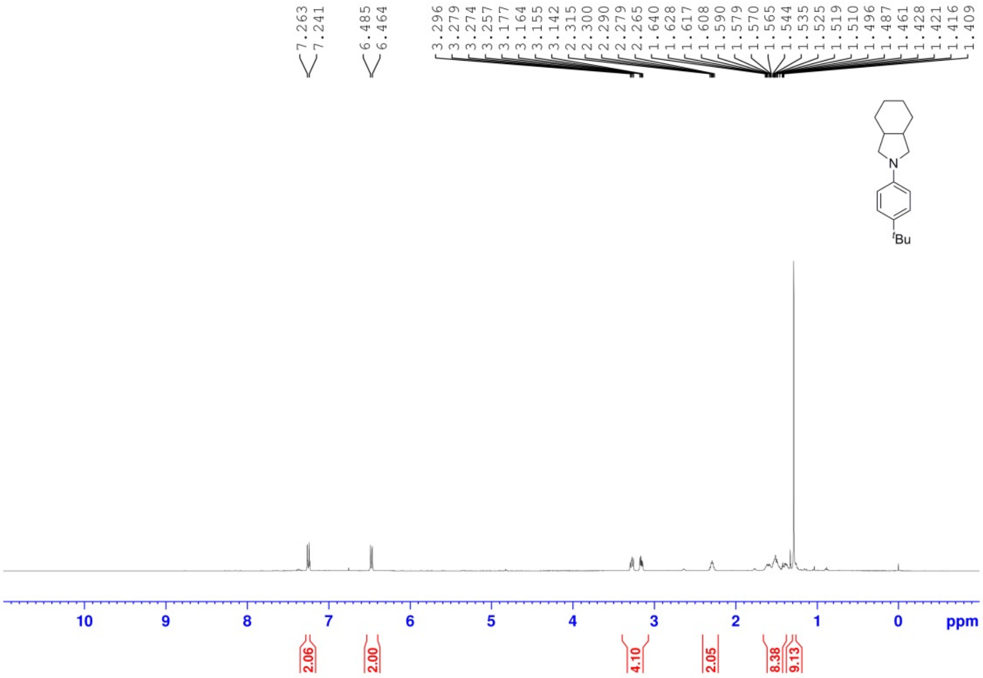

Supplementary Figure 107. ^{1}H NMR spectrum for 2-(4-(*tert*-butyl)phenyl)octahydro-1*H*-isoindole (**7n**).

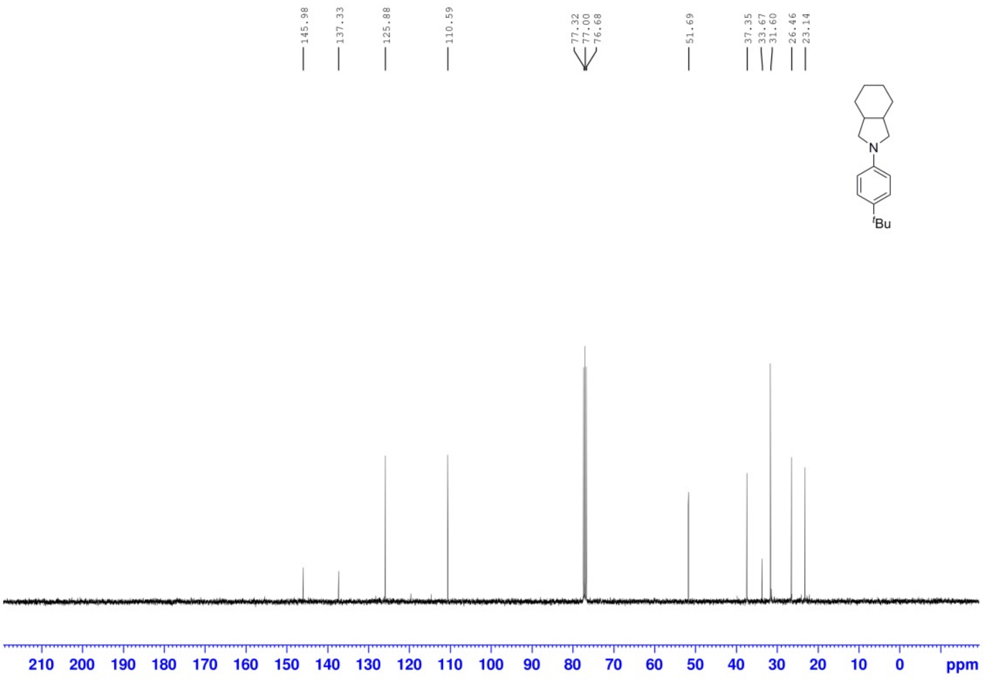

Supplementary Figure 108. ^{13}C NMR spectrum for 2-(4-(*tert*-butyl)phenyl)octahydro-1*H*-isoindole (**7n**).

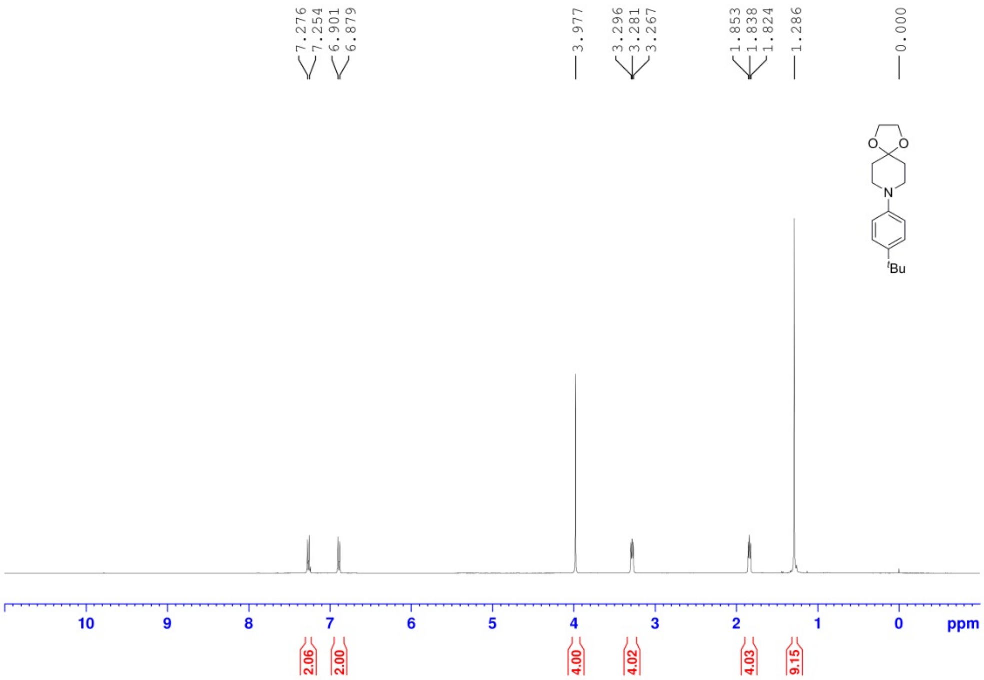

Supplementary Figure 109. ^{1}H NMR spectrum for 8-(4-(*tert*-butyl)phenyl)-1,4-dioxa-8-azaspiro[4.5]decane (**7o**).

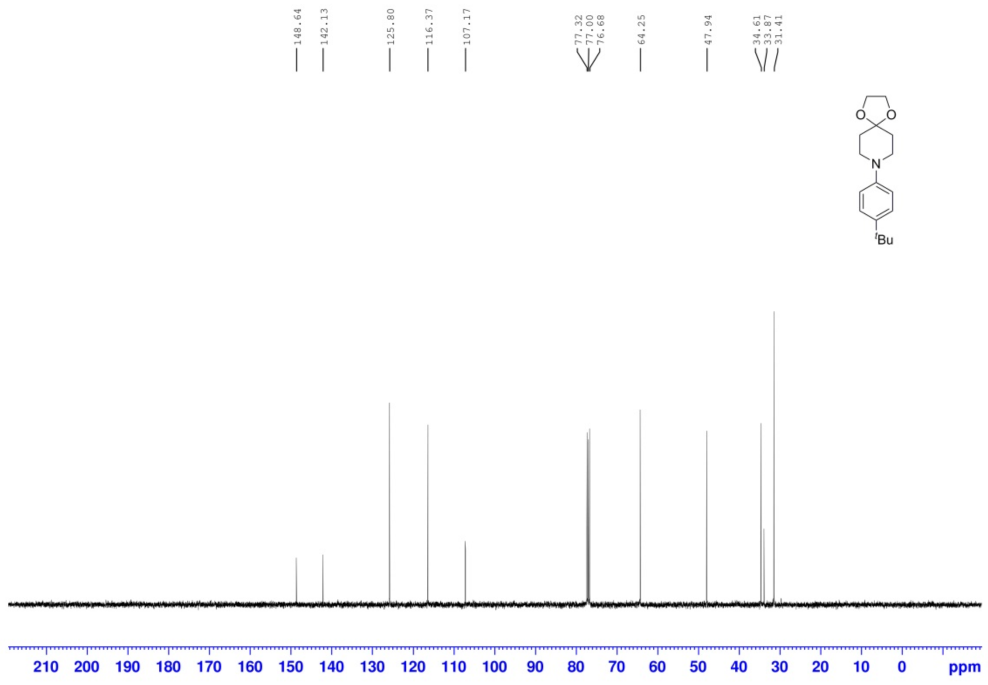

Supplementary Figure 110. ^{13}C NMR spectrum for
8-(4-(*tert*-butyl)phenyl)-1,4-dioxa-8-azaspiro[4.5]decane (**7o**).

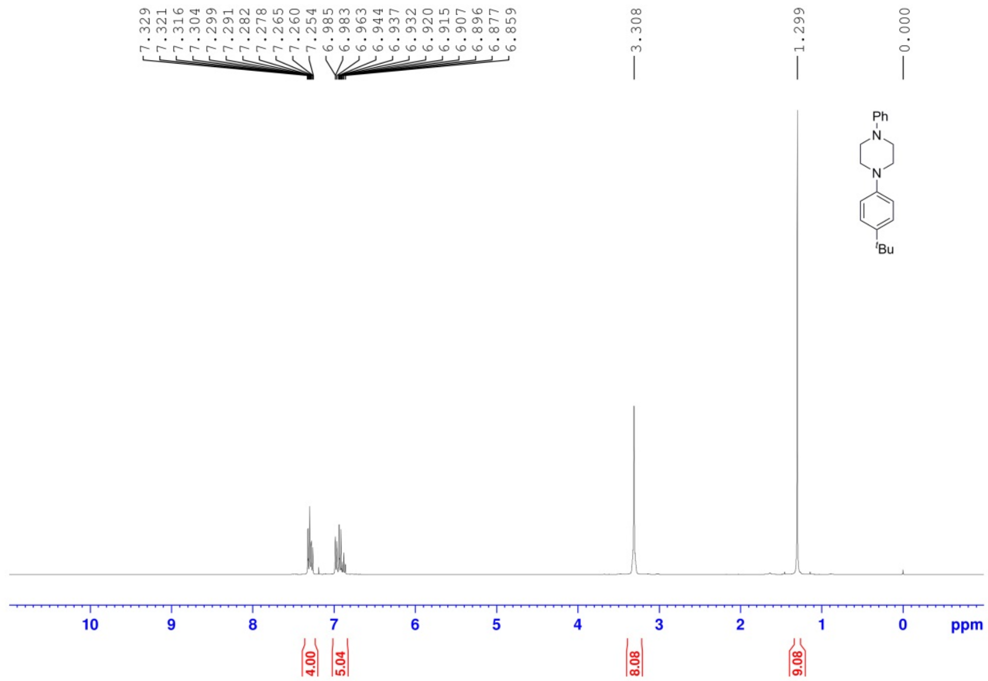

Supplementary Figure 111. ^{1}H NMR spectrum for
1-(4-(*tert*-butyl)phenyl)-4-phenylpiperazine (**7p**).

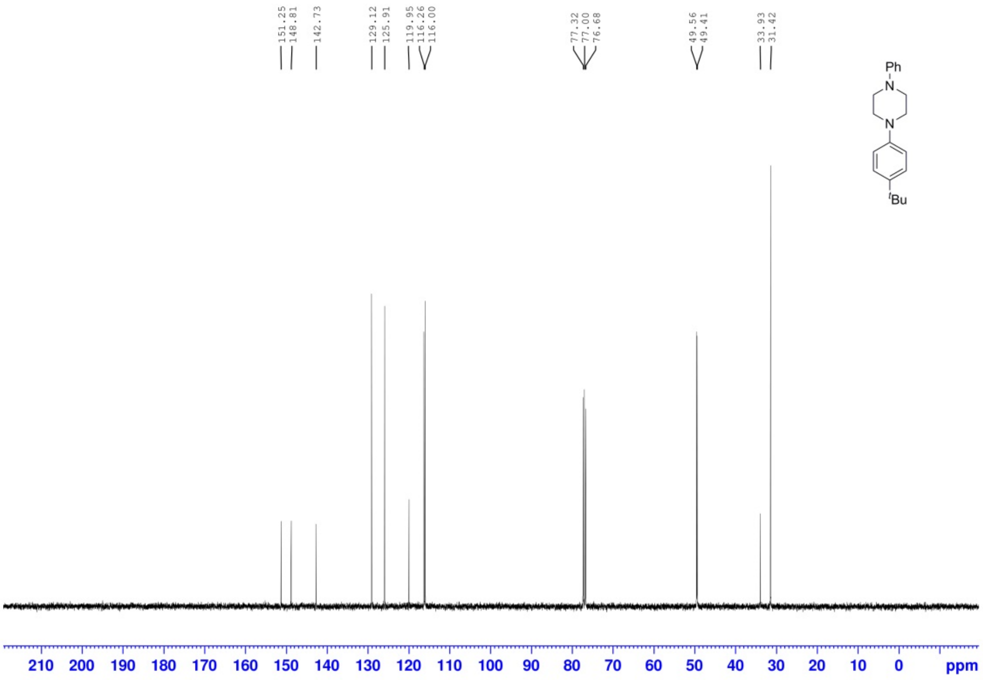

Supplementary Figure 112. ^{13}C NMR spectrum for 1-(4-(*tert*-butyl)phenyl)-4-phenylpiperazine (**7p**).

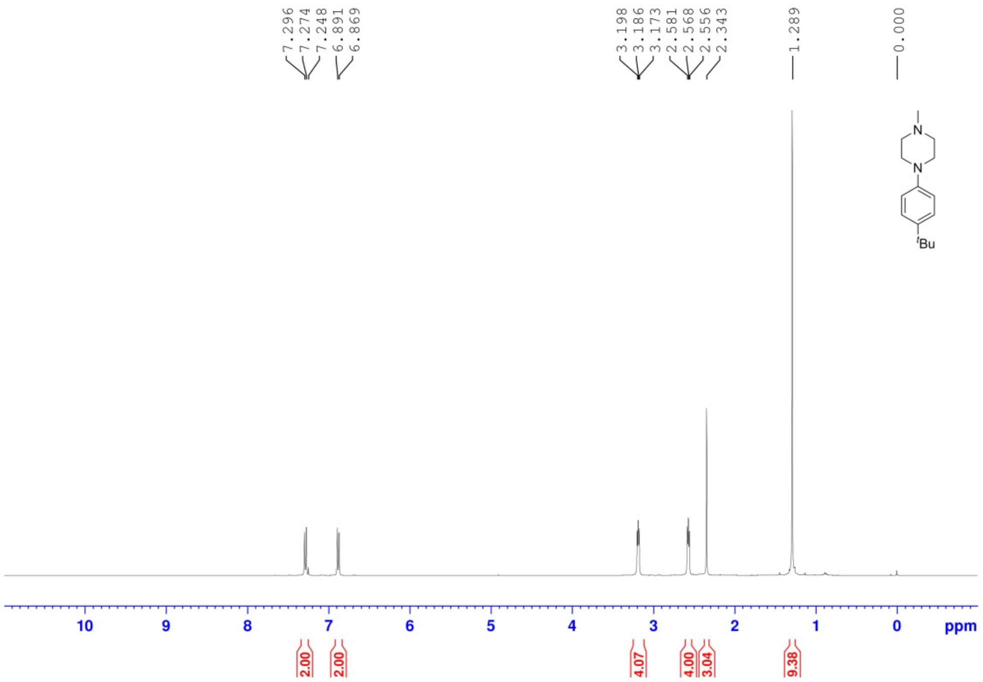

Supplementary Figure 113. ^{1}H NMR spectrum for 1-(4-(*tert*-butyl)phenyl)-4-methylpiperazine (**7q**).

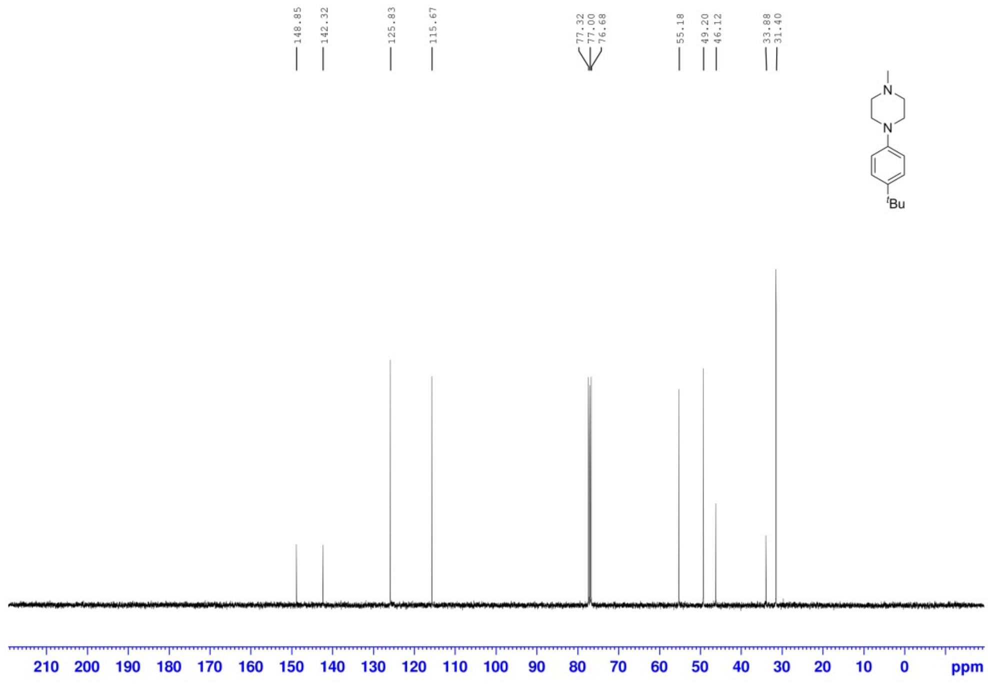

Supplementary Figure 114. ^{13}C NMR spectrum for 1-(4-(*tert*-butyl)phenyl)-4-methylpiperazine (**7q**).

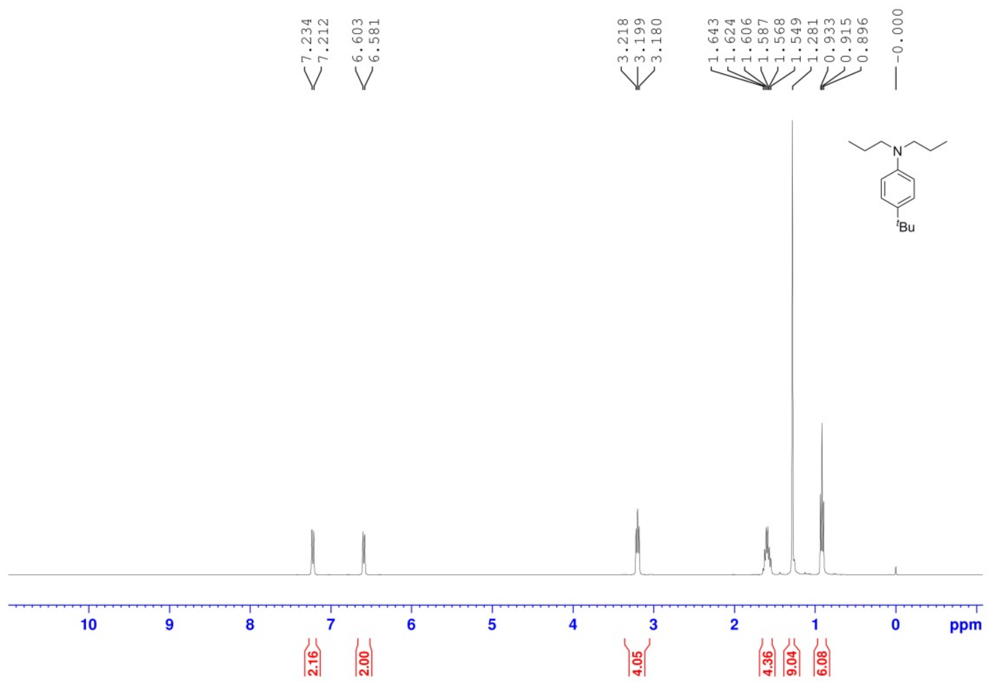

Supplementary Figure 115. ^{1}H NMR spectrum for 4-(*tert*-butyl)-*N*,*N*-dipropylaniline (**7r**).

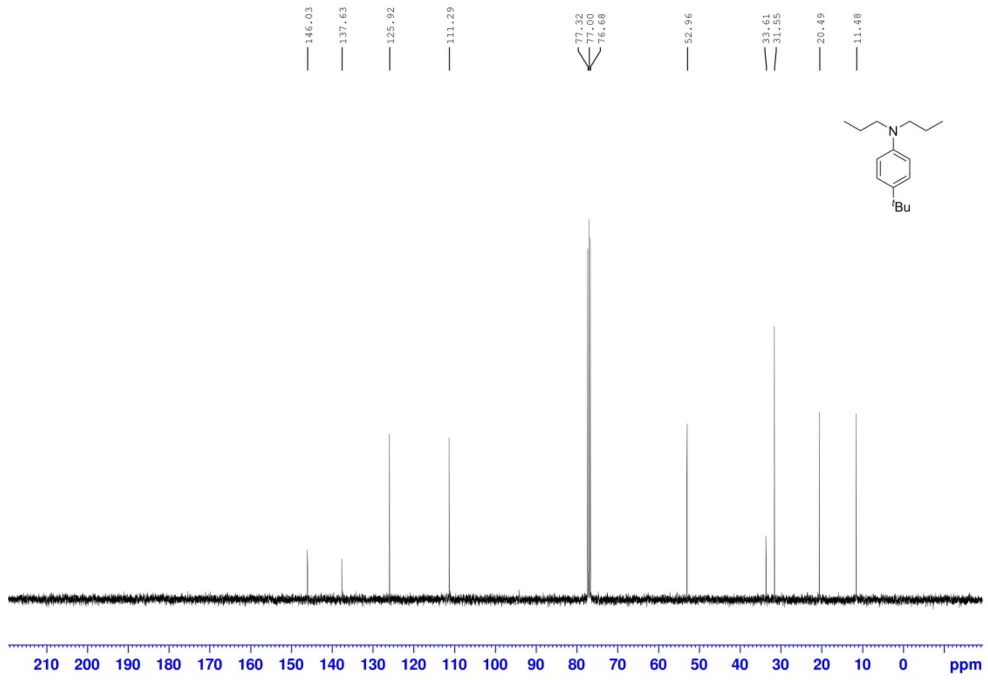

Supplementary Figure 116. ^{13}C NMR spectrum for 4-(*tert*-butyl)-*N*,*N*-dipropylaniline (**7r**).

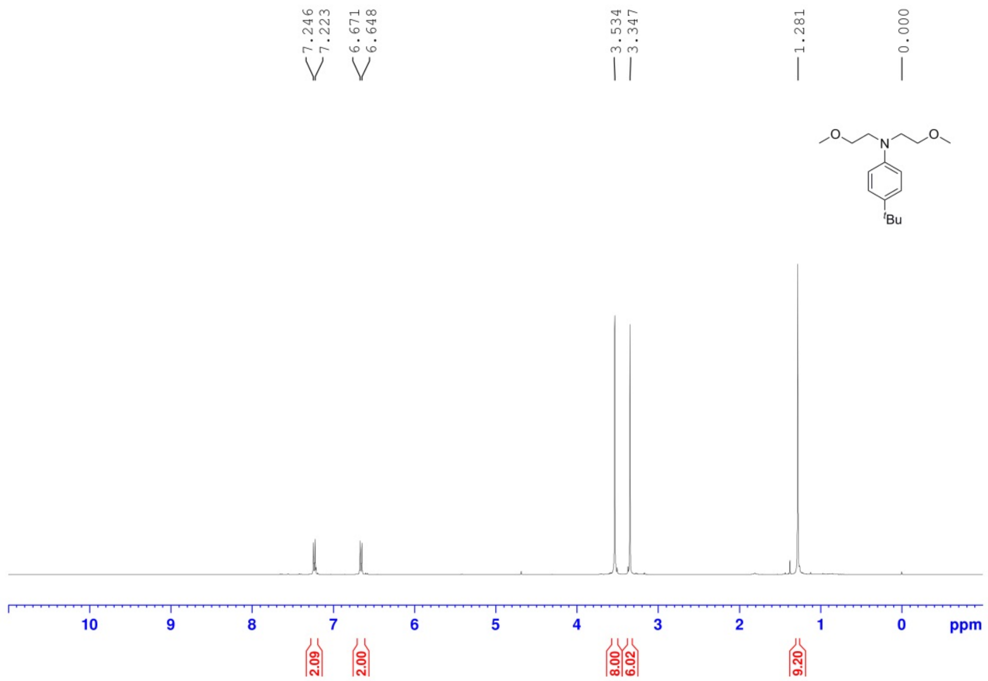

Supplementary Figure 117. ^{1}H NMR spectrum for 4-(*tert*-butyl)-*N*,*N*-bis(2-methoxyethyl)aniline (**7s**).

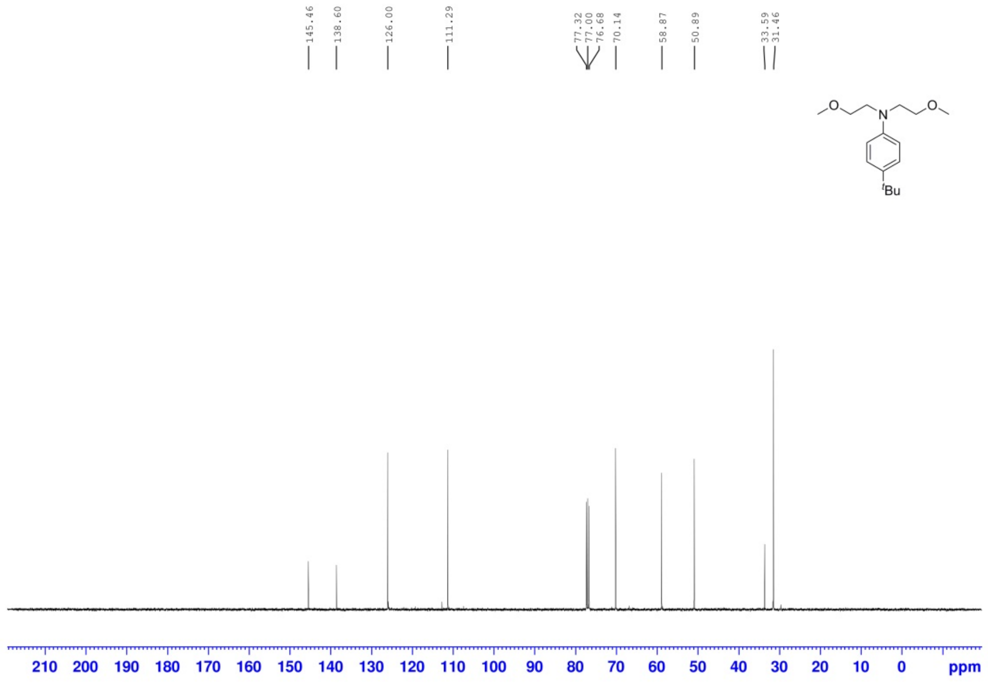

Supplementary Figure 118. ^{13}C NMR spectrum for 4-(*tert*-butyl)-*N*,*N*-bis(2-methoxyethyl)aniline (**7s**).

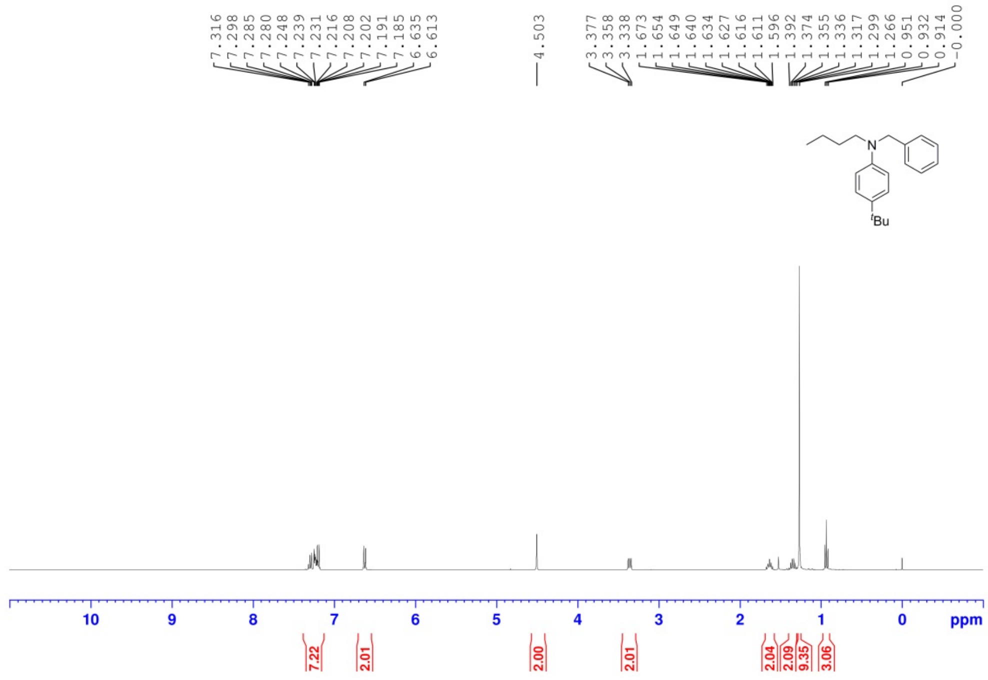

Supplementary Figure 119. ^{1}H NMR spectrum for *N*-benzyl-4-(tert-butyl)-*N*-butylaniline (**7t**).

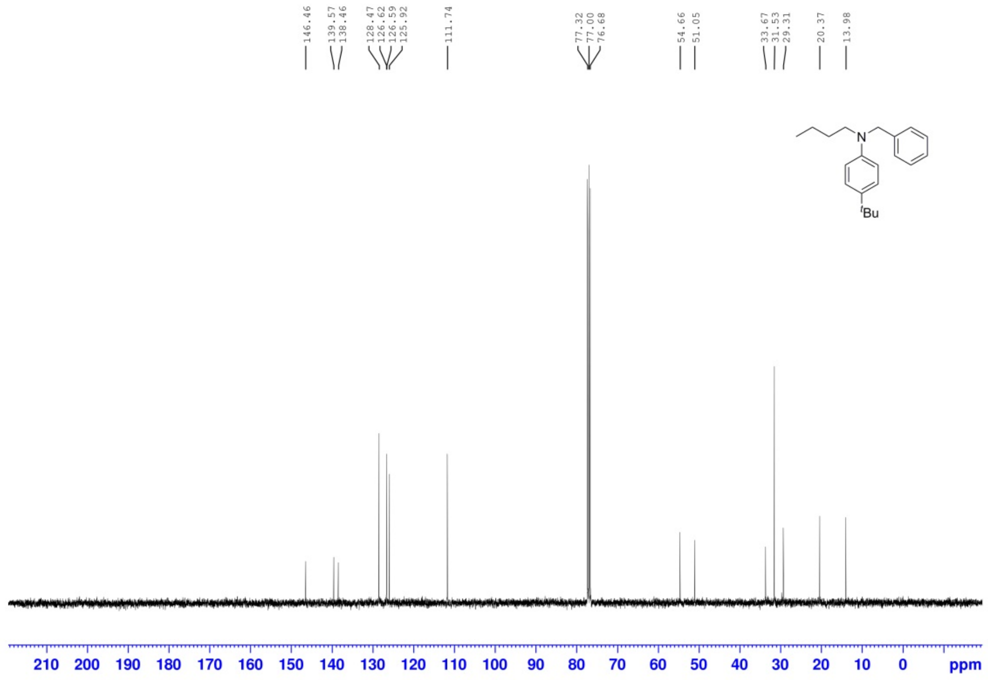

Supplementary Figure 120. ^{13}C NMR spectrum for *N*-benzyl-4-(tert-butyl)-*N*-butylaniline (**7t**).

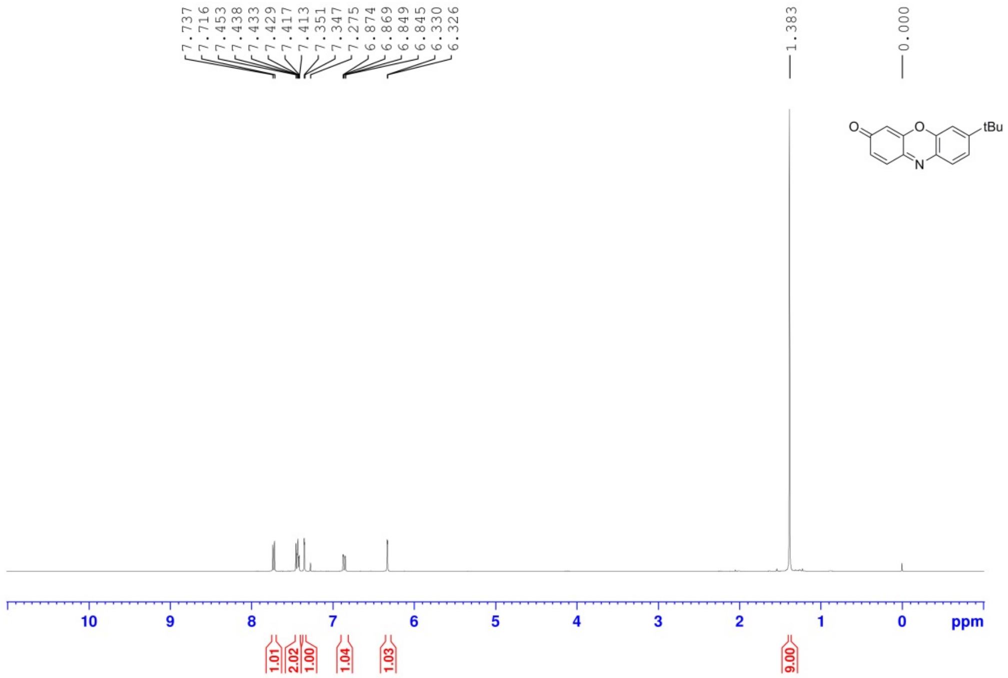

Supplementary Figure 121. ^{1}H NMR spectrum for 7-tert-butylphenoxazin-3-one (**7u**).

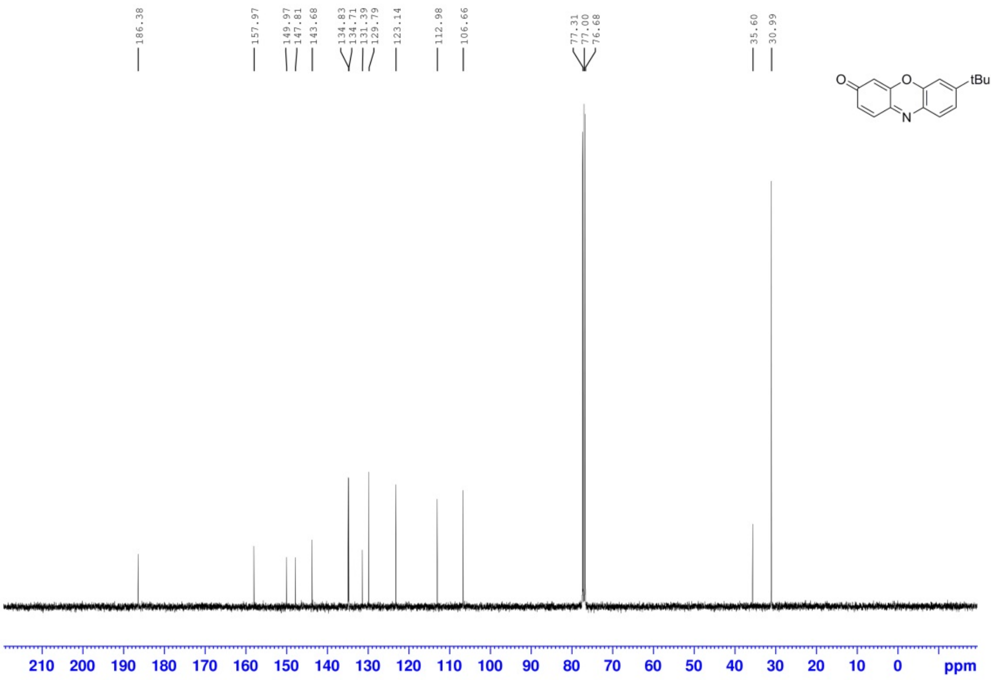

Supplementary Figure 122. ^{13}C NMR spectrum for 7-tert-butylphenoxazin-3-one (**7u**).

Supplementary References

1. Huang, Z. & Dong, G. Catalytic direct *β*-arylation of simple ketones with aryl iodides. *J. Am. Chem. Soc.* **135,** 17747-17750, (2013).
2. Jacobs, B. P., Wolczanski, P. T. & Lobkovsky, E. B. Oxidatively triggered carbon-carbon bond formation in ene-amide complexes. *Inorg. Chem.* **55,** 4223-4232, (2016).
3. Pan, J. et al. Development of resveratrol-curcumin hybrids as potential therapeutic agents for inflammatory lung diseases. *Eur. J. of Med. Chem.* **125,** 478-491, (2017).
4. Li, L., Yu, Z. & Shen, Z. Copper-catalyzed aminoxylation of different types of hydrocarbons with TEMPO: A concise route to *N*-alkoxyamine derivatives. *Adv. Synth. Catal.* **357,** 3495-3500, (2015).
5. Hayashi, M., Shibuya, M. & Iwabuchi, Y. Oxidative conversion of silyl enol ethers to *α,β*-unsaturated ketones employing oxoammonium salts. *Org. Lett.* **14,** 154-157, (2012).
6. Golubev, V. A. S., V. D.; Rozantsev, É. G. Synthesis and properties of alkali salts of 2,2,6,6-tetramethyl-1-hydroxypiperidine 1-oxide. *Russ. Chem. Bull.* **28,** 1927 (1979).
7. Morales, M. R., Momiyama, N. & Yamamoto, H. Metal-induced reactions of *O*-nitroso aldol products. *Synlett* **5**, 705-708, (2006).
8. Xiao, Q. et al. Transition-metal-free electrophilic amination of arylboroxines. *Org. Lett.* **14,** 4230-4233, (2012).
