# Supplementary material for: Differentiation between enamines and tautomerizable imines in the oxidation reaction with TEMPO
Source: Nat Commun. 2018 Nov 27;9:5002. doi: 10.1038/s41467-018-07534-x (PMC6258700; doi:10.1038/s41467-018-07534-x)
Supplement: Supplementary file 3 — Description of Additional Supplementary Files [file 41467_2018_7534_MOESM3_ESM.pdf]

Description of Additional Supplementary Files

File Name: Supplementary Data 1
Description: Computed Cartesian coordinates
