# Supplementary material for: Differentiation between enamines and tautomerizable imines in the oxidation reaction with TEMPO
Source: Nat Commun. 2018 Nov 27;9:5002. doi: 10.1038/s41467-018-07534-x (PMC6258700; doi:10.1038/s41467-018-07534-x)
Supplement: Supplementary file 4 — Supplementary Data 1 [file 41467_2018_7534_MOESM4_ESM.docx]

**A:**

C -3.25494300 0.25970700 0.91286100

C -1.80790900 0.39220200 0.47433500

C -1.22897800 1.59923200 0.34843600

C -1.91855600 2.90517800 0.65496600

C -3.43244300 2.75277300 0.79472800

C -3.75497000 1.51481400 1.62699400

H -0.19356300 1.67540400 0.03159700

H -3.89739100 0.06760800 0.04246900

H -3.34246300 -0.61586900 1.56690400

H -1.50662300 3.32912600 1.58339000

H -1.68479400 3.63340300 -0.13152800

H -3.86491700 3.65262900 1.24497600

H -3.88294900 2.63971500 -0.20019100

H -3.26576400 1.59815200 2.60621200

H -4.83191100 1.43329800 1.80860900

N -1.12914300 -0.83473100 0.29108000

C -1.81075400 -1.78930600 -0.59009100

C 0.29697700 -0.74649000 0.00644500

C -1.19603400 -3.18139900 -0.48518500

H -1.75786800 -1.44231800 -1.63941800

H -2.86623900 -1.83517900 -0.31567800

C 0.96949500 -2.11269100 0.11691100

H 0.47437400 -0.32352300 -1.00122200

H 0.74437600 -0.06069700 0.73132700

H -1.72047400 -3.85731100 -1.16939400

H -1.34796400 -3.55675100 0.53449600

H 2.03238000 -2.00700600 -0.12594400

H 0.90100200 -2.45286700 1.15757900

C 0.29742200 -3.13428200 -0.79655100

H 0.75336400 -4.12279700 -0.67722600

H 0.44002000 -2.83588600 -1.84458200

**B:**

C -3.50056300 0.89112500 -0.44768400

C -2.14178000 0.24418900 -0.29080400

C -1.11820400 1.14574400 0.37497100

C -1.65862600 1.66360300 1.71995900

C -3.01465600 2.35052500 1.55414500

C -4.02502900 1.40770200 0.90018600

H -0.15974000 0.64398100 0.52236300

H -3.40617500 1.74210200 -1.13805800

H -4.18429600 0.16550600 -0.89516300

H -1.76550700 0.81712700 2.41147300

H -0.93013500 2.34986200 2.16474900

H -3.38514900 2.69211700 2.52687800

H -2.89282400 3.24429300 0.92630500

H -4.20557400 0.55205500 1.56416100

H -4.98712200 1.91101700 0.75672100

H -0.94138500 2.00617600 -0.28597400

N -1.99641800 -0.95921900 -0.67945800

C -0.71852500 -1.64797000 -0.53699800

C -0.62922800 -2.29151500 0.85260200

C -0.61728300 -2.71924200 -1.62580800

H 0.13660900 -0.96349300 -0.65632900

C 0.64167500 -3.13020900 1.00514700

H -1.51682400 -2.92422900 0.99025400

H -0.67341300 -1.50948500 1.62133600

C 0.65951500 -3.54871600 -1.48126200

H -1.49869500 -3.36942900 -1.54384400

H -0.66548000 -2.24101000 -2.61078000

C 0.74232600 -4.19010300 -0.09424400

H 0.66307800 -3.60091200 1.99436600

H 1.51885800 -2.47022700 0.94937300

H 0.69974000 -4.31730800 -2.26094200

H 1.53484500 -2.90075000 -1.63099700

H 1.67347500 -4.75824100 0.01020600

H -0.08353500 -4.90587500 0.02008000

**C:**

C -3.39369400 1.01013200 -0.54015600

C -2.05017200 0.35551600 -0.33156400

C -1.09999100 1.16067200 0.52811800

C -1.75722600 1.50221800 1.87646900

C -3.10774900 2.19296600 1.67729700

C -4.04009000 1.34004400 0.81517900

H -0.15073500 0.63996500 0.66990000

H -3.24639900 1.94519900 -1.09889500

H -4.02434800 0.34870000 -1.13904100

H -1.90356400 0.57658600 2.44864900

H -1.08197800 2.13573100 2.46091800

H -3.57191400 2.39885800 2.64784100

H -2.94803500 3.16332100 1.18697700

H -4.26465200 0.40229500 1.34016000

H -4.99385700 1.85287700 0.65351400

H -0.89077500 2.09839400 -0.00589300

N -1.83752400 -0.78768600 -0.85485700

C -0.63213700 -1.48635300 -0.63783300

C 0.23232700 -1.71161300 -1.71444700

C -0.32800600 -2.04048600 0.61102200

C 1.39463500 -2.45364400 -1.53439000

H -0.02085500 -1.29590200 -2.68512500

C 0.83123000 -2.79200300 0.78044700

H -1.01507800 -1.88407600 1.43850600

C 1.70031300 -2.99862900 -0.28823300

H 2.06279900 -2.61289500 -2.37578000

H 1.05490800 -3.21735600 1.75454700

H 2.60404700 -3.58450500 -0.15369500

**LM1A:**

C -3.31022700 0.26860400 0.73702700

C -1.86326900 0.37763400 0.31902500

C -1.29894500 1.64944800 0.18288600

C -2.00020400 2.81598800 0.43413000

C -3.41824400 2.78668700 0.92147200

C -3.72081200 1.45663500 1.61508700

H -0.27252000 1.73841200 -0.16098500

H -3.96916800 0.22884000 -0.14412200

H -3.46227700 -0.66610800 1.28864400

H -1.52372200 3.77478500 0.25613100

H -3.60809200 3.62171600 1.60665200

H -4.11734800 2.92348500 0.07986500

H -3.15885100 1.40955800 2.55546100

H -4.78444100 1.38478400 1.86479500

N -1.15422900 -0.81222300 0.15202600

C -1.80106900 -1.85312400 -0.65412200

C 0.28088300 -0.72387900 -0.08767200

C -1.17527700 -3.22086500 -0.40311300

H -1.71223400 -1.59863000 -1.72627300

H -2.86563100 -1.87663400 -0.41412700

C 0.96210700 -2.06804200 0.16210700

H 0.48600200 -0.38666400 -1.12079900

H 0.69430900 0.02458800 0.59281100

H -1.66814400 -3.96364900 -1.03955900

H -1.35494700 -3.50527500 0.64110700

H 2.03010300 -1.97138500 -0.06088900

H 0.86832500 -2.31376000 1.22703200

C 0.32688000 -3.17687400 -0.67339800

H 0.79189600 -4.14318200 -0.45203800

H 0.49672100 -2.97259000 -1.73975100

**LM1B:**

C -3.54487400 0.27653200 0.52525400

C -2.30745800 -0.10357200 0.87873300

C -1.19456300 0.89439200 1.11166500

C -1.72708800 2.31746800 1.28516900

C -2.75824200 2.63986000 0.20566900

C -3.96534800 1.71094800 0.33889900

H -0.62875600 0.58586000 1.99894700

H -4.30639600 -0.49066900 0.38475500

H -2.20059500 2.40875200 2.27132400

H -0.89620000 3.03033600 1.25951600

H -3.07436400 3.68646200 0.27036000

H -2.29869500 2.49939700 -0.78176500

H -4.58776300 2.03824100 1.18564600

H -4.60282000 1.79544500 -0.54986000

H -0.48882600 0.86388300 0.26872900

N -1.97502700 -1.44717400 1.14225800

C -0.74722600 -1.98539900 0.55079800

C -0.52116700 -3.40459200 1.07205100

C -0.75402300 -1.97135200 -0.98331700

H 0.08765700 -1.36226600 0.89978500

C 0.76088600 -4.01484700 0.50069900

H -1.38126000 -4.02582300 0.77792700

H -0.49745400 -3.38739500 2.16687300

C 0.53270400 -2.57090100 -1.55517600

H -1.61904900 -2.55819000 -1.32776000

H -0.90398300 -0.94681300 -1.34527500

C 0.75574100 -3.99086600 -1.02975500

H 0.88395800 -5.03986500 0.86707700

H 1.62465000 -3.44240100 0.86640200

H 0.49922200 -2.56775100 -2.65021300

H 1.38462000 -1.94072700 -1.26287600

H 1.69499300 -4.39913600 -1.41914200

H -0.05102700 -4.64043500 -1.39733000

H -2.75983800 -2.05862000 0.92187800

**LM1C:**

C -3.56688900 0.33926900 0.49636300

C -2.32752400 -0.08357600 0.78549500

C -1.20203900 0.86688600 1.11171200

C -1.70522400 2.28692100 1.38137200

C -2.75111800 2.70544800 0.35176600

C -3.97076100 1.78952800 0.45007700

H -0.65958900 0.49800200 1.98815700

H -4.33530200 -0.40208900 0.27720200

H -2.15656500 2.32444200 2.38133600

H -0.85773100 2.98008400 1.38563900

H -3.04527400 3.74974200 0.50088900

H -2.31868000 2.62854900 -0.65437700

H -4.55734800 2.05342500 1.34300300

H -4.63633000 1.95625200 -0.40554300

H -0.47883400 0.86612100 0.28444000

N -2.03019400 -1.46743300 0.84032000

H -2.80279400 -2.00777500 0.46171000

C -0.78347600 -1.97933400 0.37913200

C 0.35740500 -1.91319500 1.18481900

C -0.69957800 -2.61340500 -0.86450600

C 1.56449100 -2.44013000 0.73607100

H 0.28345100 -1.47250100 2.17346000

C 0.50289900 -3.16418700 -1.29792300

H -1.58563500 -2.66580200 -1.49245000

C 1.64333000 -3.06980600 -0.50434000

H 2.44319500 -2.37832000 1.37122100

H 0.54944000 -3.65829500 -2.26390500

H 2.58367200 -3.49158500 -0.84506400

**LM2A:**

C -3.14900500 -0.55646900 1.30600900

C -2.11293600 0.16473800 0.46372700

C -2.26979600 1.45441500 0.11580800

C -3.41487100 2.31401700 0.57498100

C -4.56140000 1.50017000 1.15827100

C -4.01738900 0.42466400 2.09196700

H -1.52730900 1.96948800 -0.48134500

H -3.79627000 -1.16993500 0.66430600

H -2.63253800 -1.24432600 1.98532500

H -3.04204400 3.00315400 1.34649200

H -5.26335000 2.16600200 1.67070700

H -5.10116600 1.02968300 0.32615500

H -3.41715400 0.89181500 2.88327800

H -4.83713400 -0.11019600 2.58232000

N -0.96561000 -0.59673400 0.15281800

C -1.24205700 -1.90795800 -0.44559200

C 0.11037200 0.10550600 -0.53528400

C 0.00174800 -2.79066800 -0.45196900

H -1.60881400 -1.78000700 -1.48156600

H -2.03512600 -2.39831200 0.12174700

C 1.39007000 -0.72692000 -0.55260800

H -0.18251900 0.36077000 -1.57184700

H 0.29292500 1.04534500 -0.00728600

H -0.24213000 -3.74945100 -0.92225700

H 0.29365900 -2.99394900 0.58588900

H 2.16541700 -0.17586700 -1.09572300

H 1.73795500 -0.85446200 0.47994400

C -2.17336300 5.88687900 -2.03207800

C -2.57145900 5.60751600 -3.47687300

C -2.60354000 4.10069400 -3.70466500

C -3.52913500 3.36115600 -2.72607700

C -3.09586900 5.20203700 -1.01112500

H -2.92312900 3.86164700 -4.72609500

H -3.54943300 6.05122800 -3.69837800

H -1.85608800 6.07620800 -4.16144800

H -1.14993000 5.52328400 -1.86800900

H -2.17324600 6.96344900 -1.82311900

H -1.58723800 3.70335500 -3.58076700

N -3.12182900 3.74942500 -1.34401200

O -4.02887100 3.10127200 -0.46235400

C -3.28206200 1.85816200 -2.88375500

H -3.91975800 1.27836500 -2.21081700

H -3.50334400 1.56018000 -3.91459500

H -2.24004700 1.60724000 -2.66799300

C -5.00999200 3.62576700 -3.05291000

H -5.28913700 3.06104600 -3.94910100

H -5.64437000 3.28792700 -2.22916600

H -5.22755000 4.67626000 -3.25242900

C -2.45644400 5.34652300 0.37484700

H -2.17795400 6.39288100 0.54215100

H -3.15371000 5.06056000 1.16759800

H -1.55483600 4.73030300 0.45373100

C -4.47251200 5.88777800 -0.95712100

H -5.19583400 5.23719200 -0.45809300

H -4.39215000 6.81374000 -0.37670500

H -4.86264800 6.15683500 -1.94032400

C 1.14949600 -2.09708700 -1.18088800

H 2.05900900 -2.70605300 -1.14710700

H 0.88301300 -1.97097400 -2.23954200

**LM2B:**

C -3.64234700 0.45347900 0.28402300

C -2.31881600 -0.03281200 0.28373400

C -1.21332000 0.84039900 0.86661300

C -1.75229300 1.98048100 1.73417500

C -2.87046400 2.72873500 1.01335200

C -4.06082600 1.79361500 0.79362800

H -0.52598000 0.22043400 1.45153100

H -4.39656200 -0.21718400 -0.11722900

H -2.14293400 1.57132700 2.67543100

H -0.93377200 2.65859900 1.99638700

H -3.18458000 3.60884800 1.58322600

H -2.49958100 3.08575500 0.04382000

H -4.60153400 1.66083100 1.74509400

H -4.78972200 2.23959400 0.10505300

H -0.62305500 1.25774600 0.03896200

N -2.13048000 -1.22224600 -0.24587500

C -0.79460000 -1.78349500 -0.29784900

C -0.58137600 -2.70318600 0.91721400

C -0.64621100 -2.58832800 -1.59360600

H -0.00610700 -1.01303200 -0.28379300

C 0.77236800 -3.41360000 0.85208800

H -1.39462900 -3.44115000 0.92642700

H -0.67055600 -2.11925500 1.84131900

C 0.70889400 -3.29408100 -1.66568100

H -1.45819300 -3.32694700 -1.62696300

H -0.78649300 -1.92151500 -2.45199300

C 0.92254500 -4.20079200 -0.45157800

H 0.88753900 -4.07794100 1.71588700

H 1.57651300 -2.66713000 0.91686000

H 0.78220600 -3.87337700 -2.59277700

H 1.51017200 -2.54227500 -1.69897700

H 1.90883600 -4.67606600 -0.49743600

H 0.17732800 -5.00807500 -0.47106800

**LM2C:**

C -3.59352900 0.36230600 0.52789000

C -2.24149800 -0.00505000 0.41594700

C -1.17578600 1.06049600 0.63008300

C -1.70586200 2.23405100 1.45695100

C -2.97905400 2.78910800 0.82250700

C -4.08590100 1.73281600 0.85480400

H -0.29979400 0.60438400 1.10303300

H -4.31844000 -0.43669800 0.40200700

H -1.92104800 1.90021800 2.48052600

H -0.93626600 3.00937400 1.52620900

H -3.31393300 3.69509700 1.33693100

H -2.76620100 3.06885400 -0.21718100

H -4.53578500 1.70122900 1.86040700

H -4.90907900 2.00179500 0.18025700

H -0.83948500 1.44481500 -0.34109300

N -2.01092100 -1.28667600 0.18600100

C -0.72915400 -1.79938400 -0.01454200

C 0.23224600 -1.22478800 -0.86389000

C -0.42842400 -3.02702200 0.60065300

C 1.45892200 -1.84916100 -1.06494500

H -0.00216900 -0.30895500 -1.39619700

C 0.80827300 -3.63035000 0.41739400

H -1.18709500 -3.48251400 1.22975300

C 1.76037700 -3.04509400 -0.41745900

H 2.18395100 -1.39632900 -1.73510600

H 1.02647100 -4.56978500 0.91683600

H 2.72205200 -3.52415800 -0.57226700

**LM3B:**

C -3.67357500 -0.05591000 1.05951300

C -2.26898600 -0.60658700 0.85185900

C -1.73303500 -0.15703800 -0.49973600

C -1.60162100 1.37625500 -0.49416000

C -2.94541500 2.03335400 -0.17180000

C -3.55498200 1.47587800 1.11669600

H -0.78142200 -0.63231500 -0.74287900

H -4.24635400 -0.30227500 0.15879200

H -0.86061400 1.66350300 0.26319900

H -1.22705900 1.72511300 -1.46234300

H -2.82689500 3.11872200 -0.08786300

H -3.63996900 1.85320500 -1.00351500

H -2.93276200 1.73583000 1.98262900

H -4.55231600 1.89414900 1.28870900

H -2.44850100 -0.45318600 -1.27814200

N -1.65071000 -1.22722200 1.76699400

C -6.16259800 -3.68622400 2.27137100

C -6.20815200 -3.84704400 3.78331400

C -4.89398000 -3.34400500 4.36204700

C -4.57901000 -1.89259100 3.97060900

C -5.88416100 -2.24434400 1.80608400

H -4.89114400 -3.40668800 5.45679400

H -7.05613000 -3.29567900 4.20670400

H -6.36052600 -4.90011600 4.04428800

H -5.36894100 -4.33128000 1.87059900

H -7.10407700 -4.00684500 1.80949200

H -4.07835900 -3.98235900 3.99742400

N -4.62201800 -1.79510600 2.47557900

O -4.40591600 -0.40161500 2.21729200

C -3.14915600 -1.59539100 4.42537400

H -2.86356400 -0.56545700 4.20083100

H -3.07840000 -1.75565000 5.50753200

H -2.44616800 -2.25112400 3.91216900

C -5.51797400 -0.91565800 4.70473700

H -5.21812200 -0.85841300 5.75679700

H -5.43834100 0.08667100 4.27728100

H -6.56579900 -1.21988800 4.68552100

C -5.63715000 -2.32919900 0.28914000

H -6.35165500 -3.03297500 -0.15250500

H -5.79003900 -1.37054800 -0.21303500

H -4.62421600 -2.68994500 0.08280100

C -7.10955300 -1.34335500 2.02981600

H -6.82094500 -0.29056400 1.95996000

H -7.85307100 -1.54483100 1.25034500

H -7.59775000 -1.50677400 2.99197200

C -0.27253900 -1.66347600 1.58148200

C 0.44216600 -1.63136500 2.93536000

C -0.25864300 -3.08413900 1.00457600

H 0.28757400 -1.00703300 0.89602500

C 1.87287600 -2.16018900 2.82165100

H -0.12656200 -2.25188300 3.64042300

H 0.42857500 -0.60852100 3.32954100

C 1.16614200 -3.63299800 0.89955300

H -0.86428100 -3.72279200 1.66238500

H -0.74930700 -3.08363500 0.02323700

C 1.88481600 -3.57987500 2.24983100

H 2.36139500 -2.13868800 3.80198100

H 2.45614800 -1.50105100 2.16300100

H 1.14451700 -4.65963500 0.51736000

H 1.73002100 -3.03537600 0.16943300

H 2.91354100 -3.94286500 2.14648800

H 1.37720000 -4.25383700 2.95376200

**LM3C:**

C -3.72282100 0.04308000 1.00545300

C -2.33542700 -0.53691200 0.79537600

C -1.71268500 -0.02888700 -0.49256400

C -1.57788300 1.50216200 -0.41795500

C -2.93073200 2.15798000 -0.14120200

C -3.58508700 1.57065000 1.10961700

H -0.74078900 -0.49740900 -0.65912300

H -4.28361100 -0.17043800 0.08545000

H -0.87523900 1.75351800 0.38698700

H -1.14798600 1.87982600 -1.35127000

H -2.81265900 3.24029100 -0.02519100

H -3.59226100 2.00028700 -1.00377200

H -2.98462800 1.79772300 1.99982800

H -4.58267400 1.99245500 1.26868300

H -2.36310600 -0.29378100 -1.33652700

N -1.76893200 -1.26235900 1.66803400

C -5.89373800 -3.75829600 2.31470400

C -5.93713400 -3.86766300 3.83256100

C -4.69485700 -3.20150900 4.40858000

C -4.53501000 -1.73983300 3.96435900

C -5.76995400 -2.31102500 1.80466200

H -4.70509000 -3.22391900 5.50487100

H -6.84666900 -3.39966300 4.22776200

H -5.97498900 -4.92096800 4.13131000

H -5.02925400 -4.32462100 1.94300100

H -6.78983200 -4.19736700 1.85976400

H -3.80787400 -3.75803400 4.07927300

N -4.57313400 -1.71415600 2.46907100

O -4.48248900 -0.32430500 2.13923500

C -3.15341800 -1.26230500 4.42062200

H -2.98454100 -0.21832400 4.14319100

H -3.09154100 -1.34575400 5.51157200

H -2.36593500 -1.86467300 3.96743200

C -5.58641200 -0.84262800 4.64644400

H -5.31226600 -0.70823500 5.69837900

H -5.61061100 0.14382000 4.17653400

H -6.59404000 -1.26107100 4.62790800

C -5.48862200 -2.40004900 0.29572300

H -6.13440200 -3.16566600 -0.14854500

H -5.70812500 -1.46216300 -0.22121100

H -4.44535400 -2.67898700 0.11385800

C -7.08857500 -1.54103900 1.98793700

H -6.91175400 -0.46504300 1.90084000

H -7.79110700 -1.83463400 1.19976200

H -7.57611000 -1.73774900 2.94433300

C -0.46768800 -1.76455900 1.48294100

C 0.58710900 -1.22427200 2.22723000

C -0.22608500 -2.85848200 0.64375400

C 1.86746300 -1.75491500 2.11002400

H 0.38559800 -0.38868000 2.89147800

C 1.05679100 -3.38777200 0.53953900

H -1.05401500 -3.28693500 0.08562000

C 2.11005800 -2.83852900 1.26755900

H 2.68005300 -1.32120100 2.68569700

H 1.23274300 -4.23559600 -0.11631800

H 3.10922800 -3.25398600 1.18369500

**LM4B:**

C -3.59737300 0.15681000 1.24825600

C -2.30839700 -0.23901600 1.21887000

C -1.29313900 0.56776400 0.42684400

C -1.79144400 1.94162900 -0.01167400

C -3.19157600 1.82824900 -0.59900100

C -4.14434500 1.33431500 0.48213200

H -0.40469100 0.68876700 1.05846000

H -1.82113700 2.61324600 0.85607500

H -1.09056700 2.37502200 -0.73295400

H -3.53664300 2.78907600 -0.99472600

H -3.17942100 1.11564400 -1.43386800

H -4.37407800 2.14991800 1.18401000

H -5.10425600 1.03271000 0.04459300

H -0.96612300 -0.00311400 -0.45462700

N -1.74373200 -1.29207800 1.97274300

C -5.03012300 -3.84905600 3.00606100

C -5.20389800 -3.62185200 4.50652800

C -4.32731300 -2.45914800 4.96789300

C -4.58761500 -1.16352500 4.18356000

C -5.31106800 -2.58641000 2.17747800

H -4.47744000 -2.25449600 6.03458700

H -6.25617700 -3.41992700 4.73977500

H -4.93574000 -4.53138300 5.05474900

H -3.99628000 -4.16712100 2.80860300

H -5.68811100 -4.65120200 2.65119500

H -3.27075300 -2.73227200 4.83652600

N -4.44497800 -1.51955800 2.74545000

O -4.67031400 -0.34549300 1.98232300

C -3.48682200 -0.14866300 4.51070900

H -3.68746200 0.81319100 4.02857200

H -3.45535300 0.01478300 5.59338400

H -2.50705000 -0.50389600 4.17857700

C -5.93652100 -0.53043100 4.56433300

H -5.83319200 -0.02089200 5.52835600

H -6.22668800 0.21645200 3.81982000

H -6.74526700 -1.25603700 4.66652500

C -4.84589600 -2.80945700 0.73562900

H -5.29543900 -3.72600100 0.33871100

H -5.14527400 -1.97608800 0.09296400

H -3.75793900 -2.90464800 0.68788100

C -6.81392900 -2.26117000 2.13537000

H -6.96932800 -1.23300500 1.79648800

H -7.30343700 -2.92919200 1.41830800

H -7.31529200 -2.39385800 3.09535200

H -2.47679000 -1.87083000 2.37676300

C -0.64010100 -2.07944200 1.43269400

C -0.07264100 -2.95841200 2.54949800

C -0.99815900 -2.94985600 0.21930500

H 0.15602600 -1.38840500 1.12576500

C 1.13746000 -3.76381700 2.06907600

H -0.86209600 -3.64802600 2.88770000

H 0.18971700 -2.32716200 3.40540900

C 0.22310900 -3.72354500 -0.28420000

H -1.77324000 -3.66763400 0.52532700

H -1.43170800 -2.32915900 -0.57483400

C 0.80348500 -4.59833000 0.82979800

H 1.50505300 -4.40830200 2.87525900

H 1.95262500 -3.06877600 1.82407500

H -0.04460900 -4.33665200 -1.15186400

H 0.98888700 -3.01141500 -0.62318700

H 1.69620300 -5.12663900 0.47674500

H 0.06535800 -5.36618700 1.10108800

**LM4C:**

C -3.60463400 0.15709500 1.16098200

C -2.28996800 -0.13103700 1.13852800

C -1.30431500 0.82815100 0.48914100

C -1.88622200 2.22262000 0.27367500

C -3.24641700 2.10873400 -0.40548000

C -4.21477900 1.37371600 0.51536200

H -0.42377100 0.87377000 1.14047700

H -2.00597800 2.72731900 1.24134200

H -1.19606500 2.82538300 -0.32567600

H -3.65096700 3.09401500 -0.65826900

H -3.13479600 1.55234100 -1.34498200

H -4.56809600 2.04709900 1.31040900

H -5.11071600 1.05538700 -0.03138900

H -0.95038800 0.44007500 -0.47380100

N -1.69666200 -1.19686400 1.85419900

C -4.60605100 -3.85370400 3.32895600

C -4.96720100 -3.42997300 4.75020100

C -4.28030300 -2.10838000 5.08415800

C -4.61730600 -0.98745600 4.08816600

C -4.94937700 -2.78595200 2.27891200

H -4.55080200 -1.76520700 6.08960700

H -6.05416400 -3.33411400 4.85693200

H -4.65361400 -4.20234400 5.46041700

H -3.52591300 -4.05089000 3.27853000

H -5.11621500 -4.78369300 3.05211500

H -3.19180200 -2.26078400 5.07686100

N -4.30404800 -1.52293000 2.73342000

O -4.64235200 -0.51954700 1.78222900

C -3.66975800 0.19276900 4.33262800

H -3.93727300 1.05240100 3.70997700

H -3.73805400 0.50291600 5.38074500

H -2.63098100 -0.08085100 4.12024600

C -6.05831500 -0.48488500 4.26909200

H -6.10332100 0.16082300 5.15263500

H -6.36505200 0.10805500 3.40291200

H -6.78170000 -1.28857000 4.41712900

C -4.29555400 -3.16945800 0.94743300

H -4.68205700 -4.13921900 0.61710000

H -4.51861600 -2.43159800 0.17075100

H -3.20947600 -3.25360700 1.04535800

C -6.46675300 -2.68304000 2.05404100

H -6.70955400 -1.77500700 1.49543700

H -6.79940200 -3.54209200 1.46198700

H -7.04145100 -2.68565900 2.98158900

H -2.36395800 -1.62794100 2.49305700

C -0.82374500 -2.11551900 1.25058300

C -0.09175300 -2.97358700 2.08700100

C -0.67634500 -2.24489800 -0.13681400

C 0.77186700 -3.91878600 1.55061000

H -0.20635600 -2.87278800 3.16343200

C 0.21079300 -3.18000200 -0.66496600

H -1.28713900 -1.63866500 -0.79773100

C 0.94128500 -4.02085800 0.16929400

H 1.32755000 -4.57274300 2.21671800

H 0.31319500 -3.26254500 -1.74342200

H 1.62730600 -4.75070200 -0.24844800

**LM5B:**

C -3.33109400 0.33075400 1.50272800

C -2.07832900 -0.34546000 0.94560900

C -1.82856800 -0.14488100 -0.53756500

C -2.08215400 1.30197800 -0.98032800

C -3.48536500 1.75766700 -0.58759200

C -3.66896500 1.69397800 0.93244400

H -0.82138700 -0.46575600 -0.81479200

H -1.33894700 1.96303000 -0.51639900

H -1.94511100 1.37630100 -2.06382700

H -3.67030800 2.77862700 -0.93575800

H -4.22826400 1.11392200 -1.07578500

H -2.97716300 2.40863900 1.40287100

H -4.68229100 1.95652900 1.24686800

H -2.52809900 -0.80208200 -1.07503900

N -1.40353600 -1.01745000 1.78654600

O -3.99169000 -0.19450100 2.36874100

C -0.20099600 -1.73670200 1.38743800

C 0.71188800 -1.89274500 2.60563600

C -0.60034600 -3.11282700 0.83573300

H 0.35859000 -1.20136600 0.60388900

C 1.94755000 -2.73026400 2.27179000

H 0.13270500 -2.37722200 3.40266100

H 0.99764700 -0.90170900 2.97594000

C 0.63131600 -3.95947500 0.50747900

H -1.21452500 -3.61732000 1.59342500

H -1.22972600 -2.98344500 -0.05317900

C 1.54851100 -4.10235000 1.72419500

H 2.57656100 -2.84136100 3.16161200

H 2.55342500 -2.20443100 1.52015200

H 0.31847200 -4.94483400 0.14504800

H 1.18980300 -3.48295800 -0.31043600

H 2.43933100 -4.68353700 1.46140200

H 1.02064000 -4.66220100 2.50841700

**LM5C:**

C -3.15868300 0.48957000 1.60673600

C -1.98789600 -0.25811400 0.96612200

C -1.89754000 -0.22335600 -0.54556500

C -2.25744700 1.15619700 -1.11231200

C -3.62684600 1.61463600 -0.61824500

C -3.62916300 1.74770300 0.90765400

H -0.90057400 -0.53637100 -0.86733900

H -1.49578500 1.88681700 -0.81182400

H -2.23958800 1.10701000 -2.20547900

H -3.89766700 2.57464300 -1.06826800

H -4.39016200 0.88946300 -0.92809100

H -2.92465500 2.54266000 1.19477500

H -4.60961700 2.01546600 1.30960600

H -2.60951800 -0.96035300 -0.94453800

N -1.19545400 -0.82268200 1.78768000

O -3.65233100 0.08974100 2.63513200

C -0.09742800 -1.59337600 1.35524400

C 1.16710100 -1.28908500 1.87100400

C -0.24941300 -2.70077800 0.51296600

C 2.26945900 -2.05535300 1.51152400

H 1.26535100 -0.44672100 2.54881900

C 0.85542900 -3.47860500 0.17878900

H -1.23874300 -2.95868700 0.14559400

C 2.11883000 -3.15435000 0.66666100

H 3.24924000 -1.80226800 1.90543200

H 0.72579700 -4.34134600 -0.46787200

H 2.97915100 -3.76013100 0.40010300

**ProA:**

C -1.42437800 -2.61731400 -0.48163100

C -1.41883900 -1.42564200 0.46068300

C -2.53103700 -0.65753700 0.53176600

C -3.72812100 -1.00910900 -0.23261900

C -3.88362300 -2.22047700 -0.78214900

C -2.81070800 -3.25812100 -0.57847100

H -2.55409300 0.24849100 1.12629400

H -1.10700100 -2.28420800 -1.48096800

H -0.70046700 -3.36400500 -0.14269100

H -4.50597400 -0.25518000 -0.32699800

H -4.78915300 -2.48518200 -1.32056200

H -3.02156200 -3.81335000 0.34924300

H -2.81364400 -3.99418200 -1.38837900

N -0.25041000 -1.20954900 1.19799800

C 1.00241000 -1.23667900 0.43420000

C -0.28598100 -0.12710200 2.17384400

C 2.20906300 -1.36897700 1.35714100

H 1.09538200 -0.31027900 -0.16167000

H 0.97548500 -2.07185200 -0.26806700

C 0.89634900 -0.21615500 3.13564500

H -0.28634400 0.85617500 1.66692700

H -1.22212500 -0.20879300 2.73279300

H 3.12353300 -1.36633400 0.75429900

H 2.15363800 -2.33501600 1.87403300

H 0.85314800 0.62856200 3.83144800

H 0.79714000 -1.13517000 3.72618500

C 2.22405400 -0.23702800 2.38190300

H 3.06249800 -0.35080100 3.07691000

H 2.36286900 0.72059400 1.86103500

**ProAO:**

C -1.54828200 -1.90824000 0.19523400

C -1.15762500 -0.51436900 -0.25538500

C -2.11890200 0.43273700 -0.45647200

C -3.54569500 0.17532800 -0.29347900

C -3.96312400 -1.22399300 0.12666300

C -2.87792000 -1.90786300 0.94804800

H -1.87934800 1.42844000 -0.80951700

H -1.61919000 -2.57125700 -0.67843400

H -0.76085800 -2.30972800 0.84037700

H -4.91183900 -1.15041300 0.66484500

H -4.15455800 -1.79701400 -0.79199600

H -2.74900600 -1.37559600 1.89784300

H -3.16331200 -2.93657800 1.18885400

N 0.19243000 -0.27010000 -0.39019600

C 1.04905300 -1.31971600 -0.95566300

C 0.63160500 1.08090800 -0.73159700

C 2.50377700 -1.14281100 -0.53554200

H 0.98134900 -1.28430400 -2.05663000

H 0.68448600 -2.29647700 -0.63975900

C 2.09070400 1.30386500 -0.33743400

H 0.49198500 1.26658200 -1.81045900

H 0.00565600 1.79062100 -0.18828500

H 3.10453900 -1.93171900 -1.00026100

H 2.57713400 -1.26524800 0.55215700

H 2.38870500 2.30766900 -0.65754800

H 2.16347700 1.27331000 0.75664100

O -4.38499100 1.03338000 -0.53002600

C 3.00821600 0.24116400 -0.93543800

H 4.04039800 0.39125100 -0.60320800

H 3.00429300 0.32607100 -2.03088600

**ProB:**

C -3.00213600 -0.79629800 1.71309800

C -1.66310600 -0.92210300 1.10554000

C -1.25977600 0.18932800 0.14933500

C -1.89854300 1.52553300 0.53762600

C -3.41924900 1.41190200 0.62652400

C -3.81890000 0.23633400 1.47441900

H -0.17224200 0.29204900 0.11427100

H -3.30566200 -1.62670300 2.34486100

H -1.50960900 1.83459400 1.51546600

H -1.61233900 2.29734500 -0.18365300

H -3.84898800 2.33110700 1.03971200

H -3.85008900 1.30024200 -0.37956800

H -4.81628500 0.24018200 1.90924900

H -1.58771100 -0.09399200 -0.86159300

N -0.98877700 -1.96712500 1.39974800

C 0.32461700 -2.19091000 0.81411900

C 1.40434900 -1.64498000 1.75909200

C 0.51781800 -3.69560300 0.60423600

H 0.43771800 -1.69524000 -0.16355900

C 2.81096600 -1.96213400 1.24679100

H 1.25086000 -2.10109800 2.74645000

H 1.26956600 -0.56341800 1.88387600

C 1.92004000 -4.01340600 0.08243100

H 0.35305600 -4.19506000 1.56827100

H -0.25024000 -4.06803600 -0.08318500

C 2.99408400 -3.46520100 1.02478000

H 3.55971900 -1.58490100 1.95218800

H 2.97701700 -1.43543700 0.29645300

H 2.03794500 -5.09531700 -0.04350000

H 2.05006900 -3.56360100 -0.91212200

H 3.99401600 -3.67161600 0.62701200

H 2.92119700 -3.98245900 1.99149700

**ProBO:**

C -3.02312800 0.62301800 1.64420700

C -1.75681200 0.06696900 1.06732400

C -1.41343300 0.34968200 -0.20819500

C -2.24087400 1.21223300 -1.12293600

C -3.70198900 1.27091000 -0.68278500

C -3.78835100 1.61693500 0.80227300

H -0.48192100 -0.04483600 -0.60775800

H -1.82768600 2.23276100 -1.15243700

H -2.17102500 0.83027600 -2.14750300

H -4.25544400 2.00629500 -1.27481000

H -4.16956400 0.29363600 -0.85366100

H -3.33565600 2.60429300 0.97683800

H -4.81623300 1.65511900 1.17182000

N -1.02805800 -0.67484000 1.97689400

O -3.38554100 0.29322200 2.75861900

C -0.00727100 -1.61969600 1.56037400

C 0.74851600 -2.12079500 2.79182200

C -0.56688600 -2.80392900 0.75850400

H 0.70975200 -1.07565200 0.92739400

C 1.85504800 -3.10535700 2.40579600

H 0.03136600 -2.62084200 3.46032000

H 1.15701100 -1.26497800 3.33988500

C 0.54371400 -3.77762900 0.35855600

H -1.30280900 -3.32191000 1.39037200

H -1.10302600 -2.43921700 -0.12441400

C 1.30297400 -4.27674100 1.58976800

H 2.36038600 -3.47048800 3.30643800

H 2.61255600 -2.57719900 1.81023500

H 0.12419000 -4.62074100 -0.20111800

H 1.24585100 -3.26810700 -0.31661900

H 2.11636300 -4.94771700 1.29200900

H 0.61944400 -4.86364300 2.21903100

H -1.59071200 -0.96203900 2.77204600

**ProCO:**

C -3.13964900 0.40834400 1.47058100

C -1.89822100 -0.12657500 0.81928200

C -1.43844500 0.42993400 -0.32086100

C -2.10297800 1.59830500 -0.99805800

C -3.57674300 1.72337800 -0.62042700

C -3.73360700 1.67197800 0.89762500

H -0.55317700 0.02356200 -0.79866100

H -1.57619300 2.52915300 -0.73641200

H -1.99796200 1.48899000 -2.08273100

H -3.99915000 2.65358800 -1.01218100

H -4.13602300 0.89428900 -1.07008800

H -3.19397400 2.51547600 1.35309100

H -4.77559000 1.73384900 1.22119300

N -1.38980100 -1.21030800 1.51431400

O -3.61539000 -0.15988800 2.43620000

H -2.03920000 -1.57931700 2.19976500

C -0.20913700 -1.92119300 1.31663100

C 0.96017600 -1.33302300 0.81443100

C -0.17532300 -3.27221800 1.69558100

C 2.11806600 -2.09261200 0.67317100

H 0.97642500 -0.27654000 0.57409200

C 0.98942200 -4.01519700 1.56225200

H -1.07808800 -3.73086000 2.09080000

C 2.14430000 -3.43510200 1.03878700

H 3.01420500 -1.61803100 0.28387400

H 0.99087400 -5.05913200 1.86179100

H 3.05143600 -4.01952600 0.92514900

**TEMPH:**

C -3.32002400 0.68124000 -0.00199600

C -1.79246500 0.64850600 0.03764800

C -1.24008700 2.07258700 -0.01608100

C -1.79039800 2.95775000 1.11192700

C -3.91835800 1.53583200 1.12516400

H -0.14433500 2.07064500 0.03105100

H -1.44436500 0.13744600 0.94403000

H -1.41002100 0.06792000 -0.80907500

H -3.63673500 1.11101800 -0.96159700

H -3.73597000 -0.33192000 0.05627400

H -1.52646700 2.52410000 -0.97521100

N -3.25836500 2.85109800 1.09744500

C -1.47198400 4.42429500 0.80722200

H -1.82989900 5.07484500 1.61515800

H -0.39200700 4.57837900 0.70743700

H -1.96198100 4.73253900 -0.12081300

C -1.12746300 2.60103100 2.45618500

H -0.08140600 2.92870200 2.46143200

H -1.64010300 3.10984100 3.28131600

H -1.13410500 1.52917000 2.66562000

C -5.39732200 1.80092800 0.82946100

H -5.95376000 0.86148400 0.74062200

H -5.85540200 2.38658300 1.63647200

H -5.50106700 2.36474800 -0.10209400

C -3.83213500 0.79637600 2.47402100

H -4.10250600 1.46989400 3.29608700

H -4.53282100 -0.04651600 2.48985700

H -2.83679400 0.39667600 2.68017700

H -3.64096100 3.43033900 1.84533900

**TEMPO:**

C -3.31228800 0.68734500 0.01304600

C -1.78902100 0.64403900 0.06783100

C -1.24871500 2.06827100 -0.00158900

C -1.74561300 2.95817100 1.14459800

C -3.93555700 1.49506700 1.15850300

H -0.15271000 2.07888600 0.01392800

H -1.44763100 0.14356400 0.98198000

H -1.40261000 0.05743800 -0.77206400

H -3.61637400 1.13983000 -0.94033800

H -3.73978100 -0.32164500 0.04014100

H -1.56126500 2.51634500 -0.95433300

N -3.21770800 2.79253400 1.31425000

O -3.78709400 3.65184100 2.06502500

C -1.49565500 4.42682800 0.80128900

H -1.77266500 5.07580000 1.63377800

H -0.43390700 4.57068300 0.57631900

H -2.08076300 4.71955200 -0.07610200

C -1.04412000 2.62100400 2.46746100

H 0.00104300 2.94545300 2.42548600

H -1.53996400 3.14864700 3.28744900

H -1.05471200 1.54938800 2.68466600

C -5.39052400 1.82351700 0.82275700

H -5.93101200 0.89593200 0.60801400

H -5.87856900 2.33368500 1.65487200

H -5.44315500 2.46978600 -0.05898200

C -3.88363600 0.72863700 2.48719600

H -4.18436700 1.39314600 3.30243700

H -4.57697300 -0.11845800 2.45378600

H -2.88599200 0.33817100 2.70575300

**TEMPO(NH):**

C -3.32177900 0.69031400 -0.00546800

C -1.79554400 0.65291800 0.00718500

C -1.24583300 2.07698300 -0.01754200

C -1.73619700 2.93707100 1.15214300

C -3.91920300 1.47784900 1.16542100

H -0.14970000 2.07829000 -0.00181100

H -1.43210700 0.11669900 0.89114200

H -1.43099400 0.09947300 -0.86426400

H -3.65748500 1.14770200 -0.94789300

H -3.74261000 -0.32165200 0.01796700

H -1.54629700 2.55647700 -0.96093900

N -3.27136600 2.87141600 1.22050000

O -3.76107600 3.61343400 2.23777800

C -1.42647400 4.41070000 0.89943700

H -1.77651500 5.01257300 1.73922400

H -0.34770900 4.54402100 0.77337900

H -1.91863600 4.76684700 -0.01434300

C -1.13924300 2.52544200 2.49245900

H -0.13271200 2.94693200 2.57708800

H -1.76335700 2.93692100 3.28913200

H -1.05411400 1.44397200 2.61411800

C -5.40143900 1.75313600 0.92362800

H -5.93891600 0.80645800 0.81312000

H -5.81511600 2.31648400 1.76115300

H -5.55036100 2.33354200 0.00452500

C -3.75622100 0.77629500 2.50838700

H -3.89912800 1.51307800 3.30220400

H -4.52516300 0.00281700 2.60017000

H -2.78577200 0.29102700 2.62855200

H -3.55512300 3.28852400 0.31466800

**TEMPOH:**

C -3.31384800 0.69779200 0.01179300

C -1.78877900 0.64977000 0.04732900

C -1.23229500 2.06882600 -0.02957700

C -1.77009700 2.98108000 1.08346600

C -3.90932000 1.57292300 1.12532600

H -0.13704400 2.06737200 0.02390300

H -1.44211100 0.15094200 0.96030600

H -1.41348500 0.05444400 -0.79212800

H -3.63247800 1.10762300 -0.95593500

H -3.74291400 -0.30779900 0.09625500

H -1.51071700 2.50610000 -0.99769200

N -3.24959800 2.89759700 1.03760700

O -3.76149500 3.70668100 2.09567200

C -1.41099400 4.43281700 0.74911800

H -1.69462500 5.10425000 1.56460300

H -0.33170500 4.52798500 0.58944800

H -1.92703300 4.74991400 -0.16313800

C -1.14524200 2.63256900 2.44436200

H -0.11833500 3.01278600 2.48173700

H -1.71078600 3.10638000 3.25149000

H -1.10085300 1.55813100 2.63372200

C -5.39334100 1.81073400 0.82614100

H -5.91107300 0.85404200 0.69919600

H -5.87618000 2.35247100 1.64449700

H -5.50528600 2.39212700 -0.09503000

C -3.79945000 0.88636800 2.49677600

H -3.99238700 1.60728700 3.29609200

H -4.55094800 0.09222900 2.56812100

H -2.82401200 0.42705500 2.66977400

H -4.23406900 4.40849300 1.62411700

**TS1A-beta:**

C -2.87903300 -0.06025400 1.56227700

C -1.88085600 0.42784300 0.53093000

C -1.85661300 1.74418100 0.16955200

C -2.68402900 2.76121300 0.79494900

C -3.83757200 2.26324000 1.64111100

C -3.36800600 1.06973900 2.47040600

H -1.16556600 2.07922700 -0.59726300

H -3.74295500 -0.51676400 1.05816500

H -2.41254700 -0.84933900 2.16309200

H -1.94137600 3.37046500 1.71673100

H -2.91514400 3.61341400 0.14923400

H -4.20245100 3.06471000 2.29341100

H -4.67964400 1.95894600 1.00084300

H -2.54602600 1.39558200 3.12018700

H -4.17046000 0.69981800 3.11709000

N -0.99178700 -0.52805300 0.03827300

C -1.55644700 -1.82481800 -0.34978600

C 0.06734200 -0.07503300 -0.85501800

C -0.47885000 -2.90005100 -0.43858400

H -2.06173100 -1.73066500 -1.32823600

H -2.31281600 -2.11747700 0.37951900

C 1.17591300 -1.11885900 -0.97380100

H -0.33920000 0.15954500 -1.85610800

H 0.48507300 0.84726700 -0.44304700

H -0.94012100 -3.84150700 -0.75559700

H -0.05355000 -3.05756900 0.56033700

H 1.92712800 -0.75586400 -1.68329700

H 1.66605800 -1.21895300 0.00239800

C 1.83342900 5.40287000 1.91348400

C 1.50101700 6.83578000 2.31750500

C 0.08243500 7.16823100 1.86527400

C -0.96827200 6.21184600 2.44626400

C 0.85228100 4.37304700 2.49165500

H -0.19243500 8.19263600 2.14377000

H 1.59909200 6.96273600 3.40234600

H 2.21341800 7.52954300 1.85793200

H 1.80414200 5.33223400 0.81782200

H 2.84702400 5.12733100 2.22849700

H 0.03948000 7.10713100 0.76948200

N -0.52850200 4.81958700 2.19064300

O -1.43637800 3.93653400 2.67949900

C -2.28576800 6.40010000 1.68869400

H -3.07377100 5.77357200 2.11556800

H -2.60574400 7.44617100 1.74492200

H -2.15688300 6.13099600 0.63513400

C -1.21575200 6.49300800 3.93724200

H -1.77728900 7.42679000 4.05305300

H -1.80517100 5.68041100 4.37142000

H -0.28890900 6.59308200 4.50759500

C 1.04800800 3.03981600 1.76314900

H 2.08699600 2.70585100 1.86004800

H 0.39133800 2.26790500 2.17400300

H 0.81490100 3.16100600 0.69994500

C 1.09178400 4.15146900 3.99393200

H 0.26415700 3.57451600 4.41625000

H 2.01844200 3.58669000 4.14526100

H 1.18204900 5.08782800 4.55043600

C 0.62311800 -2.47449600 -1.40526900

H 1.42050100 -3.22390800 -1.44290900

H 0.20575400 -2.39407100 -2.41867700

**TS1A-delte:**

C -3.48439500 0.09445100 0.73217700

C -1.99641800 0.34815000 0.56180900

C -1.48951500 1.58949700 0.65897700

C -2.29875300 2.81791200 0.99105400

C -3.80589700 2.59134300 0.83865100

C -4.17673800 1.24806800 1.40552800

H -0.42293200 1.74922300 0.53643600

H -3.95570400 -0.06993700 -0.25228500

H -3.62489400 -0.83454900 1.29691100

H -2.08624800 3.12423900 2.02593700

H -1.97983500 3.65184500 0.35454300

H -4.36985700 3.39727400 1.31840300

H -4.06605000 2.60794900 -0.23290400

H -3.65712200 1.28082600 2.73112500

H -5.22968100 1.08379400 1.65043300

N -1.21844800 -0.81785700 0.38939400

C -1.66655400 -1.71245700 -0.68294300

C 0.22721500 -0.63566900 0.38798200

C -0.99431800 -3.07819000 -0.58483200

H -1.44623400 -1.26351400 -1.66983300

H -2.74936900 -1.83439600 -0.61592800

C 0.95316100 -1.97372700 0.50333900

H 0.55862100 -0.10873400 -0.52751600

H 0.48803400 -0.00441000 1.24178600

H -1.34112100 -3.70857600 -1.41087800

H -1.30617200 -3.55609900 0.35218200

H 2.03360000 -1.79591900 0.47375900

H 0.71805500 -2.41524000 1.47956000

C 0.52493300 -2.93232800 -0.60466400

H 1.01271900 -3.90573600 -0.48723000

H 0.83783900 -2.52846800 -1.57767600

C -6.01100500 1.93095000 6.17379400

C -5.81283100 0.67609900 7.01981800

C -5.44087700 -0.49595900 6.11556800

C -4.18570100 -0.22474900 5.27393600

C -4.77569900 2.28479100 5.33313200

H -5.27510500 -1.40840200 6.70073600

H -5.03327300 0.84089900 7.77332700

H -6.73202100 0.44706000 7.56991800

H -6.85648300 1.76711800 5.49221000

H -6.26176300 2.79450900 6.80142300

H -6.27778100 -0.69438400 5.43256200

N -4.37962000 1.07103800 4.58263900

O -3.28688700 1.34493900 3.79225200

C -4.07660000 -1.29111400 4.17959800

H -3.14146200 -1.18468500 3.62200600

H -4.09951400 -2.29082300 4.62675800

H -4.91629400 -1.20251500 3.48121400

C -2.91031300 -0.28179300 6.13071500

H -2.67740700 -1.32354700 6.37804400

H -2.06785000 0.13150000 5.56853200

H -3.00380300 0.26526400 7.07172100

C -5.16555000 3.33579100 4.28936900

H -5.62273900 4.20153000 4.78052200

H -4.28625200 3.67895200 3.73659200

H -5.88657100 2.91666300 3.57902600

C -3.64997300 2.86560700 6.20461000

H -2.71720400 2.89506500 5.63364300

H -3.90192100 3.89004500 6.50086900

H -3.48034300 2.29234500 7.11890000

**TS1A-epsilon:**

C -3.09793100 0.59264300 0.96560500

C -1.67567000 0.51862900 0.62384000

C -0.91893900 1.64929800 0.62579400

C -1.44073100 3.00255500 1.03058400

C -2.96755400 3.08157400 1.04281200

C -3.54573900 1.83305600 1.70456800

H 0.12948000 1.60596500 0.34988000

H -3.75562200 0.61322700 -0.27707000

H -3.50987300 -0.34117500 1.35649600

H -1.05601400 3.25499500 2.03179200

H -1.02674600 3.76451200 0.35789900

H -3.29503500 3.98700600 1.56525600

H -3.34848100 3.13633300 0.01441000

H -3.19695300 1.77611500 2.74849100

H -4.63832000 1.87554900 1.74375200

N -1.16303400 -0.76788200 0.33891700

C -1.93795900 -1.58054700 -0.60621800

C 0.27000900 -0.86183000 0.09609600

C -1.53193600 -3.04845700 -0.52620400

H -1.78134900 -1.21859600 -1.63924300

H -3.00200200 -1.47349000 -0.39290900

C 0.74579200 -2.31193700 0.16324000

H 0.53570900 -0.42313200 -0.88526400

H 0.78677800 -0.28144400 0.86513500

H -2.12380200 -3.62379300 -1.24632400

H -1.76695000 -3.42635700 0.47667700

H 1.81858300 -2.34466600 -0.05597400

H 0.60774700 -2.67443900 1.18936800

C -0.03751300 -3.20225000 -0.79789800

H 0.27419800 -4.24748700 -0.69923200

H 0.17413100 -2.89753500 -1.83234300

C -6.30165800 3.30592800 -2.47975300

C -5.61366500 3.47901100 -3.82951400

C -4.11444000 3.26096400 -3.65606100

C -3.76874700 1.88448200 -3.07043600

C -6.04743000 1.93016800 -1.84849200

H -3.58501600 3.36871800 -4.61021200

H -6.02386700 2.77681400 -4.56515100

H -5.80308000 4.48452500 -4.22043600

H -5.92508300 4.07593000 -1.79272700

H -7.38540900 3.44912800 -2.56486000

H -3.72273900 4.03264300 -2.97938500

N -4.58243100 1.68039000 -1.84347800

O -4.31412000 0.46349800 -1.29223900

C -2.29888500 1.88795400 -2.64456600

H -1.99995600 0.91150800 -2.25504600

H -1.66524100 2.12745600 -3.50553800

H -2.12129200 2.63373100 -1.86311400

C -3.96660300 0.76716500 -4.10869200

H -3.16592100 0.81035600 -4.85505600

H -3.92285300 -0.20824100 -3.61543400

H -4.91664700 0.84504100 -4.64227200

C -6.49698700 1.97171700 -0.38674500

H -7.56272700 2.21746700 -0.33057500

H -6.33953900 1.00413100 0.09835300

H -5.93667700 2.73776000 0.15901000

C -6.83796900 0.82649200 -2.57026800

H -6.49284300 -0.15595200 -2.23593200

H -7.90282100 0.91679800 -2.32948500

H -6.74202300 0.87726400 -3.65719500

**TS1A-gamma:**

C -3.28883800 0.48152900 1.08124500

C -1.88023200 0.63621500 0.53795000

C -1.28707700 1.84124000 0.47570100

C -1.92692100 3.12410500 0.94225200

C -3.37414800 2.95482400 1.31803500

C -3.67608400 1.64602600 1.99655600

H -0.28323900 1.93741500 0.07523000

H -4.00966700 0.43085600 0.25387900

H -3.35423400 -0.46677600 1.62630100

H -1.37376900 3.51744100 1.81475000

H -1.82038100 3.89602100 0.16850700

H -3.86846500 3.84428400 1.71885900

H -4.06835000 2.87447600 0.08285900

H -3.09037200 1.57974500 2.92845700

H -4.73168100 1.57467900 2.27554900

N -1.24743100 -0.57601900 0.18201200

C -2.02105500 -1.42565800 -0.73125300

C 0.15244900 -0.48200600 -0.21096900

C -1.43583300 -2.83194700 -0.80983100

H -2.03958600 -0.97661800 -1.74181400

H -3.05351000 -1.47693200 -0.38061300

C 0.79808500 -1.86344100 -0.28779500

H 0.25644300 0.03402400 -1.18472600

H 0.67347100 0.12279200 0.53663000

H -2.02918400 -3.42878500 -1.51100400

H -1.51633100 -3.30211900 0.17820400

H 1.83993800 -1.75239800 -0.60735000

H 0.80624300 -2.30150200 0.71792600

C 0.02925500 -2.78064100 -1.23539300

H 0.46844400 -3.78379100 -1.24749500

H 0.09545200 -2.38556500 -2.25869900

C -7.92342000 4.46028200 -0.61806800

C -7.72563200 5.48271200 -1.73360200

C -6.28732400 5.99233300 -1.70840900

C -5.25302000 4.86500500 -1.83804800

C -6.94398900 3.28098800 -0.70815000

H -6.11142000 6.71710700 -2.51227200

H -7.95706000 5.03643400 -2.70825600

H -8.42139800 6.31852500 -1.60239300

H -7.77384500 4.95937900 0.34872600

H -8.94518900 4.06224500 -0.62398300

H -6.11312600 6.51232400 -0.75686200

N -5.57610000 3.83806800 -0.82005300

O -4.65639300 2.81586000 -0.88031800

C -3.86826500 5.41395600 -1.48164300

H -3.09425000 4.66456000 -1.66828100

H -3.64667000 6.29691700 -2.09076200

H -3.83367200 5.70123200 -0.42503000

C -5.19769500 4.31477000 -3.27270700

H -4.68975400 5.03373000 -3.92524300

H -4.63041100 3.37955000 -3.28908800

H -6.18711200 4.12968400 -3.69722000

C -6.98991700 2.49585900 0.60595700

H -8.02001900 2.19875000 0.83036700

H -6.37810200 1.59132600 0.54119100

H -6.61749200 3.11457400 1.42977700

C -7.31717000 2.32640900 -1.85413700

H -6.49221300 1.63295500 -2.04252100

H -8.19980000 1.74007200 -1.57510200

H -7.55179600 2.84878300 -2.78439400

**TS1B:**

C -3.56158200 0.77335100 0.09598400

C -2.27435400 0.18777800 0.39430500

C -1.25143000 0.93112200 1.21771000

C -1.86796900 1.97314400 2.15406800

C -2.91847800 2.81406300 1.43291500

C -4.06294200 1.91407500 0.97349100

H -0.64018700 0.21977700 1.78110400

H -3.39089700 1.11925000 -1.08178700

H -4.32326900 0.00996800 -0.09466400

H -2.33990200 1.46373600 3.00378400

H -1.06683700 2.59770000 2.56130000

H -3.29277000 3.60147800 2.09523600

H -2.46127500 3.30868500 0.56489800

H -4.58192400 1.52511500 1.86040300

H -4.80312200 2.48910000 0.40727200

H -0.57611200 1.42695500 0.50494100

N -1.94058000 -0.93135700 -0.19508900

C -0.63408500 -1.59153500 -0.19487900

C -0.61473500 -2.74283200 0.81566400

C -0.33731800 -2.09328000 -1.61133400

H 0.13146900 -0.85756100 0.07997300

C 0.72153800 -3.48730800 0.76900100

H -1.43459000 -3.43046900 0.56771700

H -0.81376600 -2.35429200 1.82155000

C 0.99912400 -2.83734100 -1.65198800

H -1.15018200 -2.76093900 -1.92555000

H -0.33921900 -1.24456700 -2.30441900

C 1.02346900 -3.98916500 -0.64476900

H 0.70673300 -4.32093000 1.47882100

H 1.52469600 -2.80994700 1.09160200

H 1.18193800 -3.20980300 -2.66514700

H 1.81335700 -2.13668600 -1.41979200

H 1.99537000 -4.49370700 -0.66809900

H 0.26875500 -4.73406400 -0.93141900

O -3.40920100 1.02443200 -2.53156600

H -3.25063000 -0.65230400 -2.65777000

H -3.16675600 1.82519800 -3.01401000

O -3.51431700 -1.55988500 -2.33690500

H -4.47050800 -1.39654100 -2.20316000

H -2.63805500 -1.32510800 -0.87173100

O -5.78047400 0.05265600 -2.19227000

H -4.95116500 0.59111700 -2.40906200

H -6.27361100 0.00729800 -3.02279700

**TS1C:**

C -3.62841500 0.74440000 0.17136900

C -2.39467400 0.09711400 0.53282700

C -1.32080200 0.85552200 1.26231400

C -1.87987900 1.95239700 2.17060800

C -2.90709900 2.80793900 1.43108300

C -4.08726400 1.94337400 0.99076500

H -0.68018000 0.15878700 1.81026600

H -3.26335000 1.03438800 -0.96823600

H -4.42759800 0.03853100 -0.07345000

H -2.35548300 1.49259900 3.04629300

H -1.04985200 2.56177200 2.54075400

H -3.25306400 3.62417600 2.07345800

H -2.43468700 3.26513400 0.55101200

H -4.63802500 1.61082100 1.88098300

H -4.79001300 2.52855500 0.38925600

H -0.68820700 1.30247300 0.48018600

N -2.12414000 -1.08528600 0.03014000

O -2.92131800 0.79875600 -2.40027400

H -3.09292900 -0.89472700 -2.51862500

H -2.57670000 1.55547900 -2.89236100

O -3.53589900 -1.71913700 -2.17927500

H -4.46087600 -1.39898600 -2.17209700

H -2.81625500 -1.47363600 -0.66274600

O -5.47075500 0.25892300 -2.41948300

H -4.53252900 0.61990500 -2.51968100

H -5.83730400 0.25643000 -3.31433200

C -0.79918600 -1.62532100 -0.08136800

C -0.30325100 -2.51272100 0.86754600

C -0.04382700 -1.25576700 -1.19264700

C 0.98005800 -3.02957000 0.70488600

H -0.92058900 -2.79034600 1.71656500

C 1.23429000 -1.78308800 -1.34962700

H -0.48004700 -0.56398500 -1.91017100

C 1.74641100 -2.66650200 -0.40062600

H 1.37847000 -3.72109900 1.44059400

H 1.82995000 -1.50445600 -2.21323200

H 2.74417500 -3.07578400 -0.52457200

**TS2A-nitrogen:**

C -2.70187400 -1.98824300 1.58203500

C -2.24770000 -0.99399800 0.53090600

C -3.17312000 -0.03987300 0.05297800

C -4.50104300 -0.10618800 0.42644300

C -5.02811700 -1.07921100 1.32320700

C -3.98256500 -1.57288000 2.30958700

H -2.93539600 0.67608100 -0.72273600

H -2.85323700 -2.94427100 1.06634100

H -1.90060000 -2.13167900 2.31333900

H -5.14804200 0.58228800 -0.11944400

H -6.00246600 -0.83434400 1.74894400

H -5.16617800 -2.03218000 0.46718400

H -3.74261400 -0.77809500 3.03041600

H -4.34487300 -2.43058100 2.88505400

N -1.00982000 -1.10324600 0.05955900

C -0.12353700 -2.24191800 0.33197200

C -0.43955700 -0.20150900 -0.94693100

C 1.22207700 -1.76093100 0.86979300

H 0.00807100 -2.76518000 -0.62298800

H -0.60610300 -2.94157000 1.00743000

C 0.91236300 0.33374300 -0.47130400

H -0.32573500 -0.77356900 -1.87801800

H -1.11965000 0.62342200 -1.13788300

H 1.86245600 -2.63007700 1.05238100

H 1.06485300 -1.25810700 1.83241700

H 1.32857500 0.97505200 -1.25487200

H 0.74357000 0.96219000 0.41170700

C -4.53916100 0.43241900 -5.23186700

C -6.01819700 0.29871700 -5.58540600

C -6.79553500 -0.16465400 -4.35616000

C -6.64519200 0.77795100 -3.15475500

C -4.28456300 1.40383100 -4.07262800

H -7.86573600 -0.25462000 -4.57889500

H -6.41506600 1.25009400 -5.95960400

H -6.14001900 -0.42846300 -6.39529400

H -4.15597500 -0.55598100 -4.94869500

H -3.95744600 0.77885300 -6.09497000

H -6.43479100 -1.15965600 -4.06618500

N -5.15682500 1.00845100 -2.87822100

O -4.96504200 1.86121900 -1.82575400

C -7.19426700 0.10137700 -1.89753500

H -7.19743100 0.80040400 -1.05829700

H -8.22201500 -0.22627900 -2.08670700

H -6.60326700 -0.78093300 -1.62225400

C -7.36331000 2.10963300 -3.34794400

H -8.43062700 1.97030400 -3.14698100

H -6.95925700 2.82861200 -2.63130100

H -7.26946000 2.50953400 -4.35956500

C -2.84728900 1.25383900 -3.57330600

H -2.15118800 1.40870200 -4.40433200

H -2.64668200 1.98852200 -2.79014700

H -2.68350900 0.24752000 -3.17061100

C -4.52410900 2.86187700 -4.45114200

H -4.64589500 3.44245000 -3.53376200

H -3.65078700 3.23651600 -4.99531200

H -5.39575400 3.00477100 -5.09279700

C 1.87551600 -0.80064600 -0.12389700

H 2.80906600 -0.39840600 0.28175800

H 2.13163800 -1.35178800 -1.03883300

O -4.97892700 -2.99267100 -0.50331100

H -4.83262300 -2.20664200 -1.96839000

H -5.60862600 -3.71886000 -0.39648300

O -4.37592000 -1.73947000 -2.72511000

H -3.45166000 -1.96861400 -2.51859400

H -4.80847500 0.05510900 -2.63774200

O -2.50462800 -3.34397200 -1.29652900

H -3.44409200 -3.33304000 -0.93021500

H -2.47678900 -4.13091300 -1.85886500

**TS2A-oxygen:**

C -1.53477900 -1.66852500 -0.73419900

C -1.49504200 -0.81459400 0.51565600

C -2.71606600 -0.27945300 0.99485700

C -3.89423200 -0.52573400 0.31549600

C -4.00095200 -1.38843600 -0.81354400

C -2.86988100 -2.39540800 -0.89999500

H -2.72769200 0.43096700 1.81028800

H -1.39482500 -1.00323200 -1.59954200

H -0.71985400 -2.39479800 -0.72757600

H -4.78309900 0.02779700 0.61199400

H -5.00928400 -1.76596400 -0.99208400

H -3.78096700 -0.63135500 -1.78020900

H -2.96956900 -3.16362000 -0.11934300

H -2.87667900 -2.88882600 -1.87575500

N -0.31920600 -0.53652800 1.07082400

C 0.98062500 -0.90192700 0.48933200

C -0.15461100 0.10183100 2.38385400

C 1.68439600 -1.93156100 1.37141100

H 1.57303900 0.02117300 0.45268400

H 0.85554000 -1.24517400 -0.53407900

C 0.52095200 -0.88263000 3.34260000

H 0.46623700 0.99530700 2.24664900

H -1.11770000 0.41466100 2.77589300

H 2.65367200 -2.18043000 0.92817700

H 1.08429900 -2.84980700 1.39330800

H 0.66023000 -0.39037300 4.31036100

H -0.15702000 -1.73046300 3.49906000

C -8.96519000 3.04138900 -1.26867000

C -8.16482400 4.19085800 -1.87570100

C -6.72089000 4.12968300 -1.38307300

C -6.04942000 2.78208000 -1.68918000

C -8.34766500 1.66268900 -1.55328300

H -6.11891200 4.93081400 -1.82909600

H -8.19686700 4.14230100 -2.97095400

H -8.61608900 5.14934600 -1.59619500

H -9.00557000 3.17632600 -0.17974500

H -9.99842300 3.04458500 -1.63641700

H -6.71013500 4.27788300 -0.29511100

N -6.93073500 1.73077800 -1.13762700

O -6.32371000 0.46272800 -1.24294900

C -4.72766800 2.69165200 -0.91932600

H -4.17174300 1.79582600 -1.21230900

H -4.10958700 3.57193700 -1.12967200

H -4.91996700 2.64493800 0.15762300

C -5.72440600 2.66631300 -3.19062900

H -4.85886600 3.29711000 -3.42522700

H -5.46401400 1.63770800 -3.45415200

H -6.54475000 2.99013800 -3.83492300

C -9.02069600 0.62636000 -0.64722400

H -10.10813000 0.66712600 -0.77446500

H -8.67963900 -0.38394300 -0.88503500

H -8.77936300 0.83293200 0.39998700

C -8.58498400 1.24477300 -3.01702800

H -7.98236300 0.36911000 -3.28133400

H -9.63694300 0.96851800 -3.14988700

H -8.36452400 2.04177200 -3.73083300

C 1.85755900 -1.37638100 2.78654200

H 2.28582400 -2.13584400 3.44758000

H 2.56715400 -0.53857400 2.75671700

O -3.36362000 -0.08852400 -3.05340700

H -3.21396500 0.86651400 -3.00712200

H -4.74487500 -0.40353100 -3.51352200

O -5.70901100 -0.75262200 -3.68337900

H -5.94047500 -0.45575100 -4.57525500

H -6.26742100 0.18832600 -2.18970700

O -3.41516700 -2.55910000 -4.18298500

H -4.36513200 -2.41446900 -4.03990300

H -3.06673200 -1.67945600 -3.93284700

**TS2B-enamine-beta:**

C -3.11604000 2.13259400 0.18634300

C -2.63188300 0.94598000 0.65931500

C -1.70180800 0.88889000 1.84788200

C -1.71822600 2.18348800 2.66676200

C -1.66273900 3.41926500 1.77184200

C -2.83395500 3.40675500 0.80840800

H -1.99371000 0.04513800 2.48516000

H -3.78598600 2.10499000 -0.67235800

H -2.64483000 2.22536800 3.25021200

H -0.88621000 2.17596100 3.37843600

H -1.67728000 4.32871100 2.38242200

H -0.71532500 3.42626700 1.21104400

H -3.93241600 3.65480300 1.54149600

H -2.87129200 4.26002900 0.12537300

H -0.67965400 0.67518500 1.50158400

N -3.04478500 -0.26790500 0.13123700

C -2.13558700 -1.40623100 0.01371600

C -2.94261300 -2.66599800 -0.30165700

C -1.04392000 -1.18418300 -1.04178000

H -1.65300900 -1.54755100 0.98910300

C -2.03450800 -3.89109200 -0.43267300

H -3.48010600 -2.50920700 -1.24933800

H -3.69858400 -2.81554300 0.47647400

C -0.13124500 -2.40658200 -1.16561200

H -1.53395600 -0.99044300 -2.00743300

H -0.46636900 -0.28595400 -0.79212800

C -0.94204400 -3.66571800 -1.48056000

H -2.63194100 -4.77352300 -0.68562100

H -1.56468600 -4.09494200 0.53947400

H 0.62688600 -2.23489100 -1.93733500

H 0.40762400 -2.55166600 -0.21878800

H -0.28334700 -4.53932900 -1.53571700

H -1.40859200 -3.55475900 -2.46935400

H -3.62334200 -0.15715000 -0.69842500

C -7.66434000 3.02853200 -0.26848900

C -8.43968400 2.04578500 0.60335100

C -7.45955400 1.21609800 1.42714600

C -6.52909700 2.07295400 2.29736500

C -6.74000200 3.94641700 0.54311600

H -7.99041200 0.51115700 2.07832800

H -9.13463100 2.58227100 1.26059600

H -9.05205100 1.38926600 -0.02460000

H -7.04619300 2.45920300 -0.97638800

H -8.34386300 3.65388800 -0.86007200

H -6.83555900 0.62266500 0.74455600

N -5.90012100 3.09641700 1.42489500

O -5.00672600 3.85076000 2.11249300

C -5.41605300 1.18155800 2.85031000

H -4.77422100 1.74535800 3.53168900

H -5.85867000 0.34785800 3.40664600

H -4.80604700 0.77495700 2.03841400

C -7.27944400 2.68645200 3.49315900

H -7.47650800 1.91287000 4.24366200

H -6.65967900 3.46118400 3.95458700

H -8.24000200 3.12719300 3.21783600

C -5.79482000 4.66972000 -0.42048500

H -6.37809100 5.21407000 -1.17098500

H -5.16524800 5.38527500 0.11449300

H -5.14918000 3.95341300 -0.93863900

C -7.53550200 5.01483300 1.31273000

H -6.88796000 5.48377000 2.05943400

H -7.88051200 5.79131600 0.62085700

H -8.41634600 4.61175800 1.81709700

**TS2B-enamine-delte:**

C -3.64389300 0.18459700 1.01898000

C -2.30831700 0.05754900 1.04121600

C -1.38479400 1.21320700 0.72121600

C -2.11946000 2.52650500 0.66786900

C -3.45600400 2.46285100 -0.02185500

C -4.36803000 1.46646000 0.70138800

H -0.58068300 1.24327800 1.46710300

H -4.25264900 -0.68091600 1.27963000

H -2.44285300 2.82929500 2.02124800

H -1.49666300 3.40185900 0.46343000

H -3.92268000 3.45113100 -0.07437300

H -3.30568300 2.11951000 -1.05922000

H -4.74333800 1.92953900 1.62611100

H -5.24885800 1.25269900 0.08453100

H -0.88718200 1.03253800 -0.24847800

N -1.66348900 -1.12155200 1.45797400

C -0.55007000 -1.63306900 0.65581300

C 0.07099400 -2.83129300 1.37325800

C -0.95315800 -2.01231400 -0.77507700

H 0.21080400 -0.84201700 0.60434600

C 1.25498100 -3.40328300 0.59003000

H -0.69995700 -3.60921000 1.48504000

H 0.37211100 -2.53057700 2.38234100

C 0.23331500 -2.57436700 -1.56198800

H -1.74951600 -2.76967800 -0.71735800

H -1.38374600 -1.14049700 -1.28310000

C 0.85104300 -3.77343500 -0.83910800

H 1.66225300 -4.27653800 1.11112800

H 2.05755600 -2.65355800 0.55405000

H -0.08114800 -2.85594700 -2.57294600

H 0.99521500 -1.79003900 -1.67462900

H 1.71813600 -4.14844600 -1.39406600

H 0.11626500 -4.59002000 -0.80572700

H -2.34218800 -1.86248900 1.62563500

C -0.28192400 5.48003300 4.49685100

C -1.25407100 6.62033700 4.78697100

C -2.27746700 6.71743800 3.65911600

C -3.05003100 5.40872000 3.43938000

C -0.98674500 4.12913100 4.30750500

H -3.00129700 7.51853200 3.85097400

H -1.75678600 6.46002000 5.74828500

H -0.70657800 7.56483200 4.87738700

H 0.27088100 5.71268200 3.57673400

H 0.45535400 5.37567200 5.30182100

H -1.75295100 6.96881400 2.72747900

N -2.05793600 4.31747300 3.30141200

O -2.70296100 3.12290200 3.07721400

C -3.79899200 5.49045900 2.10593400

H -4.43672200 4.61322400 1.96345900

H -4.43287800 6.38350800 2.08550600

H -3.08810400 5.54811600 1.27428400

C -4.07934200 5.16846500 4.55601600

H -4.92996200 5.84719300 4.42770800

H -4.45130400 4.14101800 4.50216900

H -3.67186000 5.33891700 5.55513300

C 0.00448900 3.12676000 3.70837400

H 0.90606600 3.07488300 4.32827500

H -0.43652000 2.12733000 3.65820600

H 0.29366700 3.43702600 2.69803000

C -1.49080600 3.56506200 5.64608100

H -2.17311800 2.72993100 5.46259000

H -0.64379000 3.19111000 6.23208100

H -2.00740600 4.31016600 6.25551500

**TS2B-enamine-epsilon:**

C -3.36974100 0.60129700 0.80320900

C -2.15028200 0.05338900 1.02753900

C -0.99973900 0.87675500 1.40359900

C -1.23542800 2.35295700 1.62476000

C -2.35902100 2.88245500 0.73413400

C -3.63856200 2.07346800 0.94116700

H -0.36732000 0.39636400 2.15524200

H -4.20458400 -0.05440600 0.55883300

H -1.49521000 2.52711200 2.68083300

H -0.29935300 2.90191500 1.44406100

H -2.53498100 3.94497100 0.93426300

H -2.05859700 2.79074700 -0.31602400

H -4.06540300 2.29516000 1.93232700

H -4.39808200 2.38004100 0.21248500

H -0.04195400 0.83477100 0.38370600

N -1.94322900 -1.34118200 1.00702200

C -0.79882900 -1.84654100 0.23977800

C -0.63098600 -3.34244700 0.49931800

C -0.91369600 -1.56835500 -1.26363400

H 0.09988700 -1.33694300 0.61069400

C 0.57886300 -3.89903400 -0.25598100

H -1.54120400 -3.86482900 0.16618500

H -0.53773800 -3.51518300 1.57697400

C 0.29465500 -2.11884300 -2.02233300

H -1.83530400 -2.04504100 -1.63293700

H -1.01412000 -0.48993900 -1.43212300

C 0.47796500 -3.61509300 -1.75689400

H 0.67398500 -4.97541300 -0.07495300

H 1.49143100 -3.42886700 0.13724400

H 0.18771300 -1.93044300 -3.09674700

H 1.18881700 -1.57343600 -1.69014500

H 1.36870500 -3.98930200 -2.27409000

H -0.38227300 -4.16191000 -2.16848300

H -2.79199400 -1.82010700 0.70988900

C 2.70665200 3.88379200 -0.96520400

C 4.03165900 3.48172700 -0.32591000

C 3.75404500 2.68088300 0.94202500

C 2.90282800 1.42958500 0.68533700

C 1.80851400 2.68305100 -1.29433800

H 4.68700900 2.36781400 1.42530200

H 4.63610100 2.89458000 -1.02767300

H 4.61666400 4.37546300 -0.08388800

H 2.16504600 4.53865000 -0.26931900

H 2.86830900 4.45367700 -1.88782200

H 3.21812700 3.32255100 1.65453600

N 1.69450300 1.84068100 -0.07503800

O 0.89425900 0.76480700 -0.31698000

C 2.42110300 0.87844600 2.03019400

H 1.89930400 -0.07463000 1.90184900

H 3.27935300 0.71004700 2.68935000

H 1.74430200 1.58933100 2.51633100

C 3.71591200 0.33069700 -0.01768800

H 4.42183900 -0.11483200 0.69186300

H 3.04491200 -0.45846000 -0.37105400

H 4.29412600 0.70268800 -0.86624200

C 0.40427100 3.19305400 -1.62115500

H 0.44746700 3.88258500 -2.47089500

H -0.26158100 2.36552100 -1.88204200

H -0.01609700 3.72823600 -0.76390700

C 2.33541400 1.90300200 -2.51007600

H 1.80036200 0.95290900 -2.60290800

H 2.16274200 2.48313500 -3.42311900

H 3.40607900 1.69542800 -2.45119200

**TS2B-enamine-gamma:**

C -3.67354700 0.97828700 0.90804400

C -2.45662500 0.42681600 1.03198600

C -1.22803100 1.23612800 1.38105800

C -1.58121800 2.62098300 1.93447700

C -2.67652200 3.25446200 1.11590500

C -3.94720000 2.45030200 1.06326100

H -0.63540100 0.67339700 2.11184100

H -4.51914200 0.33839400 0.65730200

H -1.92972500 2.51563700 2.97494300

H -0.68895300 3.25475400 1.95883800

H -2.80614200 4.33267700 1.23348000

H -2.11750100 3.17013000 -0.18645000

H -4.53243600 2.64257600 1.98123100

H -4.58219200 2.79980800 0.23880700

H -0.60098300 1.35361700 0.48710000

N -2.23595700 -0.95982900 0.89854300

C -1.15595400 -1.36503500 -0.01082000

C -1.06174400 -2.88961700 -0.03343600

C -1.32782100 -0.81142900 -1.43153700

H -0.21430400 -0.97602500 0.40109700

C 0.06204700 -3.36473500 -0.95790300

H -2.02117100 -3.29409900 -0.39145900

H -0.91740800 -3.25995100 0.98727200

C -0.20237900 -1.28054600 -2.35599400

H -2.29703900 -1.15968500 -1.82222700

H -1.37693700 0.28465600 -1.39770900

C -0.10752500 -2.80813700 -2.37397200

H 0.09306700 -4.45962700 -0.97811900

H 1.02629300 -3.02810600 -0.55246000

H -0.35824600 -0.89311300 -3.36929100

H 0.75126600 -0.86360300 -2.00195100

H 0.72303400 -3.13253400 -3.01083100

H -1.02661800 -3.21954900 -2.81440100

H -3.09959200 -1.42418100 0.61921500

C -3.32259200 4.01974900 -4.20693200

C -2.38491800 5.14513400 -4.63446900

C -1.93123400 5.91956900 -3.40069000

C -1.24022800 5.02950900 -2.35770300

C -2.68792100 3.06267100 -3.18725900

H -1.24407600 6.72971300 -3.67236800

H -1.51963500 4.74304400 -5.17500000

H -2.89983600 5.81682400 -5.32987100

H -4.21889200 4.46331500 -3.75334600

H -3.65361000 3.43062400 -5.07068600

H -2.81022000 6.38219100 -2.93209900

N -2.13182300 3.87697400 -2.07816500

O -1.53783900 3.04918500 -1.15199800

C -1.11373300 5.81072600 -1.04646900

H -0.56795800 5.23106400 -0.29614600

H -0.57220900 6.74727600 -1.21729000

H -2.10672700 6.04960200 -0.65167500

C 0.16694600 4.61631700 -2.81679900

H 0.84059400 5.47906800 -2.76753100

H 0.55947500 3.83813300 -2.15597900

H 0.18577900 4.24185900 -3.84280500

C -3.78806400 2.18949800 -2.57867900

H -4.28676000 1.61183900 -3.36454200

H -3.37649300 1.49410700 -1.84070400

H -4.53555300 2.81963800 -2.08546100

C -1.64401100 2.15057600 -3.85774000

H -0.99077900 1.70626700 -3.10126500

H -2.14851700 1.33822900 -4.39309300

H -1.02118800 2.68163400 -4.58219100

**TS2B-imine-alpha:**

C -3.68057900 1.09792000 0.52420100

C -2.46193000 0.33945600 0.22389400

C -1.15514300 1.10924600 0.35890200

C -1.15266600 2.05095500 1.57010700

C -2.37500900 2.96672900 1.57433700

C -3.65543100 2.13340800 1.62464800

H -0.30165700 0.43011800 0.41437200

H -3.78130700 1.91726600 -0.64035200

H -4.59457000 0.50950900 0.41621800

H -1.14871200 1.45249300 2.49123100

H -0.22861100 2.63859600 1.56851700

H -2.33641200 3.65259900 2.42721400

H -2.37831000 3.58032800 0.66331000

H -3.71413700 1.62187000 2.59927900

H -4.54331000 2.77148900 1.55984600

H -1.03309300 1.70575500 -0.55788400

N -2.60393400 -0.86461400 -0.20671100

C -1.44550000 -1.65913400 -0.58733400

C -0.91519900 -2.41872900 0.63836500

C -1.86409500 -2.64856000 -1.67907700

H -0.62866900 -1.03827600 -0.99038800

C 0.23037000 -3.36214000 0.26582800

H -1.74991500 -2.99069600 1.06605800

H -0.59605200 -1.70128700 1.40465400

C -0.71763500 -3.58741000 -2.05872600

H -2.71670200 -3.22862700 -1.30119700

H -2.21913300 -2.09339400 -2.55510400

C -0.19249200 -4.33896400 -0.83358100

H 0.56933300 -3.90762800 1.15362400

H 1.08602200 -2.77017700 -0.08851800

H -1.04960600 -4.29393700 -2.82750900

H 0.10162300 -3.00226600 -2.50013700

H 0.64746600 -4.98540300 -1.11168500

H -0.98565000 -4.99364700 -0.44661200

C -5.79947500 1.08525000 -4.23169100

C -6.89393300 2.14339100 -4.33556800

C -7.25806600 2.63852000 -2.93879300

C -6.05479700 3.20992900 -2.17546500

C -4.53926700 1.59517500 -3.51792500

H -8.03504000 3.41087100 -2.98362300

H -6.56211300 2.97836500 -4.96453800

H -7.77827200 1.71966400 -4.82347400

H -6.18926500 0.22606100 -3.67000600

H -5.50836200 0.72012600 -5.22366200

H -7.66603300 1.79857600 -2.36093400

N -4.96156200 2.21396600 -2.24104100

O -3.88401800 2.62888800 -1.52152200

C -6.42554600 3.36633600 -0.69789200

H -5.63417900 3.88631700 -0.15142800

H -7.34739700 3.95037000 -0.60565000

H -6.58708400 2.38499000 -0.23877600

C -5.64586500 4.58845900 -2.72037400

H -6.37700600 5.34172000 -2.40636500

H -4.66899500 4.87174100 -2.31712700

H -5.59349100 4.61570100 -3.81130700

C -3.64996300 0.39975100 -3.16146800

H -3.46218100 -0.19768100 -4.06065500

H -2.68637000 0.73024300 -2.76157400

H -4.13076900 -0.23307200 -2.40893600

C -3.73390800 2.55331500 -4.41045100

H -2.97044400 3.06149800 -3.81374000

H -3.22903500 1.98598500 -5.20017200

H -4.35738200 3.30871300 -4.89451600

**TS2B-imine-beta:**

C -3.60928300 0.28379200 -0.78573700

C -2.23878400 -0.16283300 -0.31514600

C -1.41199100 0.96336900 0.27668400

C -2.17748300 1.66918900 1.40966200

C -3.55498800 2.15108900 0.93937400

C -4.33230000 1.01610500 0.32124000

H -0.43779700 0.61746100 0.62835600

H -3.47914900 0.97276800 -1.64005900

H -4.16752900 -0.58493900 -1.14222200

H -2.31053700 0.97007900 2.24425000

H -1.58602500 2.51232300 1.78116500

H -4.11169200 2.59255900 1.77274800

H -3.42010300 2.95011700 0.18964700

H -4.39782700 0.07751100 1.40032800

H -5.40028200 1.18295900 0.15954100

H -1.22596700 1.68913400 -0.52831800

N -1.93803500 -1.39226400 -0.43488700

C -0.65176000 -1.90773300 0.01857700

C -0.82933500 -3.37693900 0.40971500

C 0.38475500 -1.77544300 -1.10428500

H -0.27822300 -1.36483400 0.90132600

C 0.50136300 -4.01802200 0.80623400

H -1.25985000 -3.90601000 -0.45121200

H -1.55842500 -3.44893800 1.22502000

C 1.71773900 -2.42094700 -0.71856700

H -0.02302200 -2.26175000 -2.00115200

H 0.52352300 -0.71534300 -1.35106700

C 1.53018700 -3.88632500 -0.31903200

H 0.34920300 -5.07184200 1.06416400

H 0.88962800 -3.52475000 1.70859900

H 2.42770400 -2.33772900 -1.54897400

H 2.15381400 -1.87124100 0.12742800

H 2.48834500 -4.32272400 -0.01539100

H 1.18200000 -4.45520900 -1.19231100

C -7.71673100 -0.99127800 3.79805400

C -7.84544100 -2.50082100 3.61328100

C -7.25358900 -2.90155500 2.26471100

C -5.78724000 -2.47543100 2.10315100

C -6.26544100 -0.50051600 3.69522300

H -7.31660800 -3.98562200 2.11244200

H -7.33812800 -3.03118000 4.42810700

H -8.89963300 -2.79462700 3.66143600

H -8.30684900 -0.48839300 3.02021100

H -8.12043000 -0.67592800 4.76764000

H -7.84034900 -2.42648500 1.46709900

N -5.69823200 -1.03376300 2.43542400

O -4.39976800 -0.60269000 2.29110000

C -5.38793400 -2.60898800 0.63051000

H -4.32365000 -2.39953600 0.48268100

H -5.58887700 -3.62741000 0.28032200

H -5.97256000 -1.91155700 0.01976600

C -4.84865500 -3.35635700 2.94387500

H -4.73333600 -4.33542000 2.46569500

H -3.86135100 -2.88934800 3.00615600

H -5.21820200 -3.52746100 3.95796200

C -6.26006900 1.02578000 3.56546600

H -6.81904600 1.47562800 4.39303600

H -5.23773600 1.41373400 3.59120400

H -6.72834500 1.32889200 2.62274100

C -5.45441600 -0.88122800 4.94477600

H -4.38871800 -0.71749200 4.75953800

H -5.75561500 -0.24987500 5.78825500

H -5.59843700 -1.92139900 5.24550100

**TS2B-imine-gamma:**

C -3.78016200 0.99519700 -0.05443800

C -2.37751900 0.43616000 -0.15691700

C -1.29671700 1.45353400 0.16129700

C -1.53841600 2.06139000 1.55751400

C -2.94282000 2.59363700 1.68607500

C -4.02705900 1.60674600 1.33629900

H -0.29410700 1.02546700 0.10798400

H -3.90202200 1.78716100 -0.80680800

H -4.49455400 0.19779900 -0.27180000

H -1.37898100 1.27847900 2.31837700

H -0.80131700 2.84631300 1.75451300

H -3.13362200 3.23886900 2.54792500

H -3.02861500 3.56964700 0.64833900

H -4.03224500 0.79581700 2.08378900

H -5.01464200 2.07774300 1.37480900

H -1.35767400 2.26238600 -0.57994400

N -2.23737200 -0.79526800 -0.44712200

C -0.91355900 -1.40146800 -0.53777600

C -0.45590300 -1.85846600 0.85327000

C -0.98698200 -2.59676800 -1.49149600

H -0.16384600 -0.69851400 -0.93477100

C 0.87618000 -2.60880000 0.78976100

H -1.23627900 -2.51046600 1.26932600

H -0.38000500 -0.98828500 1.51787000

C 0.34802100 -3.33942700 -1.56499900

H -1.77270600 -3.27247300 -1.12762100

H -1.29939200 -2.25060100 -2.48329200

C 0.79940000 -3.79431000 -0.17521600

H 1.16353700 -2.94766800 1.79133900

H 1.66253200 -1.91989200 0.45043000

H 0.26467700 -4.19821900 -2.24009600

H 1.11242800 -2.67483000 -1.99208500

H 1.76981600 -4.29981400 -0.23373600

H 0.07951900 -4.52780600 0.21366500

C -4.87898600 7.35399600 0.80509500

C -3.75480900 8.34997100 0.53387500

C -2.41880500 7.73599300 0.94316400

C -2.12862700 6.40799000 0.22898500

C -4.67424900 6.01305400 0.08555300

H -1.58981700 8.42492200 0.74197900

H -3.73905400 8.63089400 -0.52601700

H -3.92966200 9.27329400 1.09658300

H -4.92803900 7.16199100 1.88528600

H -5.85022100 7.76341300 0.50287400

H -2.43242900 7.54970200 2.02539000

N -3.31046900 5.53390000 0.40979800

O -3.08735100 4.31513100 -0.19082400

C -0.95805600 5.71118700 0.92924900

H -0.65457500 4.81365200 0.38232100

H -0.09767900 6.38673100 0.98383600

H -1.24225700 5.42461300 1.94776800

C -1.74690200 6.63265000 -1.24326200

H -0.73061000 7.03766500 -1.30412200

H -1.76724800 5.68073300 -1.78218100

H -2.41053800 7.33488900 -1.75297900

C -5.64830600 4.98180200 0.66354000

H -6.67363700 5.36308400 0.61020700

H -5.60189200 4.04450200 0.10157200

H -5.40651500 4.77670400 1.71220300

C -4.94182800 6.13752100 -1.42319300

H -4.57710100 5.24508500 -1.94033800

H -6.01976400 6.21867400 -1.60199800

H -4.46882100 7.01559100 -1.86876600

**TS2B:**

C -3.17415200 0.04333400 2.26288200

C -2.14866800 -0.27612000 1.39662700

C -1.53462200 0.79927100 0.51823100

C -1.96633000 2.21086300 0.92067800

C -3.47618900 2.26977100 1.13106800

C -3.85894900 1.37603800 2.30956600

H -0.44257900 0.72545500 0.55787100

H -3.52419500 -0.73313300 2.93450100

H -1.46271900 2.49623300 1.85361000

H -1.64578900 2.92189700 0.15250900

H -3.80950200 3.29707500 1.30963700

H -3.98111700 1.91740700 0.22152300

H -3.60296000 1.88215700 3.25412200

H -4.94675200 1.22705500 2.34516800

H -1.81701900 0.61171900 -0.52613900

N -1.70920000 -1.55705500 1.36385700

C -0.68002600 -1.99857200 0.42995400

C -0.01507600 -3.25407700 1.00587900

C -1.25396300 -2.30819800 -0.96559100

H 0.08862300 -1.22042800 0.32157200

C 1.06958200 -3.79456400 0.07222000

H -0.79565400 -4.01463200 1.15068600

H 0.39586700 -3.02232800 1.99481800

C -0.16828200 -2.83922800 -1.90481100

H -2.04720900 -3.05769700 -0.84560800

H -1.72229400 -1.41131600 -1.38629500

C 0.50691300 -4.08222400 -1.32133300

H 1.51537400 -4.69958300 0.49936500

H 1.87733300 -3.05354200 -0.01134200

H -0.60078500 -3.06401900 -2.88613200

H 0.58879200 -2.05797100 -2.06341200

H 1.30182000 -4.43329400 -1.98891100

H -0.23281300 -4.89139400 -1.24998800

H -2.57914400 -2.44440000 1.39924900

C -5.67738700 -2.98124300 4.09614400

C -6.99434800 -2.86341000 3.33358300

C -6.78210400 -2.04050200 2.06586900

C -5.69272400 -2.61724600 1.15135300

C -4.55087300 -3.59035500 3.25055200

H -7.71149500 -1.95756900 1.48970500

H -7.38156200 -3.85836700 3.08311600

H -7.75135800 -2.38717800 3.96611200

H -5.36510300 -1.97872000 4.42057200

H -5.79937200 -3.58890700 5.00083400

H -6.48521300 -1.02156600 2.35147500

N -4.47686700 -2.84201100 1.96863400

O -3.44255100 -3.27357800 1.20486200

C -5.34033000 -1.58514200 0.07688400

H -4.58696600 -1.98162700 -0.60880400

H -6.23757600 -1.32947000 -0.49694300

H -4.94419500 -0.67450400 0.53725700

C -6.16675100 -3.89671800 0.43811300

H -6.86259300 -3.63585400 -0.36665200

H -5.30534600 -4.40645100 -0.00311100

H -6.68202700 -4.59113100 1.10510200

C -3.21307800 -3.40006000 3.97341100

H -3.26883800 -3.85714300 4.96715200

H -2.39925800 -3.87377900 3.41857300

H -2.96929400 -2.34109500 4.09769600

C -4.75285000 -5.10121400 3.03792800

H -4.09095400 -5.44549000 2.23806700

H -4.49451000 -5.64076000 3.95573100

H -5.78104900 -5.36508800 2.78174200

**TS2C-enamine-beta:**

C -2.76256900 2.50931200 0.12976600

C -1.99391200 1.43041100 0.44785000

C -1.02411100 1.48209300 1.60652400

C -1.45231400 2.51499900 2.64929700

C -1.73582600 3.86414100 1.99027500

C -2.80663100 3.71535700 0.92735400

H -0.89538700 0.49918400 2.06544900

H -3.41863000 2.44088500 -0.73805700

H -2.36336900 2.16808700 3.15340600

H -0.67469600 2.61259800 3.41357600

H -2.04610400 4.59997800 2.74064500

H -0.80848900 4.24647600 1.53684800

H -3.96562000 3.60205500 1.60098200

H -3.03124700 4.62771800 0.36823400

H -0.03935100 1.75052000 1.19635100

N -1.94475900 0.32072200 -0.39221100

H -2.30139100 0.47044300 -1.32870200

C -7.71619100 3.10517300 -0.30660500

C -8.36767500 1.87424800 0.31489700

C -7.27941100 0.94734100 0.84582600

C -6.35910800 1.62206200 1.87318400

C -6.81986800 3.86739700 0.67883200

H -7.71236300 0.05275400 1.30908900

H -9.05282200 2.16551100 1.12007000

H -8.97258600 1.35132300 -0.43381800

H -7.10059600 2.78519300 -1.15840500

H -8.47029100 3.80031800 -0.69458000

H -6.66351100 0.60910000 0.00167200

N -5.87603800 2.90438600 1.30152300

O -5.04550100 3.54037400 2.16357100

C -5.13531800 0.72919800 2.08728500

H -4.46547400 1.16149200 2.83647500

H -5.45262300 -0.26139800 2.43196200

H -4.58323800 0.61481700 1.15009600

C -7.06845900 1.81031900 3.22454500

H -7.18035300 0.84177600 3.72419500

H -6.46877000 2.46171600 3.86680700

H -8.06565500 2.24513700 3.12305100

C -5.97186500 4.87563500 -0.10181100

H -6.62160300 5.53139800 -0.69123500

H -5.37916700 5.49548300 0.57613900

H -5.29293700 4.35426000 -0.78539400

C -7.65431200 4.63912500 1.71423000

H -7.00611100 4.98319300 2.52515200

H -8.11178700 5.51621900 1.24300200

H -8.46041900 4.03896700 2.14262700

C -1.88545700 -1.02507600 0.00167900

C -2.28573900 -1.45595700 1.27347700

C -1.44440300 -1.97488300 -0.92968900

C -2.20932700 -2.80464100 1.60869400

H -2.68150900 -0.73798900 1.98547300

C -1.39684900 -3.32145600 -0.59291900

H -1.13288900 -1.64050400 -1.91582800

C -1.76741600 -3.74561300 0.68255100

H -2.52088300 -3.12137400 2.59978900

H -1.05524000 -4.04293200 -1.32928000

H -1.71902300 -4.79678500 0.94754000

**TS2C-enamine-delte:**

C -4.02767200 0.54287200 0.33450200

C -2.74385700 0.47307600 -0.03325900

C -1.93733500 1.69116100 -0.42438300

C -2.62402700 2.96363400 -0.00622000

C -4.10287000 2.98817500 -0.29134300

C -4.81594300 1.82299800 0.40555700

H -0.92757100 1.63110700 -0.00212500

H -4.53824600 -0.37530900 0.62241000

H -2.49683400 2.91875300 1.40926600

H -2.07214100 3.88615900 -0.20244300

H -4.54992900 3.94203800 0.00495500

H -4.24683300 2.89242600 -1.38064100

H -4.97833800 2.07301000 1.46386800

H -5.81200100 1.67967200 -0.02979900

H -1.79933200 1.67166100 -1.51906200

N -2.05874700 -0.75863400 -0.16789200

H -2.66758100 -1.54911700 -0.35049200

C 0.10560700 5.22589500 3.85164700

C -0.82957600 5.93856100 4.82356600

C -2.20218000 6.09867100 4.17749500

C -2.81829000 4.76070500 3.74395800

C -0.42418800 3.85528600 3.40519900

H -2.90403100 6.59657100 4.85703600

H -0.90907600 5.37734400 5.76238000

H -0.42112800 6.92118300 5.08324100

H 0.23267600 5.85374200 2.95957700

H 1.09949300 5.08266000 4.29206400

H -2.10104200 6.73671300 3.28933400

N -1.81438200 4.03925400 2.92596000

O -2.33289100 2.82939300 2.51868700

C -4.00831100 5.04206400 2.82145000

H -4.53104700 4.11663300 2.56199300

H -4.72026600 5.70996100 3.31814900

H -3.66390300 5.52238400 1.89914600

C -3.31726700 3.95462600 4.95352300

H -4.22765800 4.41388900 5.35463300

H -3.55678400 2.93262800 4.64584200

H -2.58699300 3.91069000 5.76493200

C 0.39909600 3.37718800 2.20548700

H 1.46626600 3.39898000 2.45191700

H 0.13843400 2.35184300 1.92894600

H 0.22787600 4.03001800 1.34278800

C -0.29644700 2.81380000 4.52921800

H -0.87860000 1.92084400 4.27956100

H 0.75352200 2.52077200 4.64336300

H -0.63833300 3.18804600 5.49729600

C -0.98396300 -1.07537900 0.68796900

C -0.66131900 -0.28847300 1.80075900

C -0.21677600 -2.21549300 0.41423300

C 0.43099700 -0.62489000 2.59743800

H -1.27674000 0.57285300 2.05343300

C 0.85197400 -2.55699600 1.23297200

H -0.46599500 -2.82172900 -0.45289100

C 1.19199200 -1.75783400 2.32453800

H 0.67325900 0.00173600 3.45184700

H 1.43247400 -3.44709900 1.00786400

H 2.03630700 -2.01886700 2.95447400

**TS2C-enamine-epsilon:**

C -3.33548200 0.56554300 0.27513300

C -2.08056600 0.08110500 0.43101500

C -1.03765100 0.85174400 1.10543200

C -1.46044400 2.14086000 1.76832300

C -2.55003000 2.84746400 0.96416300

C -3.75338900 1.92516100 0.76053300

H -0.31036800 0.26843400 1.67042500

H -4.07503900 -0.05891300 -0.22197500

H -1.83365500 1.92975300 2.78314500

H -0.58033600 2.78498300 1.89241700

H -2.85773900 3.77298000 1.46195800

H -2.14514200 3.12230000 -0.01678400

H -4.30958600 1.82476700 1.70565600

H -4.45645600 2.37364100 0.04857900

H -0.17282100 1.14372400 0.05847600

N -1.73704400 -1.15312500 -0.17865000

H -1.96753200 -1.18942600 -1.16394800

C 2.87147900 4.12091400 -0.54269900

C 4.17703100 3.34971700 -0.37484400

C 3.93478500 2.13651400 0.51737200

C 2.84127700 1.20176400 -0.01894300

C 1.74289400 3.26215600 -1.12941800

H 4.85272400 1.55097200 0.64703700

H 4.56728800 3.03770400 -1.35104800

H 4.93950400 3.99693600 0.07188600

H 2.54959300 4.48970000 0.44055900

H 3.00919300 4.99596300 -1.18895900

H 3.63069600 2.48730200 1.51277200

N 1.63923300 2.02116200 -0.32208100

O 0.62889300 1.23722200 -0.78858300

C 2.44108900 0.21883800 1.08394100

H 1.70848800 -0.50240300 0.71257100

H 3.32162700 -0.33412700 1.42852400

H 2.01286900 0.75740700 1.93686300

C 3.33197400 0.38906000 -1.22824400

H 4.01252000 -0.40046200 -0.89056100

H 2.47860100 -0.08687800 -1.72028700

H 3.86843700 0.99628700 -1.96145100

C 0.41558700 4.00814400 -0.97928900

H 0.48875800 4.99637500 -1.44577200

H -0.39422900 3.45721000 -1.46449400

H 0.16755600 4.14156400 0.07904900

C 1.96971800 2.98517400 -2.62526900

H 1.27569100 2.21190700 -2.96733000

H 1.77957400 3.89664700 -3.20277700

H 2.98844800 2.66094600 -2.84865000

C -0.60290600 -1.89904100 0.16509600

C -0.35048600 -2.24369900 1.50053500

C 0.27414800 -2.35263100 -0.82757900

C 0.77207400 -2.99464000 1.83080100

H -1.04792100 -1.92434800 2.26985400

C 1.38243200 -3.12363100 -0.49072900

H 0.08948700 -2.07781800 -1.86315300

C 1.64535200 -3.44155200 0.83993900

H 0.95633800 -3.24572000 2.87138300

H 2.05299200 -3.46254100 -1.27526000

H 2.51694900 -4.03276600 1.10175600

**TS2C-enamine-gamma:**

C -3.50197500 1.42554300 0.70173500

C -2.51412800 0.53668100 0.86961900

C -1.10315000 0.95329700 1.20097200

C -1.07266000 2.33196700 1.86317600

C -1.90801800 3.32902100 1.10588600

C -3.32248800 2.91588700 0.79298500

H -0.65268500 0.20992200 1.86648500

H -4.49931600 1.06518500 0.44911500

H -1.47023200 2.23885500 2.88743700

H -0.04001300 2.68387300 1.95657900

H -1.80400300 4.36518600 1.43575400

H -1.27664900 3.44065800 -0.17428700

H -4.00772800 3.33613000 1.54869800

H -3.62843200 3.38097800 -0.16083500

H -0.49596000 0.96349000 0.28534900

N -2.74930700 -0.86006000 0.78651500

H -3.70522000 -1.02794800 0.48133200

C -2.96531400 3.48663100 -4.10719100

C -2.86473900 4.95517500 -4.50823700

C -2.84505300 5.82235700 -3.25322000

C -1.70448900 5.45624200 -2.29325400

C -1.83293000 3.03821500 -3.17199500

H -2.75425000 6.88501600 -3.50807400

H -1.96401900 5.12864000 -5.10927500

H -3.71653300 5.22978200 -5.13988700

H -3.92072400 3.32771800 -3.58893400

H -2.96165100 2.83532400 -4.98941200

H -3.79756800 5.69534500 -2.72087700

N -1.77704000 3.99499700 -2.03766100

O -0.77748400 3.62333800 -1.16696100

C -1.94599200 6.15257300 -0.95067400

H -1.10510600 5.99267900 -0.27012200

H -2.06041800 7.23064500 -1.10624600

H -2.85864700 5.77163200 -0.47935600

C -0.34236100 5.92210900 -2.83414600

H -0.24647700 7.00600100 -2.70582900

H 0.46371300 5.43971900 -2.27381500

H -0.20731900 5.70629400 -3.89621900

C -2.18941300 1.66852600 -2.59059800

H -2.35184100 0.95446800 -3.40594900

H -1.38520600 1.28566300 -1.95585600

H -3.10260700 1.73115800 -1.99070000

C -0.49892100 2.90692800 -3.92577100

H 0.32204100 2.79496500 -3.21162300

H -0.52191900 2.01448700 -4.56078200

H -0.28521700 3.76201400 -4.57045800

C -1.83737800 -1.63167800 -0.00799800

C -0.74046800 -2.24965600 0.59298400

C -2.04651200 -1.78706000 -1.38074800

C 0.14805500 -2.99792100 -0.17511900

H -0.60464600 -2.15031100 1.66558400

C -1.16845800 -2.55187200 -2.14337700

H -2.90059700 -1.29808000 -1.84396400

C -0.06419400 -3.15338100 -1.54305000

H 1.00059700 -3.47336700 0.30058000

H -1.34258200 -2.67051500 -3.20875800

H 0.62472900 -3.74494700 -2.13800000

**TS2C-imine-alpha:**

C -3.68808600 1.03304100 0.49122900

C -2.44947900 0.32541300 0.17216300

C -1.17551400 1.14129900 0.29989700

C -1.17119900 2.01267200 1.56137300

C -2.42173500 2.88916400 1.62743300

C -3.68363300 2.02482400 1.63230200

H -0.29196100 0.50289000 0.25269600

H -3.79589500 1.91037500 -0.65109700

H -4.58886700 0.43048900 0.35666600

H -1.13065300 1.36787800 2.44974700

H -0.26706500 2.62999600 1.57337000

H -2.39907400 3.52349600 2.51968500

H -2.44484700 3.55629400 0.75531400

H -3.73729500 1.46964800 2.58301900

H -4.58460400 2.64622800 1.59090100

H -1.14566100 1.79873900 -0.58240300

N -2.54588500 -0.87472500 -0.28894500

C -5.71165300 1.19569700 -4.31297600

C -6.83080900 2.23019800 -4.39019900

C -7.23832900 2.64675500 -2.97982600

C -6.06790600 3.21150000 -2.16262100

C -4.48062400 1.70056400 -3.54673100

H -8.03454400 3.40003300 -3.00508900

H -6.50799800 3.10311500 -4.97025400

H -7.69282700 1.80850900 -4.91801800

H -6.09150300 0.30037600 -3.80332200

H -5.38950500 0.88742300 -5.31454900

H -7.63608100 1.76938000 -2.45273100

N -4.94682600 2.24897900 -2.25294800

O -3.89684400 2.65402700 -1.48887800

C -6.47351100 3.28460100 -0.68765300

H -5.70794000 3.79610500 -0.09803800

H -7.41180400 3.84033700 -0.58759700

H -6.62025900 2.27792700 -0.28142100

C -5.68394900 4.62450800 -2.63138000

H -6.44471600 5.34160100 -2.30359700

H -4.72679300 4.91564800 -2.18841800

H -5.60311300 4.70364800 -3.71797100

C -3.57057400 0.51137200 -3.22360900

H -3.34434300 -0.03681300 -4.14502800

H -2.62554200 0.84392500 -2.78329500

H -4.05689100 -0.16876200 -2.51681500

C -3.67880900 2.71865100 -4.37326800

H -2.94071300 3.21480300 -3.73598700

H -3.14353600 2.20144500 -5.17714200

H -4.30966200 3.48188400 -4.83492000

C -1.41647000 -1.65145300 -0.59751500

C -1.25743500 -2.13094300 -1.90406600

C -0.50272700 -2.05209100 0.38768600

C -0.18512500 -2.95643200 -2.22372300

H -1.98420100 -1.84330900 -2.65813500

C 0.55871500 -2.89102100 0.06321300

H -0.64598100 -1.70970400 1.40927900

C 0.72840200 -3.34188800 -1.24395300

H -0.06836800 -3.30930200 -3.24437300

H 1.25590200 -3.19468600 0.83883400

H 1.55827700 -3.99498500 -1.49476600

**TS2C-imine-beta:**

C -3.66194200 0.51135300 -0.92859200

C -2.33364300 0.04143200 -0.38097200

C -1.51829800 1.14101700 0.26488700

C -2.31852800 1.83042300 1.38488700

C -3.69677900 2.28925500 0.88996200

C -4.42550700 1.15665900 0.21053000

H -0.56697100 0.75986000 0.64207500

H -3.49085200 1.25884400 -1.72221200

H -4.20165500 -0.33319900 -1.36252500

H -2.45466600 1.12542700 2.21369700

H -1.74818900 2.68207100 1.76903700

H -4.28764900 2.68693600 1.72158000

H -3.56457200 3.11566600 0.17023400

H -4.40394000 0.13221700 1.22220300

H -5.50021300 1.27769300 0.05813600

H -1.29892600 1.88218900 -0.51679400

N -2.05830500 -1.20037700 -0.43707700

C -7.34764100 -1.20399500 3.93738400

C -7.52101100 -2.68455800 3.60962900

C -7.13377800 -2.93451300 2.15460000

C -5.69696800 -2.49654600 1.83561500

C -5.91969600 -0.70039200 3.68168500

H -7.23706200 -3.99480200 1.89546900

H -6.90887000 -3.30006500 4.27970300

H -8.56119800 -2.98428900 3.77715100

H -8.03631600 -0.62066500 3.31159100

H -7.60460600 -0.99794400 4.98329900

H -7.81821400 -2.37228000 1.50520100

N -5.54252500 -1.10014600 2.30694900

O -4.27792400 -0.64450800 2.01842700

C -5.50276100 -2.46858700 0.31652000

H -4.45970000 -2.26048800 0.05727100

H -5.77687500 -3.43867600 -0.11214000

H -6.14257500 -1.69908200 -0.13077900

C -4.66711700 -3.46698700 2.43422800

H -4.68215400 -4.40867400 1.87449900

H -3.66145900 -3.04359600 2.35033600

H -4.86207900 -3.70383400 3.48285400

C -5.91657200 0.83141800 3.70743100

H -6.35652100 1.19220200 4.64330300

H -4.89651600 1.22052700 3.63658800

H -6.50351700 1.22606600 2.87086500

C -4.94548500 -1.20431800 4.75916300

H -3.91362800 -1.02714500 4.44065100

H -5.11229400 -0.65724200 5.69381700

H -5.06338500 -2.26839000 4.97596300

C -0.93087000 -1.72463100 0.22725900

C 0.11760200 -2.28772000 -0.50555800

C -0.89906700 -1.77012900 1.62676400

C 1.19796900 -2.86266900 0.15638500

H 0.07396900 -2.26465200 -1.59033400

C 0.18009200 -2.35861000 2.27942100

H -1.74008800 -1.35549100 2.17891000

C 1.23497700 -2.90257700 1.54918400

H 2.01351900 -3.28839600 -0.42106000

H 0.19548600 -2.39143700 3.36506900

H 2.07599200 -3.36013600 2.06063600

**TS2C-imine-gamma:**

C -3.74503100 1.02333700 -0.18069800

C -2.34000500 0.47615300 -0.24996800

C -1.26751500 1.47306800 0.13274700

C -1.54495200 2.04063800 1.53819600

C -2.95702200 2.55945300 1.64272100

C -4.02815900 1.58303100 1.22642200

H -0.27080600 1.03142000 0.07672500

H -3.84293300 1.84242100 -0.90632500

H -4.45156500 0.23410700 -0.44717700

H -1.39588200 1.24031700 2.28241000

H -0.81965600 2.82608400 1.77243100

H -3.17835200 3.17408100 2.51918000

H -3.01889500 3.56906100 0.63133600

H -4.05372500 0.74472000 1.94247600

H -5.01749100 2.05047600 1.25336600

H -1.31521600 2.30225300 -0.58674000

N -2.17278300 -0.74153600 -0.58900500

C -4.90225700 7.33333500 0.82768400

C -3.77809900 8.34336100 0.61451200

C -2.44959400 7.72822300 1.04610200

C -2.12933000 6.42059100 0.30762700

C -4.66694200 6.01248200 0.08083000

H -1.62054400 8.42761500 0.88558300

H -3.73432000 8.65185300 -0.43687000

H -3.97592700 9.25049200 1.19560200

H -4.98137400 7.11348800 1.90075000

H -5.86728400 7.74382900 0.50757900

H -2.49214100 7.51485200 2.12254800

N -3.30965900 5.53462400 0.43323700

O -3.06036000 4.33203100 -0.18967600

C -0.97388600 5.71431800 1.02339400

H -0.64789400 4.83300800 0.46322500

H -0.12058900 6.39418800 1.11899300

H -1.28417600 5.40070700 2.02622300

C -1.70764600 6.68471500 -1.14706800

H -0.69282400 7.09728300 -1.16837500

H -1.70573700 5.74675100 -1.71029300

H -2.36101900 7.39580000 -1.65777800

C -5.65024900 4.96079900 0.60375300

H -6.67610000 5.33725700 0.53047300

H -5.58178200 4.03862000 0.01938400

H -5.43753600 4.73027700 1.65346000

C -4.89064800 6.17350200 -1.43153400

H -4.50320500 5.29759900 -1.96033000

H -5.96343700 6.25087100 -1.64035300

H -4.41207700 7.06638300 -1.84012600

C -0.89202200 -1.33174900 -0.60442100

C -0.32761800 -1.71547200 -1.82540700

C -0.21490000 -1.62495800 0.58489100

C 0.90610200 -2.35676800 -1.85401900

H -0.86784700 -1.50113000 -2.74262900

C 1.01411300 -2.27736400 0.54854500

H -0.66883200 -1.34627500 1.53229400

C 1.58285800 -2.64254000 -0.66927500

H 1.33877100 -2.64052800 -2.80898700

H 1.52891900 -2.50008000 1.47871200

H 2.54159400 -3.15068100 -0.69531500

**TS2C:**

C -2.99448000 -0.11590700 2.47424500

C -2.15223600 -0.40333300 1.42585800

C -1.89933000 0.63697700 0.34908900

C -2.26903600 2.05016300 0.79954200

C -3.67038400 2.07228800 1.40337100

C -3.72076700 1.18455000 2.64797600

H -0.85852200 0.60453600 0.02061000

H -3.10404200 -0.86127400 3.25295800

H -1.54504500 2.39527800 1.54961500

H -2.20081300 2.73547600 -0.05143000

H -3.97116200 3.09306100 1.66055600

H -4.38652600 1.70596200 0.65633800

H -3.27685600 1.72045300 3.50187700

H -4.76092500 0.98755800 2.94073700

H -2.50063000 0.35212400 -0.52752300

N -1.65500700 -1.66810900 1.32493100

H -2.57337200 -2.59384800 1.29600700

C -5.62337900 -2.94626200 4.03130600

C -6.92919300 -2.65147200 3.29678800

C -6.64727500 -1.80303400 2.05951100

C -5.63315300 -2.44574300 1.10374700

C -4.58540600 -3.64083700 3.14061900

H -7.56949600 -1.60199900 1.50152800

H -7.42794700 -3.58612200 3.01385500

H -7.61994100 -2.12170700 3.96148100

H -5.19433500 -1.99963900 4.38868700

H -5.80064900 -3.57459000 4.91214800

H -6.24348100 -0.83291900 2.38053100

N -4.44165300 -2.83538900 1.89832200

O -3.45486800 -3.31708500 1.10728100

C -5.18438500 -1.40748800 0.07339000

H -4.46021800 -1.83747500 -0.62401300

H -6.05214200 -1.05635600 -0.49466300

H -4.72297500 -0.55314400 0.57553600

C -6.24141600 -3.63547500 0.33831300

H -6.90445400 -3.26233000 -0.44931000

H -5.44342700 -4.21676400 -0.13267600

H -6.83073800 -4.29794700 0.97503100

C -3.22657800 -3.66351900 3.84670900

H -3.33206500 -4.16782200 4.81281800

H -2.48722700 -4.20509500 3.25177800

H -2.84461200 -2.65614700 4.02843500

C -4.97169600 -5.10242500 2.84785200

H -4.36453700 -5.48287400 2.02131900

H -4.76893200 -5.71595500 3.73221200

H -6.02654200 -5.22830700 2.59750100

C -0.61241100 -1.98595100 0.43818200

C 0.63244300 -1.34155800 0.52216300

C -0.77043300 -3.03287300 -0.48134200

C 1.68031500 -1.72281000 -0.30706700

H 0.77101900 -0.55841200 1.26269200

C 0.28623200 -3.40874700 -1.30455000

H -1.72933700 -3.53855700 -0.54887500

C 1.51442000 -2.75629600 -1.22798400

H 2.63711600 -1.21510700 -0.22470800

H 0.14438600 -4.21816500 -2.01495200

H 2.33577600 -3.05390800 -1.87207400

**TS3A:**

C -1.74322900 -1.72536400 0.56529000

C -1.28927300 -0.43322600 -0.08392300

C -2.19608500 0.45998100 -0.53964300

C -3.64659000 0.35370800 -0.33969400

C -4.13739500 -1.01270800 0.10694800

C -3.16509200 -1.61845600 1.12659500

H -1.85407200 1.34691700 -1.06901900

H -1.70966400 -2.55249300 -0.15922000

H -1.04094900 -1.99202500 1.36635200

H -3.46771800 1.21253600 1.25439500

H -5.13636600 -0.95108600 0.56219900

H -4.23110700 -1.70457100 -0.74997300

H -3.15332300 -0.98511700 2.02107400

H -3.51517400 -2.60848200 1.44029300

N 0.12666800 -0.23444400 -0.11206800

C 0.84726200 -1.28830400 -0.83161900

C 0.56870100 1.08616100 -0.53098900

C 2.35154500 -1.19045000 -0.59462900

H 0.63653300 -1.22407000 -1.91732600

H 0.48230000 -2.25998800 -0.48892600

C 2.06435400 1.26795900 -0.28224000

H 0.35474700 1.26173500 -1.60474700

H -0.00176600 1.82967800 0.03636500

H 2.86111600 -1.98247400 -1.15460300

H 2.54904500 -1.35745200 0.47169800

H 2.36797100 2.26735500 -0.61353700

H 2.25013100 1.20601100 0.79722100

C -7.78141900 2.66228400 -1.31754000

C -7.32585100 4.07206000 -1.68124800

C -6.02087100 4.38942600 -0.95923900

C -4.89015700 3.38369000 -1.25031700

C -6.73166400 1.58654200 -1.64303900

H -5.64938900 5.38543900 -1.22770300

H -7.20316900 4.17131500 -2.76595600

H -8.09306800 4.79572100 -1.38824700

H -7.99922900 2.61804800 -0.24185500

H -8.70377000 2.39468500 -1.84620400

H -6.20309500 4.39856300 0.12431000

N -5.43166700 1.99971600 -0.99866300

O -4.35165900 0.87759400 -1.39142500

C -3.76038100 3.63457000 -0.25113000

H -2.88970500 3.00778800 -0.44744800

H -3.45710500 4.68314700 -0.33708600

H -4.09163900 3.46726800 0.77621700

C -4.34108800 3.52625900 -2.67083800

H -3.69865100 4.41178200 -2.69746700

H -3.73642000 2.65571000 -2.93486200

H -5.11622500 3.66488500 -3.42555200

C -7.14984300 0.26248800 -1.00269000

H -8.16398900 0.02033400 -1.33600400

H -6.48223400 -0.54631500 -1.30572200

H -7.15780100 0.33814400 0.08893300

C -6.57682500 1.37700300 -3.15170100

H -5.66861300 0.81320900 -3.37272600

H -7.43473300 0.79180700 -3.49751300

H -6.56846700 2.30709800 -3.72066700

C 2.86969100 0.18740300 -1.00120300

H 3.93819700 0.28511100 -0.78045400

H 2.75132000 0.31109400 -2.08687500

H -5.54061600 1.87355600 0.03139900

O -3.26241200 1.48089900 2.22268000

H -2.33436700 1.22253500 2.32700400

O -4.63370900 3.61806700 3.10044400

H -4.72466100 3.59395100 4.06366300

H -3.93585300 2.95340300 2.88584800

O -6.03663800 1.67198700 1.77245800

H -5.76498200 2.46989900 2.28126800

H -5.37307400 1.01681500 2.04871700

**TS3B:**

C -3.53302100 0.01708000 1.31855800

C -2.11574800 0.35051100 1.53130900

C -1.48232100 1.57313900 0.89298800

C -2.46093800 2.66570600 0.44874400

C -3.69001900 2.05759700 -0.22179800

C -4.41163000 1.15638200 0.78193200

H -0.74495500 1.99333000 1.58565600

H -3.35385100 -0.85155400 0.36358300

H -2.78050300 3.25093200 1.32123300

H -1.93429900 3.35682000 -0.21950600

H -4.36903200 2.84517900 -0.56935300

H -3.39347600 1.47859500 -1.10790200

H -4.76204000 1.76923700 1.62536500

H -5.30459400 0.72108800 0.32349300

H -0.90629100 1.21862000 0.02498300

N -1.32785100 -0.53538900 2.10434600

C -6.42982200 -3.53386900 2.56906700

C -6.39124900 -3.75333700 4.07768200

C -4.98156100 -3.45972200 4.58099800

C -4.47994300 -2.04183900 4.22360100

C -5.97178100 -2.12550000 2.10894700

H -4.91945000 -3.56929100 5.67096000

H -7.12665100 -3.11818700 4.58674100

H -6.66556100 -4.78969300 4.31161400

H -5.78584700 -4.28095800 2.08670800

H -7.44072600 -3.69257200 2.17364700

H -4.28912300 -4.19558300 4.14962600

N -4.59754100 -1.87310100 2.71496700

O -4.19510100 -0.46988900 2.51248000

C -2.99316000 -1.97169000 4.61429100

H -2.58837400 -0.96768600 4.47214600

H -2.88997500 -2.22676600 5.67530900

H -2.39468600 -2.68545800 4.04167900

C -5.22086900 -0.97234800 5.06073900

H -4.87869300 -1.03745900 6.10037600

H -4.99575000 0.03191400 4.69526500

H -6.30414000 -1.09995400 5.07139400

C -5.84045900 -2.21056600 0.57857100

H -6.80973500 -2.51729100 0.16646900

H -5.57992500 -1.25904300 0.11551600

H -5.10023700 -2.95029200 0.26829700

C -7.03707700 -1.06043500 2.45111100

H -6.62083200 -0.05296900 2.38340500

H -7.86025100 -1.13295300 1.73120700

H -7.47090200 -1.18386100 3.44566600

C 0.14076100 -0.51469100 2.23674000

C 0.54420000 -0.91834900 3.66549100

C 0.77943700 -1.45300600 1.19247600

H 0.48888900 0.50483700 2.05525300

C 2.07315400 -0.96232000 3.81557700

H 0.12510000 -1.90884900 3.88758900

H 0.10577500 -0.21553400 4.38427400

C 2.30750400 -1.49702000 1.34961600

H 0.35786900 -2.45762500 1.32186200

H 0.50649700 -1.12251100 0.18254700

C 2.71847800 -1.88970500 2.77586700

H 2.33072700 -1.28646000 4.83121300

H 2.47973900 0.05343100 3.70019400

H 2.72792200 -2.20167000 0.62195100

H 2.72885500 -0.50980400 1.10804800

H 3.81088600 -1.86642200 2.87491900

H 2.40593500 -2.92564800 2.97034800

O -2.99699300 -1.79776700 -0.57010600

H -2.27747000 -1.41253600 -1.09723500

O -2.27838600 -3.14247600 1.57757400

H -3.16366300 -2.96251900 1.97504500

H -2.39402700 -2.69186700 0.67975300

O -3.51298800 -4.48853500 -0.90233700

H -3.08156700 -4.61011400 -0.04003100

H -3.40496800 -3.51331600 -1.01473300

H -1.73551200 -1.47557700 2.25449200

**TS3B’:**

C -3.81682000 0.53493400 1.06295200

C -2.57987500 -0.20129600 0.97664800

C -1.64835600 0.18932200 -0.17864500

C -1.77086300 1.65118500 -0.60713000

C -3.21675400 2.00385400 -0.92868500

C -4.07916100 1.83441200 0.32125000

H -0.61364500 -0.01549800 0.10718600

H -4.42903900 -0.61300600 -0.55243700

H -1.41733000 2.29503600 0.20930200

H -1.11844300 1.83940400 -1.46760000

H -3.29701200 3.03092800 -1.30129900

H -3.58196000 1.34724300 -1.72911800

H -3.89113600 2.69148300 0.99054100

H -5.14242100 1.91032400 0.04526500

H -1.85376800 -0.46371900 -1.04185000

N -2.30322700 -1.15375700 1.81893800

C -8.17631700 0.11834700 3.24400900

C -8.15646300 -1.32127300 3.74594100

C -7.31878200 -2.17065000 2.79837600

C -5.87046600 -1.67349400 2.62391500

C -6.77081400 0.72828400 3.11033900

H -7.26334100 -3.21114700 3.13849400

H -7.75846100 -1.37390000 4.76586600

H -9.17801900 -1.71249200 3.78932400

H -8.66520300 0.15106000 2.26067200

H -8.75305400 0.76718000 3.91359400

H -7.80093500 -2.18503900 1.81050800

N -5.92917800 -0.20612700 2.27366900

O -4.45474900 0.46381200 2.25385200

C -5.25223000 -2.41208500 1.43788100

H -4.19684000 -2.15031300 1.32361200

H -5.31929000 -3.48753300 1.63563200

H -5.80982100 -2.21477000 0.51877100

C -5.00467800 -1.91524800 3.85876400

H -4.85576400 -2.99605800 3.95314100

H -4.02715700 -1.44851000 3.71238800

H -5.45825200 -1.56406100 4.78657800

C -6.86795000 2.04853300 2.34414300

H -7.59581800 2.69148200 2.84911500

H -5.90513900 2.56269300 2.32566900

H -7.21183100 1.88342900 1.31823700

C -6.14155100 0.99189500 4.48236900

H -5.06348300 1.13675300 4.39820400

H -6.58147200 1.91205000 4.87998200

H -6.33964900 0.20039900 5.20621900

C -1.08258000 -1.92645000 1.70428200

C 0.03440000 -1.31159200 2.56299900

C -1.35902600 -3.35945300 2.17722100

H -0.70820100 -2.00016800 0.66704900

C 1.30414900 -2.16582400 2.56054800

H -0.35237100 -1.21455300 3.58708800

H 0.24971200 -0.29425900 2.21265600

C -0.09670300 -4.22395300 2.16898000

H -1.76890500 -3.30500200 3.19574900

H -2.13905400 -3.80303800 1.54622600

C 1.00787000 -3.59475300 3.02112200

H 2.07023300 -1.70987300 3.19850800

H 1.71859000 -2.19639100 1.54247700

H -0.32577900 -5.23448700 2.52687600

H 0.26496500 -4.33010800 1.13602000

H 1.91714200 -4.20585800 2.98292900

H 0.68186000 -3.57219100 4.07046200

H -6.25993800 -0.12014700 1.28970100

O -4.75807800 -0.89021600 -1.44866200

H -3.97221200 -0.88916900 -2.01486100

O -7.06817200 -2.41193400 -1.51531400

H -7.43637000 -2.44764300 -2.40973600

H -6.14097600 -2.11508400 -1.62637000

O -7.10930300 0.06625700 -0.31680600

H -7.38461700 -0.81601600 -0.64929000

H -6.36952200 0.27686300 -0.91266800

**TS3C:**

C -3.48714400 -0.41919500 0.98319400

C -2.05256100 -0.52244800 0.74082800

C -1.52513700 -0.06453900 -0.58721500

C -2.01532900 1.36505500 -0.90070200

C -3.53454700 1.46321800 -0.75037800

C -3.98417800 1.00988100 0.64441700

H -0.43550800 -0.12563800 -0.61663000

H -3.92322800 -1.19702400 0.06617300

H -1.52544400 2.07672800 -0.22216500

H -1.70163900 1.62748100 -1.91740200

H -3.86851300 2.49300400 -0.92730000

H -4.01431300 0.83025100 -1.50696700

H -3.60163300 1.70557200 1.40646000

H -5.07277800 1.04655900 0.71477500

H -1.92823100 -0.75612400 -1.33528100

N -1.26678400 -1.01874800 1.67106900

C -6.91354400 -2.86944300 2.98322900

C -6.47555700 -3.46243400 4.31519700

C -4.98868900 -3.77963500 4.23127500

C -4.10225900 -2.56261100 3.86328500

C -6.12214500 -1.60781400 2.55101300

H -4.61366700 -4.17700900 5.18232300

H -6.68426700 -2.77370100 5.14335100

H -7.04276800 -4.37807800 4.52374400

H -6.79148900 -3.63014500 2.20114800

H -7.97566600 -2.59659100 3.00112800

H -4.83391300 -4.56366700 3.47813100

N -4.64374500 -1.96384100 2.57853200

O -3.83615000 -0.74290800 2.34562500

C -2.69042500 -3.13461200 3.59935600

H -1.89446500 -2.41721600 3.82303300

H -2.51005100 -3.97987500 4.27262300

H -2.58324700 -3.49814000 2.57364000

C -4.00259500 -1.58554300 5.05607000

H -3.38728400 -2.04284100 5.84040700

H -3.52431800 -0.64929000 4.75614100

H -4.96934600 -1.34633700 5.50251100

C -6.55630400 -1.34581700 1.09516700

H -7.65096500 -1.38291800 1.04531900

H -6.25783300 -0.36241700 0.73237800

H -6.15050400 -2.10047700 0.42034300

C -6.51129800 -0.38370700 3.40551400

H -5.80786400 0.43810300 3.24956700

H -7.50695200 -0.03664200 3.10497200

H -6.55478300 -0.59744900 4.47570600

O -4.12670900 -1.98962400 -1.06509900

H -4.85833000 -1.67354800 -1.61765600

O -1.94968900 -3.59423700 -1.10442700

H -2.44858100 -4.08499300 -0.42252500

H -2.65381700 -2.96164700 -1.38637500

O -4.34907100 -4.15780500 0.48049400

H -4.38208200 -3.56723000 1.25909300

H -4.42050300 -3.47044300 -0.23917900

H -1.74934200 -1.19725100 2.54936400

C 0.10755000 -1.41956200 1.58261900

C 0.50746000 -2.34192600 0.61058400

C 1.01104000 -0.91549400 2.52317000

C 1.84675300 -2.73501800 0.57346500

H -0.23054400 -2.76051100 -0.07652000

C 2.34260000 -1.32709600 2.47783500

H 0.67372700 -0.20301900 3.27122100

C 2.76296100 -2.23200000 1.49971100

H 2.16829300 -3.45141900 -0.17738900

H 3.05066400 -0.93690200 3.20351700

H 3.80149700 -2.54945200 1.46532000

**TS3C’:**

C -3.79127600 0.67048000 1.09835800

C -2.56904800 -0.04871600 0.96983800

C -1.66908400 0.33162100 -0.21125800

C -1.84554200 1.78164900 -0.65813300

C -3.30867800 2.05595400 -0.98336100

C -4.16019200 1.88937400 0.27545200

H -0.63076100 0.13658400 0.07282700

H -4.26623000 -0.72567200 -0.56066800

H -1.51929800 2.45470600 0.14537800

H -1.20549400 1.98079100 -1.52485900

H -3.44265000 3.06317500 -1.39176000

H -3.64292700 1.35344400 -1.75856000

H -4.04506600 2.79151200 0.89791600

H -5.22538500 1.86028100 0.00120400

H -1.86866600 -0.32771100 -1.06835300

N -2.20964700 -0.94095400 1.86460200

C -8.15679100 0.06342700 3.23728400

C -8.06163300 -1.35141100 3.79653800

C -7.17142500 -2.18935000 2.88810900

C -5.75020300 -1.62098600 2.70397100

C -6.78530400 0.74497800 3.09003900

H -7.06142800 -3.21113200 3.26892100

H -7.66897800 -1.34070100 4.81967600

H -9.06020800 -1.79681400 3.84993900

H -8.63835500 0.02996700 2.25033300

H -8.77405400 0.70560100 3.87637000

H -7.64349400 -2.26850000 1.89851900

N -5.88505800 -0.17320600 2.29955100

O -4.42946300 0.58167400 2.27864200

C -5.08687500 -2.37022900 1.54928600

H -4.03958000 -2.07767000 1.44421000

H -5.11963000 -3.44160100 1.77250600

H -5.63496500 -2.21590800 0.61622000

C -4.88481000 -1.77348600 3.95322700

H -4.68202800 -2.84075900 4.08820100

H -3.93049400 -1.26282200 3.80243800

H -5.36469600 -1.41226300 4.86374400

C -6.94948200 2.02568800 2.27064500

H -7.71951300 2.64458100 2.74180800

H -6.01855700 2.59515900 2.24114100

H -7.27089600 1.80047500 1.24865600

C -6.18838600 1.09651600 4.45666000

H -5.12167100 1.31089300 4.37722900

H -6.69340100 1.99766700 4.81876500

H -6.34033500 0.31789700 5.20511400

H -6.19721600 -0.13954500 1.30636100

O -4.58247800 -1.03303100 -1.44043800

H -3.81453000 -0.95950400 -2.02629600

O -6.87586900 -2.61562200 -1.40597500

H -7.25211300 -2.75862400 -2.28618700

H -5.95370000 -2.32749500 -1.55798200

O -6.97634600 -0.08312700 -0.34266500

H -7.23369800 -0.98494800 -0.63492300

H -6.23980100 0.11490400 -0.94628500

C -1.13889700 -1.80038200 1.66034900

C -1.00820500 -2.60778900 0.51297400

C -0.18827300 -1.97101300 2.68449500

C 0.03832700 -3.51518200 0.38594200

H -1.76240200 -2.52983500 -0.26710300

C 0.85795400 -2.87504800 2.55109100

H -0.29473100 -1.36986100 3.58299500

C 0.98457700 -3.65280700 1.39952900

H 0.10989900 -4.12651100 -0.51010900

H 1.58189800 -2.97660600 3.35545000

H 1.80040100 -4.36197900 1.29971000

**TS4B:**

C -3.67808100 0.02614500 0.94023800

C -2.30453700 -0.31412100 0.72987600

C -1.29663500 0.68473500 0.23067800

C -1.72322300 2.12144200 0.55916200

C -3.18832800 2.37116900 0.20650900

C -4.09976000 1.47498500 1.04880600

H -0.30914900 0.48239100 0.65654200

H -1.57781000 2.30227800 1.63215700

H -1.06892400 2.81859200 0.02625700

H -3.44297600 3.42347300 0.36855300

H -3.34838000 2.16056500 -0.85900900

H -4.06506400 1.80408200 2.09942600

H -5.14299200 1.55753000 0.72757200

H -1.20012700 0.56687200 -0.85793600

N -1.99596100 -1.54875000 1.09296500

C -5.08513600 -4.17303600 3.75476500

C -5.32922000 -3.44838400 5.07641700

C -4.28850900 -2.34776200 5.26234000

C -4.23294900 -1.37319800 4.05604000

C -5.06522900 -3.19910400 2.54942400

H -4.47771900 -1.77351300 6.17954000

H -6.34054200 -3.02404000 5.09666500

H -5.27776500 -4.15778200 5.91076700

H -4.10970300 -4.67664300 3.80249200

H -5.84578900 -4.94619400 3.58109100

H -3.29788600 -2.81179900 5.36473700

N -4.08707800 -2.15543500 2.83640500

O -4.48014100 -0.93037800 1.15369600

C -2.96056600 -0.52602600 4.19784700

H -2.91490700 0.25501000 3.43141300

H -2.92874100 -0.03583400 5.17804600

H -2.07372700 -1.15975200 4.09650800

C -5.44364500 -0.41487000 4.10335800

H -5.27585300 0.36175200 4.85978500

H -5.57427400 0.06434200 3.12903300

H -6.37817700 -0.91875300 4.36149000

C -4.54779700 -3.95927600 1.32168400

H -5.19088900 -4.81588700 1.08890400

H -4.51418400 -3.29796900 0.45116700

H -3.53559100 -4.33622700 1.51372700

C -6.50831700 -2.73955900 2.24391000

H -6.50006100 -1.92823500 1.51475400

H -7.07870900 -3.57878100 1.82784500

H -7.04245900 -2.39948900 3.13360300

H -2.75443500 -2.00770000 1.67520600

C -0.72586900 -2.24258700 0.96572300

C -0.38201000 -2.92018700 2.29644000

C -0.81220100 -3.26722900 -0.17595700

H 0.06185000 -1.51983900 0.72132500

C 0.88466700 -3.77146900 2.18148900

H -1.23480800 -3.54562200 2.59971700

H -0.26059900 -2.15420600 3.07008000

C 0.46909000 -4.09610500 -0.27923500

H -1.66697800 -3.92816600 0.02018200

H -1.02161100 -2.74231200 -1.11494800

C 0.77589000 -4.79233500 1.04776600

H 1.07806600 -4.27344100 3.13531700

H 1.74433700 -3.11399400 1.98995000

H 0.37189500 -4.82953600 -1.08667100

H 1.30916000 -3.43935600 -0.54590500

H 1.70198200 -5.37209000 0.96978000

H -0.02990700 -5.50338400 1.27636700

**TS4C:**

C -3.78301100 -0.01735400 0.80059900

C -2.41657900 -0.33916000 0.55824500

C -1.41241100 0.68213500 0.10025000

C -1.86162900 2.10402700 0.45878300

C -3.31723800 2.34210200 0.06448200

C -4.23848800 1.42410100 0.86983400

H -0.43393400 0.46341100 0.54098700

H -1.75109000 2.25683000 1.54019800

H -1.19911100 2.82157900 -0.03522700

H -3.59398900 3.38784300 0.23144700

H -3.44164100 2.14299100 -1.00795500

H -4.25298700 1.75103900 1.92108100

H -5.27095000 1.48022700 0.51042600

H -1.29054500 0.60651400 -0.98942800

N -2.09897900 -1.57344600 0.96268100

C -4.86842400 -4.15017500 3.82555300

C -5.03118200 -3.39531300 5.14294900

C -4.01949200 -2.25519300 5.22105300

C -4.08572800 -1.31144000 3.98961800

C -4.97750500 -3.21173100 2.59582600

H -4.16367000 -1.65821300 6.13150500

H -6.05225200 -3.00539300 5.23366200

H -4.88789400 -4.07878900 5.98788200

H -3.87932100 -4.62797700 3.80682700

H -5.61799000 -4.94700100 3.73110500

H -3.00734600 -2.67983600 5.26626200

N -4.01193400 -2.13618400 2.79253900

O -4.56113200 -0.97252900 1.09967900

C -2.83186200 -0.42473700 4.00844000

H -2.87847000 0.34927800 3.23512000

H -2.73491200 0.07883200 4.97705700

H -1.93124200 -1.02578300 3.84027700

C -5.32041700 -0.39214400 4.10039400

H -5.11892900 0.41485200 4.81509800

H -5.54307000 0.04766900 3.12428700

H -6.21318000 -0.91675900 4.44850800

C -4.53163100 -3.98401600 1.34808800

H -5.14081700 -4.88466700 1.21194000

H -4.62820900 -3.35776400 0.45732200

H -3.48351100 -4.29212100 1.44221400

C -6.45143300 -2.79868800 2.39841100

H -6.52528600 -1.99929000 1.65995100

H -7.02248900 -3.66199500 2.03693200

H -6.92695700 -2.46319800 3.32230000

H -2.84019100 -1.97381800 1.64119300

C -0.86006500 -2.22038500 0.95432700

C -0.62001600 -3.14304800 1.98451100

C 0.10112100 -2.03820300 -0.04830100

C 0.58153200 -3.83653100 2.03159000

H -1.39020900 -3.29764600 2.73681300

C 1.29870600 -2.74349300 0.01006100

H -0.10040500 -1.37638600 -0.88319400

C 1.55059900 -3.63696000 1.04879900

H 0.75973200 -4.54156400 2.83781500

H 2.03636300 -2.59969500 -0.77366700

H 2.48668200 -4.18477400 1.08342200

**TS5B:**

C -2.26794500 1.87637300 1.56280500

C -1.90835200 0.65768700 0.70907000

C -2.20877900 0.65933800 -0.71219800

C -2.75883400 1.94266300 -1.31829000

C -3.78921400 2.55580100 -0.37553700

C -3.10014100 2.96514800 0.92225800

H -1.40341500 0.19521000 -1.29087500

H -1.96535100 2.67649500 -1.51808400

H -3.21680300 1.70355500 -2.28303300

H -4.26934900 3.43108900 -0.82269200

H -4.57602000 1.81956800 -0.16926000

H -2.40127000 3.79058800 0.72293400

H -3.79655600 3.32629100 1.68526300

H -3.04091500 -0.25370800 -0.73758100

N -1.39374700 -0.40681400 1.25385200

O -1.83380500 1.99062400 2.68698400

C -1.12864800 -0.73486900 2.66873100

C -2.43799300 -0.97651100 3.43048400

C -0.25656700 -1.99177100 2.69526100

H -0.58920200 0.10102400 3.11947700

C -2.13695000 -1.38180100 4.87583000

H -2.97987300 -1.77769900 2.91334000

H -3.05516600 -0.07367200 3.41039400

C 0.04970900 -2.39477100 4.13902200

H -0.80699800 -2.80160200 2.19865900

H 0.66821000 -1.81661600 2.13318100

C -1.24132200 -2.62129400 4.92836100

H -3.07615300 -1.56556700 5.40856700

H -1.63724800 -0.54894600 5.39027700

H 0.66834100 -3.29825100 4.14574700

H 0.63769700 -1.60209000 4.62233000

H -1.01099500 -2.88179300 5.96734300

H -1.78167500 -3.47241900 4.49254300

O -3.64857200 -1.57326800 -0.62392700

H -2.14697800 -2.14633500 -0.85767200

H -4.21788100 -1.74281300 -1.38701100

O -1.20388900 -2.42409700 -0.62111800

H -1.39953500 -3.18393200 -0.04350400

H -1.21238400 -1.19618000 0.57835200

O -3.05832200 -3.38040600 1.17771500

H -3.46239200 -2.71218900 0.54550300

H -3.71911800 -4.07837400 1.27801800

**TS5C:**

C -2.57748400 1.68340000 1.62501800

C -2.00242100 0.55743700 0.75067000

C -2.24922600 0.57991500 -0.67907900

C -2.58556700 1.92364600 -1.32185300

C -3.46366400 2.78643000 -0.42137800

C -2.76757100 3.00349000 0.92931000

H -1.46575100 0.03601400 -1.21709300

H -1.66540500 2.47716200 -1.55274300

H -3.08908800 1.74042900 -2.27593900

H -3.66138600 3.75583300 -0.88793900

H -4.43237300 2.29796800 -0.25925200

H -1.77600900 3.44612200 0.75492600

H -3.32766500 3.65965100 1.59904800

H -3.15335800 -0.23870500 -0.75270400

N -1.48008500 -0.50055700 1.30187800

O -2.91997600 1.45779600 2.75949200

O -3.82560800 -1.57161300 -0.70027200

H -2.39867800 -2.21673800 -0.71999200

H -4.31923700 -1.75299800 -1.51176700

O -1.49434100 -2.55800200 -0.37798700

H -1.80894500 -3.26804000 0.21275800

H -1.33739700 -1.34239900 0.64440200

O -3.61065500 -3.35826900 1.20159100

H -3.88811300 -2.66764400 0.52763500

H -4.29765200 -4.03701200 1.18091700

C -1.13531200 -0.71983200 2.67249100

C -0.25320400 0.13955500 3.32374700

C -1.62914900 -1.86258300 3.29579100

C 0.11665300 -0.13495700 4.63476600

H 0.14199900 1.00629300 2.80127400

C -1.24653800 -2.12603000 4.60855800

H -2.32261700 -2.50941700 2.76009800

C -0.37922700 -1.26732700 5.27921000

H 0.80121600 0.53156800 5.14984100

H -1.63329900 -3.00844000 5.10877900

H -0.08437400 -1.48163300 6.30167900

**TSB-nitrogen:**

C -3.15962000 0.11618500 1.83056000

C -1.91117500 -0.64765600 1.47155300

C -0.59931300 -0.06192700 1.96555600

C -0.81642800 1.12597600 2.90350500

C -1.73445300 2.15880400 2.24633300

C -3.09470400 1.53868600 1.93955400

H -0.00722200 -0.84516600 2.45372600

H -3.99032200 -0.23818300 1.22758000

H -1.27511000 0.76623700 3.83270700

H 0.15057900 1.56820500 3.16531000

H -1.82417300 3.03679900 2.90647400

H -1.21944500 2.52423800 1.33834200

H -4.15059500 2.52213200 2.84324400

H -3.65700600 2.02396600 1.13831100

H -0.01578600 0.27055700 1.09475900

N -2.05837500 -1.67793600 0.73444500

C -6.01055500 -0.42525800 5.90761000

C -6.74612500 -1.72983500 5.62458800

C -6.83682100 -1.93494300 4.11663400

C -5.46936800 -1.98155800 3.42232800

C -4.59911300 -0.37075300 5.30737400

H -7.35223300 -2.87126600 3.87482600

H -6.24277700 -2.57726400 6.10383500

H -7.75299300 -1.68316200 6.05102500

H -6.59654400 0.40576200 5.49576300

H -5.90942000 -0.25160200 6.98507400

H -7.42487300 -1.11700900 3.68026900

N -4.68950800 -0.71288600 3.80904000

O -3.41103600 -0.74968800 3.27128700

C -5.66356100 -1.94705500 1.90669000

H -4.72351900 -2.13468700 1.38005800

H -6.37268700 -2.73556700 1.63612100

H -6.08526800 -0.99188900 1.57542400

C -4.67327500 -3.23793000 3.76869400

H -5.09459200 -4.06924300 3.19500300

H -3.62994200 -3.11291000 3.46980800

H -4.72241100 -3.51198800 4.82281100

C -4.07609400 1.05879400 5.38664800

H -3.96865600 1.33535500 6.43976900

H -3.10868900 1.15572400 4.88781400

H -4.78243200 1.75082900 4.92407500

C -3.62215000 -1.30628600 6.01741100

H -2.72089500 -1.44661100 5.41702200

H -3.33519200 -0.83885800 6.96401900

H -4.05076100 -2.28052400 6.25402200

C -0.89743700 -2.43766800 0.29521200

C -0.77294100 -3.69571200 1.16540600

C -1.08117400 -2.82691600 -1.17447400

H 0.04067500 -1.86643100 0.38352000

C 0.36068200 -4.60323600 0.68299300

H -1.73021400 -4.23341400 1.11944300

H -0.62120200 -3.40241900 2.21159900

C 0.05507100 -3.72776500 -1.66230800

H -2.04245900 -3.35021300 -1.26792500

H -1.15107300 -1.91945100 -1.78517700

C 0.17896200 -4.97887400 -0.78959300

H 0.41025700 -5.50420400 1.30473200

H 1.31985100 -4.08078800 0.80520100

H -0.10878400 -4.00650300 -2.70914100

H 1.00188200 -3.17047100 -1.62833600

H 1.01451100 -5.60106200 -1.12950300

H -0.73314100 -5.58252800 -0.89611600

O -4.79038600 3.30711500 3.11897400

H -4.45285800 3.65693300 3.95714600

H -6.08680700 2.15360500 3.12880300

O -6.57992800 1.29824100 3.07091400

H -6.97837700 1.30023900 2.18820200

H -5.21485700 0.08671800 3.38141200

O -3.61589600 4.67705000 0.91568800

H -2.80486600 4.15119200 0.92485800

H -4.11894800 4.32752400 1.67500600

**TSB-oxygen:**

C -3.49271200 -0.77644800 1.44407300

C -2.02172000 -1.01449500 1.53017500

C -1.12630700 0.17570100 1.83058000

C -1.87443000 1.51160200 1.74977200

C -2.86748400 1.49198100 0.58847000

C -3.95579000 0.46529200 0.90776500

H -0.66174700 0.03968700 2.81592000

H -4.05056700 -1.69161700 1.26929500

H -2.43816200 1.68325100 2.67407800

H -1.15257900 2.32806700 1.65329000

H -3.32186400 2.48070600 0.45961300

H -2.33714200 1.26252800 -0.34709200

H -4.62283100 1.11016000 1.81987100

H -4.73201500 0.33007100 0.15059300

H -0.30803300 0.18013100 1.10115000

N -1.64548400 -2.19550000 1.23235600

C -6.76656100 -3.17899000 3.97659600

C -6.18612000 -3.90855000 5.18275100

C -4.68632400 -4.09626400 4.98296700

C -3.93903700 -2.77402600 4.74107400

C -6.10514000 -1.81373800 3.72057000

H -4.22957600 -4.58875500 5.84956000

H -6.38763600 -3.34762500 6.10313300

H -6.67131000 -4.88341300 5.30043900

H -6.62501900 -3.80085700 3.08287100

H -7.84362800 -3.01542700 4.09495000

H -4.52211700 -4.74557700 4.11321100

N -4.64378500 -2.08126000 3.62930500

O -3.91911600 -0.92421500 3.26910300

C -2.53159400 -3.10688800 4.24089000

H -1.90450500 -2.21147900 4.18196500

H -2.05606000 -3.81217400 4.93163300

H -2.58230900 -3.55498600 3.24505700

C -3.81883900 -1.96875600 6.04761500

H -3.01524600 -2.39507600 6.65811800

H -3.57132500 -0.92028400 5.85451200

H -4.72967300 -1.99288300 6.64926600

C -6.57962500 -1.29860700 2.35887200

H -7.67455500 -1.27985800 2.34616500

H -6.23071600 -0.27854200 2.17371500

H -6.24246000 -1.95688200 1.55131800

C -6.53191400 -0.78891300 4.77929500

H -6.03824400 0.17254000 4.63265800

H -7.60703400 -0.60385600 4.68070000

H -6.34786300 -1.11755700 5.80336900

C -0.22521700 -2.50818700 1.14040200

C 0.01312200 -3.92354800 1.67504300

C 0.19688200 -2.41694200 -0.33425300

H 0.39558700 -1.81089500 1.72444400

C 1.46869600 -4.35442600 1.48359900

H -0.65759600 -4.60733800 1.13774500

H -0.26405800 -3.96720900 2.73417500

C 1.65222400 -2.84713800 -0.52691100

H -0.46813100 -3.06874800 -0.91630100

H 0.04005800 -1.39412400 -0.69838400

C 1.88674700 -4.25853300 0.01507400

H 1.60603300 -5.37553600 1.85536800

H 2.12165000 -3.70821500 2.08707800

H 1.91721800 -2.79453300 -1.58852400

H 2.31176500 -2.14365700 0.00021700

H 2.93884000 -4.54071400 -0.10194800

H 1.29603000 -4.97278600 -0.57483600

O -5.18680100 1.94034300 2.70528700

H -5.05526800 2.83444500 2.35394800

H -4.42458100 1.69784700 3.84845800

O -3.96188700 1.21968400 4.68684500

H -3.07473100 1.59110900 4.80252900

H -3.97683000 -0.12106200 3.91674700

O -6.54293400 2.59221400 5.12345200

H -5.75104100 2.20677600 5.52821800

H -6.39783200 2.37598600 4.18379500
